# Scalable, Chemoselective Nickel Electrocatalytic Sulfinylation of Aryl Halides with SO_2_

**DOI:** 10.1002/anie.202208080

**Published:** 2022-08-03

**Authors:** Terry Shing‐Bong Lou, Yu Kawamata, Tamara Ewing, Guillermo A. Correa‐Otero, Michael R. Collins, Phil S. Baran

**Affiliations:** ^1^ Department of Chemistry Scripps Research 10550 North Torrey Pines Road La Jolla CA 92037 USA; ^2^ Oncology Medicinal Chemistry Department Pfizer Pharmaceuticals 10770 Science Center Drive San Diego CA 92121 USA

**Keywords:** Chemoselectivity, Electrochemistry, Fluoride, Nickel, Sulfur

## Abstract

Simple access to aryl sulfinates from aryl iodides and bromides is reported using an inexpensive Ni‐electrocatalytic protocol. The reaction exhibits a broad scope, uses stock solution of simple SO_2_ as sulfur source, and can be scaled up in batch and recycle flow settings. The limitations of this reaction are clearly shown and put into context by benchmarking with state‐of‐the‐art Pd‐based methods.

## Graphical abstract

Enabled by electrochemistry, the conversion of aryl halides to sulfinates using an inexpensive Ni catalyst at room temperature is reported. The use of SO_2_ stock solution at controlled concentration represents a convenient sulfur source. The sulfinate intermediates can be derivatized into sulfonyl fluorides, sulfonamide, and sulfone in a one‐pot fashion. This reaction exhibits a broad substrate scope and can be easily scaled up in batch and flow.

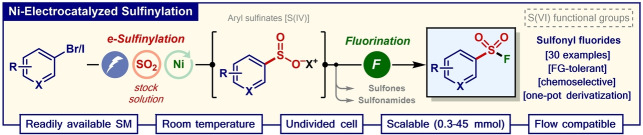

## Introduction

**Figure 1:**
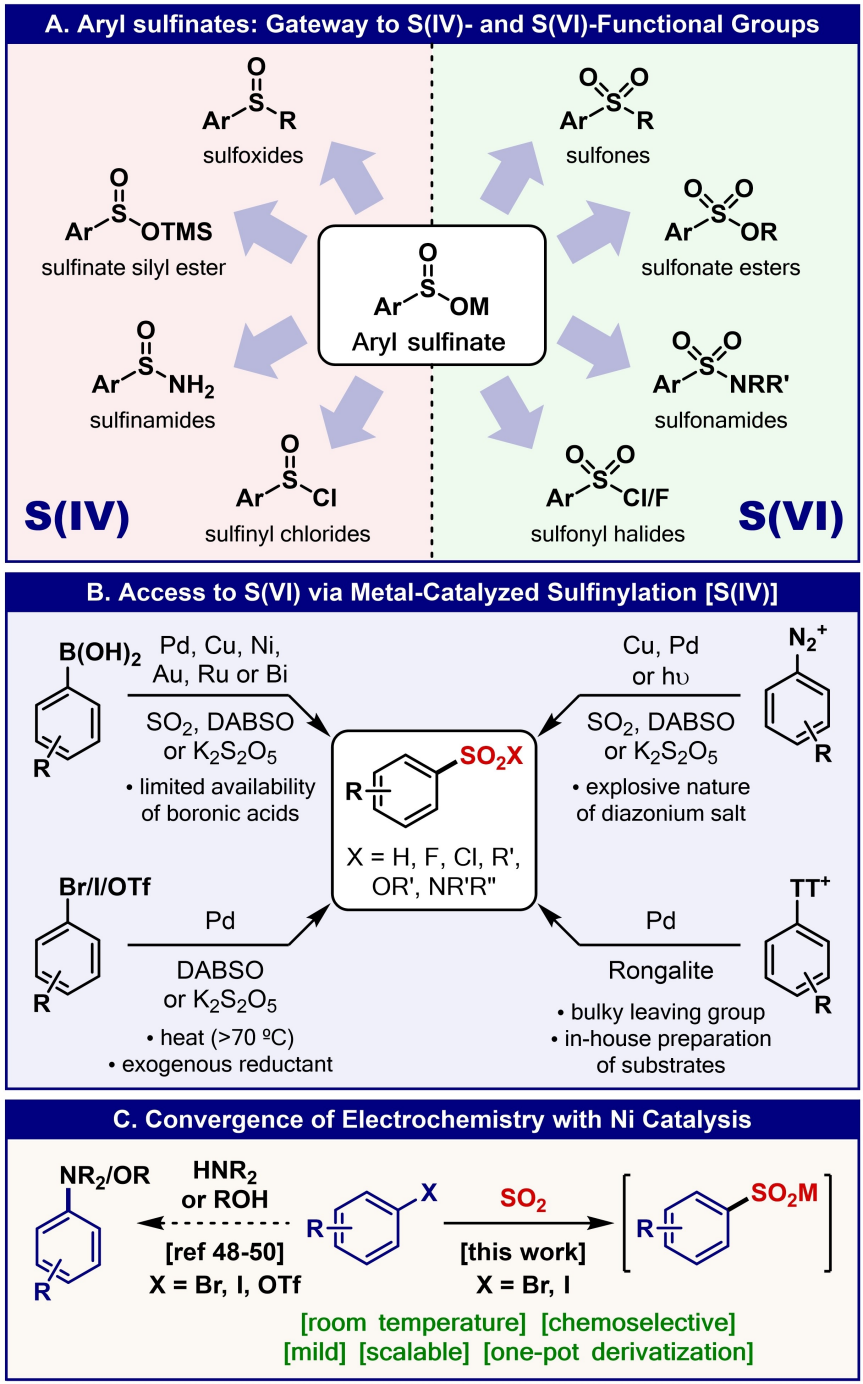
A) Aryl sulfinates as the gateway to various S^IV^ and S^VI^ functional groups. B) Established metal‐catalyzed sulfinylation for the formation of various sulfonyl‐containing compounds. C) Recent advancement of Ni catalysis enabled by electrochemistry.

Sulfur‐containing functional groups in the S^VI^‐oxidation state appended to arenes are widespread in products of societal importance such as medicines, agrochemicals, and other functional materials.[^1^, ^2^, ^3^, ^4^, ^5^, ^6^, ^7^, ^8^] One of the most convenient precursors to these essential derivatives is aryl sulfinates, existing in the S^IV^‐oxidation state. These can be considered as versatile gateway intermediates to numerous desirable functionalities in either the S^IV^ or S^VI^ oxidation state such as sulfoxides, sulfinate esters, sulfinamides, sulfinyl chlorides, sulfonamides, sulfonyl halides, sulfones, and sulfonate esters (Figure 1A).[^9^, ^10^, ^11^, ^12^, ^13^, ^14^, ^15^, ^16^] As such, there is burgeoning interest in accessing aryl sulfinates directly from readily available aryl precursors through canonical cross‐coupling logic (Figure 1B).[^17^, ^18^, ^19^, ^20^, ^21^] While SO_2_ is ambiphilic in nature, it commonly reacts as an electrophile with carbanion equivalents.[^22^, ^23^, ^24^, ^25^] For instance, boronic acids have been employed using a variety of transition metals (e.g. Pd^II^,[^26^, ^27^, ^28^, ^29^] Cu^I^,[^30^, ^31^, ^32^] Cu^II^,^[33]^ Ni^II^,^[14]^ Au^I^,[^34^, ^35^] and Ru^II[36]^) or even Bi‐catalysis^[37]^ along with various SO_2_ sources such as DABSO, K_2_S_2_O_5_, or SO_2_ itself. Unlike aryl boronic acids, the use of aryl halides is akin to a cross‐electrophile coupling, which is more challenging as this requires exogenous reductant to balance the redox. The use of aryl halides under Pd‐catalysis was pioneered by the groups of Pfizer,^[38]^ Willis,[^39^, ^40^, ^41^, ^42^] and Ball^[43]^ to access a range of useful S^VI^ derivatives. All of these processes proceed through the intermediacy of aryl sulfinates and require in situ oxidation to their S^VI^ analogs. Aryl diazonium species have also been employed in a Sandmeyer process in flow using a solution of SO_2_.^[44]^ Finally, electrochemical C−H sulfonylation has been developed by the Waldvogel group to convert electron rich arenes to valuable S^VI^ derivatives.[^45^, ^46^, ^47^] Building on prior work from this lab on the development of Ni‐electrocatalytic amination[^48^, ^49^] and etherification^[50]^ of aryl halides we now present an analogously useful method for aryl sulfinate synthesis using simple SO_2_ as a sulfur source (Figure 1C). The current method is benchmarked against known Pd‐based methods, exhibits good chemoselectivity across a range of substrates, and can be easily scaled up in both batch and recycle flow settings.

## Results and Discussion

The development of a workable electrochemical aryl sulfinate synthesis from the corresponding halide proved challenging stemming from the redox active nature of SO_2_ combined with its ability to interfere with Ni‐catalysis. Unlike Ni‐electrocatalytic amination or etherification reactions, which are redox neutral, sulfinylation is a net reductive process. By employing an electrochemical reaction mode, exogenous reductant can be replaced with a sacrificial anode. Preliminary analysis of all constituents of the reaction (Ni catalyst, aryl halide **1**, SO_2_) revealed that SO_2_ would be subject to preferential reduction with a mild reduction potential of only −0.78 V vs. Ag/AgCl in CH_3_CN. This is particularly problematic because the reduced radical anion could dimerize to form dithionite dianion, or form aggregates with SO_2_.^[51]^ Although super‐stoichiometric SO_2_ could be employed to overcome this, excessive SO_2_ can poison Ni catalysts. Various SO_2_ surrogates have been developed and used over the last decade, however, their limited solubility in organic solvents at room temperature makes them poor candidates for use in a simple electrochemical setup. Despite these concerns, optimization was deliberately focused on the use of SO_2_ in a stock solution (in dimethylacetamide, DMA) of controlled concentration (as determined by iodometry) due to its inexpensive nature and high solubility. In support of this choice, the use of SO_2_ solutions in electrochemical reactions have been documented in as early as 1960s,[^52^, ^53^, ^54^, ^55^] and has recently been reviewed by Waldvogel.^[25]^ Furthermore, SO_2_ readily forms Lewis adducts with amines, so a sterically hindered tertiary amine could in principle form an amine‐SO_2_ complex in situ to overcome issues of aggregation and catalyst poisoning.[^56^, ^57^]

We began by evaluating the known Pd‐catalyzed sulfinylation of aryl halides on substrate **1** (Table 1A). These conditions often require sterically hindered, electron‐rich phosphine ligands, such as AmPhos[^41^, ^42^] and PAd_2_Bu[^39^, ^43^] which impede their effectiveness with *ortho*‐substituted and electron‐rich substrates. Not surprisingly, various reported Pd‐catalyzed methods delivered the corresponding sulfinate **2** in only modest yields. Finally, no sulfinate product was detected using a Ni catalyst which was optimized for boronic acids.^[14]^

**Table 1:**
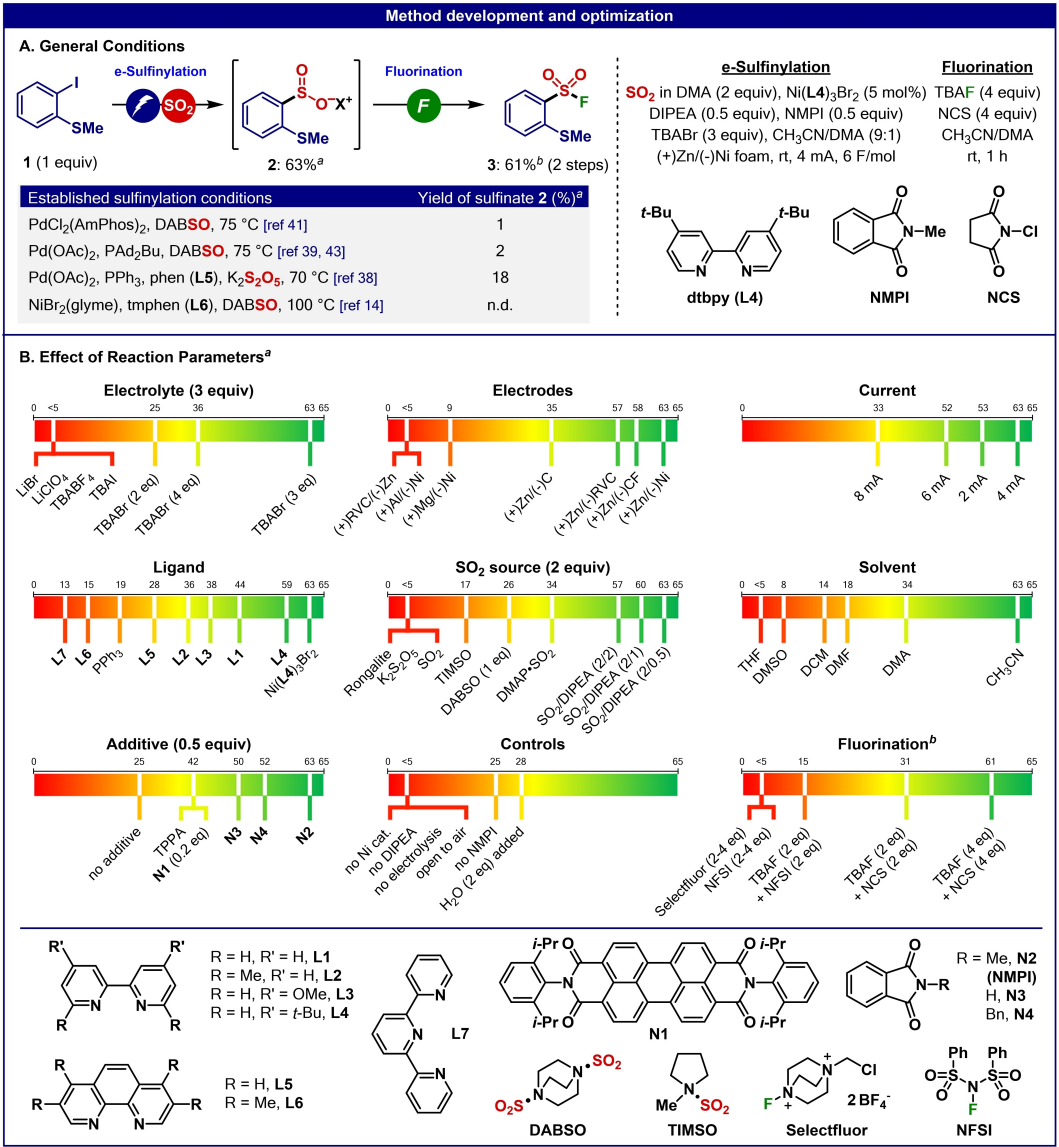
A) Optimized conditions for one‐pot sulfonyl fluoride synthesis through electrochemical sulfinylation and fluorination, and comparison of sulfinate **2** formation from 2‐iodothioanisole (**1**) with established conditions. B) Evaluation of the effect of various reaction parameters.

[a] HPLC yields of sulfinate **2**. [b] Yields of sulfonyl fluoride **3** over twp steps determined by ^19^F NMR spectroscopy.

With those background results in hand, attention turned to the development of a workable Ni‐electrocatalytic solution. In the fully optimized system, 5 mol % of preformed Ni(dtbpy)_3_Br_2_ complex was used as a catalyst, and a stock solution of SO_2_ in DMA (2 equiv) was used as the S source. The stock solution can be conveniently prepared in‐house by simply bubbling SO_2_ gas into the solvent, and can be stored in a sealed vessel at 4 °C. In fact, a 5.6 M solution still retained 84 % of its SO_2_ content after repeated usage for 6 months. *N*,*N*‐Diisopropylethylamine (DIPEA) and *N*‐methylphthalimide (NMPI) (0.5 equiv each) were added to the reaction, and 3 equiv of tetra‐*n*‐butylammonium bromide (TBABr) was employed as the electrolyte. The electrolysis was performed in acetonitrile, using a standard ElectraSyn 2.0 potentiostat, with Zn anode and Ni foam cathode, at 4 mA (or 5 mA cm^−2^) for 6 F/mol. The reaction was conducted over a nitrogen atmosphere without the need for a glovebox. To simplify the isolation and quantification of the e‐sulfinylation reaction, sulfinates were converted in situ into the stable sulfonyl fluorides. Sulfonyl fluorides are a versatile functional group, capable of SuFEx Click chemistry,[^58^, ^59^] acting as a covalent warhead in protein inhibitors,[^60^, ^61^, ^62^] and as synthetic intermediates to other S^VI^ derivatives.[^42^, ^43^, ^63^, ^64^, ^65^, ^66^, ^67^, ^68^, ^69^] The fluorination was optimized and performed in the same pot after sulfinylation, using inexpensive TBAF and *N*‐chlorosuccinimide (NCS).

The optimization of an electrocatalytic reaction is not as complicated as it may seem.[^70^, ^71^] Although numerous variables not associated with canonical organic methodology need to be evaluated such as electrode, electrolyte, and current, the results are independently optimizable. Thus, keeping all variables constant, the individual variables can be systemically optimized and combined to give higher yields. A graphical summary is presented in Table 1B to show representative reaction permutations from the entire optimization process. To highlight a few important factors, it was found that 3 equiv of TBABr was needed to produce sulfinate in a good yield. While SO_2_ is capable to form Lewis adduct with amines in a 1 : 1 ratio, 0.5 equiv of DIPEA with 2 equiv of SO_2_ elicited a marginally higher yield. This is likely due to the instability of DIPEA‐SO_2_ adduct^[72]^—allowing a rapid equilibrium between the Lewis adduct and free SO_2_, while preventing aggregation between SO_2_ and its radical anion. CH_3_CN was found to be the best solvent, but when an acetonitrile stock solution of SO_2_ was used, an impermeable black passivation layer was formed on the Zn anode, which eventually insulated the electrode and halted the electrolysis. In contrast, when a DMA stock solution was employed, deposits forming at the electrode peeled off leading to the choice of a 9 : 1 CH_3_CN/DMA mixture as reaction solvent. Control experiments have shown that all of the components are necessary for a successful sulfinylation. Omission of the Ni catalyst or DIPEA resulted in no sulfinate formation. Electricity is also needed for the reaction to proceed, as no sulfinate was formed when stirred with Zn dust. Inspired by a recent report on the use of perylene bisimide mediators^[73]^ in facilitating reductive reactions, such compounds were also screened (see Supporting Information for list). From that study the addition of NMPI was found beneficial, where its absence led to a drastic drop in yield.

When Selectfluor or NFSI was employed in fluorination, which is commonly employed to convert sulfinates into sulfonyl fluorides, little sulfonyl fluoride **3** was detected. This is likely due to the high concentration of other halides (Br and I) in the pot which can compete with sulfinate for oxidation. As increasing the amount of the fluorinating agents up to 4 equiv was found to be ineffective, more cost‐effective options were pursued using TBAF as a fluoride source and NCS as an oxidant (4 equiv each). This delivered the sulfonyl fluoride **3** in 61 % NMR yield (60 % isolated) over two steps, which is comparable to the sulfinylation HPLC yield (63 %).

With the established optimized conditions for aryl sulfinate synthesis, the substrate scope was explored. To improve the handling of the sulfinylated product, all sulfinates were fluorinated and isolated as sulfonyl fluorides (Table 2). In general, the Ni‐catalyzed electrochemical sulfinylation, i.e. e‐sulfinylation, works well on aryl iodides with various electron‐donating and withdrawing groups, with a great tolerance to different substituents, particularly at the *ortho*‐position (a notable limitation in Pd‐catalyzed sulfinylation). Electron‐deficient aryl bromides were also successfully converted into sulfinates and isolated as sulfonyl fluorides. Aryl sulfonyl fluorides substituted with one or more electron‐donating groups, such as methyl groups (**4** and **5**), methoxy ethers (**6**–**8**), a 1,3‐benzodioxole (**9**), a 2‐amino group (**10**), and a *tert*‐butyl carbamate (**11**) were isolated in modest to good yields. Electron‐neutral substrates, such as 2‐thioanisole (**3**) and 2‐biphenyl (**12**) were also converted effectively. For comparison, established Pd conditions were also performed on these substrates, yet these usually only resulted in poor to modest yields, as determined by ^19^F NMR spectroscopy. In particular, the *ortho*‐substituted thioanisole (**3**) and aniline (**10**) were poor substrates in Pd‐catalysis, possibly due to their coordination to Pd. In contrast, this was not the case in e‐sulfinylation, as both substrates were able to deliver sulfonyl fluorides in good 60 % and 51 % yields, respectively. Next, a series of sulfonyl fluorides were synthesized electrolytically from electron‐deficient aryl iodides, showing good tolerance to a range of functional groups, including reductively labile halides, such as F (**13**), Cl (**14**), and Br (**15**), amide (**16**), trifluoromethyl (**17**), 2‐methylbenzothiazole (**18**), ketone (**19**), ester (**20**), carboxylic acid (**21**), and nitrile (**22**, **23**). Whilst state‐of‐the‐art Pd catalysis performed comparably, and sometimes better, on these substrates, the synthesis of **19** and **23** were unsuccessful, likely due to their Lewis basicity. Notably, when 1‐bromo‐4‐iodobenzene was employed, excellent chemoselectivity was achieved with Ni‐catalyzed e‐sulfinylation—only the iodide was converted into sulfinate, then sulfonyl fluoride **15**. The C−Br bond remained intact and no 4‐iodobenzenesulfonyl fluoride **15 a** was detected; in contrast, poor selectivity between the bromide and iodide was observed under both benchmarked Pd conditions, resulting in a mixture of **15** and **15 a**, which have similar polarity and hence are difficult to separate.

**Table 2:**
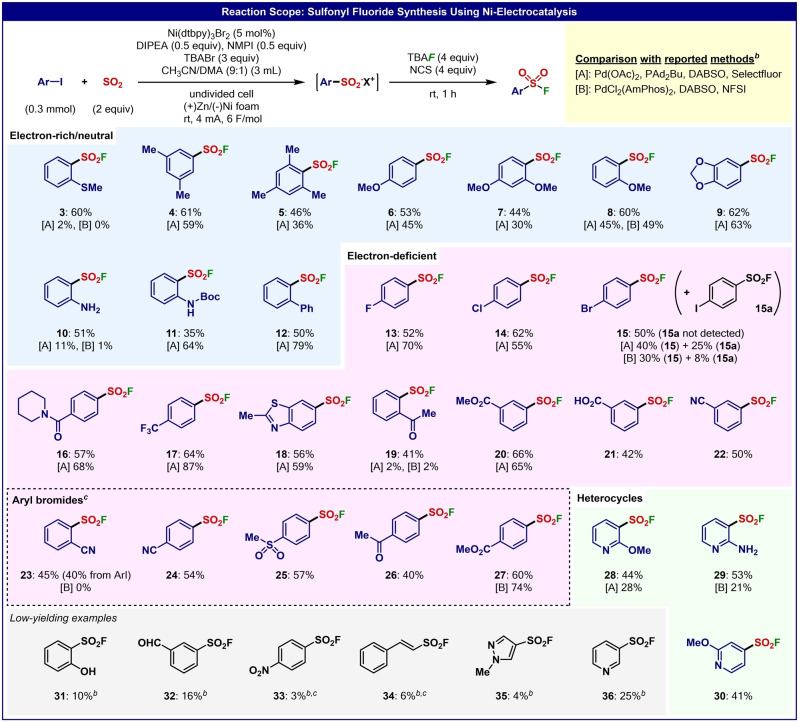
Reaction scope of sulfonyl fluoride using Ni‐electrocatalysis.

[a] Isolated yields presented. [b] ^19^F NMR yields. [c] NFSI (1.5 equiv) was used in fluorination in place of TBAF and NCS.

In addition, the viability of electrochemical sulfinylation on aryl bromides bearing an electron‐withdrawing group was showcased, including nitrile (**23**, **24**), sulfone (**25**), ketone (**26**), and ester (**27**) functionalities. In these cases, 2 equiv of NFSI was used for the fluorination step. Furthermore, the preparation of substituted 3‐ and 4‐pyridine sulfonyl fluorides from the corresponding iodides were also successful in the presence of an electron‐donating group, such as a 2‐methoxy (**28**, **30**) and a 2‐amino group (**29**), despite 3‐iodopyridine only delivered **36** in 25 % yield. These were found to be challenging under Pd conditions as well, which only delivered **28** and **29** in modest 28 % and 21 % yield, respectively. Meanwhile, a few unsuccessful examples are listed to acknowledge the limitations of the current method, which gave low yields in the presence of a phenol (**31**), an aldehyde (**32**) or a nitro group (**33**). Only trace amount of sulfonyl fluorides was detected when β‐iodostyrene (**34**) and 4‐iodo‐1‐methylpyrazole (**35**) were employed.

**Figure 2:**
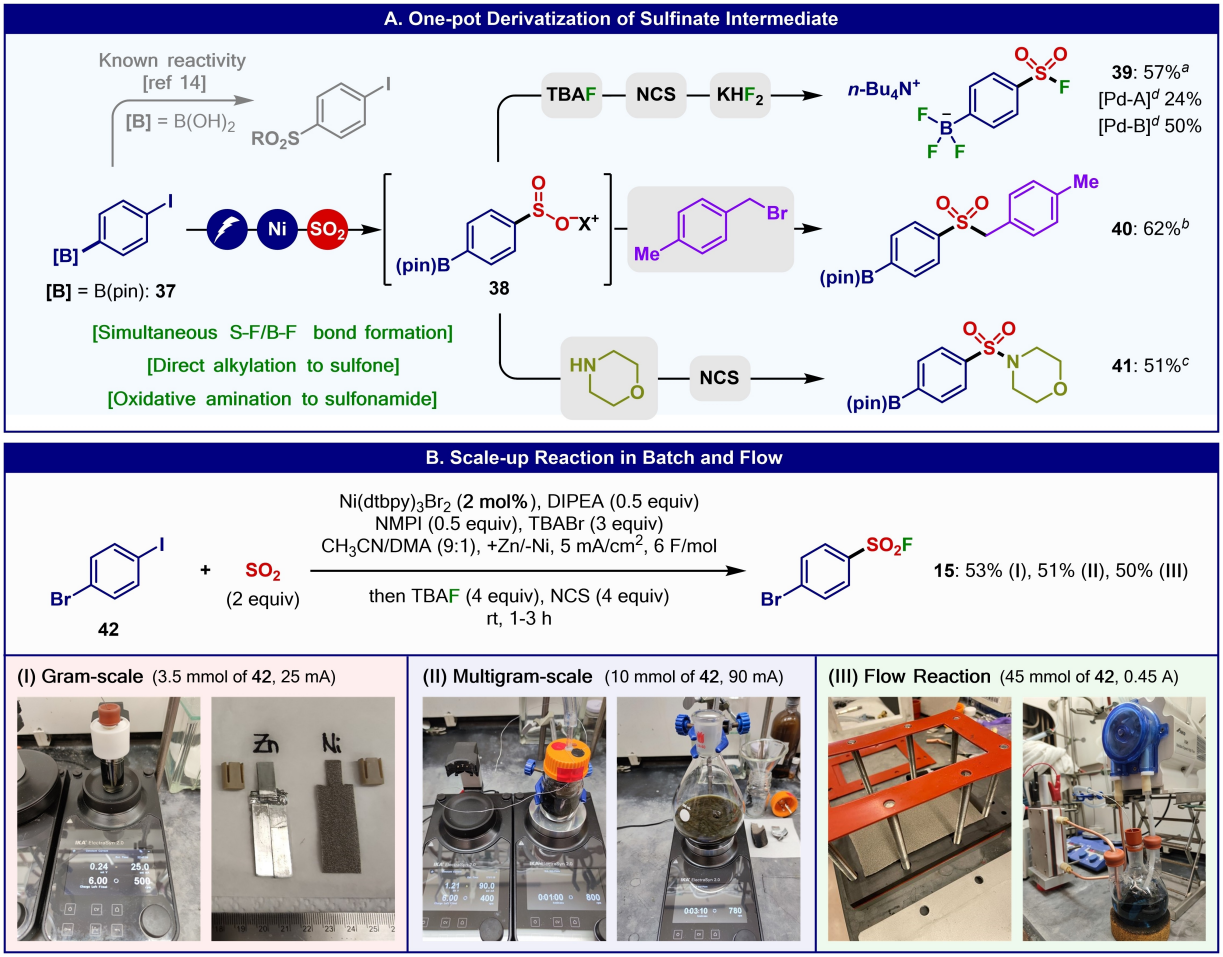
A) One‐pot derivatization of aryl sulfinate **38** into various sulfonyl functional groups. B) Scale‐up reaction in I) gram scale using ElectraSyn 2.0 setup, II) multigram scale in capped reagent bottle, and III) decagram scale in recycle flow system. [a] TBAF (4 equiv), NCS (4 equiv), rt, 0.5 h, then aq. KHF_2_ (4 equiv), rt, 2 h. [b] 4‐MeC_6_H_4_CH_2_Br (2 equiv), rt, 1 h. [c] Morpholine (3 equiv), NCS (4 equiv), rt, 1 h. [d] Refer to Table 2 and Supporting Information for comparison conditions, followed by the addition of aq. KHF_2_ (4 equiv).

To further highlight the excellent selectivity and versatility of the current method, 4‐iodophenylboronic acid pinacol ester (**37**) was subjected to e‐sulfinylation, as depicted in Figure 2A. Although typical fluorination of sulfinate **38** resulted in partial substitution on the boronic ester, the addition of KHF_2_ (4 equiv) enabled its full conversion into trifluoroborate—a versatile functionality in cross‐coupling reactions. The preservation of the C−B bond enabled efficient access to the bifunctional sulfonyl fluoride **39** in a 57 % overall isolated yield. Meanwhile, the two benchmarked Pd conditions reported by the Ball and Willis laboratories, with the addition of KHF_2_, delivered **39** in 24 % and 50 %, respectively. Whilst the sulfinylation of aryl boronic acids using metal catalysis has been well documented, for instance, 4‐iodophenylboronic acid was converted into 4‐iodobenzene sulfinate using a Ni catalyst,^[14]^ this method demonstrates an orthogonal reactivity for accessing sulfinylated product carrying a cross‐coupling handle. The capability of further coupling reactivities would make **39** another candidate as SuFEx building block for the quick installation of arylsulfonyl fluoride module.^[74]^ On the other hand, sulfinate **38** can be alkylated by 4‐methylbenzyl bromide to produce sulfone **40** directly in 62 %. Finally, a sulfonamide, a functional group commonly found in active pharmaceutical ingredients, could also be accessed easily through a sulfonyl chloride generated in situ, as evidenced by the formation of **41** in 51 %. All these transformations of sulfinate **38** were conveniently performed in a one‐pot manner—no additional work up is needed prior to the derivatizations, hence proving the compatibility of various ingredients in e‐sulfinylation with these downstream reactions.

In order to capitalize on the superior functional group tolerance and chemoselectivity over canonical methods using expensive Pd catalysts, the scalability of the developed method was illustrated in Figure 2B, as exemplified by the sulfinylation of 1‐bromo‐4‐iodobenzene **42**. In fact, scaling up the electrochemical reaction was straightforward in this case. A brief optimization revealed that the Ni catalyst loading can be lowered to 2 mol % without sacrificing efficiency or yield. Current density, i.e. current per unit area of each of the electrode, should be maintained at 5 mA cm^−2^. A larger immersed area of electrodes enabled a higher current to be applied, and therefore shortened the time required for the electrolysis. The concentration with respect to the substrate has little impact on the reaction when above 0.1 M, which allows flexible adjustment of the solvent volume to achieve the optimum current density. To demonstrate the ease of scaling up the reaction, 1.0 g of substrate was subjected to a 20‐mL vial and an ElectraSyn 2.0 potentiostat was used (Figure 2B–I). With larger pieces of Zn plate and Ni foam used as electrodes, 25 mA of current was applied and the electrolysis was completed in 23 h with 6 F mol^−1^ of electricity passed. This produced the sulfonyl fluoride **15** in 53 %, which is comparable to a 0.3 mmol‐scale reaction. Alternatively, scaling up in batch was also achieved using a capped reagent bottle at 10‐mmol scale, forming sulfonyl fluoride **15** in a similar 51 % yield (Figure 2B‐II). With more room to accommodate even larger pieces of Zn and Ni foam electrodes, a constant current of 90 mA was applied, and the e‐sulfinylation was completed in 18 h. Furthermore, a 45‐mmol scale reaction (12.7 g of aryl iodide **42**) was also performed using a recycle flow system (Figure 2B‐III). While the use of copper tubing to connect the flow reactor with the pump and the reservoir was found crucial for a successful electrolysis, no further optimization was needed. By applying a constant current of 0.45 A for 16 h, 50 % of sulfonyl fluoride **15** was yielded upon fluorination (see Supporting Information for details). This evidenced the compatibility of using SO_2_ as a reagent with electrochemistry and flow chemistry, as well as the workability of scaling up the reaction in both batch and flow settings.

**Figure 3:**
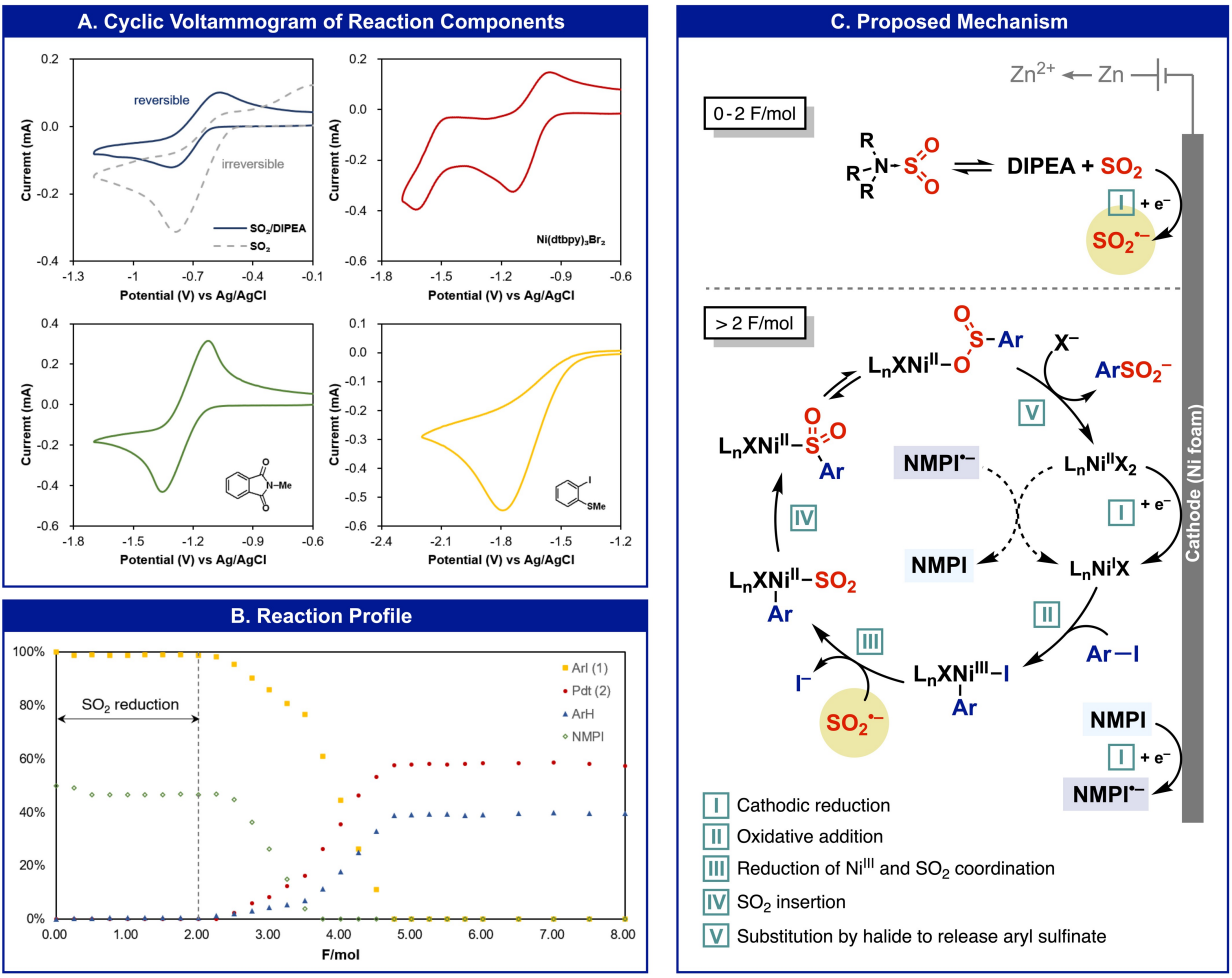
A) Cyclic voltammograms of SO_2_ alone (grey dotted line), SO_2_ with DIPEA (blue line), Ni(dtbpy)_3_Br_2_ (red line), NMPI (green line), and 2‐iodoanisole (**1**) (yellow line), which were taken with *n*‐Bu_4_NBF_4_ [0.1 M] as electrolyte in CH_3_CN, glassy carbon working electrode, Ag/AgCl as reference, at 298 K. Scan rate=200 mV s^−1^. B) Reaction profile of the e‐sulfinylation of 2‐iodoanisole (**1**) over a course of 8 F/mol, with the amount of aryl iodide **1** (yellow squares), sulfinate product **2** (red dots), anisole (blue triangles), and NMPI (green diamonds) determined by HPLC using 4,4′‐di‐*tert*‐butylbiphenyl as internal standard. C) Proposed mechanism for e‐sulfinylation.

Since the e‐sulfinylation is a net reductive process, its mechanism is clearly distinct from the established electrochemical amination[^48^, ^49^] and etherification^[50]^ reactions. A cyclic voltammetry study (Figure 3A) revealed an irreversible reduction of SO_2_ at −0.78 V against Ag/AgCl standard electrode in CH_3_CN, and a reversible reduction at similar potential when DIPEA was present, indicating that the amine coordination to SO_2_ profoundly changes its redox behavior. Since diminished yield of sulfinate **2** was observed without DIPEA, long‐lived SO_2_^.−^ might be beneficial for an efficient reaction. When the reaction was monitored by HPLC throughout its entire course (Figure 3B), little conversion of aryl iodide **1** and NMPI was observed during the first 2 F mol^−1^. As 2 equiv of SO_2_ was employed, this is in agreement with the preferential reduction of SO_2_ over other constituents in the reaction. Therefore, it is proposed that the plausible mechanism would first involve the single‐electron reduction of SO_2_, followed by the reduction of the Ni^II^ complex (Figure 3C, step I). The low‐valent Ni species would undergo oxidative addition with aryl iodide (step II), and SO_2_^.−^ would substitute iodide and reduce the Ni center (step III). The SO_2_‐coordinated aryl Ni complex then undergoes SO_2_ insertion to give an Ni‐sulfinate species (step IV), where substitution by a halide releases aryl sulfinate product from the Ni center (step V). While a workable yield of sulfinate **2** was recorded without NMPI, it is thought that NMPI acted as both a reductive mediator[^75^, ^76^] and an overcharging protector, which mitigates the unproductive cathodic reduction of substrates (despite the reduction potential of aryl iodides varies with substituents, NMPI has a distinctively less negative potential in general). Although an Ni^I/III/II^ system is proposed herein, we cannot exclude the involvement of other possible low‐valent Ni‐catalytic cycles.

## Conclusion

In conclusion, the first Ni‐catalyzed sulfinylation of aryl halides has been enabled at room temperature using electrochemistry. The employment of an inexpensive catalyst and SO_2_ stock solution not only provides a mild and economical alternative to canonical Pd catalysis, but also represents a chemoselective method with admirable tolerance to various functional groups that is currently absent in other methods. The scalability and practicality of this reaction were demonstrated in both batch and flow, with convenient access to various S^VI^ functional groups, such as sulfonyl fluorides, sulfone and sulfonamide, in a one‐pot fashion.

## Experimental Section

Experimental procedures, tables of optimization, cyclic voltammograms, cathodic potential‐time graph, reaction profile, and characterization of compounds.

## Conflict of interest

The authors declare no conflict of interest.

1

## Supporting information

As a service to our authors and readers, this journal provides supporting information supplied by the authors. Such materials are peer reviewed and may be re‐organized for online delivery, but are not copy‐edited or typeset. Technical support issues arising from supporting information (other than missing files) should be addressed to the authors.

Supporting Information

## Data Availability

The data that support the findings of this study are available in the Supporting Information of this article.
