# Supplementary material for: Scalable, Chemoselective Nickel Electrocatalytic Sulfinylation of Aryl Halides with SO2
Source: Angew Chem Int Ed Engl. 2022 Aug 3;61(37):e202208080. doi: 10.1002/anie.202208080 (PMC9452475; doi:10.1002/anie.202208080)

Angewandte Chemie
Eine Zeitschrift der Gesellschaft Deutscher Chemiker

Supporting Information

Scalable, Chemoselective Nickel Electrocatalytic Sulfinylation of Aryl Halides with SO_2

*T. S.-B. Lou, Y. Kawamata, T. Ewing, G. A. Correa-Otero, M. R. Collins, P. S. Baran**

Supporting Information

Table of Contents

1. General Considerations S2
2. Preparation of $Ni(dtbpy)_3Br_2$ catalyst S3
3. Preparation and Titration of SO_2 Solution S4
4. Tables of Optimization S5
5. Cyclic Voltammograms S11
6. Cathodic Potential-Time (V-t) Graph S15
7. Reaction Profile S16
8. General Procedures and Graphical Guide S17
9. Procedure for Gram-Scale Reaction S24
10. Procedure for Multigram-Scale Reaction S30
11. Procedure for e-Sulfonylation in Flow S33
12. Reaction Scope of Sulfonyl Fluorides S38
13. Experimental Procedures and Characterization Data S39
14. Comparison Conditions S55
15. Trouble Shooting & FAQ S56
16. References S60
17. NMR Spectra S61

1. General Considerations

Chemicals were purchased at the highest commercial quality and used without further purification. Reactions were performed with continuous magnetic stirring, under an atmosphere of nitrogen (passed through a Drierite® filled tube), unless otherwise specified. All glassware was dried in an oven (150 °C, > 1 h) and allowed to cool under a flow of nitrogen prior to use. Thin-layer chromatography (TLC) was performed using 0.25 mm E. Merck Silica plates (60F-254), using short-wave UV light, I_2, and $KMnO_4$ for visualization. Flash column chromatography was performed using E. Merck silica gel (60, particle size 0.040–0.063 mm).

Nuclear magnetic resonance (NMR) spectra were recorded on JEOL ECZ400R 400 MHz spectrometer. Chemical shifts (δ) are reported in parts per million (ppm) and referenced to the residual solvent peak(s) ($CHCl_3$ at 7.26 ppm ^{1}H NMR, 77.16 ppm ^{13}C NMR) or $CFCl_3$ (at 0 ppm ^{19}F NMR). Coupling constants (J) are reported in Hertz (Hz). Proton-decoupled spectra are denoted as {^{1}H}. Signal multiplicities are denoted as: s, singlet; d, doublet; t, triplet; q, quartet; quint, quintet; sext, sextet; dd, doublet of doublets; dt, doublet of triplets; m, multiplet; br, broad; app., apparent. Mass measurements for high-resolution mass spec (HRMS) were performed on a Waters Xevo G2-XS TOF calibrated against sodium formate clusters and using a LeuEnk lockmass. Expected monoisotopic masses were calculated using MassLynx 4.1 and the m/z values for calibrant and lockmass were MassLynx-default values.

2. Preparation of $Ni(dtbpy)_3Br_2$ catalyst

To a solution of $NiBr_2 \cdot 3H_2O$ (0.54 g, 2.0 mmol, 1.0 equiv) in MeOH (3 mL) was added 4,4'-di-*tert*-butyl-2,2'-bipyridine (1.61 g, 6.0 mmol, 3.0 equiv). The mixture was stirred at ambient temperature for 10 min, and was concentrated *in vacuo*. Acetone was then added to the residue and concentrated *in vacuo*, and this was repeated twice. The resulting solid was then triturated with acetone for three time, and then dried under vacuum to yield the titled catalyst as a pink solid (1.65 g, 81%).

Alternatively, a solution of $NiBr_2$ (1.75 g, 8.00 mmol) and 4,4'-di-*tert*-butyl-2,2'-bipyridine (6.50 g, 24.0 mmol) in MeOH (20 mL) was heated under reflux for 30 min. The boiling hot solution was then filtered quickly and concentrated *in vacuo*. Acetone was then added, and the solid was filtered, washed with acetone for three times, and dried under vacuum to afford the titled catalyst (8.18 g, 99%).

3. Preparation and Titration of SO_2 Solution

Preparation

Following a modified procedure of Waldvogel, an oven-dried culture tube sealed with septum was charged with degassed anhydrous DMA under N_2. A balloon filled with SO_2 gas was attached with a needle, and bubbled through the solvent with a needle vent. The balloon was refilled until DMA was saturated with SO_2 (ca. 7–8 M; see *Trouble Shooting & FAQ* – Q1 for further details). The resulting SO_2 solution was then titrated (optional) and diluted to desired concentration. For convenient use in e-sulfinylation, ca. 2 M SO_2 in DMA was prepared, and the exact concentration was determined by titration.

Graphical guide:

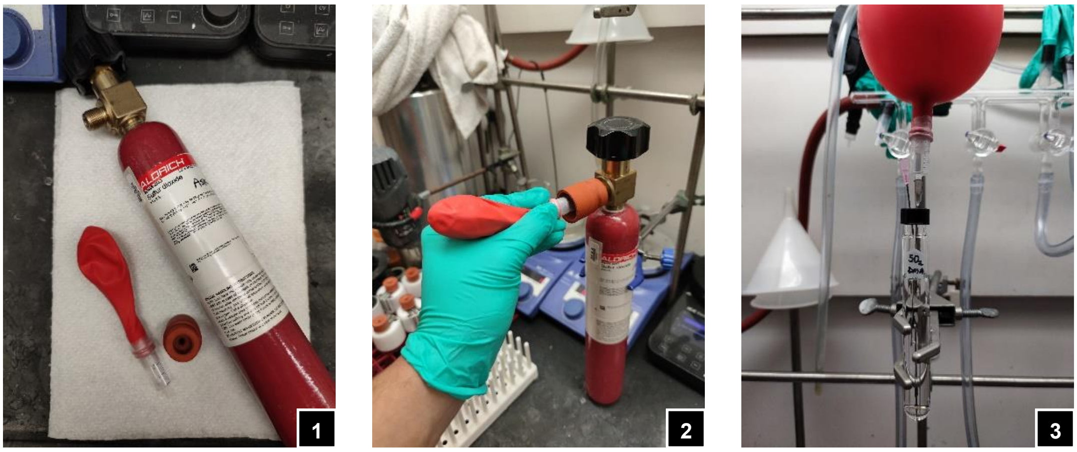

Left: SO_2 gas cylinder, double-layer balloon, and a B24 rubber septum punctured with a hole (diameter ca. 4 mm). **Center:** Filling balloon with SO_2 gas. **Right:** Bubbling SO_2 through DMA with a needle vent.

Titration

For the titration of a ca. 2 M SO_2 solution, a round-bottom flask was charged with *accurately weighed* I_2 (ca. 65 mg), KI (100 mg) and deionized water (10 mL). SO_2 solution (0.10 mL) was then added, and stirred for 5 min. The resulting solution was then titrated with a freshly prepared aq. $Na_2S_2O_3$ solution (0.2 M) as titrant to determine the amount of excess I_2, with the end point marked by the color change from brown to colorless (an optional starch solution can be added for better visualization).

4. Tables of Optimization

Optimization of sulfinylation was performed on 2-iodothioanisole (**1**), yields of sulfinate **2**, proto-dehalogenated side product **2a** and unreacted aryl iodide **1** were determined by HPLC using 4,4'-di-*tert*-butylbiphenyl as internal standard:

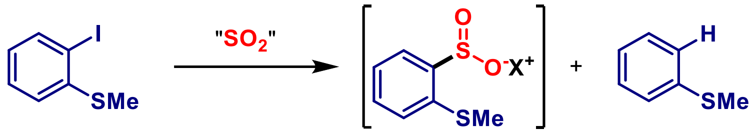

1 **2** **2a**

4.1. Initial evaluation of known conditions and reaction development

Entry	Conditions	**2** (%)	**2a** (%)	**1** (%)
1[1]	ArI (0.3 mmol), $PdCl_2(AmPhos)_2$ (5 mol%), DABSO (0.6 equiv), Et_3N (3 equiv), *i*-PrOH (1 mL), 75 °C, 24 h	1	6	89
2[2]	ArI (0.3 mmol), $Pd(OAc)_2$ (5 mol%), PAd_2Bu (8 mol%), DABSO (0.6 equiv), Et_3N (3 equiv), *i*-PrOH (1.5 mL), 75 °C, 16 h	2	3	90
3[3]	ArI (0.3 mmol), $Pd(OAc)_2$ (5 mol%), PPh_3 (15 mol%), phen (15 mol%), $K_2S_2O_5$ (2 equiv), TBAB (1.1 equiv), HCO_2Na (2.2 equiv), DMSO (0.8 mL), 70 °C, 16 h	18	25	34
4[4]	ArI (0.2 mmol), $NiBr_2$(glyme) (10 mol%), tmphen (10 mol%), DABSO (0.6 equiv), LiO*t*-Bu (1 equiv), DMI (1 mL), 100 °C, 16 h	0	1	99
5[5]	ArI (0.2 mmol), $Ni(dtbpy)_3Cl_2$ (10 mol%), DABSO (1 equiv), TBAB (0.2 M, 3 equiv), DMA (3 mL), (+)RVC/(-)Ni, 4 mA, 6 F/mol	0	0	100
6	ArI (0.2 mmol), $Ni(dtbpy)_3$**Br_2** (10 mol%), **TIMSO (2 equiv)**, TBAB (0.2 M, 3 equiv), DMA (3 mL), (+)RVC/(-)Ni, 4 mA, 6 F/mol	0	0	100
7	As entry 6, (+)Zn/(-)Ni instead	5	92	0
8	As entry 7, SO_2 in DMA (2 equiv) and DIPEA (0.5 equiv) instead of TIMSO	16	77	0
9	As entry 8, NMPI (0.5 equiv) added	25	72	0
10	As entry 9, CH_3CN as solvent	47	51	0
11	As entry 10, 0.3 mmol scale	58	42	0
12	As entry 11, 5 mol% $Ni(dtbpy)_3Br_2$	63	36	0

4.2. Electrolyte

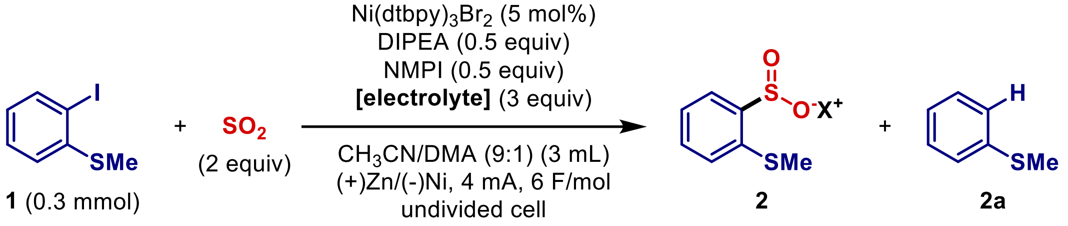

Entry	Electrolyte	**2** (%)	**2a** (%)	**1** (%)
1	*n*-Bu_4NBr	63	36	0
2	*n*-Bu_4NI	0	86	1
3	*n*-Bu_4NBF_4	0	79	7
4	LiBr	0	85	0
5	$LiClO_4$	0	41	48
6	*n*-Bu_4NBr (2 equiv)	25	58	0
7	*n*-Bu_4NBr (4 equiv)	36	48	0

Discussion:

Sodium and potassium salts tend to have poor solubility in the reaction solvent and hence excluded from the screening. It is believed that bromide is capable to coordinate to Ni catalyst and mitigate its over-reduction into Ni black. Meanwhile, *n*-Bu_4NBr likely improved the solubility and the retention of SO_2.[6]

4.3. Electrodes

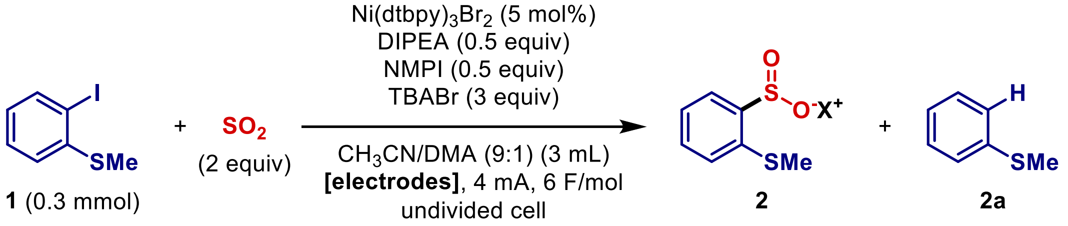

Entry	Anode (+)	Cathode (-)	**2** (%)	**2a** (%)	**1** (%)
1	Al	Ni foam	0	61	7
2	Mg	Ni foam	9	4	0
3	Zn	Ni foam	63	36	0
4	Zn	C (graphite)	35	60	0
5	Zn	RVC	57	41	0
6	Zn	CF	58	39	0

RVC = reticulated vitreous carbon. CF = carbon felt.

4.4. Current

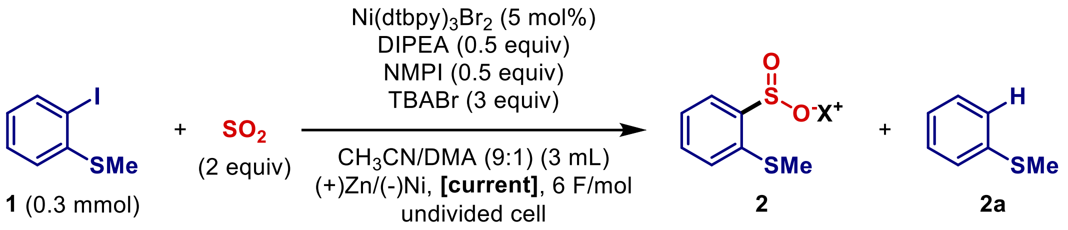

Entry	Current	2 (%)	2a (%)	1 (%)
1	2.0 mA	53	46	0
2	4.0 mA	63	36	0
3	6.0 mA	52	37	0
4	8.0 mA	33	59	0

Discussion:

Higher current resulted in higher terminal voltage and tends to increase direct reduction of aryl iodides and formation of proto-dehalogenated product.

4.5. Ligand

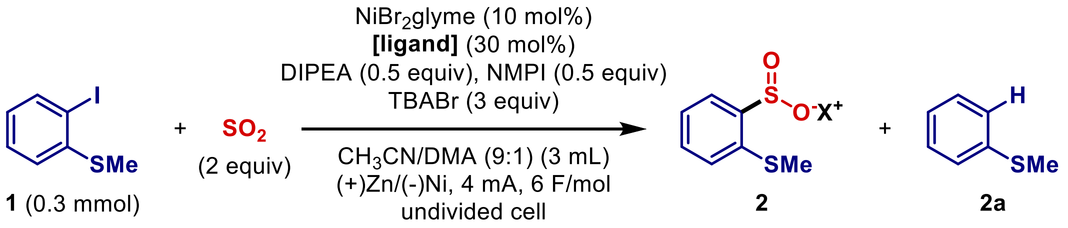

Entry	Ligand	2 (%)	2a (%)	1 (%)
1	**L1**	44	51	0
2	**L2**	36	61	0
3	**L3**	38	59	0
4	**L4**	59	41	0
5	**L5**	28	65	2
6	**L6**	15	68	0
7	**L7**	13	63	9
8	PPh_3	19	73	0
9	Ni(**L4**)$_3$Br$_2$ (10 mol%)	58	42	0
10	Ni(**L4**)$_3$Br$_2$ (5 mol%)	63	36	0

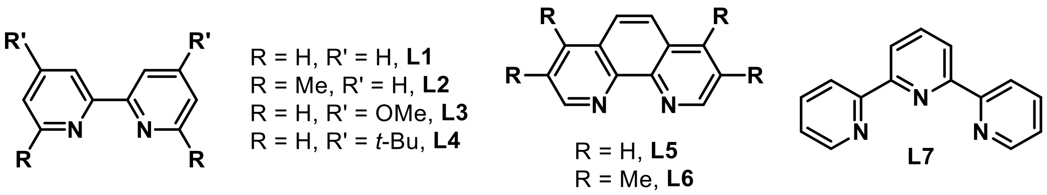

4.6. SO_2 source

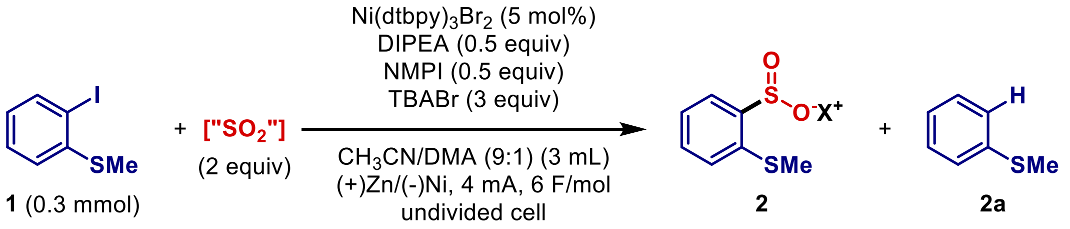

Entry	SO_2 source	**2** (%)	**2a** (%)	**1** (%)
1	DABSO	26	65	0
2	TIMSO	17	70	0
3	DMAP·SO_2	34	56	0
4	$K_2S_2O_5$	1	54	0
5	Rongalite	0	73	0
6	SO_2 in DMA	47	52	0
7	SO_2 in DMA + DIPEA (2 equiv)	57	42	0
8	SO_2 in DMA + DIPEA (1 equiv)	60	40	0
9	SO_2 in DMA + DIPEA (0.5 equiv)	63	36	0

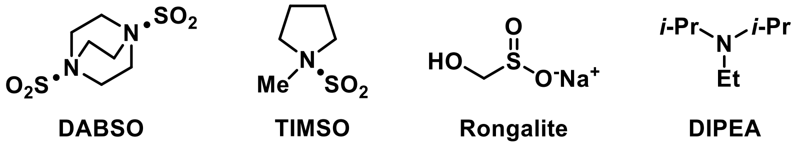

Discussion:

Rongalite and $K_2S_2O_5$ have poor solubility, while DABSO and DMAP·SO_2 are sparingly soluble in the reaction solvent at room temperature.

4.7. Solvent

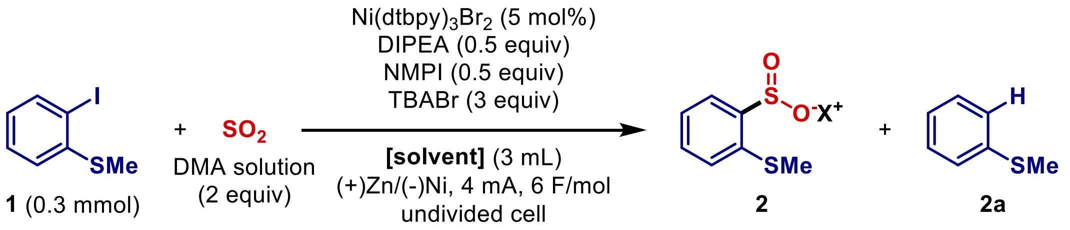

Entry	Solvent	**2** (%)	**2a** (%)	**1** (%)
1	DMA	34	61	0
2	DMF	18	72	0
3	DMSO	8	82	1
4	DCM	14	68	0
5	CH_3CN	63	36	0
6	THF	4	71	1

4.8. Additive

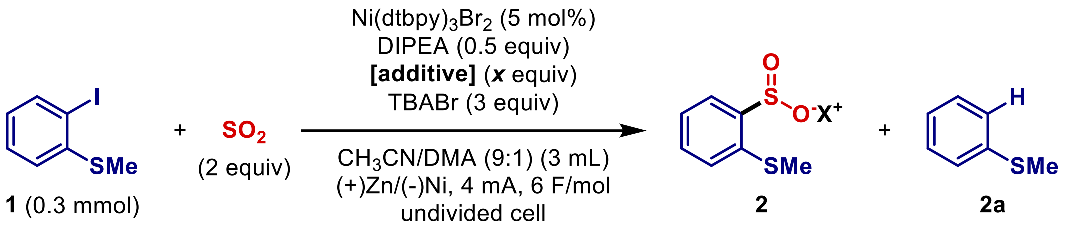

Entry	Additive	(*x* equiv)	**2** (%)	**2a** (%)	**1** (%)
1	**A1**	0.2	47	38	0
2	**A2**	0.2	46	41	0
3	**A3**	0.2	50	43	0
4	**A4**	0.2	46	39	0
5	**A5**	0.2	37	56	0
6	**A6**	0.2	28	59	0
7	**A7**	0.2	37	46	0
8	**A8**	0.2	42	44	0
9	NMPI (**N3**)	0.2	51	48	0
10	**N3**	0.5	63	36	0
11	**N3**	1.0	53	46	0
12	**N1**	0.5	50	49	0
13	**N2**	0.5	52	47	0
14	**N4**	0.5	57	37	0
15	**N5**	0.5	38	51	0
16	TPPA	0.5	42	54	0

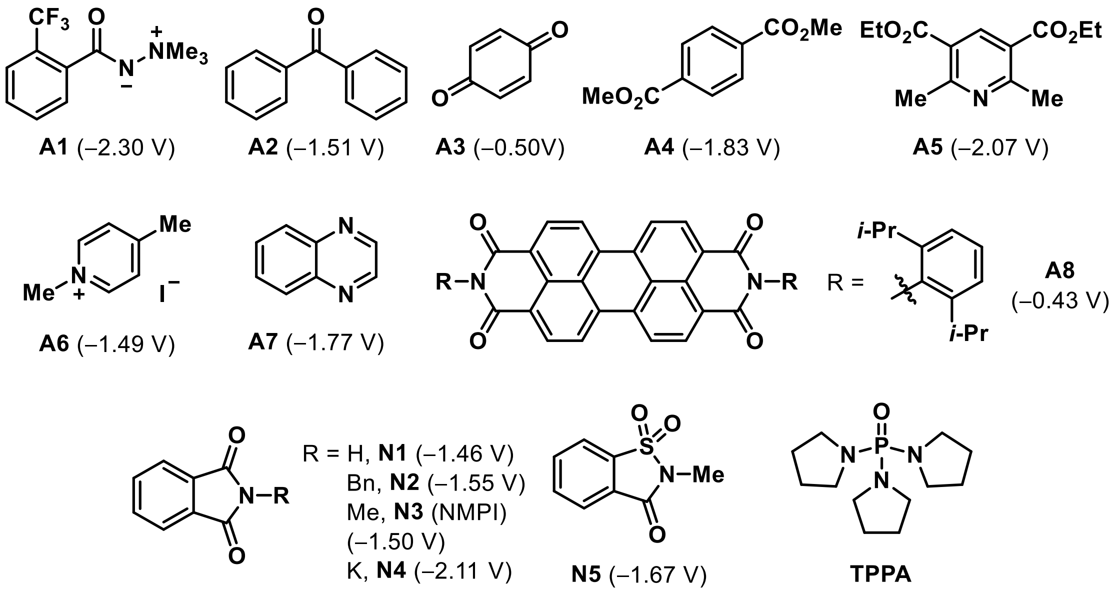

Cathodic peak potentials ($E_{p,c}$) were presented in parentheses and measured with *n*-Bu_4NBr [0.1 M] as electrolyte in CH_3CN/DMA (9:1), glassy carbon working electrode, Ag/AgCl as reference, at 298 K.

4.9. Control experiments

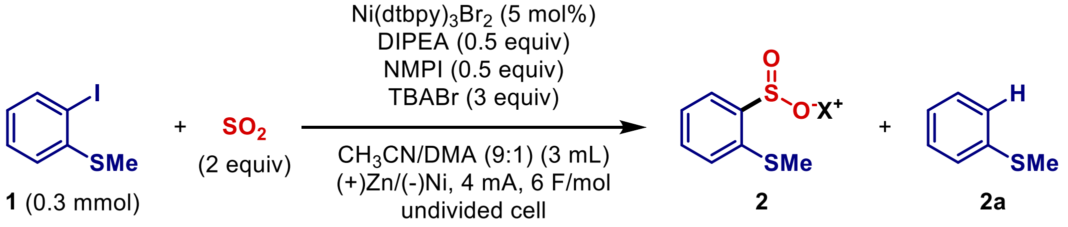

Entry	Conditions	**2** (%)	**2a** (%)	**1** (%)
1	no SO_2	0	66	0
2	no $Ni(dtbpy)_3Br_2$	2	90	0
3	no DIPEA	5	68	7
4	no NMPI	25	65	0
5	no electrolysis	0	0	100
6	Zn dust (3 equiv) added, no electrolysis	0	0	100
7	open to air	3	39	37
8	H_2O (2 equiv) added	28	69	0

4.10. Fluorination

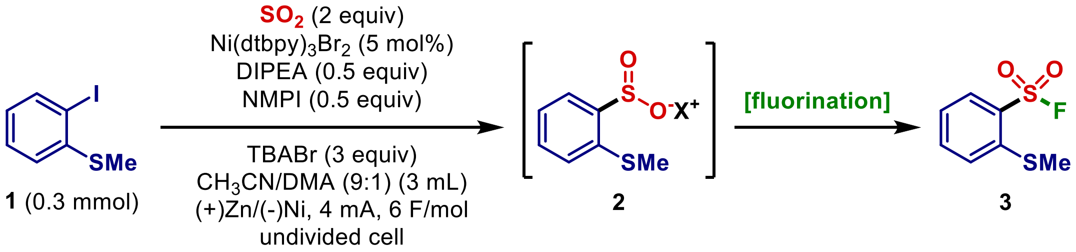

Entry	Conditions	Yield of **3** (%)
1	NFSI (2 equiv)	<5
2	NFSI (4 equiv)	<5
3	Selectfluor (2 equiv)	<5
4	Selectfluor (4 equiv)	<5
5	TBAF (2 equiv), NFSI (2 equiv)	15
6	TBAF (2 equiv), NCS (2 equiv)	31
7	TBAF (4 equiv), NCS (4 equiv)	61 (60)

Yield determined by ^{19}F NMR spectroscopy using $PhCF_3$ as internal standard. Isolated yield presented in parenthesis.

5. Cyclic Voltammograms

Preliminary Study on Reaction Components

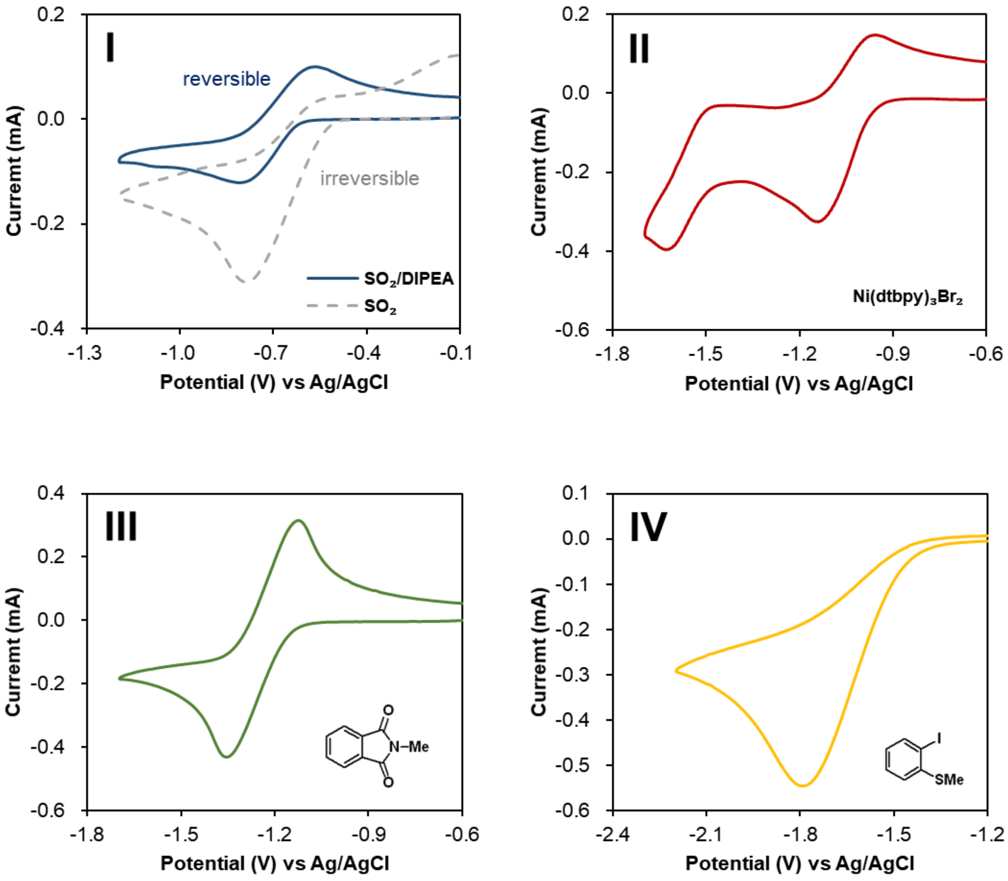

I: SO_2 stock solution in DMA alone [10 mM] (grey dotted line) ($E_{p,c}$ = −0.78 V) and SO_2 solution [10 mM] with DIPEA [10 mM] (blue solid line) ($E_{p,c}$ = −0.81 V; $E_{1/2}$ = −0.69 V). **II**: Ni(dtbpy)$_3$Br$_2$ catalyst [10 mM] (red line) ($E_{p,c,1}$ = −1.14 V; $E_{1/2,1}$ = −1.05 V; $E_{p,c,2}$ = −1.63 V). **III**: *N*-Methylphthalimide [10 mM] (green line) ($E_{p,c}$ = −1.35 V; $E_{1/2}$ = −1.24 V). **IV**: 2-Iodothioanisole (**1**) [10 mM] (yellow line) ($E_{p,c}$ = −1.79 V). Cyclic voltammograms were taken with *n*-Bu_4NBF_4 [0.1 M] as electrolyte in degassed CH_3CN, glassy carbon working electrode, Ag/AgCl as reference, at 298 K. Scan rate = 200 mV s^{-1}. $E_{p,c}$ and $E_{1/2}$ represent cathodic peak potential and half wave potential, respectively.

Cyclic Voltammograms Recorded at Optimized Conditions

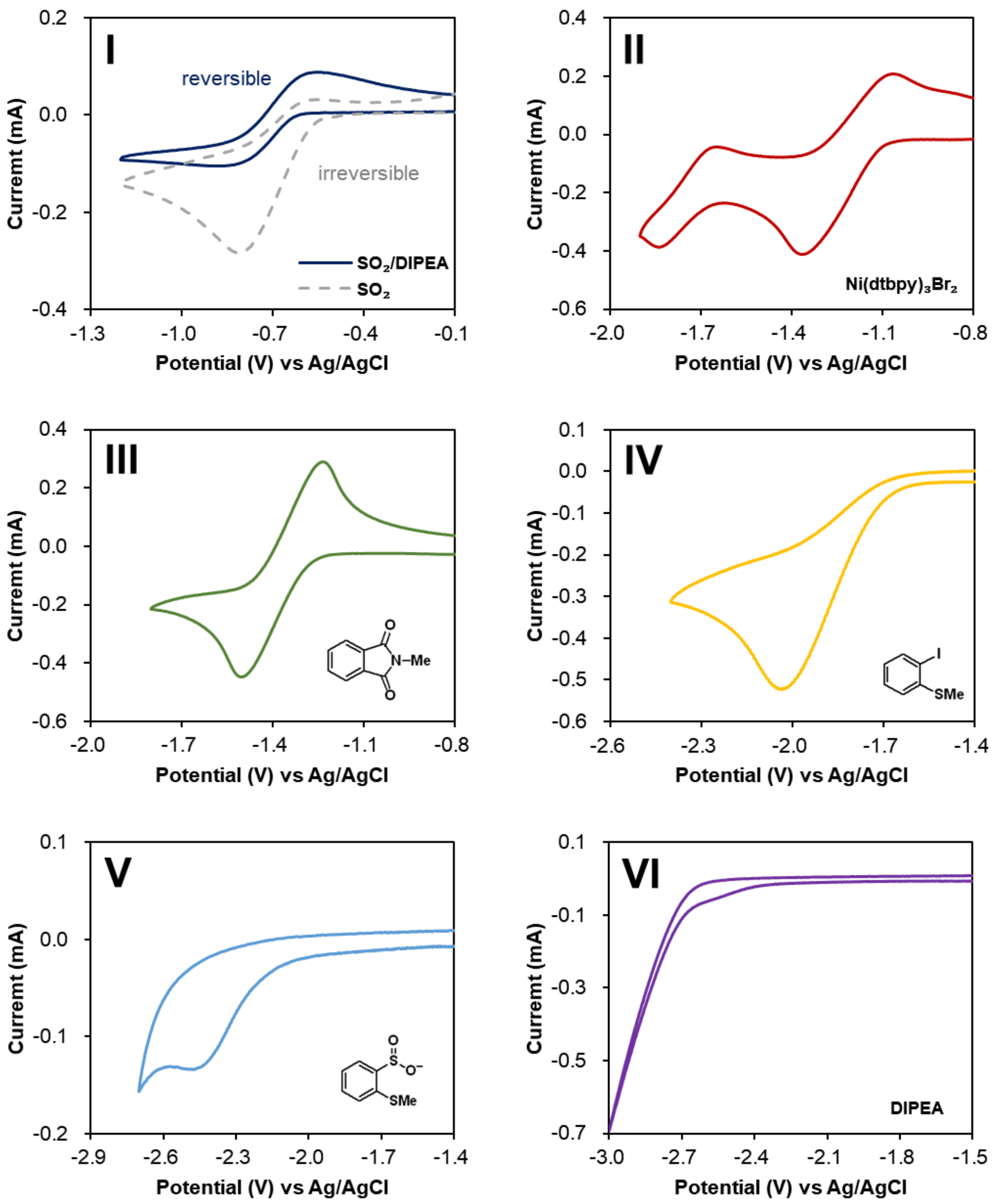

I: SO_2 stock solution in DMA alone [10 mM] (grey dotted line) ($E_{p,c}$ = −0.81 V) and SO_2 solution [10 mM] with DIPEA [10 mM] (dark blue solid line) ($E_{p,c}$ = −0.89 V; $E_{1/2}$ = −0.68 V). **II**: Ni(dtbpy)$_3$Br$_2$ catalyst [10 mM] (red line) ($E_{p,c,1}$ = −1.36 V; $E_{1/2,1}$ = −1.21 V; $E_{p,c,2}$ = −1.83 V; $E_{1/2,2}$ = −1.73 V). **III**: *N*-Methylphthalimide [10 mM] (green line) ($E_{p,c}$ = −1.50 V; $E_{1/2}$ = −1.36 V). **IV**: 2-Iodothioanisole (**1**) [10 mM] (yellow line) ($E_{p,c}$ = −2.04 V). **V**: 2-(Methylthio)benzenesulfinate (**2**) [10 mM] (blue line) ($E_{p,c}$ = −2.48 V). **VI**: DIPEA [10 mM] (violet line). Cyclic voltammograms were taken with *n*-Bu$_4$NBr [0.1 M] as electrolyte in degassed CH_3CN/DMA (9:1), glassy carbon working electrode, Ag/AgCl as reference, at 298 K. Scan rate = 200 mV s^{-1}. $E_{p,c}$ and $E_{1/2}$ represent cathodic peak potential and half wave potential, respectively.

Cyclic Voltammograms of Additives for Table 4.8

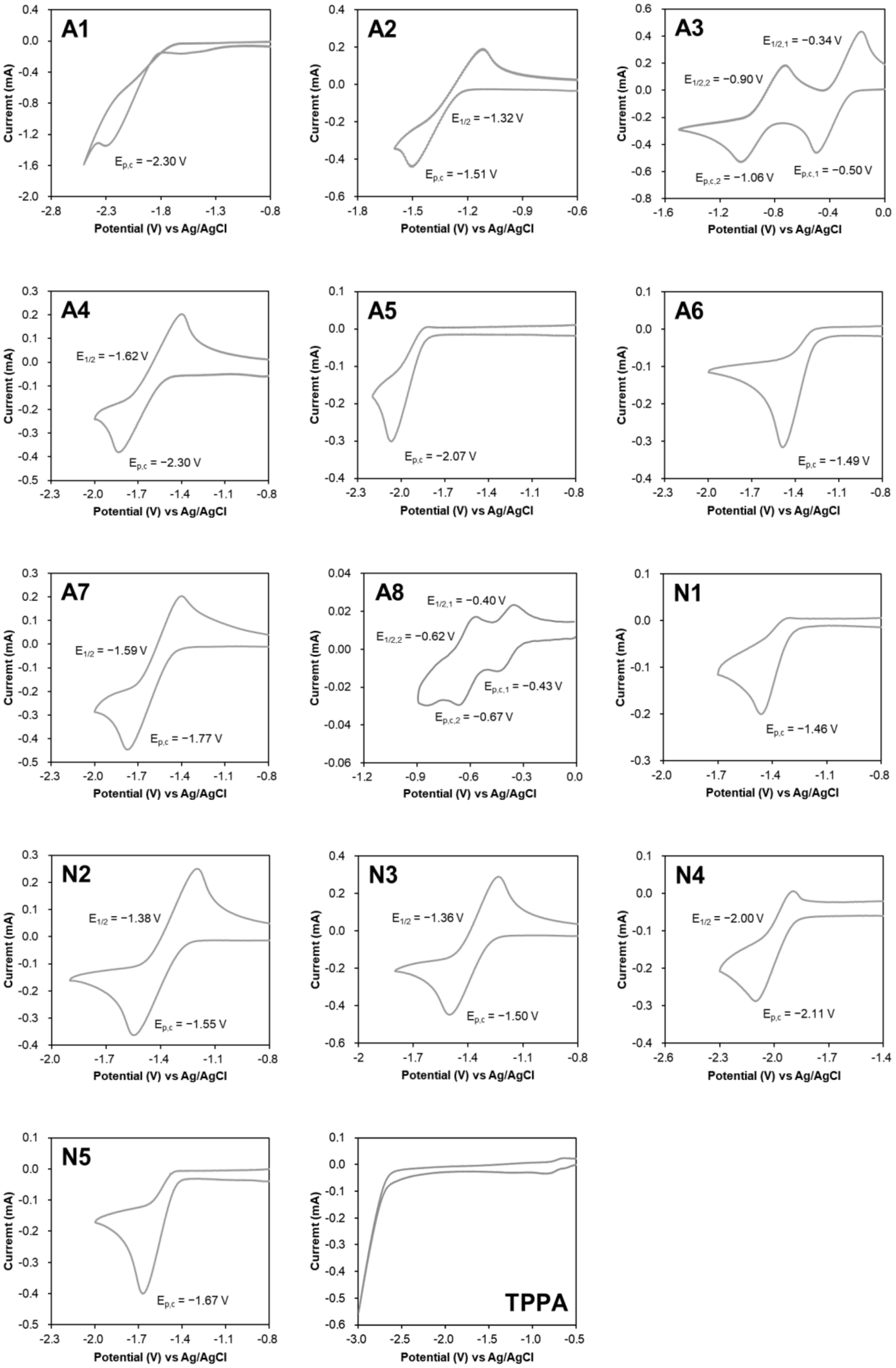

See next page for cyclic voltammetry conditions.

Cyclic Voltammograms of Selected Aryl Iodide

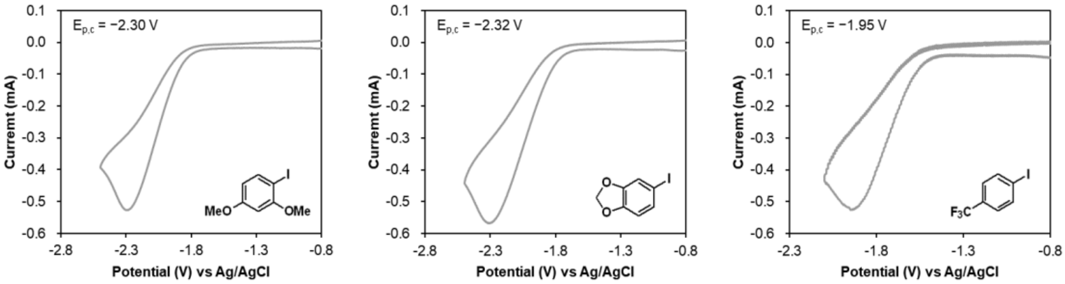

Cyclic voltammograms were taken with *n*-Bu_4NBr [0.1 M] as electrolyte in degassed CH_3CN/DMA (9:1), glassy carbon working electrode, Ag/AgCl as reference, at 298 K. Scan rate = 200 mV s^{-1}. $E_{p,c}$ and $E_{1/2}$ represent cathodic peak potential and half wave potential (if reversible), respectively.

Discussion

The corresponding yields of sulfonyl fluoride for the above substrates are 44% (**7**), 62% (**9**) and 64% (**17**), respectively. Despite the similar reduction potential of the aryl iodides for **7** and **9**, their yields are notably different; meanwhile the reduction potential of 5-iodo-1,3-benzodioxole and 4-iodobenzotrifluoride are distinctly different, and both were effectively converted into sulfonyl fluorides in similar yields. This shows that there is no clear correlation between substrate reduction potentials and reaction yields, and further suggesting that the reaction is mediated by nickel catalyst rather than direct reductive activation of substrate.

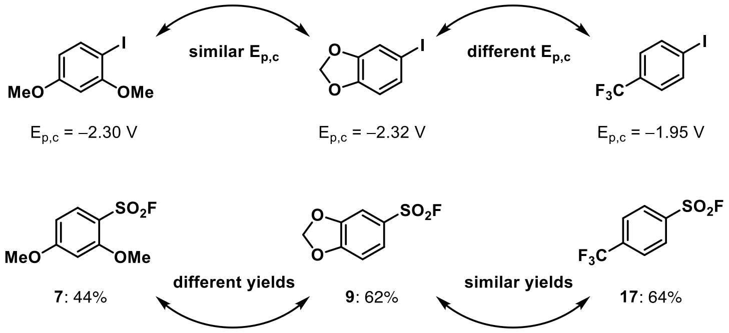

6. Cathodic Potential-Time (V-t) Graph

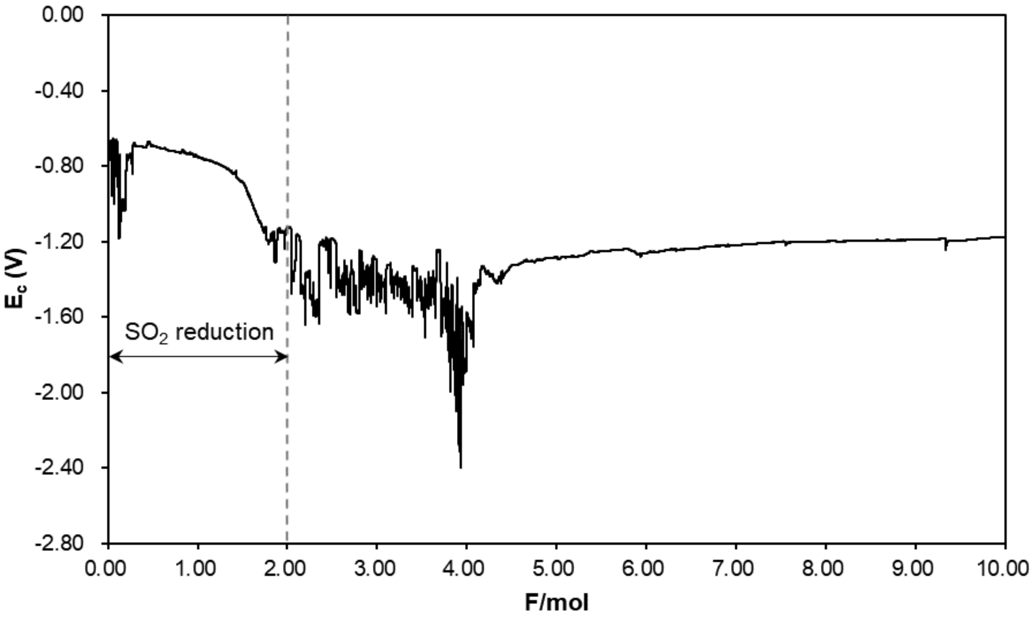

Cathodic potential (E_c)-time graph for the e-sulfinylation of 2-iodothioanisole (**1**) was recorded on EZstat PRO potentiostat/galvanostat under optimized conditions and a constant current of 4.0 mA, using Ag/AgCl as reference, at an interval of 1 second.

7. Reaction Profile

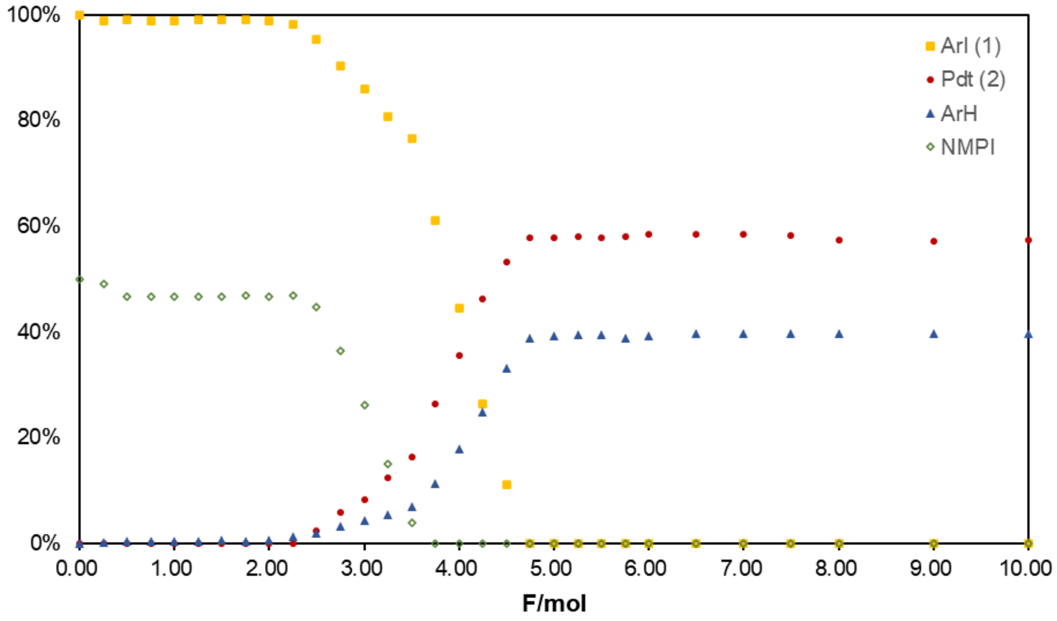

Reaction profile for the e-sulfinylation of 2-iodothioanisole (**1**). Yellow squares represent aryl iodide starting material (**1**), red dots represent thioanisole-2-sulfinate product (**2**), blue triangles represent proto-dehalogenated side product (thioanisole), and green hollow diamonds represent *N*-methylphthalimide (NMPI). Reaction conditions: 2-iodothioanisole (**1**) (0.3 mmol, 1 equiv), SO_2 in DMA [2.03 M] (2 equiv), $Ni(dtbpy)_3Br_2$ (5 mol%), DIPEA (0.5 equiv), NMPI (0.5 equiv), TBABr (3 equiv), CH_3CN (2.7 mL), undivided cell, (+)Zn/(-)Ni foam, 4 mA, 10 F/mol, rt, N_2. Reaction was monitored by HPLC using 4,4'-di-*tert*-butylbiphenyl (0.2 equiv) as internal standard, at an interval of 0.25 F/mol (from 0.0 to 6.0 F/mol), 0.5 F/mol (from 6.0 to 8.0 F/mol), and 1.0 F/mol (from 8.0 to 10.0 F/mol).

8. General Procedures and Graphical Guide

General Procedure A: Electrochemical Ni-Catalyzed Synthesis of Aryl Sulfonyl Fluorides from Aryl Iodides

A 5-mL ElectraSyn vial with a magnetic stir bar was charged with aryl iodide (0.30 mmol, 1.0 equiv), NMPI (24.2 mg, 0.15 mmol, 0.50 equiv), $Ni(dtbpy)_3Br_2$ (15.4 mg, 0.015 mmol, 5.0 mol%), TBABr (290 mg, 0.90 mmol, 3.0 equiv) and DIPEA (26 μL, 0.15 mmol, 0.50 equiv), and was capped with an ElectraSyn vial cap equipped with zinc (anode) and nickel foam (cathode), sealed with a rubber septum, and evacuated and back-filled with N_2 for three times. Anhydrous CH_3CN (2.7 mL), and SO_2 stock solution (in DMA, concentration determined by titration) (ca. 0.3 mL, 0.60 mmol, 2.0 equiv) were added subsequently. The reaction mixture was stirred until a homogeneous clear solution was formed, and electrolyzed under a constant current of 4.0 mA for 6.0 F/mol (ca. 12 h) and a positive pressure of N_2. After the electrolysis, the ElectraSyn vial cap was removed and electrodes were rinsed with CH_3CN (ca. 2 mL). TBAF (1 M in THF) (1.2 mL, 1.2 mmol, 4.0 equiv) and NCS (160 mg, 1.2 mmol, 4.0 equiv) were then added with effective stirring, and the reaction mixture was stirred at room temperature for 1 h. [Crude yield can be determined by adding $PhCF_3$ (12.3 μL, 0.10 mmol) (δ_F -63 ppm) or 1,4-difluorobenzene (15.4 μL, 0.15 mmol) (δ_F -120 ppm) as internal standard, and an aliquot of crude mixture was taken and diluted with $CDCl_3$ for ^{19}F NMR spectroscopy. Note that the chemical shifts would be slightly different due to the mixing of solvent.] The crude mixture was diluted with Et_2O and washed with brine. The organic layer was dried over anhydrous $MgSO_4$, filtered and concentrated *in vacuo*. The crude material was purified by flash column chromatography or preparative thin-layer chromatography (PTLC) to afford the desired product.

General Procedure B: Electrochemical Ni-Catalyzed Synthesis of Aryl Sulfonyl Fluorides from Aryl Bromides

Following General Procedure A, with aryl bromide (0.30 mmol, 1.0 equiv) was used in lieu of aryl iodide. After electrolysis, NFSI (142 mg, 0.45 mmol, 1.5 equiv) was added instead of TBAF and NCS.

Graphical Guide for General Procedure A (0.3-mmol Scale)

As exemplified by the preparation of 2-(methylthio)benzenesulfonyl fluoride (**3**):

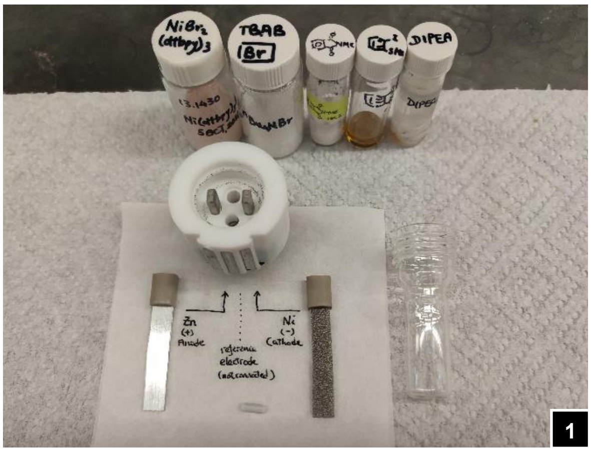

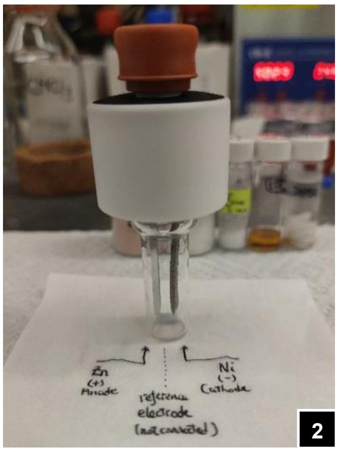

Left: Materials and reagents for e-sulfonylation. **Right**: 5-mL ElectraSyn vial equipped with cap, electrodes and a magnetic stir bar.

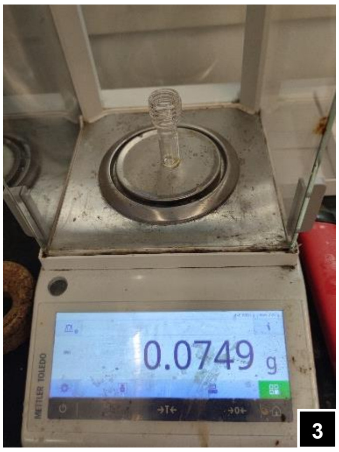

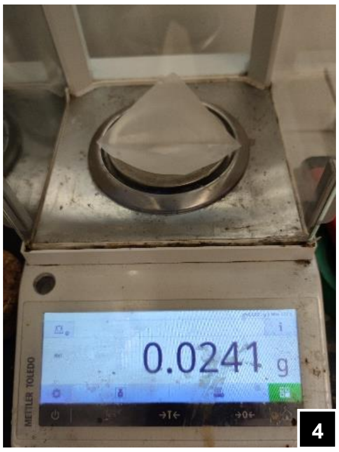

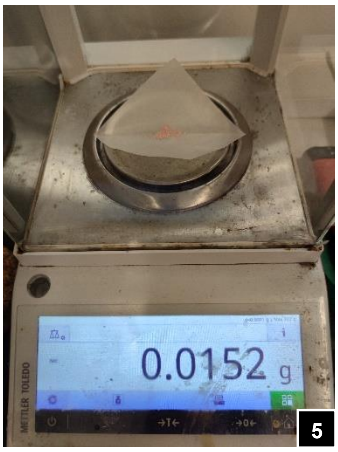

Left: 2-Iodothioanisole (**1**) (75 mg, 0.30 mmol). **Center:** *N*-Methylphthalimide (NMPI) (24 mg, 0.15 mmol). **Right:** $Ni(dtbpy)_3Br_2$ (15.4 mg, 0.015 mmol).

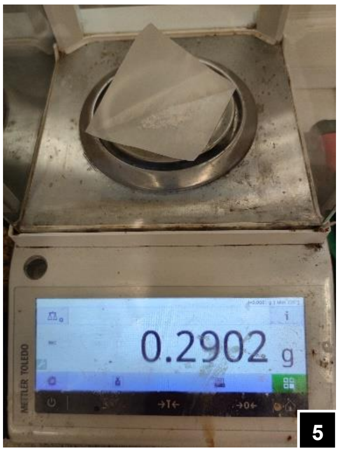

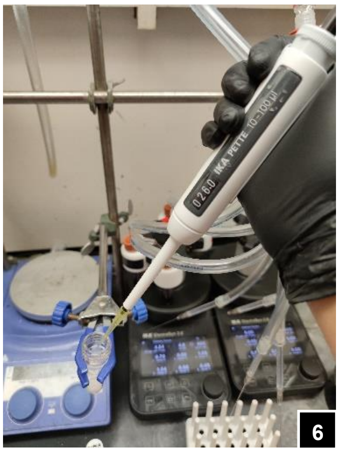

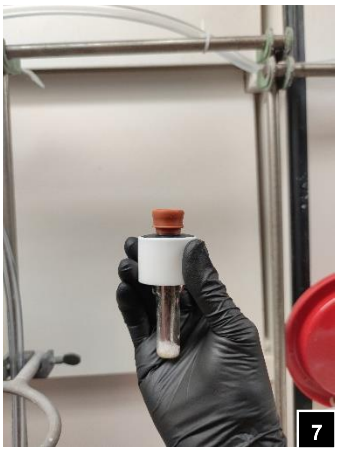

Left: TBABr (290 mg, 0.90 mmol). **Center:** DIPEA (26 μL, 0.15 mmol). **Right:** The vial was capped and sealed with a rubber septum.

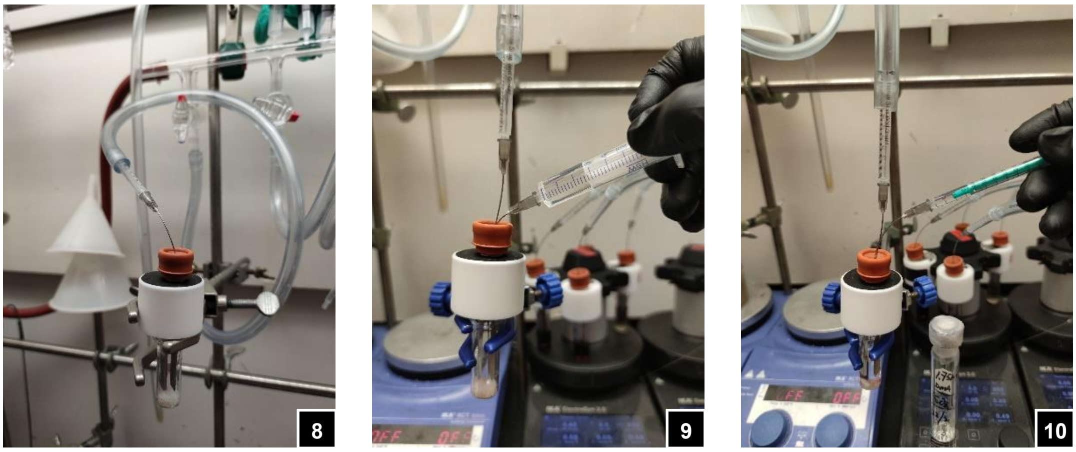

Left: Sealed vial was evacuated and back-filled with N_2 for three times. **Center:** Anhydrous CH_3CN (2.7 mL). **Right:** SO_2 solution (1.75 M in DMA) (0.34 mL, 0.60 mmol).

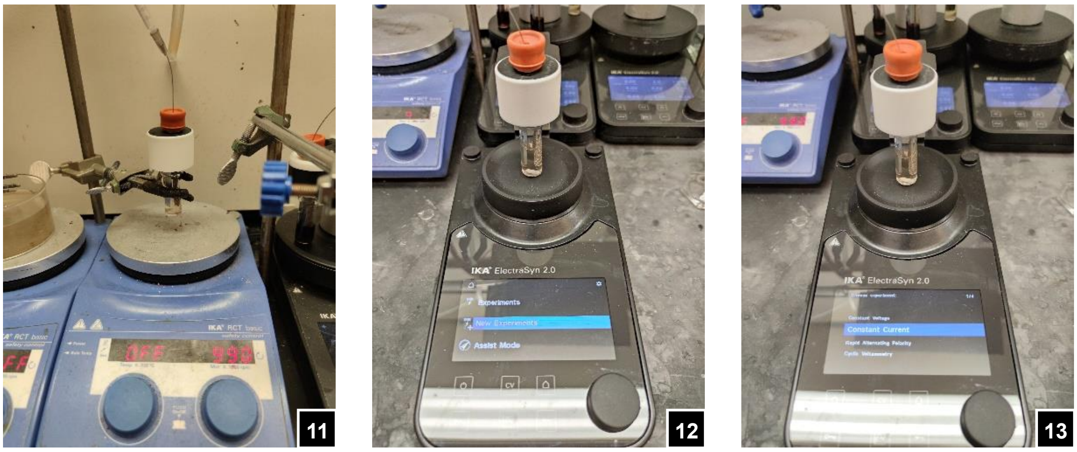

Left: The reaction mixture was stirred until a clear solution was formed. **Center:** Connect vial to an ElectraSyn 2.0, select "*New Experiments*". **Right:** Select "*Constant Current*".

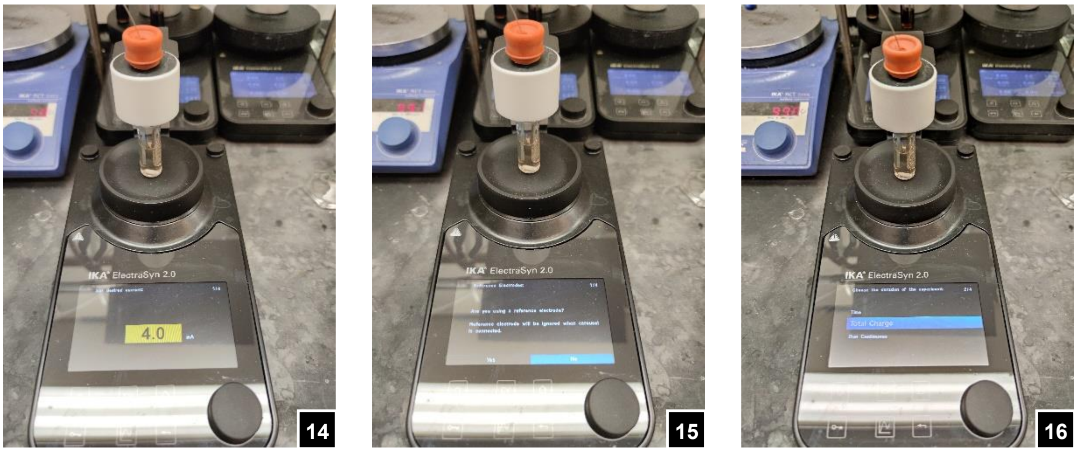

Left: Set current to 4.0 mA. **Center:** Select "*No*" for reference electrode. **Right:** Select "*Total Charge*".

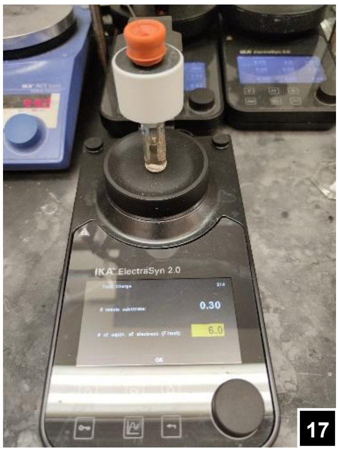

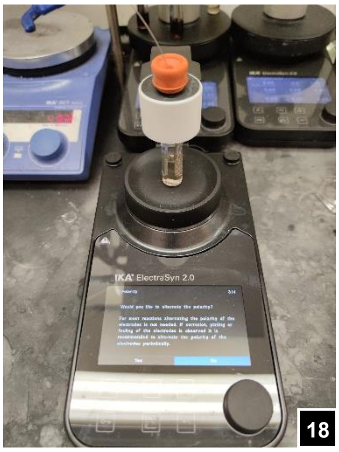

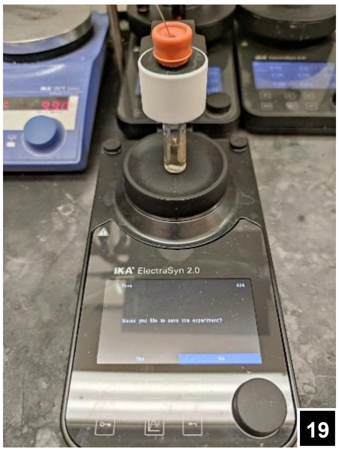

Left: Set 0.30 mmol of substrate and 6.0 F/mol. **Center:** Select "*No*" for alternating polarity. **Right:** Save the experimental parameters if desired.

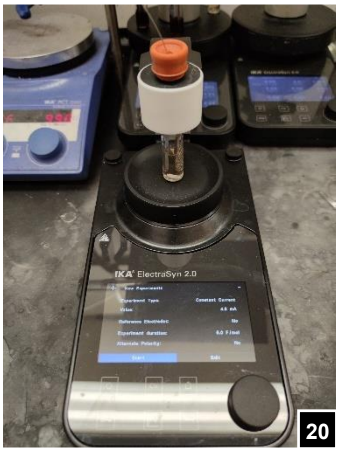

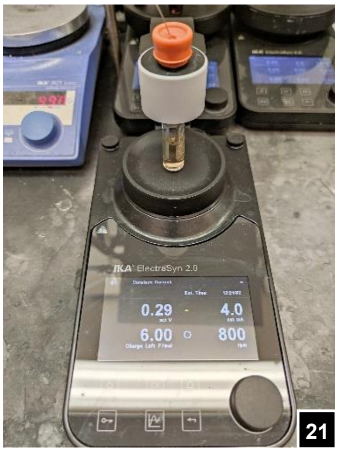

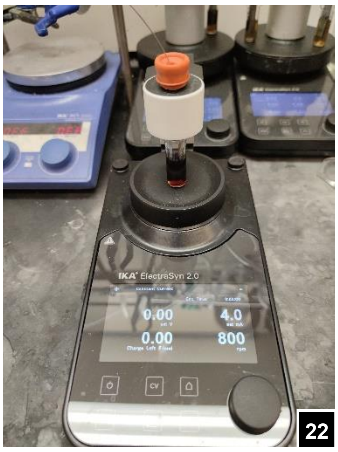

Left: Review experimental parameters and press "*Start*" when ready. **Center:** Reaction started with stirred at 800 rpm; reaction time estimated to be around 12 h, and voltage normally remained below 1 V (check reaction setup if voltage went beyond 1.5 V). **Right:** E-sulfonylation completed.

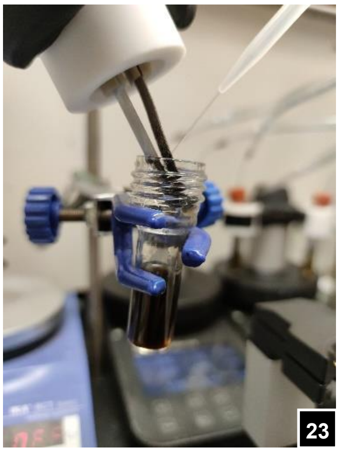

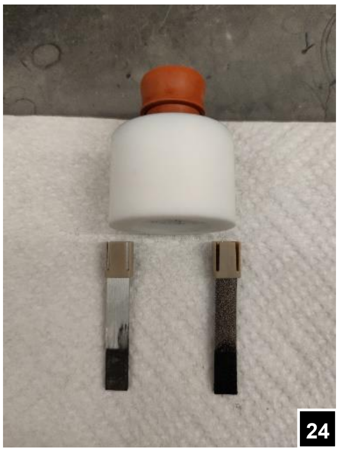

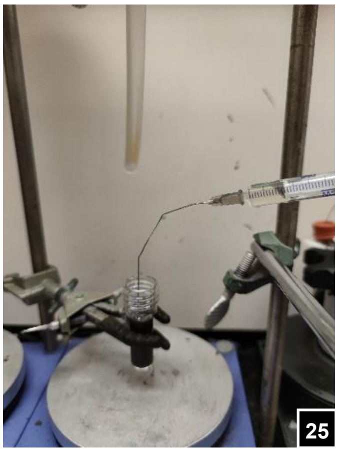

Left: Electrodes were rinsed with CH_3CN. **Center:** Electrodes covered in black deposit (ZnS) after reaction. **Right:** TBAF (1 M in THF) (1.2 mL, 1.2 mmol).

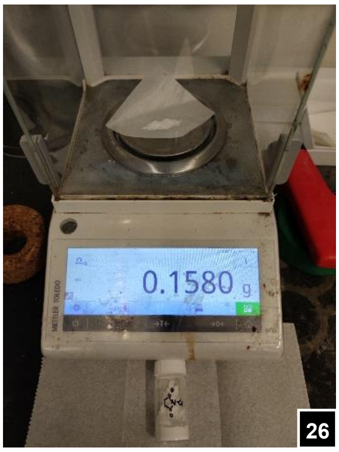

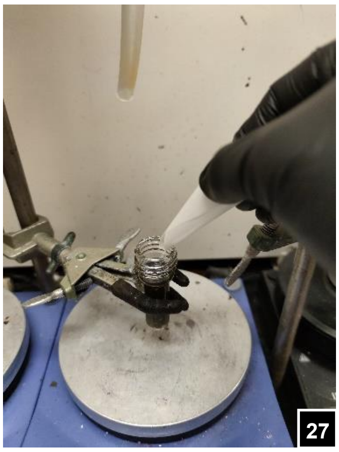

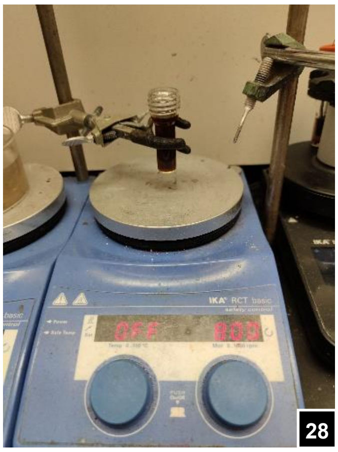

Left: NCS (160 mg, 1.2 mmol). **Center:** NCS was added directly into the sulfonylation mixture. **Right:** Reaction was stirred for 1 h.

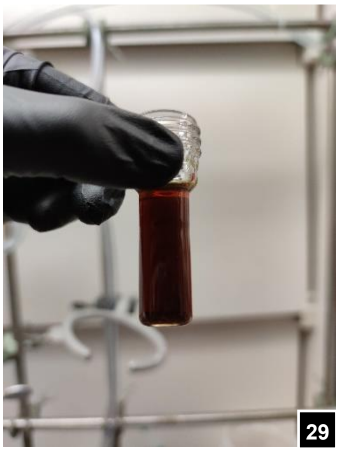

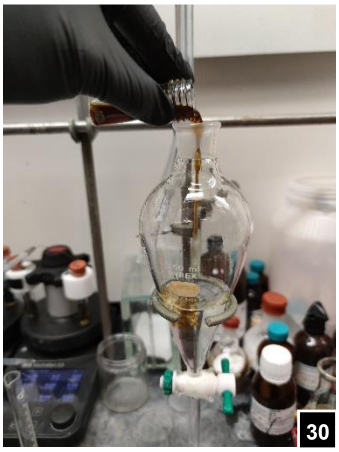

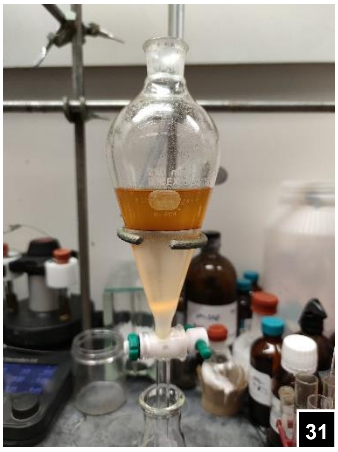

Left: Fluorination completed. **Center:** Reaction mixture was poured into a separating funnel containing brine, and diluted with Et_2O. **Right:** The biphasic mixture was shaken rigorously.

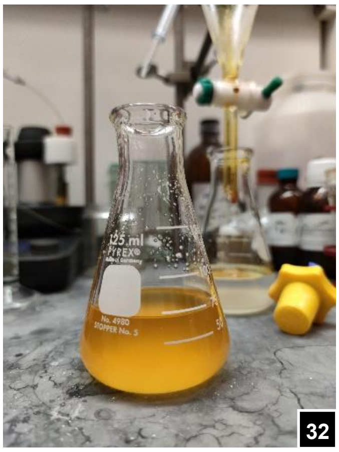

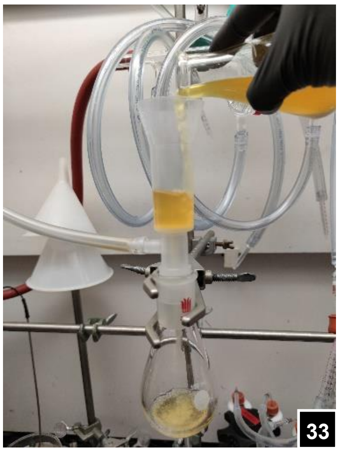

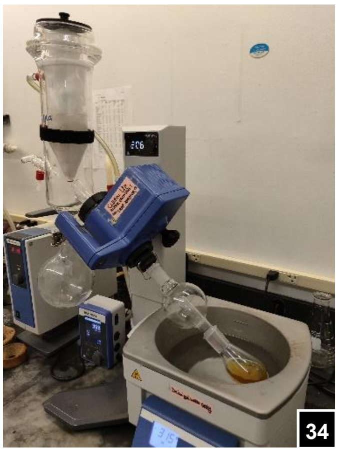

Left: Ethereal extract was dried over anhydrous $MgSO_4$. **Center:** $MgSO_4$ was filtered off under suction. **Right:** Crude mixture was concentrated *in vacuo*.

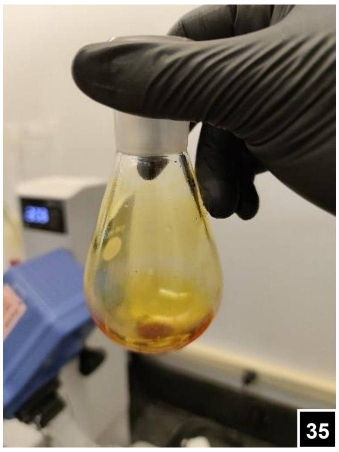

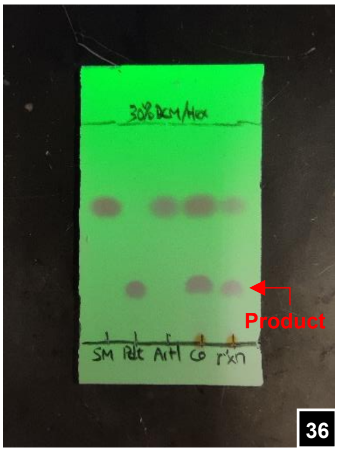

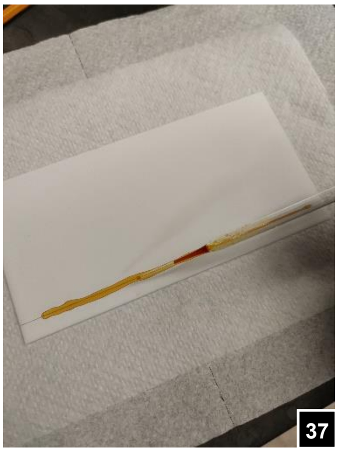

Left: Concentrated crude mixture. **Center:** Crude TLC under UV (30% CH_2Cl_2 in hexane) – [from left to right] aryl iodide, sulfonyl fluoride product, thioanisole, co-spot, and reaction crude. **Right:** Purified by preparative TLC.

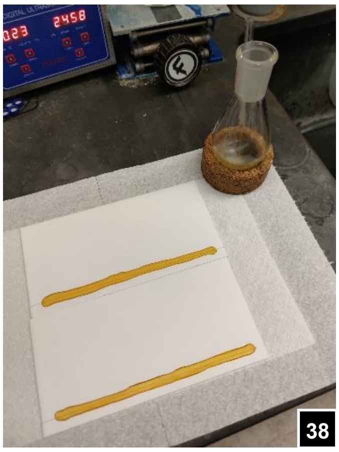

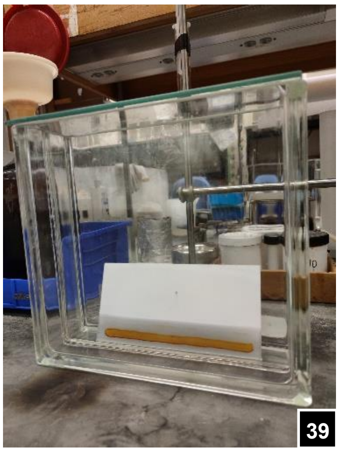

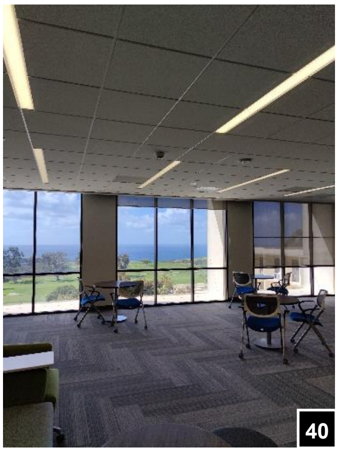

Left: Use two plates if necessary. **Center:** Preparative TLC was developed using 30% CH_2Cl_2 in hexane. **Right:** Take a break in the library while waiting...

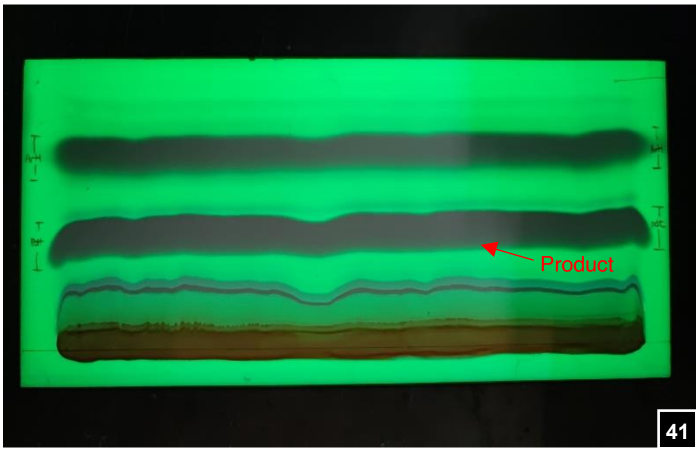

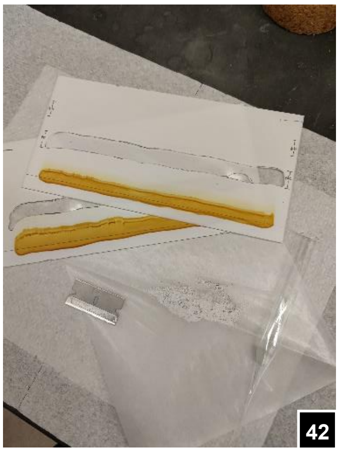

Left: Developed preparative TLC under UV. **Right:** Product band was scratched off.

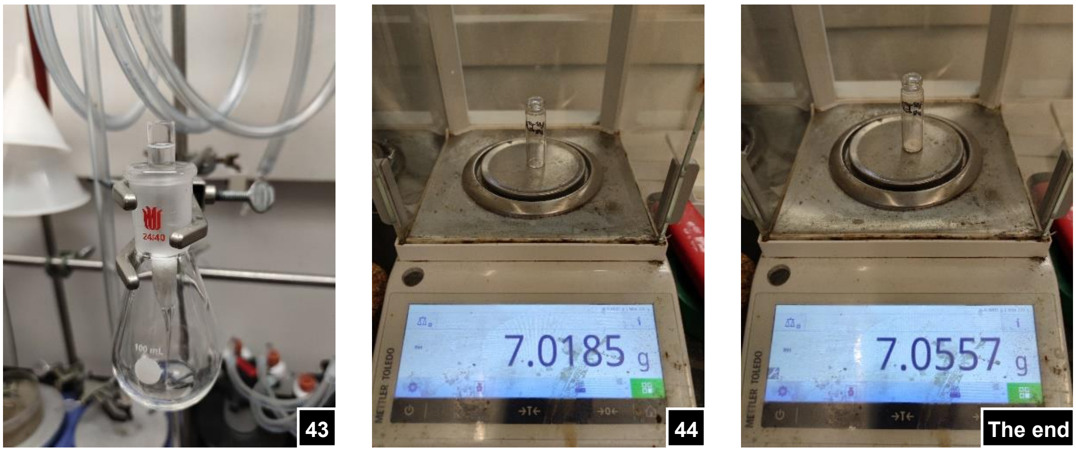

Left: Silica gel containing product was filtered over cotton wool and extracted with EtOAc. **Center:** Tare weight of an empty vial. **Right:** Weight of vial containing product **3** (37.2 mg, 60%).

9. Procedure for Gram-Scale Reaction

As exemplified by the preparation of 4-bromo-benzenesulfonyl fluoride (**15**):

A 20-mL ElectraSyn vial with a stir bar was charged with 1-bromo-4-iodobenzene **42** (1.00 g, 3.53 mmol, 1.0 equiv), NMPI (283.7 mg, 1.76 mmol, 0.50 equiv), $Ni(dtbpy)_3Br_2$ (72.3 mg, 0.071 mmol, 2.0 mol%) and TBABr (3.40 g, 10.6 mmol, 3.0 equiv), and was capped with an ElectraSyn vial cap equipped with zinc (anode) and nickel foam (cathode) [ca. 5 cm^2 each, see graphical guide below for further details], sealed with a rubber septum, and evacuated and back-filled with N_2 for three times. Anhydrous CH_3CN (11 mL) was added until ca. 3 cm of each electrode was immersed in the solution, followed by the addition of DIPEA (0.31 mL, 1.78 mmol, 0.50 equiv). The mixture was stirred for 5 min until all solid dissolved, and SO_2 stock solution (6.5 M in DMA) (1.1 mL, 7.15 mmol, 2.0 equiv) was then added. The reaction mixture was electrolyzed under a constant current of 25 mA for 6.0 F/mol (ca. 24 h) and a positive pressure of N_2. After the electrolysis, the ElectraSyn vial cap was removed and electrodes were rinsed with CH_3CN (ca. 2 mL). TBAF (1 M in THF) (14 mL, 14 mmol, 4.0 equiv) and NCS (1.89 g, 14.2 mmol, 4.0 equiv) were then added slowly with effective stirring, and the reaction mixture was stirred at room temperature for 1 h. The crude mixture was diluted with brine and extracted with Et_2O for three times. The combined organic extract was dried over anhydrous $MgSO_4$, filtered and concentrated *in vacuo*. The crude material was purified by flash column chromatography (0–30% CH_2Cl_2 in hexane) to afford 4-bromo-benzenesulfonyl fluoride **15** as a white solid (444 mg, 53%).

Graphical Guide (Gram-scale Reaction)

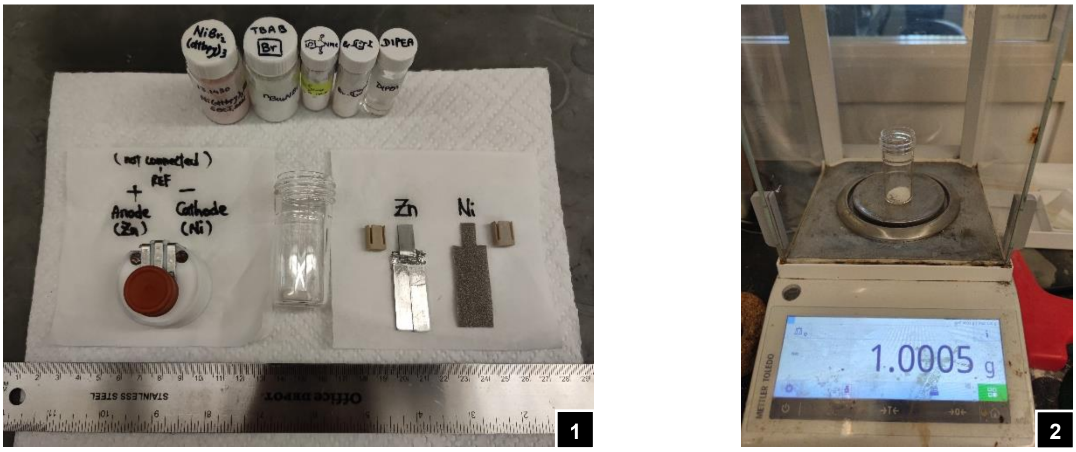

Left: Materials and reagents for e-sulfonylation; Zn electrode was constructed by tying up three pieces of used Ika's ElectraSyn Zn electrodes, and Ni foam was cut into the same shape as the Zn electrode. **Right:** 1.00 g of 1-bromo-4-iodobenzene (**42**) (3.53 mmol).

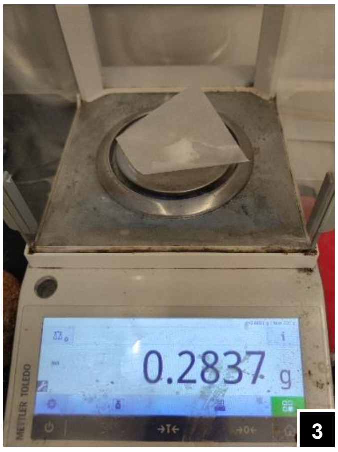

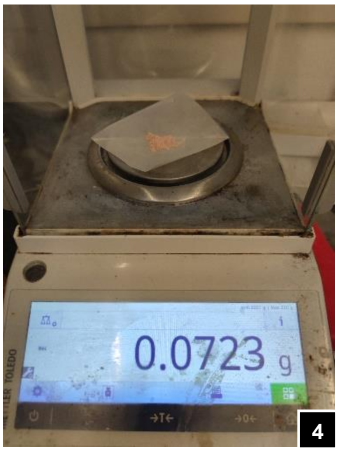

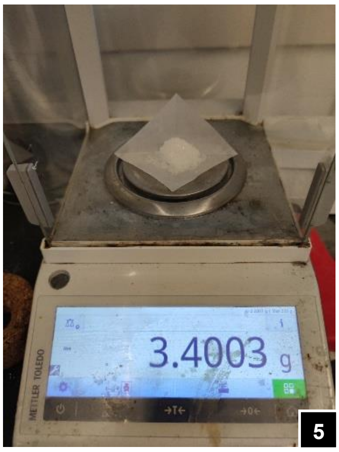

Left: *N*-methylphthalimide (NMPI) (284 mg, 1.76 mmol). **Center:** Ni(dtbpy)$_3$Br$_2$ (72.3 mg, 0.071 mmol). **Right:** TBABr (3.40 g, 10.6 mmol).

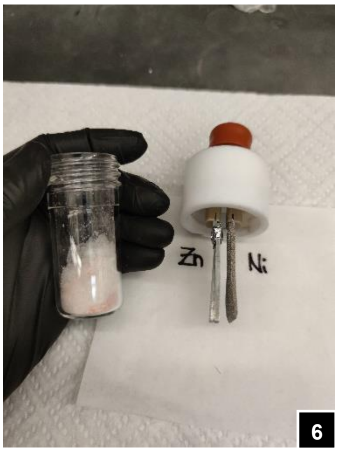

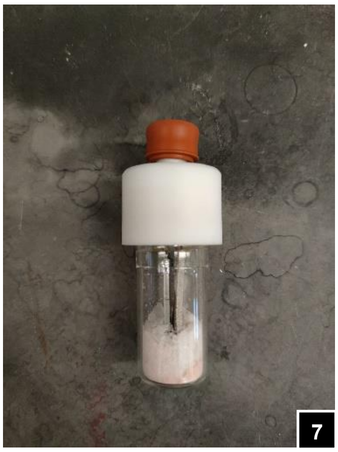

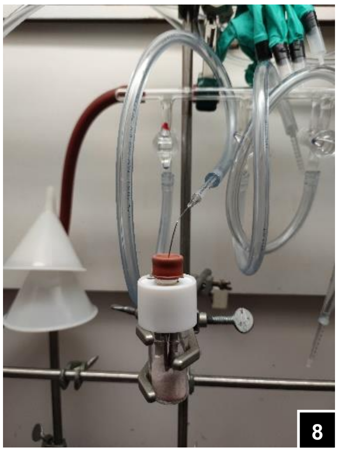

Left: All solid reagents in a 20-mL ElectraSyn vial and vial cap equipped with Zn anode and Ni foam cathode. **Center:** The vial was capped and sealed with a rubber septum. **Right:** The sealed vial was evacuated and back-filled with N$_2$ for three times.

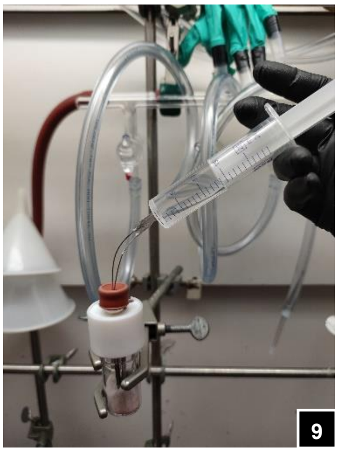

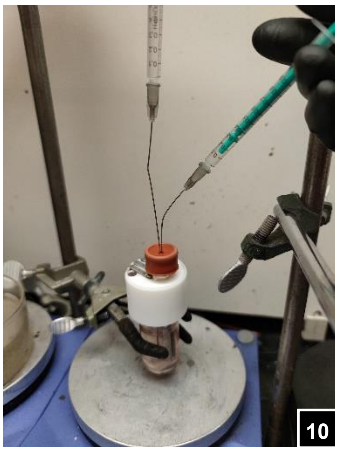

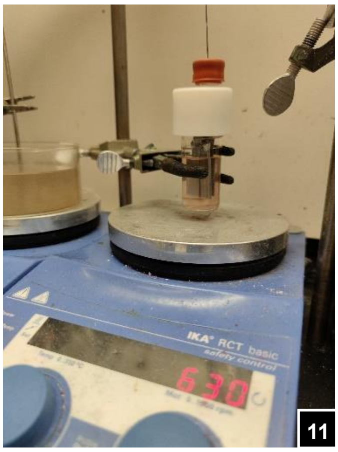

Left: Anhydrous CH$_3$CN (ca. 11 mL) was added until ca. 3 cm of each electrode was immersed in the solution. **Center:** DIPEA (0.31 mL, 1.78 mmol). **Right:** The mixture was stirred for 5 min until all solid dissolved to give a clear pink solution.

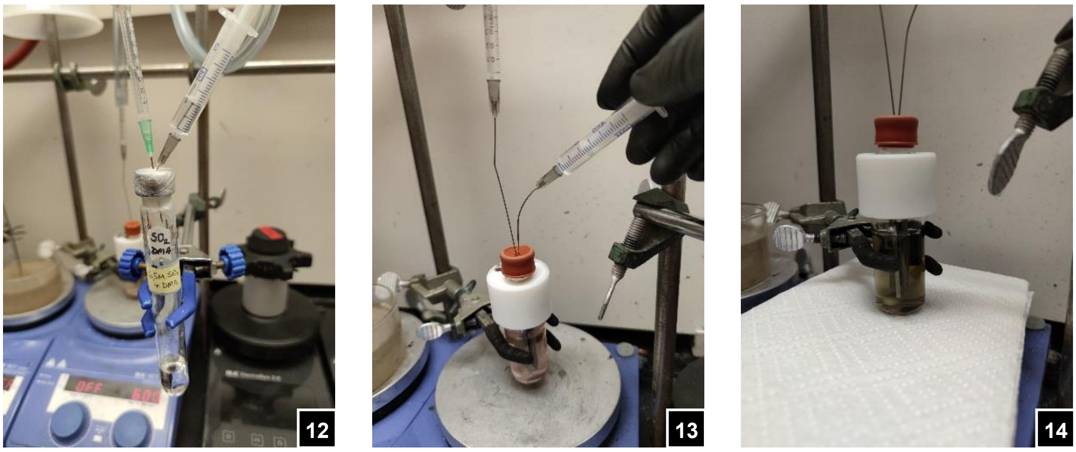

Left: SO_2 stock solution (6.5 M in DMA) (1.1 mL, 7.15 mmol). **Center:** SO_2 stock solution was added slowly. **Right:** Solution turned dark yellow upon the addition of SO_2.

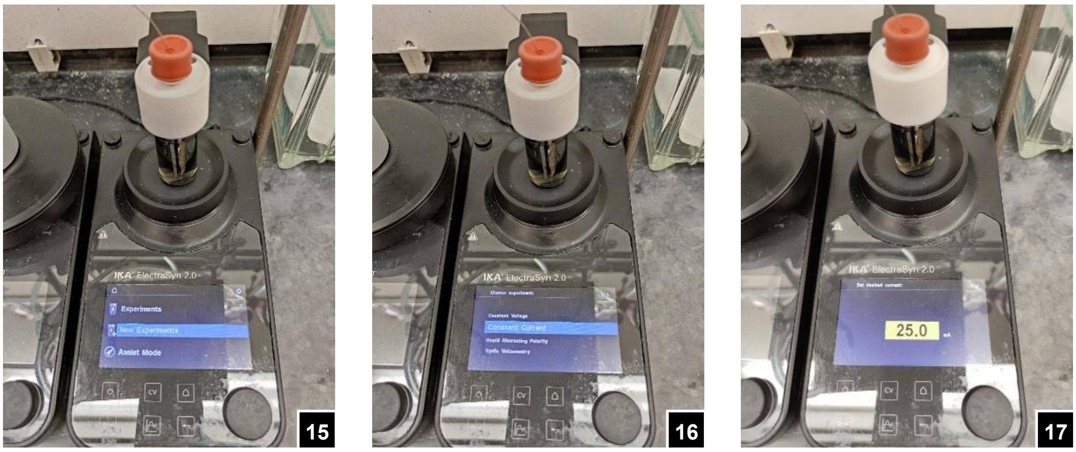

Left: Connect vial to an ElectraSyn 2.0, select "*New Experiments*". **Center:** Select "*Constant Current*". **Right:** Set current to 25.0 mA.

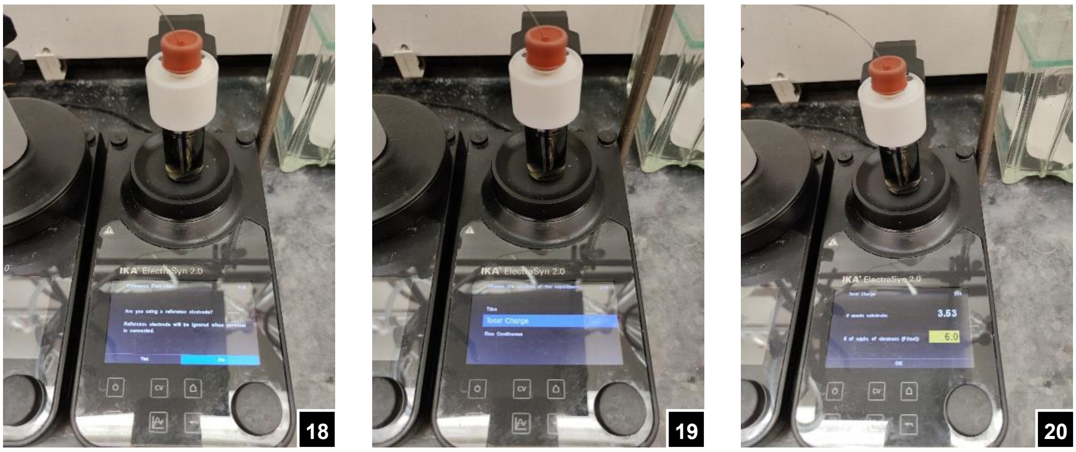

Left: Select "*No*" for reference electrode. **Center:** Select "*Total Charge*". **Right:** Set 3.53 mmol of substrate and 6.0 F/mol.

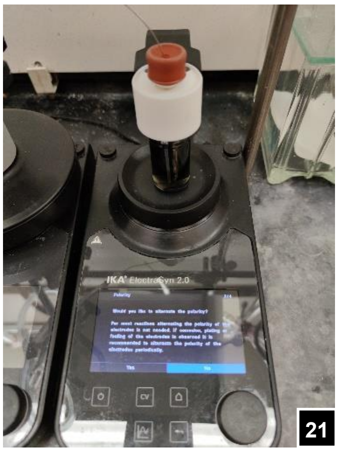

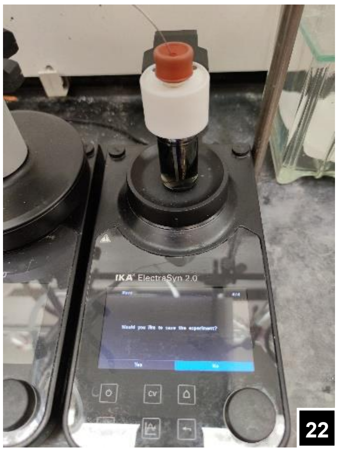

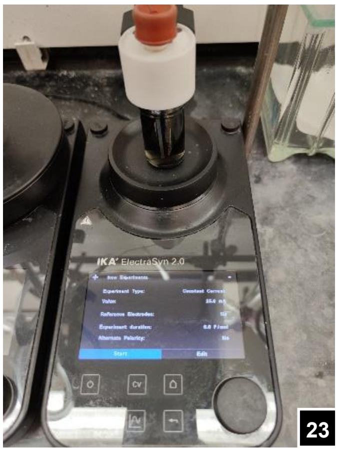

Left: Select "*No*" for alternating polarity. **Center:** Save the experimental parameters if desired. **Right:** Review experimental parameters and press "*Start*" when ready.

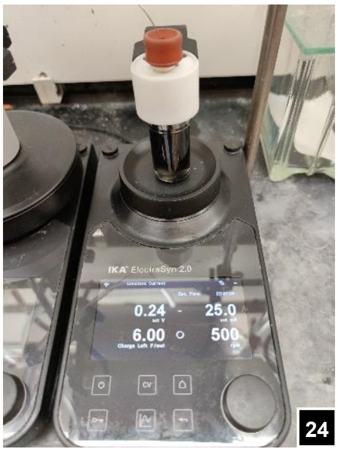

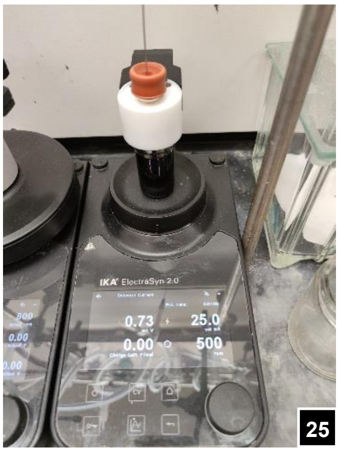

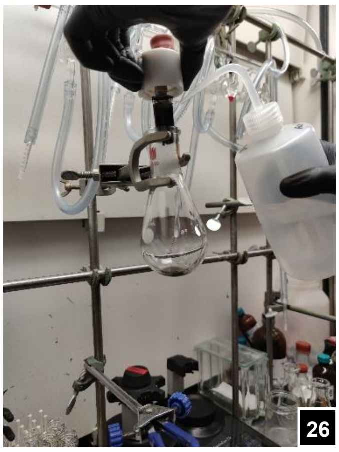

Left: Reaction started with stirred at 500 rpm; reaction time estimated to be around 23 h, and voltage remained below 1 V throughout the course. **Center:** E-sulfonylation completing in 1 min. **Right:** Upon completion, the vial cap was taken off and electrodes were rinsed with CH_3CN into a round-bottom flask.

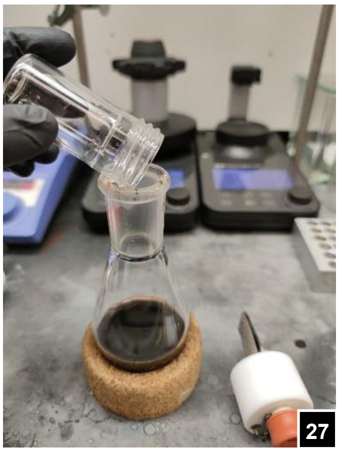

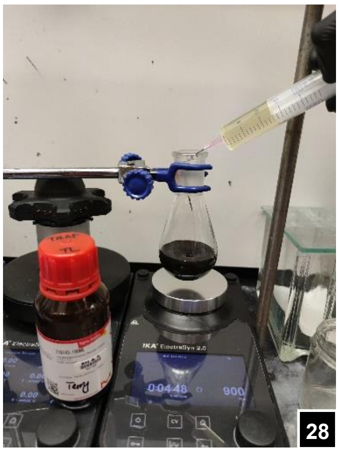

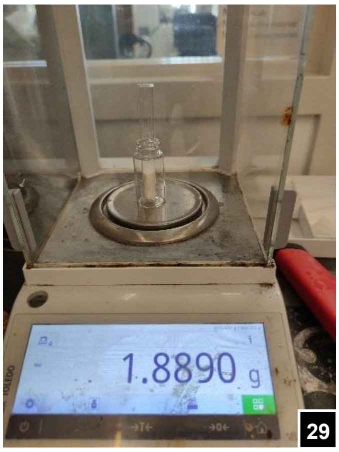

Left: The reaction crude was transferred to the round-bottom flask. **Center:** TBAF (1 M in THF) (14 mL, 14 mmol). **Right:** NCS (1.89 g, 14.2 mmol).

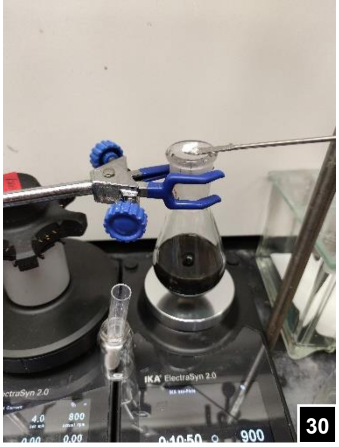

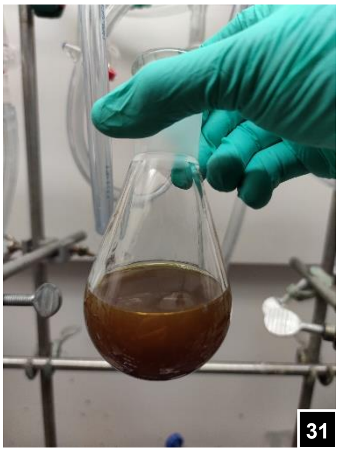

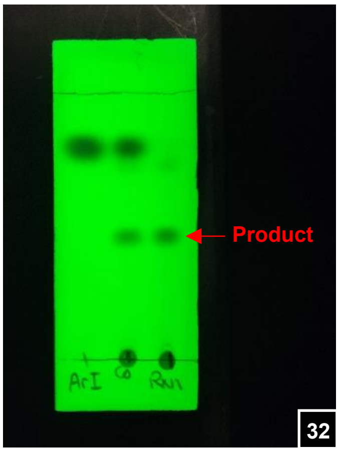

Left: NCS was added slowly with effective stirring. **Center:** After stirring for 1 h. **Right:** Crude TLC under UV (30% CH_2Cl_2 in hexane) – [from left to right] aryl iodide, co-spot, and reaction crude.

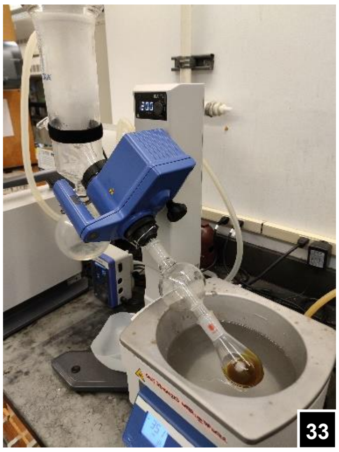

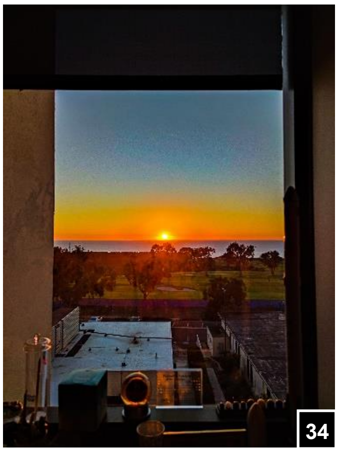

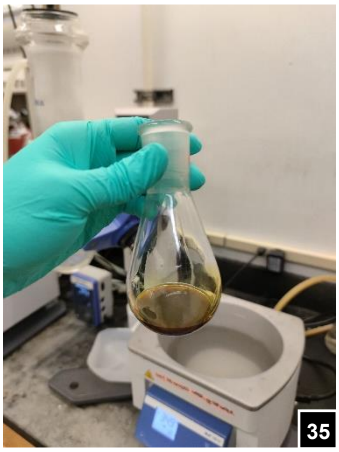

Left: The reaction mixture was concentration *in vacuo*. **Center:** Watching sunset over the Pacific in Phil's office while waiting... **Right:** Concentrated crude mixture.

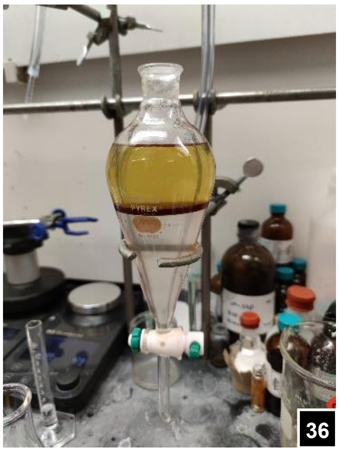

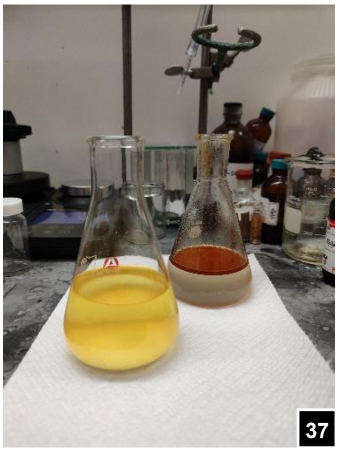

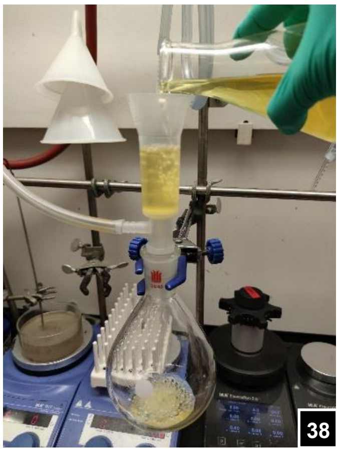

Left: The crude mixture was poured into a separating funnel, diluted with Et_2O and washed with brine. **Center:** Ethereal extract was collected and dried over anhydrous $MgSO_4$. **Right:** $MgSO_4$ was filtered off under suction, and the filtrate was concentrated *in vacuo*.

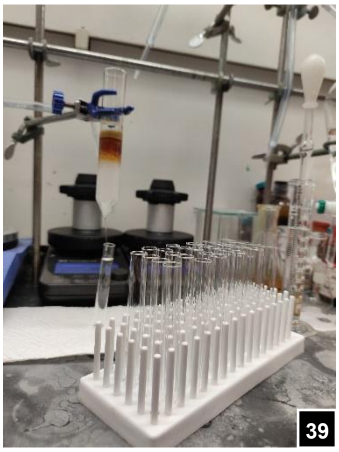

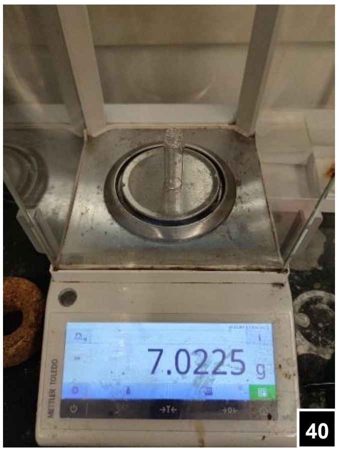

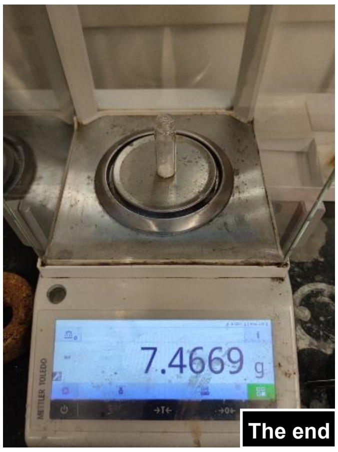

Left: The crude material was purified by flash column chromatography (0–30% CH_2Cl_2 in hexane). **Center:** Tare weight of an empty vial. **Right:** Weight of vial containing product **15** (444 mg, 53%).

10. Procedure for Multigram-Scale Reaction

As exemplified by the preparation of 4-bromo-benzenesulfonyl fluoride (**15**):

A 100-mL reagent bottle with a stir bar and electrodes (Zn anode and Ni foam cathode, 4.5 cm × 4 cm each) was capped with a screw cap that was drilled with 4 holes (one for connecting to the Schlenk line, one sealed with a rubber septum for injection, and two for connecting each of the electrodes, which was sealed with parafilm and electrical tape). The vessel was evacuated and back-filled with N_2 for three times, and charged with a solution of 1-bromo-4-iodobenzene **42** (2.83 g, 10.0 mmol, 1.0 equiv), NMPI (0.80 g, 5.0 mmol, 0.50 equiv), Ni(dtbpy)$_3$Br$_2$ (0.20 g, 0.20 mmol, 2.0 mol%), TBABr (9.70 g, 30.0 mmol, 3.0 equiv) and DIPEA (0.86 mL, 5.0 mmol, 0.50 equiv) in anhydrous CH_3CN (60 mL) and anhydrous DMA (6 mL). The solution was degassed by bubbling N_2 for 15 min, and SO_2 stock solution (8.8 M in DMA) (2.3 mL, 20.2 mmol, 2.0 equiv) was then added. The reaction mixture was electrolyzed under a constant current of 90 mA (maintaining a current density of 5 mA cm^{-2}) for 6.0 F/mol (ca. 18 h, see *Trouble Shooting & FAQ* – Q7 for further details) and a positive pressure of N_2. After the electrolysis, the screw cap was removed, the reaction mixture was transferred to a round-bottom flask, and electrodes were rinsed with CH_3CN (ca. 10 mL). TBAF (1 M in THF) (40 mL, 40 mmol, 4.0 equiv) and NCS (5.34 g, 40.0 mmol, 4.0 equiv) were then added slowly with effective stirring, and the reaction mixture was stirred at room temperature for 3 h. The crude mixture was concentrated *in vacuo*, then diluted with brine and extracted with Et_2O for three times. The combined organic extract was dried over anhydrous $MgSO_4$, filtered and concentrated *in vacuo*. The crude material was purified by flash column chromatography (10–30% CH_2Cl_2 in hexane) to afford 4-bromo-benzenesulfonyl fluoride **15** as an off-white solid (1.23 g, 51%).

Graphical Guide (Multigram-scale Reaction)

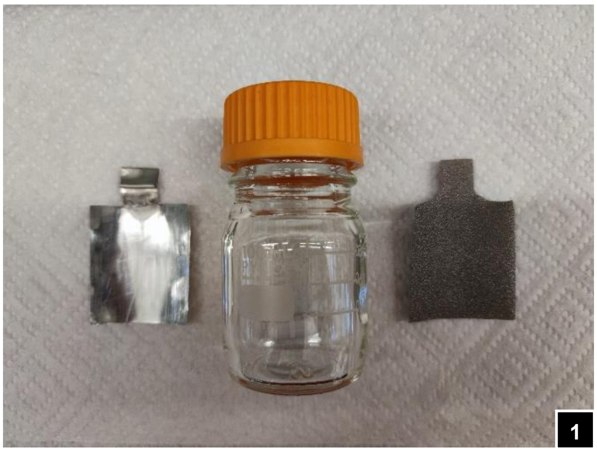

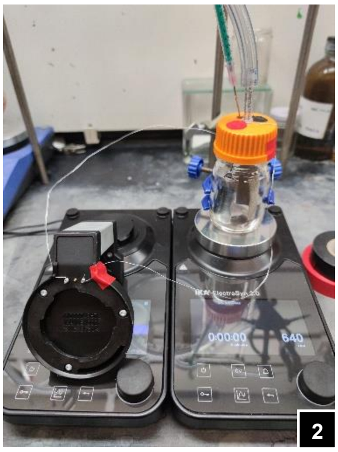

Left: Reagent bottle and electrodes for scale-up reaction. **Right:** Assembled electrochemical cell connected to the ElectraSyn vial holder for power supply. (The right ElectraSyn 2.0 was used as a stir plate.)

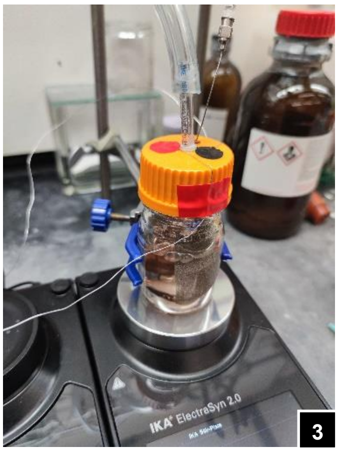

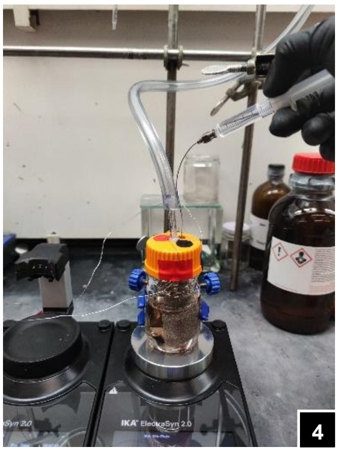

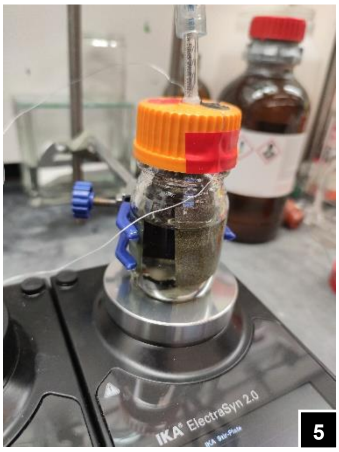

Left: Capped reagent bottle was charged with reaction mixture except SO_2, and which degassed by bubbling N_2 through a needle. **Center:** SO_2 stock solution was added. **Right:** The solution turned dark after the addition of SO_2.

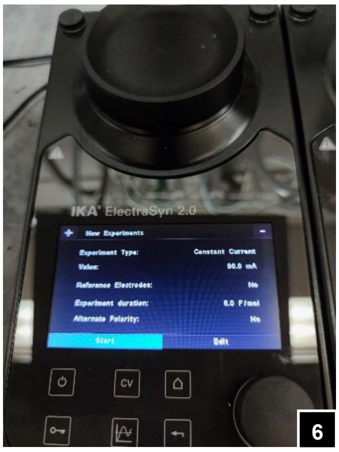

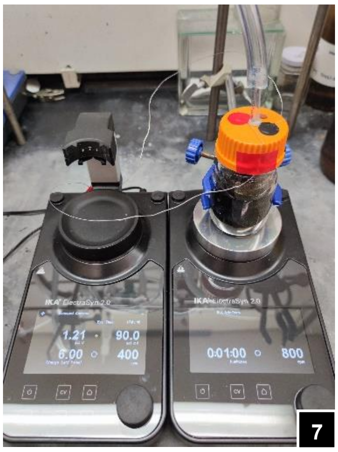

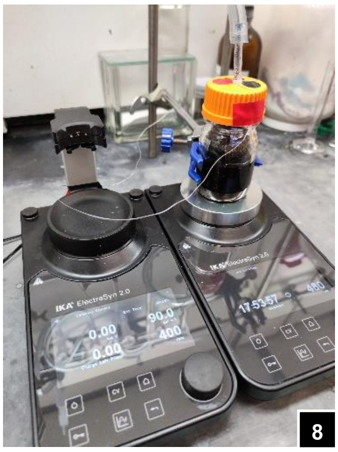

Left: Set up ElectraSyn 2.0 accordingly (other DC power source can also be used). **Center:** Electrolysis started. **Right:** E-sulfonylation completed in about 18 h.

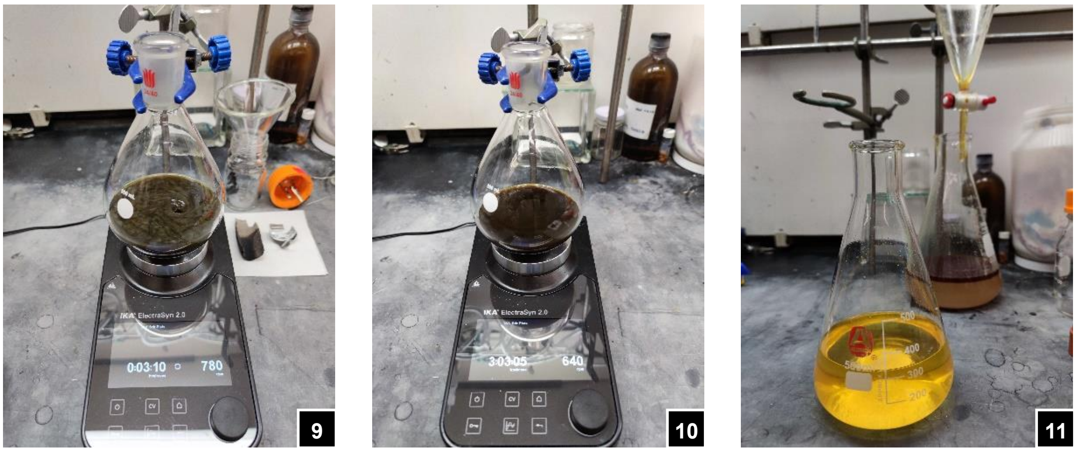

Left: Reaction mixture was transferred to a round-bottom flask; electrodes were rinsed with CH_3CN; TBAF and NCS were added subsequently. **Center:** Reaction was stirred for 3 h. **Right:** The crude mixture was concentrated *in vacuo*, then diluted with brine and extracted with Et_2O for three times. The combined ethereal extract was dried over anhydrous $MgSO_4$ as shown, which was then filtered and concentrated *in vacuo*.

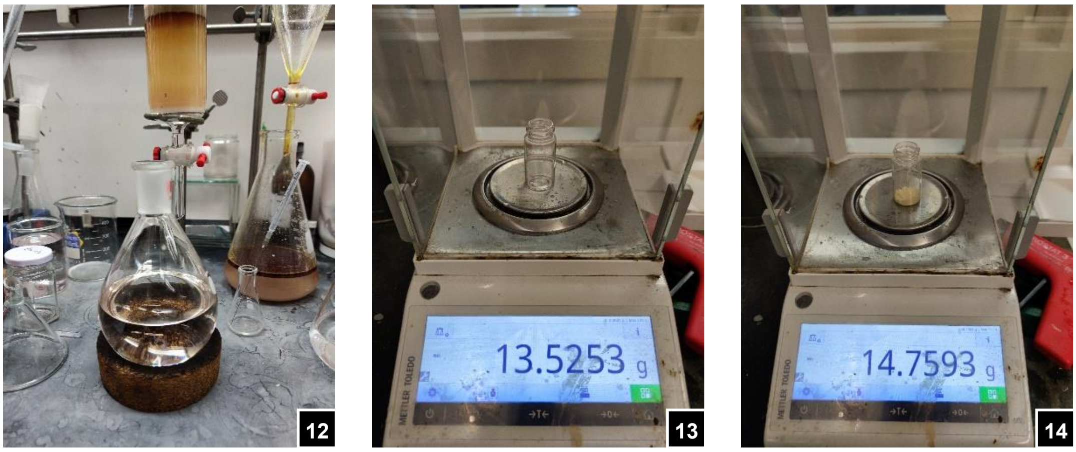

Left: The crude material was purified by flash column chromatography (10–30% CH_2Cl_2 in hexane). **Center:** Tare weight of an empty vial. **Right:** Weight of vial containing product **15** (1.23 g, 51%).

11. Procedure for e-Sulfonylation in Flow

As exemplified by the preparation of 4-bromo-benzenesulfonyl fluoride (**15**):

Flow cell setup

The flow cell was assembled using the following components: 8 hex socket cap screws with nuts (304 stainless steel, length: 108 mm, thread diameter/outer diameter (OD): 8 mm), 8 rubber O-rings (inner diameter (ID) 8 mm), 2 stainless steel pressure plates (180 mm × 120 mm × 10 mm), 4 fluororubber gaskets (with a space of 130 mm × 70 mm × 2 mm), 1 polytetrafluoroethylene (PTFE) frame (with a channel size of 130 mm × 70 mm × 18 mm), 1 graphite current collector plate (180 mm × 120 mm × 10 mm) for cathodic connection, 1 nickel foam as cathode (145 mm × 85 mm × 3 mm), and 1 zinc plate as anode (180 mm × 120 mm × 2 mm).

The cell was assembled from bottom to top: all the 8 cap screws were wrapped with TPAE tape and inserted through a stainless steel pressure plate (back plate), followed by a fluororubber gasket and graphite plate. A piece of Ni foam was laid at the center of the graphite plate with a fluororubber gasket on top to hold the Ni foam in place. The PTFE frame, Zn electrode and stainless steel pressure plate (front plate) were then layered sequentially, with gaskets inserted between each layer according to the scheme below. A rubber O-ring and hex nut were added onto each of the cap screws before tightening the entire assembly with an Allen key.

Preparation for the flow experiment

A 500-mL three-neck round-bottom flask (reservoir) was charged with 1-bromo-4-iodobenzene **42** (12.7 g, 45.0 mmol, 1.0 equiv), NMPI (3.62 g, 22.5 mmol, 0.50 equiv), Ni(dtbpy)$_3$Br$_2$ (0.92 g, 0.90 mmol, 2.0 mol%) and TBABr (43.5 g, 135 mmol, 3.0 equiv), sealed with rubber septa, and evacuated and back-filled with N_2 for three times. Anhydrous CH_3CN (400 mL), anhydrous DMA (45 mL) and DIPEA (3.85 mL, 22.5 mmol, 0.50 equiv) were added subsequently, and the mixture was stirred for 10 min until all solid dissolved.

A peristaltic pump (Part No. VSH-A603150R, ANKO®) equipped with Norprene® tubing (3/8" ID, 9.5 mm; 5/8" OD, 15.8 mm; Part No. T63-N1R50, ANKO®) was installed and connected to the assembled flow reactor inlet by copper tubing (1/4" OD, 6.4 mm) with compression fittings (1/4" OD) on both ends, with a short piece of silicone tubing (ca. 8 mm) wrapped around the fittings for a sealed connection. The fitting on the flow cell was secured with a short piece of Norprene® tubing (ca. 20 mm). The outlet of the flow cell was connected and secured to copper tubing (1/4" OD) in a similar fashion. The inlet of the peristaltic pump was connected to a polypropylene tube (3/8" OD) with an adapter.

The flow system was flushed with N_2 by inserting the polypropylene tube (inlet) in an open conical flask with a continuous flow of N_2 and pumped for 10 min. Following the N_2 flush, the pump was turned off, and the open tubes (polypropylene inlet and copper outlet) were quickly inserted into the reservoir containing the reaction mixture, through two rubber septa respectively. The peristaltic pump was turned on, and set to a pump rate of 30 rpm (ca. 11 mL/s). The reaction mixture was then degassed and residual headspace air was removed by bubbling N_2 through a long needle inserted into the third neck of the reservoir with a needle vent. After 15 min of degassing, the long needle was lifted to the headspace of the reservoir and the vent was removed, a stock solution of SO_2 (8.8 M in DMA) (10.3 mL, 90.6 mmol, 2.0 equiv) was then added. The flow system was pumped for another 15 min to ensure the homogeneity of the mixture.

Electrolysis in flow

With the peristaltic pump turned on (30 rpm, ca 11 mL/s, counter-clockwise) and the reaction mixture flowing through the system, the electrodes were connected to a direct current power source (Model No. KA3005D, KORAD®) via Alligator clips: red to the Zn anode (+) and black to the graphite current collector plate, which passes current to Ni foam cathode (−). A constant current of 0.45 A was applied. After 6 F/mol (ca. 16 h, see *Trouble Shooting & FAQ* – Q7 for further details), TLC was taken to confirm the complete consumption of starting material before disconnecting from the power source. The reaction mixture was collected by reversing the direction of the flow (clockwise) and lifting the copper tubing out of the reaction mixture to allow drainage. The flow system was then rinsed by pumping two rounds of CH_3CN (250 mL each) through the flow cell and tubes for 5 min. The solution was combined with the reaction mixture and transferred to a 2-L round-bottom flask for subsequent fluorination.

Fluorination, work up and purification

To the combined mixture from the electrolysis, TBAF (1 M in THF) (180 mL, 180 mmol, 4.0 equiv) and NCS (24.0 g, 40.0 mmol, 4.0 equiv) were then slowly added with effective stirring. The reaction mixture was stirred at room temperature for 3 h. The resulting solution was concentrated *in vacuo*, then diluted with brine (500 mL) and extracted with Et_2O (300 mL) three times. The combined organic extract was dried over anhydrous $MgSO_4$, filtered and concentrated *in vacuo*. The crude material was purified by flash column chromatography (10–25% CH_2Cl_2 in hexane) to afford 4-bromo-benzenesulfonyl fluoride **15** as an off-white solid (5.43 g, 50%).

Graphical Guide (Flow Reaction)

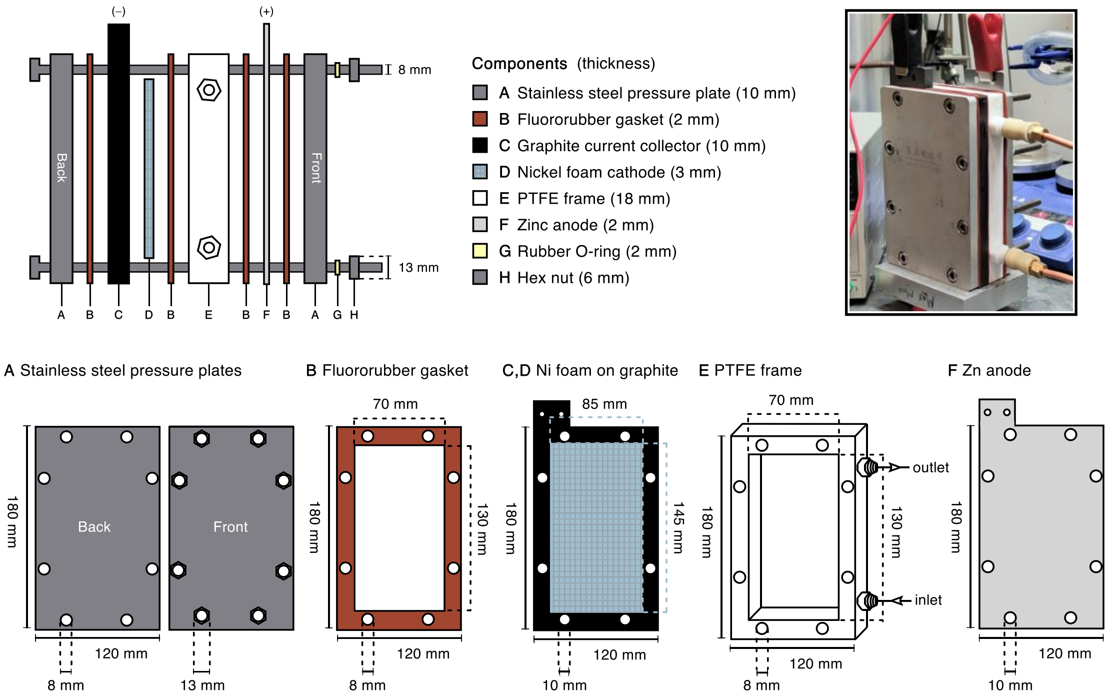

Schematic diagram of flow cell components and their dimensions.

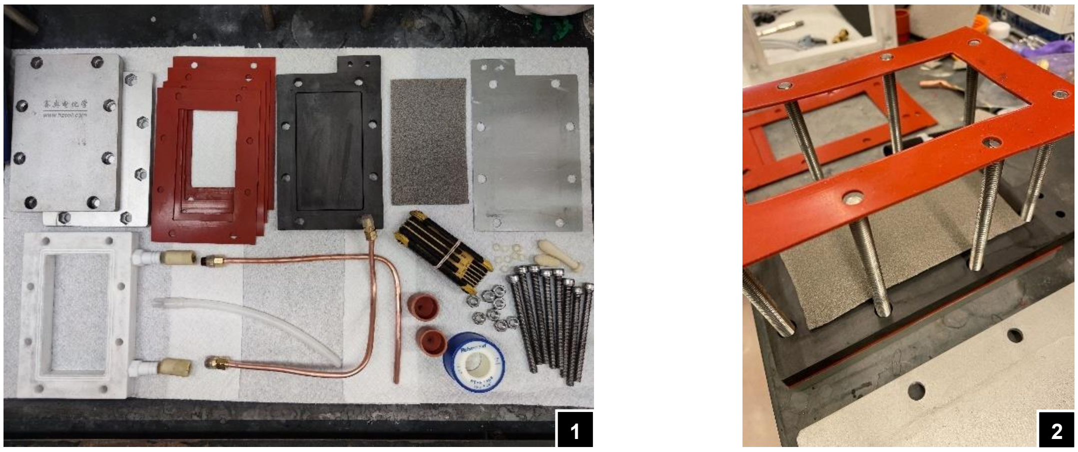

Left: Components for building the flow cell. **Right:** Assembling the flow cell. Ni foam cathode was placed at the center of the graphite current collector plate, with a fluororubber gasket on top to hold the Ni foam in place.

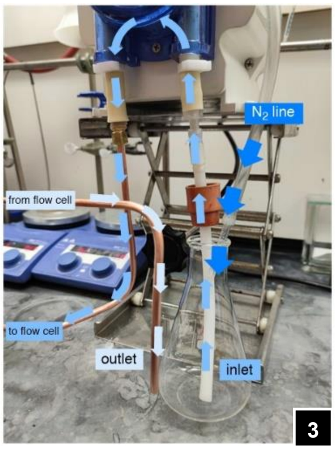

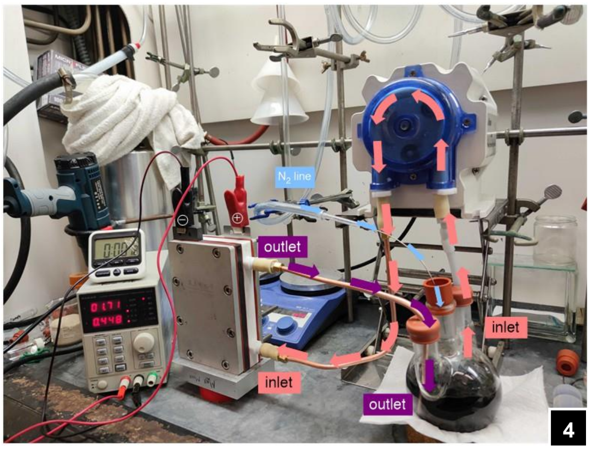

Left: Flushing the flow system with N_2. **Right:** Inlet and outlet tubes inserted into the reservoir. Peristaltic pump was turned on (flow rate ~11 mL/s). Reaction mixture was degassed for 15 min. SO_2 stock solution was then added, and the electrodes were connected to power source after 15 min. Arrows indicate the direction of flow.

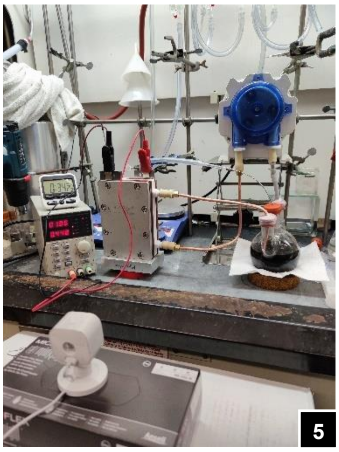

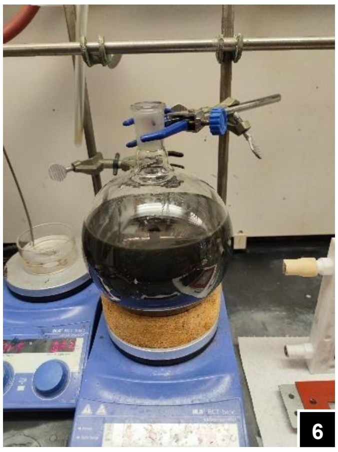

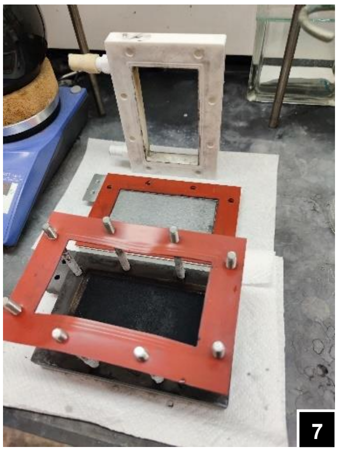

Left: The recycle flow reaction was conducted for 16 h and monitored by a Blink Mini camera. **Center:** Flow system was rinsed and combined mixture was transferred to a 2-L round-bottom flask for fluorination. **Right:** Disassembled flow cell.

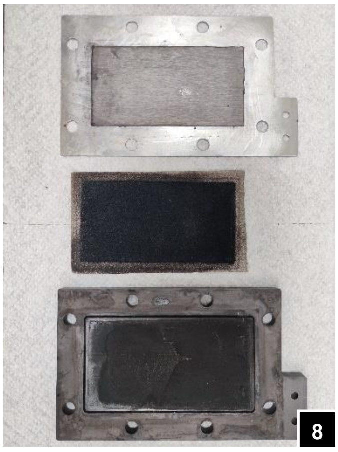

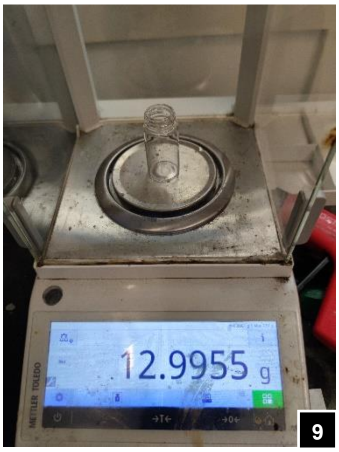

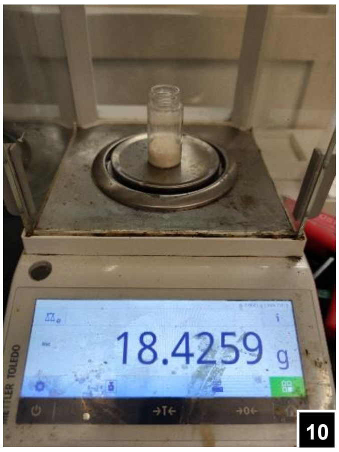

Left: Electrodes after the electrolysis. **Center:** Tare weight of an empty vial. **Right:** Weight of vial containing product **15** (5.43 g, 50%).

Discussion

Choice of tubing material

Copper tubing was chosen for connecting the peristaltic pump with the flow cell, and the flow cell with the reservoir, due to its chemical compatibility and ductility which allows it to be shaped into desirable angles. To avoid any possible hard collisions with the reservoir caused by the mechanical movement of the peristaltic pump, polypropylene tubing was used. A few different rubber and elastomer tubing materials were tested and found unsuitable as they swelled and became brittle when in contact with acetonitrile or the reaction mixture overnight. For instance, when Fluran® tubing was employed in the flow system as described, as much as 30% of solvent was lost after 16 h, with the best yield of 18% recorded. This suggested the possibility of solvent evaporation, as well as SO_2 and air leakage, through the tubing.

Avoiding leakage

The general rule of thumb to avoid leakage is to alternate layers of hard and soft materials, e.g. fluororubber gaskets were inserted between metal plates, electrodes, and the PTFE frame. Leakage is commonly observed at the joints of tubing, and through the screw holes of the flow cell. To secure and seal the joints, additional soft tubing materials can be added and tightened with cable ties. In addition to wrapping the screws with TPFE tape, adding rubber O-rings (made from pipette bulbs) to the screws prior to securing with hex nuts was found to be effective in preventing leaks through the screw holes. Additionally, ensuring that the Ni foam is cut to the right dimensions is important. If the Ni foam electrode is not centered on the graphite plate or is too large (i.e. overlapping with the screw holes), it can be a source of leakage as well.

Avoiding short circuit

Short circuiting is possible when the screws come into direct contact with the cathode and anode. In this case, while the current and voltage readings on the DC power source might appear to be normal, electrolysis would not take place. Wrapping all screws with TPFE tape can avoid a short circuit.

Use of current collector

Ni foam, as the name suggested, is a porous material. Therefore, opting to use a full-size Ni foam electrode would likely lead to leakage at the edges of the flow cell and is not recommended. A graphite plate was used as current collector to connect the negative terminal of the power supply with the Ni foam cathode sealed in the flow cell. Since Ni foam has a high surface area and is more conductive than graphite, cathodic reduction is expected to take place on Ni instead of graphite.

12. Reaction Scope of Sulfonyl Fluorides

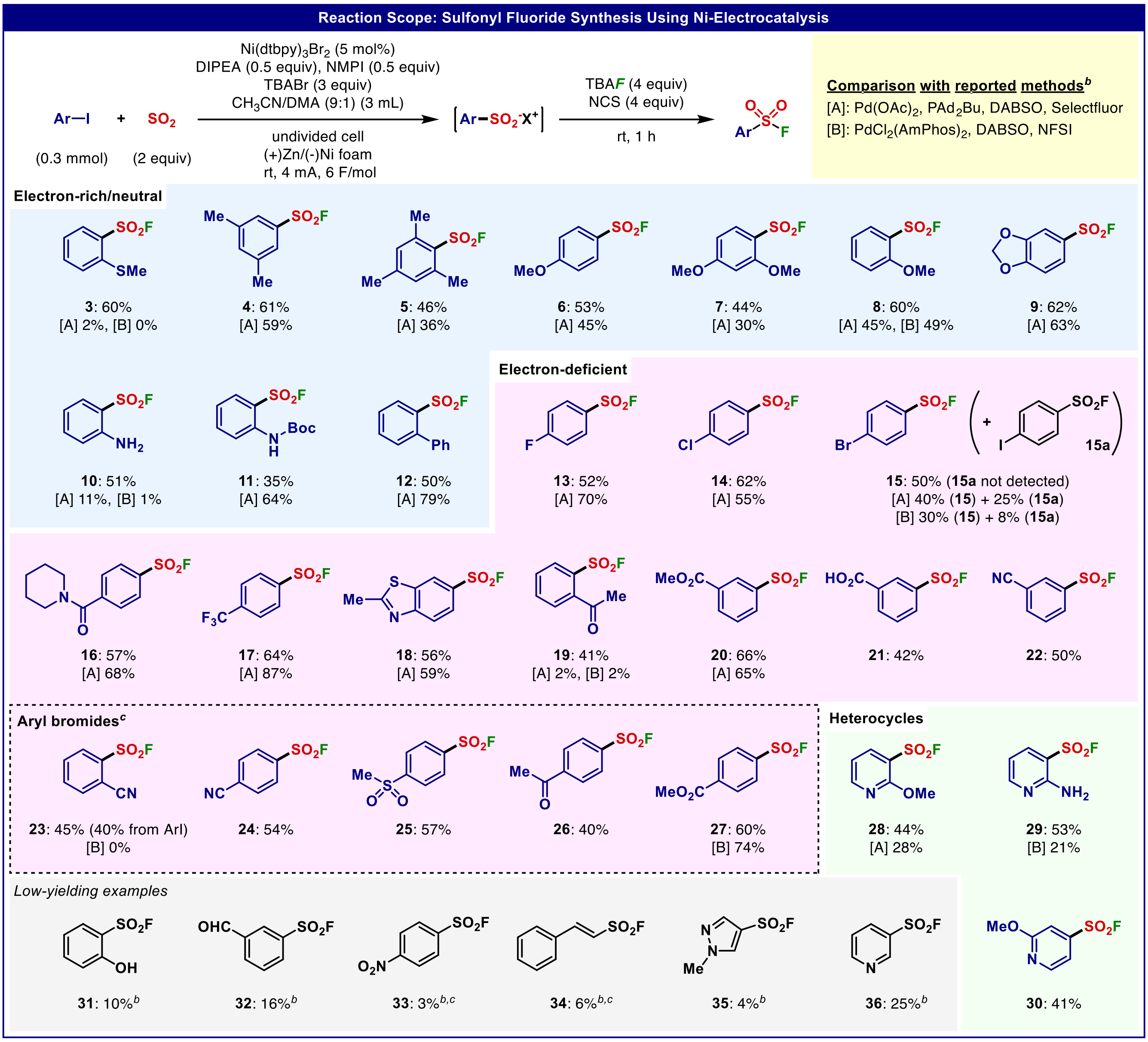

[a] Isolated yields presented. [b] ^{19}F-NMR yields. [c] NFSI (1.5 equiv) was used in fluorination in place of TBAF and NCS.

Note:

For low-yielding examples **31–36**, proto-dehalogenation was found to be dominant. For aryl bromides with electron-donating or electron-neutral substituents, little to no conversions were observed.

13. Experimental Procedures and Characterization Data

2-(Methylthio)benzenesulfonyl fluoride (3)

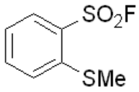

Prepared according to General Procedure A using 2-iodothioanisole (75.0 mg, 0.30 mmol). The crude product was purified by preparative thin-layer chromatography (30% CH_2Cl_2 in hexane) to afford the *aryl sulfonyl fluoride* **3** as an off-white solid (37.2 mg, 60%);

^{1}H NMR (400 MHz, $CDCl_3$) δ 8.05 (d, J = 8.0 Hz, 1H), 7.66 (t, J = 7.8 Hz, 1H), 7.43 (d, J = 8.0 Hz, 1H), 7.32 (t, J = 7.8 Hz, 1H), 2.58 (s, 3H);

^{13}C {^{1}H} NMR (101 MHz, $CDCl_3$) δ 142.0, 135.3, 131.5, 130.4 (d, J = 22.7 Hz), 127.1, 124.8, 16.2;

^{19}F {^{1}H} NMR (377 MHz, $CDCl_3$) δ 57.7;

HRMS (APCI-TOF) calc'd for $C_7H_7FO_2S_2$ $[M]^+$: 205.9871; found 205.9868.

3,5-Dimethylbenzenesulfonyl fluoride (4)

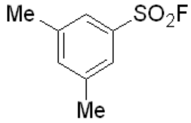

Prepared according to General Procedure A using 1-iodo-3,5-dimethylbenzene (69.6 mg, 0.30 mmol). The crude product was purified by flash column chromatography (0–20% CH_2Cl_2 in pentane) to afford the aryl sulfonyl fluoride **4** as an off-white solid (34.3 mg, 61%) with spectroscopic data in accordance with the literature[2];

^{1}H NMR (400 MHz, $CDCl_3$) δ 7.62 (s, 2H), 7.38 (s, 1H), 2.43 (s, 6H);

^{13}C {^{1}H} NMR (101 MHz, $CDCl_3$) δ 140.1, 137.4, 132.9 (d, J = 22.9 Hz), 126.0, 21.3;

^{19}F {^{1}H} NMR (377 MHz, $CDCl_3$) δ 65.8;

HRMS (ESI or APCI) not found.

2,4,6-Trimethylbenzenesulfonyl fluoride (5)

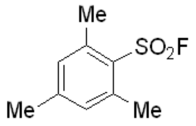

Prepared according to General Procedure A using 2-iodo-1,3,5-trimethylbenzene (73.8 mg, 0.30 mmol). The crude product was purified by preparative thin-layer chromatography (10% EtOAc in hexane) to afford the aryl sulfonyl fluoride **5** as a white solid (27.7 mg, 46%) with spectroscopic data in accordance with the literature[7];

^{1}H NMR (400 MHz, $CDCl_3$) δ 7.03 (s, 2H), 2.64 (s, 6H), 2.35 (s, 3H);

^{13}C {^{1}H} NMR (101 MHz, $CDCl_3$) δ 145.2, 140.2, 132.0, 129.2 (d, J = 20.6 Hz), 22.5, 21.3;

^{19}F {^{1}H} NMR (377 MHz, $CDCl_3$) δ 68.3;

HRMS (ESI or APCI) not found.

4-Methoxybenzenesulfonyl fluoride (6)

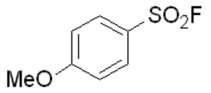

Prepared according to General Procedure A using 1-iodo-4-methoxybenzene (70.2 mg, 0.30 mmol). The crude product was purified by flash column chromatography (10–30% CH_2Cl_2 in hexane) to afford the aryl sulfonyl fluoride **6** as a white solid (30.3 mg, 53%) with spectroscopic data in accordance with the literature[1];

^{1}H NMR (400 MHz, $CDCl_3$) δ 7.95 (d, J = 8.7 Hz, 2H), 7.06 (d, J = 8.7 Hz, 2H), 3.92 (s, 3H);

^{13}C {^{1}H} NMR (101 MHz, $CDCl_3$) δ 165.3, 131.0, 124.2 (d, J = 24.5 Hz), 115.0, 56.1;

^{19}F {^{1}H} NMR (377 MHz, $CDCl_3$) δ 67.4;

HRMS (ESI-TOF) calc'd for $C_7H_7FO_3S$ $[M]^+$: 190.0100; found 190.0100.

2,4-Dimethoxybenzenesulfonyl fluoride (7)

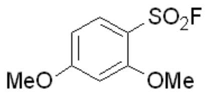

Prepared according to General Procedure A using 1-iodo-2,4-dimethoxybenzene (79.2 mg, 0.30 mmol). The crude product was purified by preparative thin-layer chromatography (33% CH_2Cl_2 in hexane) to afford the aryl sulfonyl fluoride **7** as a white solid (28.8 mg, 44%) with spectroscopic data in accordance with the literature[8];
^{1}H NMR (400 MHz, $CDCl_3$) δ 7.86 (d, *J* = 9.0 Hz, 1H), 6.57 (dd, *J* = 9.0, 2.0 Hz, 2H), 6.54 (d, *J* = 2.0 Hz, 1H), 3.97 (s, 3H), 3.90 (s, 3H);
^{13}C {^{1}H} NMR (101 MHz, $CDCl_3$) δ 167.1, 160.0, 133.4, 113.4 (d, *J* = 25.0 Hz), 105.0, 99.6, 56.6, 56.1;
^{19}F {^{1}H} NMR (377 MHz, $CDCl_3$) δ 59.9;
HRMS (APCI-TOF) calc'd for $C_8H_9FO_4S$ $[M]^+$: 220.0206; found 220.0218.

2-Methoxybenzenesulfonyl fluoride (8)

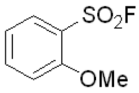

Prepared according to General Procedure A using 1-iodo-2-methoxybenzene (70.2 mg, 0.30 mmol). The crude product was purified by flash column chromatography (10–30% CH_2Cl_2 in pentane) to afford the aryl sulfonyl fluoride **8** as an off-white solid (34.0 mg, 60%) with spectroscopic data in accordance with the literature[2];
^{1}H NMR (400 MHz, $CDCl_3$) δ 7.93 (dd, *J* = 8.0, 1.9 Hz, 1H), 7.70 (t, *J* = 8.0 Hz, 1H), 7.14–7.08 (m, 2H), 4.01 (s, 3H);
^{13}C {^{1}H} NMR (101 MHz, $CDCl_3$) δ 158.2, 137.5, 131.3, 121.5 (d, *J* = 23.2 Hz), 120.6, 112.9, 56.6;
^{19}F {^{1}H} NMR (377 MHz, $CDCl_3$) δ 58.7;
HRMS (ESI or APCI) not found.

Benzo[d][1,3]dioxole-5-sulfonyl fluoride (9)

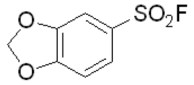

Prepared according to General Procedure A using 5-iodo-1,3-benzodioxole (74.4 mg, 0.30 mmol). The crude product was purified by preparative thin-layer chromatography (40% CH_2Cl_2 in hexane) to afford the aryl sulfonyl fluoride **9** as a white solid (37.7 mg, 62%) with spectroscopic data in accordance with the literature[1];

^{1}H NMR (400 MHz, $CDCl_3$) δ 7.60 (dd, J = 8.3, 2.0 Hz, 1H), 7.37 (s, 1H), 6.97 (d, J = 8.3 Hz, 1H), 6.16 (s, 2H);

^{13}C {^{1}H} NMR (101 MHz, $CDCl_3$) δ 154.0, 148.8, 125.7 (d, J = 24.7 Hz), 125.4, 108.9, 108.3, 103.1;

^{19}F {^{1}H} NMR (377 MHz, $CDCl_3$) δ 67.0;

HRMS (ESI or APCI) not found.

2-Aminobenzenesulfonyl fluoride (10)

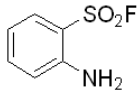

Prepared according to General Procedure A using 2-iodoaniline (65.7 mg, 0.30 mmol). The crude product was purified by preparative thin-layer chromatography (60% CH_2Cl_2 in hexane, with 1% Et_3N) to afford the *aryl sulfonyl fluoride* **10** as a red solid (26.8 mg, 51%);

^{1}H NMR (400 MHz, $CDCl_3$) δ 7.74 (d, J = 8.1 Hz, 1H), 7.44 (t, J = 7.8 Hz, 1H), 6.84–6.77 (m, 2H), 5.12 (s, 2H);

^{13}C {^{1}H} NMR (101 MHz, $CDCl_3$) δ 147.3, 137.1, 130.6, 117.8, 117.6, 113.4 (d, J = 21.9 Hz);

^{19}F {^{1}H} NMR (377 MHz, $CDCl_3$) δ 63.8;

HRMS (APCI-TOF) calc'd for $C_6H_6FNO_2S$ $[M]^+$: 175.0103; found 175.0103.

***tert*-Butyl (2-(fluorosulfonyl)phenyl)carbamate (11)**

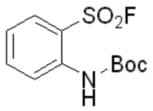

Prepared according to General Procedure A using *tert*-butyl (2-iodophenyl)carbamate (95.7 mg, 0.30 mmol). The crude product was purified by preparative thin-layer chromatography (50% CH_2Cl_2 in hexane) to afford the *aryl sulfonyl fluoride* **11** as a white solid (28.6 mg, 35%);

^{1}H NMR (400 MHz, $CDCl_3$) δ 8.46 (d, J = 8.7 Hz, 1H), 8.19 (s, 1H), 7.93 (dd, J = 8.2, 1.6 Hz, 1H), 7.69 (ddd, J = 8.7, 7.2, 1.6 Hz, 1H), 7.18 (dd, J = 8.2, 7.2 Hz, 1H), 1.53 (s, 9H);

^{13}C {^{1}H} NMR (101 MHz, $CDCl_3$) δ 152.0, 139.1, 137.3, 130.5, 122.7, 121.3, 118.9 (d, J = 22.3 Hz), 82.3, 28.3;

^{19}F {^{1}H} NMR (377 MHz, $CDCl_3$) δ 65.5;

HRMS (APCI-TOF) calc'd for $C_6H_7FNO_2S$ [M–Boc+2H]$^+$: 176.0182; found 176.0179.

[1,1'-Biphenyl]-2-sulfonyl fluoride (12)

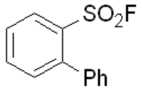

Prepared according to General Procedure A using 2-iodo-1,1'-biphenyl (84.0 mg, 0.30 mmol). The crude product was purified by preparative thin-layer chromatography (10% CH_2Cl_2 in hexane) to afford the aryl sulfonyl fluoride **12** as a white solid (35.2 mg, 50%) with spectroscopic data in accordance with the literature[9];

^{1}H NMR (400 MHz, $CDCl_3$) δ 8.18 (d, J = 8.0 Hz, 1H), 7.77 (t, J = 7.6 Hz, 1H), 7.60 (t, J = 7.8 Hz, 1H), 7.50–7.44 (m, 4H), 7.40–7.36 (m, 2H);

^{13}C {^{1}H} NMR (101 MHz, $CDCl_3$) δ 143.2, 138.0, 134.9, 133.2, 132.4 (d, J = 21.8 Hz), 130.1, 129.1, 128.7, 128.2, 128.1;

^{19}F {^{1}H} NMR (377 MHz, $CDCl_3$) δ 67.6;

HRMS (APCI-TOF) calc'd for $C_{12}H_9FO_2S$ [M]$^+$: 236.0307; found 236.0300.

4-Fluorobenzenesulfonyl fluoride (13)

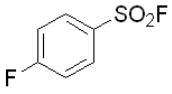

Prepared according to General Procedure A using 1-fluoro-4-iodobenzene (66.6 mg, 0.30 mmol). The crude product was purified by flash column chromatography (0–20% CH_2Cl_2 in pentane) to afford the aryl sulfonyl fluoride **13** as a light yellow oil (27.8 mg, 52%) with spectroscopic data in accordance with the literature[10];

^{1}H NMR (400 MHz, $CDCl_3$) δ 8.06 (dd, *J* = 8.4, 4.7 Hz, 2H), 7.32 (t, *J* = 8.4 Hz, 2H);

^{13}C {^{1}H} NMR (101 MHz, $CDCl_3$) δ 167.0 (d, *J* = 259.9 Hz), 131.7 (d, *J* = 10.1 Hz), 129.1 (d, *J* = 24.8 Hz), 117.4 (d, *J* = 23.1 Hz);

^{19}F {^{1}H} NMR (377 MHz, $CDCl_3$) δ 66.9 (1F), -99.2 (1F);

HRMS (ESI or APCI) not found.

4-Chlorobenzenesulfonyl fluoride (14)

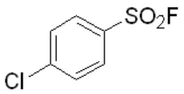

Prepared according to General Procedure A using 1-chloro-4-iodobenzene (71.5 mg, 0.30 mmol). The crude product was purified by preparative thin-layer chromatography (30% CH_2Cl_2 in hexane) to afford the aryl sulfonyl fluoride **14** as a white solid (36.4 mg, 62%) with spectroscopic data in accordance with the literature[10];

^{1}H NMR (400 MHz, $CDCl_3$) δ 7.95 (d, *J* = 8.4 Hz, 2H), 7.61 (d, *J* = 8.4 Hz, 2H);

^{13}C {^{1}H} NMR (101 MHz, $CDCl_3$) δ 142.8, 131.5 (d, *J* = 25.8 Hz), 130.3, 130.0;

^{19}F {^{1}H} NMR (377 MHz, $CDCl_3$) δ 66.6;

HRMS (ESI or APCI) not found.

4-Bromobenzenesulfonyl fluoride (15)

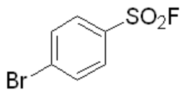

Prepared according to General Procedure A using 1-bromo-4-iodobenzene (84.9 mg, 0.30 mmol). The crude product was purified by preparative thin-layer chromatography (30% CH_2Cl_2 in hexane) to afford the aryl sulfonyl fluoride **15** as a white solid (35.6 mg, 50%) with spectroscopic data in accordance with the literature[11];

^{1}H NMR (400 MHz, $CDCl_3$) δ 7.87 (d, J = 8.3 Hz, 2H), 7.78 (d, J = 8.3 Hz, 2H);

^{13}C {^{1}H} NMR (101 MHz, $CDCl_3$) δ 133.3, 132.1(d, J = 25.5 Hz), 131.5, 130.0;

^{19}F {^{1}H} NMR (377 MHz, $CDCl_3$) δ 66.5;

HRMS (ESI or APCI) not found.

4-(Piperidine-1-carbonyl)benzenesulfonyl fluoride (16)

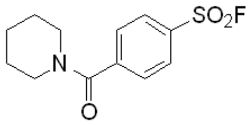

Prepared according to General Procedure A using (4-iodophenyl)(piperidin-1-yl)-methanone (94.5 mg, 0.30 mmol). The crude product was purified by preparative thin-layer chromatography (50% EtOAc in hexane, and then 20% acetone in hexane) to afford the *aryl sulfonyl fluoride* **16** as a white solid (46.5 mg, 57%);

^{1}H NMR (400 MHz, $CDCl_3$) δ 8.05 (d, J = 7.6 Hz, 2H), 7.62 (d, J = 7.6 Hz, 2H), 3.75–3.68 (m, 2H), 3.30–3.24 (m, 2H), 1.72–1.66 (m, 4H), 1.56–1.49 (m, 2H);

^{13}C {^{1}H} NMR (101 MHz, $CDCl_3$) δ 167.6, 143.9, 133.7 (d, J = 25.1 Hz), 128.9, 128.1, 48.7, 43.3, 26.6, 25.6, 24.5;

^{19}F {^{1}H} NMR (377 MHz, $CDCl_3$) δ 66.2;

HRMS (APCI-TOF) calc'd for $C_{12}H_{15}FNO_3S$ $[M+H]^+$: 272.0757; found 272.0757.

4-(Trifluoromethyl)benzenesulfonyl fluoride (17)

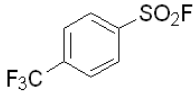

Prepared according to General Procedure A using 4-iodobenzotrifluoride (81.6 mg, 0.30 mmol). The crude product was purified by flash column chromatography (10–30% CH_2Cl_2 in pentane) to afford the aryl sulfonyl fluoride **17** as a white solid (43.8 mg, 64%) with spectroscopic data in accordance with the literature[10];

^{1}H NMR (400 MHz, $CDCl_3$) δ 8.17 (d, *J* = 8.3 Hz, 2H), 7.92 (d, *J* = 8.3 Hz, 2H);

^{13}C {^{1}H} NMR (101 MHz, $CDCl_3$) δ 137.3 (q, *J* = 34.0 Hz), 136.6 (d, *J* = 26.0 Hz), 129.3, 127.0 (q, *J* = 3.6 Hz), 122.9 (q, *J* = 272.5 Hz);

^{19}F {^{1}H} NMR (377 MHz, $CDCl_3$) δ 65.7 (1F), -63.6 (3F);

HRMS (ESI or APCI) not found.

2-Methylbenzo[d]thiazole-6-sulfonyl fluoride (18)

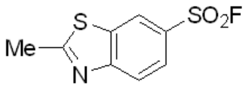

Prepared according to General Procedure A using 6-iodo-2-methyl-1,3-benzothiazole (82.5 mg, 0.30 mmol). The crude product was purified by preparative thin-layer chromatography (30% EtOAc in hexane) to afford the *aryl sulfonyl fluoride* **18** as a white solid (38.6 mg, 56%);

^{1}H NMR (400 MHz, $CDCl_3$) δ 8.54 (d, *J* = 1.8 Hz, 1H), 8.13 (d, *J* = 8.7 Hz, 1H), 8.05 (dd, *J* = 8.7, 1.8 Hz, 1H), 2.93 (s, 3H);

^{13}C {^{1}H} NMR (101 MHz, $CDCl_3$) δ 173.8, 157.8, 136.6, 128.8 (d, *J* = 25.1 Hz), 125.7, 123.6, 123.3, 20.8;

^{19}F {^{1}H} NMR (377 MHz, $CDCl_3$) δ 68.0;

HRMS (APCI-TOF) calc'd for $C_8H_7FNO_2S_2$ $[M+H]^+$: 231.9902; found 231.9903.

2-Acetylbenzenesulfonyl fluoride (19)

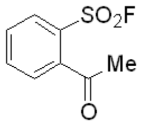

Prepared according to General Procedure A using 2'-iodoacetophenone (73.8 mg, 0.30 mmol). The crude product was purified by preparative thin-layer chromatography (70% CH_2Cl_2 in hexane) to afford the *aryl sulfonyl fluoride* **19** as a colorless oil (25.0 mg, 41%);
^{1}H NMR (400 MHz, $CDCl_3$) δ 8.12 (dd, *J* = 7.8, 1.2 Hz, 1H), 7.83 (td, *J* = 7.8, 1.2 Hz, 1H), 7.69 (t, *J* = 7.8 Hz, 1H), 7.58 (d, *J* = 7.6 Hz, 1H), 2.65 (s, 3H);
^{13}C {^{1}H} NMR (101 MHz, $CDCl_3$) δ 200.1, 142.5, 135.6, 130.8, 130.7, 130.0 (d, *J* = 26.1 Hz), 127.7, 30.6;
^{19}F {^{1}H} NMR (377 MHz, $CDCl_3$) δ 68.1;
HRMS (APCI-TOF) calc'd for $C_8H_8FO_3S$ $[M+H]^+$: 203.0178; found 203.0174.

Methyl 3-(fluorosulfonyl)benzoate (20)

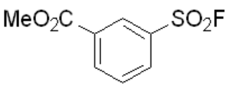

Prepared according to General Procedure A using methyl 3-iodobenzoate (78.6 mg, 0.30 mmol). The crude product was purified by preparative thin-layer chromatography (30% CH_2Cl_2 in hexane) to afford the aryl sulfonyl fluoride **20** as an off-white solid (43.3 mg, 66%) with spectroscopic data in accordance with the literature[1];
^{1}H NMR (400 MHz, $CDCl_3$) δ 8.66 (s, 1H), 8.43 (d, *J* = 8.0 Hz, 1H), 8.19 (d, *J* = 8.0 Hz, 1H), 7.75 (t, *J* = 8.0 Hz, 1H), 3.99 (s, 3H);
^{13}C {^{1}H} NMR (101 MHz, $CDCl_3$) δ 164.7, 136.5, 133.8 (d, *J* = 25.7 Hz), 132.3, 132.2, 130.2, 129.7, 53.1;
^{19}F {^{1}H} NMR (377 MHz, $CDCl_3$) δ 66.1;
HRMS (ESI or APCI) not found.

3-(Fluorosulfonyl)benzoic acid (21)

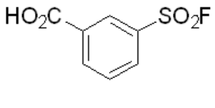

Prepared according to General Procedure A using 3-iodobenzoic acid (74.4 mg, 0.30 mmol). The crude product was purified by preparative high-performance liquid chromatography (0–99% CH_3CN in water with 1% formic acid) to afford the aryl sulfonyl fluoride **21** as a white solid (25.8 mg, 42%) with spectroscopic data in accordance with the literature[12];

^{1}H NMR (400 MHz, $CDCl_3$) δ 12.24 (br. s, 1H), 8.77 (t, *J* = 1.7 Hz, 1H), 8.53 (dt, *J* = 7.9, 1.7 Hz, 1H), 8.28 (dt, *J* = 7.9, 1.7 Hz, 1H), 7.82 (t, *J* = 7.9 Hz, 1H);

^{13}C {^{1}H} NMR (101 MHz, $CDCl_3$) δ 169.8, 137.0, 134.2 (d, *J* = 26.1 Hz), 133.3, 131.2, 130.5, 130.4;

^{19}F {^{1}H} NMR (377 MHz, $CDCl_3$) δ 66.2;

HRMS (ESI-TOF) calc'd for $C_7H_4FO_4S$ $[M-H]^-$: 202.9814; found 202.9815.

3-Cyanobenzenesulfonyl fluoride (22)

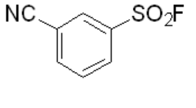

Prepared according to General Procedure A using 3-iodobenzonitrile (68.7 mg, 0.30 mmol). The crude product was purified by preparative thin-layer chromatography (50% CH_2Cl_2 in hexane) to afford the aryl sulfonyl fluoride **22** as a white solid (27.6 mg, 50%) with spectroscopic data in accordance with the literature[13];

^{1}H NMR (400 MHz, $CDCl_3$) δ 8.30 (d, *J* = 1.8 Hz, 1H), 8.25 (d, *J* = 8.0 Hz, 1H), 8.06 (d, *J* = 8.0 Hz, 1H), 7.82 (td, *J* = 8.0, 1.8 Hz, 1H);

^{13}C {^{1}H} NMR (101 MHz, $CDCl_3$) δ 138.7, 134.8 (d, *J* = 27.2 Hz), 132.4, 132.1, 131.1, 116.3, 114.8;

^{19}F {^{1}H} NMR (377 MHz, $CDCl_3$) δ 66.5;

HRMS (ESI or APCI) not found.

2-Cyanobenzenesulfonyl fluoride (23)

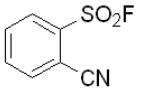

Prepared according to General Procedure A using 2-iodobenzonitrile (68.7 mg, 0.30 mmol). The crude product was purified by preparative thin-layer chromatography (50% CH_2Cl_2 in hexane) to afford the aryl sulfonyl fluoride **23** as a white solid (22.0 mg, 40%) with spectroscopic data in accordance with the literature[14]. Alternatively, General Procedure B was followed using 2-bromobenzonitrile (54.6 mg, 0.30 mmol) to afford **X** (25.1 mg, 45%).

^{1}H NMR (400 MHz, $CDCl_3$) δ 8.23 (dd, *J* = 6.8, 2.2 Hz, 1H), 8.01 (dd, *J* = 6.8, 2.2 Hz, 1H), 7.95–7.87 (m, 2H);

^{13}C {^{1}H} NMR (101 MHz, $CDCl_3$) δ 136.0, 135.7, 135.2 (d, *J* = 27.8 Hz), 133.6, 131.0, 114.2, 112.0;

^{19}F {^{1}H} NMR (377 MHz, $CDCl_3$) δ 64.6;

HRMS (ESI-TOF) calc'd for $C_7H_5FNO_2S$ $[M+H]^+$: 186.0025; found 186.0031.

4-Cyanobenzenesulfonyl fluoride (24)

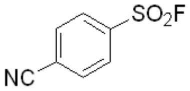

Prepared according to General Procedure B using 4-bromobenzonitrile (54.6 mg, 0.30 mmol). The crude product was purified by preparative thin-layer chromatography (50% CH_2Cl_2 in hexane) to afford the aryl sulfonyl fluoride **24** as a white solid (29.9 mg, 54%) with spectroscopic data in accordance with the literature[14];

^{1}H NMR (400 MHz, $CDCl_3$) δ 8.16 (d, *J* = 8.7 Hz, 2H), 7.95 (dd, *J* = 8.7, 0.9 Hz, 2H);

^{13}C {^{1}H} NMR (101 MHz, $CDCl_3$) δ 137.1 (d, *J* = 26.8 Hz), 133.5, 129.3, 119.6, 116.6;

^{19}F {^{1}H} NMR (377 MHz, $CDCl_3$) δ 66.1;

HRMS (ESI or APCI) not found.

4-(Methylsulfonyl)benzenesulfonyl fluoride (25)

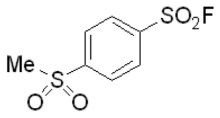

Prepared according to General Procedure B using 1-bromo-4-(methylsulfonyl)benzene (70.5 mg, 0.30 mmol). The crude product was purified by preparative thin-layer chromatography (50% EtOAc in hexane, and then 20% acetone in hexane) to afford the aryl sulfonyl fluoride **25** as a white solid (40.8 mg, 57%) with spectroscopic data in accordance with the literature[15];

^{1}H NMR (400 MHz, $CDCl_3$) δ 8.27–8.21 (m, 4H), 3.13 (s, 3H);

^{13}C {^{1}H} NMR (101 MHz, $CDCl_3$) δ 147.2, 138.1 (d, *J* = 26.6 Hz), 129.8, 129.1, 44.4;

^{19}F {^{1}H} NMR (377 MHz, $CDCl_3$) δ 66.2;

HRMS (ESI or APCI) not found.

4-Acetylbenzenesulfonyl fluoride (26)

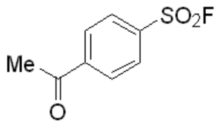

Prepared according to General Procedure B using 4'-bromoacetophenone (59.7 mg, 0.30 mmol). The crude product was purified by preparative thin-layer chromatography (67% CH_2Cl_2 in hexane) to afford the aryl sulfonyl fluoride **26** as a white solid (24.1 mg, 40%) with spectroscopic data in accordance with the literature[2];

^{1}H NMR (400 MHz, $CDCl_3$) δ 8.18 (d, *J* = 8.3 Hz, 2H), 8.13 (d, *J* = 8.3 Hz, 2H), 2.69 (s, 3H);

^{13}C {^{1}H} NMR (101 MHz, $CDCl_3$) δ 196.3, 142.3, 136.7 (d, *J* = 25.0 Hz), 129.4, 129.0, 27.1;

^{19}F {^{1}H} NMR (377 MHz, $CDCl_3$) δ 66.0;

HRMS (ESI or APCI) not found.

Methyl 4-(fluorosulfonyl)benzoate (27)

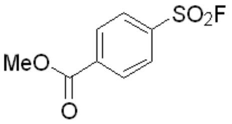

Prepared according to General Procedure B using methyl 4-bromobenzoate (64.5 mg, 0.30 mmol). The crude product was purified by preparative thin-layer chromatography (50% CH_2Cl_2 in hexane) to afford the aryl sulfonyl fluoride **27** as a white solid (39.0 mg, 60%) with spectroscopic data in accordance with the literature[1];

^{1}H NMR (400 MHz, $CDCl_3$) δ 8.28 (d, J = 8.2 Hz, 2H), 8.10 (d, J = 8.2 Hz, 2H), 3.99 (s, 3H);

^{13}C {^{1}H} NMR (101 MHz, $CDCl_3$) δ 165.1, 136.8 (d, J = 25.4 Hz), 136.6, 130.9, 128.7, 53.1;

^{19}F {^{1}H} NMR (377 MHz, $CDCl_3$) δ 65.9;

HRMS (ESI or APCI) not found.

2-Methoxypyridine-3-sulfonyl fluoride (28)

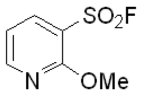

Prepared according to General Procedure A using 3-iodo-2-methoxypyridine (70.5 mg, 0.30 mmol). The crude product was purified by preparative thin-layer chromatography (20% EtOAc in hexane) to afford the *pyridine sulfonyl fluoride* **28** as a colorless oil (25.4 mg, 44%);

^{1}H NMR (400 MHz, $CDCl_3$) δ 8.50 (d, J = 5.0 Hz, 1H), 8.23 (d, J = 7.7 Hz, 1H), 7.10 (dd, J = 7.7, 5.0 Hz, 1H), 4.15 (s, 3H);

^{13}C {^{1}H} NMR (101 MHz, $CDCl_3$) δ 160.6, 154.4, 141.0, 116.8, 116.6 (d, J = 25.6 Hz), 55.2;

^{19}F {^{1}H} NMR (377 MHz, $CDCl_3$) δ 58.6;

HRMS (APCI-TOF) calc'd for $C_6H_7FNO_3S$ $[M+H]^+$: 192.0131; found 132.0130.

2-Aminopyridine-3-sulfonyl fluoride (29)

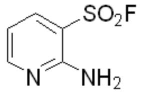

Prepared according to General Procedure A using 2-amino-3-iodopyridine (66.0 mg, 0.30 mmol). The crude product was purified by preparative thin-layer chromatography (2:2:1 hexane/CH_2Cl_2/EtOAc) to afford the *pyridine sulfonyl fluoride* **29** as a yellow solid (27.8 mg, 53%);

^{1}H NMR (400 MHz, $CDCl_3$) δ 8.39 (d, *J* = 4.7 Hz, 1H), 8.05 (d, *J* = 8.1 Hz, 1H), 6.81 (dd, *J* = 8.1, 4.7 Hz, 1H), 5.89 (s, 2H);

^{13}C {^{1}H} NMR (101 MHz, $CDCl_3$) δ 156.6, 155.9, 140.2, 113.7, 109.5 (d, *J* = 24.2 Hz);

^{19}F {^{1}H} NMR (377 MHz, $CDCl_3$) δ 64.3;

HRMS (APCI-TOF) calc'd for $C_5H_6FN_2O_2S$ $[M+H]^+$: 177.0134; found 177.0137.

2-Methoxypyridine-4-sulfonyl fluoride (30)

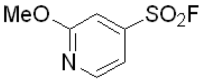

Prepared according to General Procedure A using 4-iodo-2-methoxypyridine (70.5 mg, 0.30 mmol). The crude product was purified by preparative thin-layer chromatography (67% CH_2Cl_2 in hexane) to afford the *pyridine sulfonyl fluoride* **30** as a light yellow oil (23.3 mg, 41%);

^{1}H NMR (400 MHz, $CDCl_3$) δ 8.47 (d, *J* = 5.2 Hz, 1H), 7.37 (d, *J* = 5.2 Hz, 1H), 7.30 (s, 1H), 4.02 (s, 3H);

^{13}C {^{1}H} NMR (101 MHz, $CDCl_3$) δ 165.0, 149.6, 143.4 (d, J = 27.1 Hz), 113.2, 110.4, 54.7;

^{19}F {^{1}H} NMR (377 MHz, $CDCl_3$) δ 64.0;

HRMS (APCI-TOF) calc'd for $C_6H_7FNO_3S$ $[M+H]^+$: 192.0131; found 192.0130.

Tetrabutylammonium trifluoro(4-(fluorosulfonyl)phenyl)borate (39)

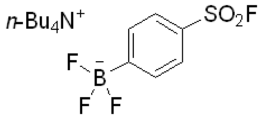

Prepared according to General Procedure A using 4-iodophenylboronic acid pinacol ester **37** (99.0 mg, 0.30 mmol). After the addition of TBAF and NCS, the reaction was stirred at room temperature for 0.5 h, followed by the addition of an aqueous solution (0.5 mL) of KHF_2 (93.7 mg, 1.2 mmol, 4.0 equiv). The reaction mixture was stirred rigorously at room temperature for a further 2 h, which was then diluted brine and extracted with EtOAc for three times. The combined organic extract was dried over anhydrous $MgSO_4$, filtered and concentrated *in vacuo*. The crude product was purified by flash column chromatography (3:1:1 then 1:1:1 hexane/CH_2Cl_2/EtOAc) to afford the *aryl sulfonyl fluoride* **39** as a colorless oil (79.6 mg, 57%);

^{1}H NMR (400 MHz, $CDCl_3$) δ 7.85 (d, J = 8.1 Hz, 2H), 7.81 (d, J = 8.1 Hz, 2H), 3.14–3.07 (m, 8H), 1.60–1.49 (m, 8H), 1.35 (sext, J = 7.3 Hz, 8H), 0.96 (t, J = 7.3 Hz, 12H);

^{13}C {^{1}H} NMR (101 MHz, $CDCl_3$) δ 132.8, 129.6 (d, J = 21.7 Hz), 126.6, 58.7, 23.9, 19.7, 13.7 (1C missing);

^{19}F {^{1}H} NMR (377 MHz, $CDCl_3$) δ 66.3 (1F), -142.6 (3F);

HRMS (APCI-TOF) calc'd for $C_6H_4{}^{10}BF_4O_2S$ $[M]^-$: 225.9998; found 226.0004.

4,4,5,5-tetramethyl-2-(4-((4-methylbenzyl)sulfonyl)phenyl)-1,3,2-dioxaborolane (40)

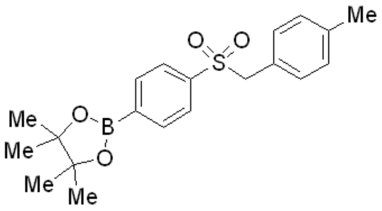

Prepared according to General Procedure A using 4-iodophenylboronic acid pinacol ester **37** (99.0 mg, 0.30 mmol). After the electrolysis, 4-methylbenzyl bromide (111 mg, 0.60 mmol, 2.0 equiv) was added instead of TBAF and NCS, and the reaction was stirred at room temperature for 1 h. The crude mixture was diluted with Et_2O and washed with brine. The organic layer was dried over anhydrous $MgSO_4$, filtered and concentrated *in vacuo*. The crude product was purified by flash column chromatography (0–30% EtOAc in hexane) to afford the *sulfone* **40** as a white solid (69.0 mg, 62%);

^{1}H NMR (400 MHz, $CDCl_3$) δ 7.86 (d, *J* = 8.2 Hz, 2H), 7.62 (d, *J* = 8.2 Hz, 2H), 7.06 (d, *J* = 7.8 Hz, 2H), 6.96 (d, *J* = 7.8 Hz, 2H), 4.26 (s, 2H), 2.32 (s, 3H), 1.36 (s, 12H);
^{13}C {^{1}H} NMR (101 MHz, $CDCl_3$) δ 140.3, 138.9, 135.2, 130.8, 129.5, 127.7, 124.9, 84.6, 62.6, 25.0, 21.4 (1C missing);
HRMS (ESI-TOF) calc'd for $C_{20}H_{25}{}^{10}BNaO_4S$ $[M+Na]^+$: 394.1501; found 394.1489.

4-((4-(4,4,5,5-tetramethyl-1,3,2-dioxaborolan-2-yl)phenyl)sulfonyl)morpholine (41)

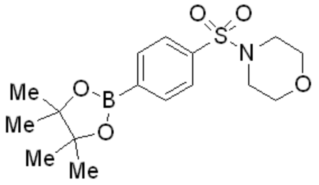

Prepared according to General Procedure A using 4-iodophenylboronic acid pinacol ester **37** (99.0 mg, 0.30 mmol). After the electrolysis, morpholine (78 μL, 0.90 mmol, 3.0 equiv) and NCS (160 mg, 1.2 mmol, 4.0 equiv) was added, and the reaction was stirred at room temperature for 1 h. The crude mixture was diluted with Et_2O and washed with brine. The organic layer was dried over anhydrous $MgSO_4$, filtered and concentrated *in vacuo*. The crude product was purified by preparative thin-layer chromatography (30% EtOAc in hexane) to afford the sulfonamide **41** as an off-white solid (53.7 mg, 51%) with spectroscopic data in accordance with the literature[16];
^{1}H NMR (400 MHz, $CDCl_3$) δ 7.96 (d, *J* = 8.3 Hz, 2H), 7.71 (d, *J* = 8.3 Hz, 2H), 3.73–3.69 (m, 4H), 2.99–2.95 (m, 4H), 1.35 (s, 12H);
^{13}C {^{1}H} NMR (101 MHz, $CDCl_3$) δ 137.3, 135.5, 126.9, 84.6, 66.2, 46.1, 25.0 (1C missing);
HRMS (ESI-TOF) calc'd for $C_{16}H_{24}{}^{10}BNO_5S$ $[M+H]^+$: 353.1583; found 353.1581.

14. Comparison Conditions

Pd-catalysis [A][2]

An oven-dried resealable screw-cap 10-mL test tube was charged with aryl iodide (0.30 mmol, 1.0 equiv), $Pd(OAc)_2$ (3.4 mg, 0.015 mmol, 5.0 mol%), PAd_2Bu (8.6 mg, 0.024 mmol, 8.0 mol%), DABSO (43.2 mg, 0.18 mmol, 0.60 equiv), and a magnetic stir bar. The tube was sealed, evacuated and back-filled with N_2 for three times. Under N_2, degassed anhydrous *i*-PrOH (1 mL, 0.3 M) and anhydrous Et_3N (0.13 mL, 0.90 mmol, 3.0 equiv) were subsequently added. The reaction was stirred in a preheated oil bath at 75 °C for 14 h, which was then cooled to room temperature. The tube was opened to air, and Selectfluor (212 mg, 0.60 mmol, 2.0 equiv) was added, followed by acetonitrile (1 mL). The reaction was stirred at room temperature for 2 h. $PhCF_3$ (12.3 μL, 0.10 mmol, 0.33 equiv) was then added as internal standard, and an aliquot of the mixture (ca. 0.1 mL) was taken and diluted with $CDCl_3$ for determining ^{19}F-NMR yield.

Pd-catalysis [B][1]

An oven-dried resealable screw-cap 10-mL test tube was charged with aryl iodide or bromide (0.30 mmol, 1.0 equiv), $PdCl_2(AmPhos)_2$ (10.6 mg, 0.015 mmol, 5.0 mol%), DABSO (43.2 mg, 0.18 mmol, 0.60 equiv), and a magnetic stir bar. The tube was sealed, evacuated and back-filled with N_2 for three times. Under N_2, degassed anhydrous *i*-PrOH (1.5 mL, 0.2 M) and anhydrous Et_3N (0.13 mL, 0.90 mmol, 3.0 equiv) were subsequently added. The reaction was stirred in a preheated oil bath at 75 °C for 24 h, which was then cooled to room temperature. The tube was opened to air, and NFSI (142 mg, 0.45 mmol, 1.5 equiv) was added. The reaction was stirred at room temperature for 3 h. $PhCF_3$ (12.3 μL, 0.10 mmol, 0.33 equiv) was then added as internal standard, and an aliquot of the mixture (ca. 0.1 mL) was taken and diluted with $CDCl_3$ for determining ^{19}F-NMR yield.

15. Trouble Shooting & FAQ

About SO_2 solution

Q1: How to prepare a saturated SO_2 solution?

A1: In short, simply by bubbling SO_2 gas in a degassed solvent – also check procedure described in previous section. Some observations would help to determine whether the solution is saturated: while the volume of the solution increases as SO_2 dissolves, the volume of a saturated solution would remain constant with further bubbling of SO_2; another noticeable difference is the size of SO_2 gas bubble, which would be significantly larger (similar to degassing with N_2) when the solution is saturated.

Q2: When titrating a SO_2 solution, what can I do if iodine crystals are poorly dissolving?

A2: Make sure KI is added. Breaking the I_2 crystals into smaller pieces or powder, and sonicating the solution would significantly help its dissolution. Avoid adding excessive water as this would make the color change less visible.

Q3: How to calculate the molarity of SO_2 from titration results?

A3: Following the described procedure (employing 0.1 mL of SO_2 solution and 0.2 M $Na_2S_2O_3$ as titrant), the calculation of SO_2 concentration can be simplified using the formula:

$$[SO_2] = (\textit{mass of } I_2\textit{, mg}) \div 25.4 - (\textit{volume of titrant, mL})$$

e.g. when 65.0 mg of I_2 was used and require 0.50 mL of titrant, the concentration of SO_2 is (65.0 ÷ 25.4) – 0.50 = 2.06 M.

Q4: How long can SO_2 stock solution be kept?

A4: SO_2 stock solution can be stored in a sealed vessel for months at 4 °C. A more dilute solution is expected to have better retention of SO_2 over time, especially in low temperature. For reference, the concentration of a SO_2 stock solution in DMA dropped from 5.6 M to 4.7 M (84% retention) over a course of 6 months with repeated usage, or from 2.1 M to 2.0 M (95% retention) over one month of repeated usage.

Q5: Why DMA was chosen as the solvent for SO_2 solution?

A5: The choice of using a SO_2 solution in DMA was based on two main reasons. First, DMA was found to be effective to in preventing electrode passivation. Second, a high concentration of SO_2 can be achieved when saturated (ca. 8 M in room temperature). A concentrated solution can be stored with good retention of SO_2, and it can be diluted into fresh smaller batches when required, hence saving the effort of handling SO_2 gas every time.

About electrolysis

Q6: What are the suitable current and voltage values for reactions on different scales?

A6: A current density of 5 mA/cm^2 should be maintained under constant current. Current density refers to current per unit area of electrode submerged into the reaction mixture, so the optimal 5 mA/cm^2 can be achieved by adjusting applied current, the size of electrodes, and volume of the reaction mixture. Using a standard IKA ElectraSyn 2.0 setup, a constant current of 4 mA is applied, and the submerged exterior area of the electrodes is ca. 1 cm × 0.8 cm in 0.3 mmol scale. The normal range of readout voltage for the reaction is 0.5–2.0 V (this value represents the terminal potential across the pair of electrodes without a reference electrode; also check out the V-t graph when a Ag/AgCl reference electrode was employed).

Q7: How to convert F/mol to time?

A7: Multiply the number of moles of substrate (in mol) by Faraday constant (96,485 C mol^{-1}) and divided by applied current (in A) gives the time (in second) required for each F/mol. Divide the number by 3,600 to convert it into number of hours.

Q8: What happen if the voltage has gone up to >10 V?

A8: A high voltage signifies the increase in resistance through the reaction mixture. Two most probable reasons are the evaporation of solvent and electrode passivation of the Zn anode. Add CH_3CN if the solvent level is too low. If solvent level is normal, check if the anode is covered by a layer of black deposit. This can be removed by scratching with a razor blade. Be sure to protect the reaction with N_2 (e.g. with a flow of N_2 under an inverted funnel) if the reaction vessel has to be dissembled.

Q9: Can the electrodes be reused?

A9: Yes. To reuse Zn electrodes, the black passivation layer was removed by sandpaper. Sonication of the sanded electrode in deionized water would further remove any deposit on it. The electrode was then cleaned with acetone and oven-dried at 150 °C, which was cooled down to ambient temperature in a desiccator. Scratch the Zn surface with a razor blade if needed. For Ni foam cathode, black deposition was normally observed after the reaction, which can be removed by submerging in 1 M HCl and sonication. A slight discoloration of the Ni foam does not seem to affect its performance, but we recommend to replace it after two to three runs due to its low cost.

Q10: What are alternative electrodes to Ni foam?

A10: Reticulated vitreous carbon (RVC) or carbon felt (CF) can be used as cathode, albeit the use of Ni foam is optimal.

Q11: Would it affect the yield if electrolysis is continued beyond 6 F/mol?
A11: There was no drop in yield of sulfinate when electrolysis was performed with up to 10 F/mol.

Q12: Is this reaction sensitive to air and water?
A12: The reaction is not particular sensitive to air and water. Glovebox or freeze-pump-thaw technique is not required, but evacuation-N_2 backfill cycle is still needed for the e-sulfinylation step. The electrolyte employed, TBABr, and other components were used without drying, and the addition of 0.5 equiv of water does not affect the yield. Molecular sieve is also not needed for the reaction. However, a lower yield is expected in the presence of excessive water (e.g. when 2 equiv of water added). For the fluorination, it is not sensitive to air or water at all.

Q13: What is the color change of the reaction?
A13: The clear pink solution would turn brown upon the addition of SO_2. Once the electrolysis was started, the color remained brown for the first 2 F/mol, and turned deep red after 2 F/mol and remained deep red until completion.

Q14: What is the side product of this reaction?
A14: The major side reaction was proto-dehalogenation of the aryl iodide/bromide. Occasionally, trace amount of dimer formation was detected.

Q15: Do reduction potentials of substrates correlate to yields?
A15: No. This is evident in the fact that both electron-rich and deficient substrates were converted into sulfonyl fluorides effectively. For instance, while there is a distinctive difference in the reduction potentials of the aryl iodides for **9** and **17** (−2.32 V and −1.95 V, respectively), sulfonyl fluoride **9** (62%) and **17** (64%) were formed in similar yields; meanwhile, the aryl iodides for **7** and **9** have similar reduction potentials (−2.30 V and −2.32 V, respectively), yet gave a distinguishable difference in yields (44% and 62%, respectively). Therefore, there is no clear correlation between substrate reduction potentials and yields, which further suggests that the reaction is mediated by nickel catalyst rather than direct reductive activation of substrate.

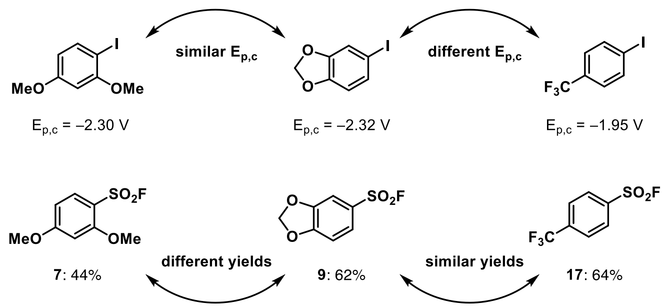

Q16: What factors should be considered when screening additives during optimization of an electrochemical reaction like this one?

A16: While a workable sulfinylation with 25% yield was recorded without any additives, large portion of substrate was proto-dehalogenated. It was speculated that over-potential, and hence the direct cathodic reduction of substrate contributed to the unproductive side reaction. A series of structurally-diverse redox mediators were screened, which have different chemical functionalities and properties, such as reduction potential, Lewis and π-basicity. These factors might affect the dynamic interactions of various reaction components, such as SO_2. On the other hand, TPPA was added in an attempt to mitigate electrode passivation, but was found ineffective. Therefore, an extensive screening of a wide variety of additives was encouraged when optimizing a workable electrochemical reaction like this e-sulfinylation.

Q17: What is the role of NMPI in the reaction?

A17: While direct reduction of aryl halides may take place due to over-potential, the presence of NMPI enables its reduction over the substrate (as an overcharge protector), hence reserving the substrate for the productive reaction with Ni catalyst. Meanwhile the more negative potential of NMPI (vs Ni catalyst) suggests that the reduced $NMPI^{\bullet-}$ is more reducing than the Ni(I) complex, hence is capable to reduce the Ni(II) catalyst into Ni(I), which plays a role as a reductive mediator.

Q18: What is the X in sulfinate $ArSO_2X$?

A18: While aryl sulfinates were not isolated, X can be any cation that is present in the reaction mixture, such as Bu_4N^+, Zn^{2+} or $Ni^{+/2+}$, and there is no evidence of what cation would sulfinate bound to in the homogeneous solution.

Q19: Can TBAF/NCS be used for fluorination when aryl bromides were employed?

A19: Yes, both NFSI and TBAF/NCS conditions worked with aryl bromides, with NFSI gave marginally higher yields (e.g. 60% vs 56% for sulfonyl fluoride **27**).

Q20: What is the mechanism for the fluorination with TBAF/NCS?

A20: Sulfinates, which are nucleophilic at the S atom, are thought to attack on the Cl of NCS to generate sulfonyl chloride in situ. The addition of TBAF prior to NCS ensures the reactive sulfonyl chloride can be converted to the more stable sulfonyl fluoride through a rapid Cl/F exchange.

16. References

[1] A. T. Davies, J. M. Curto, S. W. Bagley, M. C. Willis, *Chem. Sci.* **2017**, *8*, 1233-1237.

[2] A. L. Tribby, I. Rodriguez, S. Shariffudin, N. D. Ball, *J. Org. Chem.* **2017**, *82*, 2294-2299.

[3] A. Shavnya, S. B. Coffey, A. C. Smith, V. Mascitti, *Org. Lett.* **2013**, *15*, 6226-6229.

[4] P. K. T. Lo, Y. Chen, M. C. Willis, *ACS Catalysis* **2019**, *9*, 10668-10673.

[5] H. J. Zhang, L. Chen, M. S. Oderinde, J. T. Edwards, Y. Kawamata, P. S. Baran, *Angew. Chem. Int. Ed.* **2021**, *60*, 20700-20705.

[6] E. Duan, P. Zhang, K. Yang, W. Liang, M. Yu, S. Wang, J. Niu, *RSC Adv.* **2016**, *6*, 55401-55405.

[7] C. Lee, N. D. Ball, G. M. Sammis, *Chem. Commun.* **2019**, *55*, 14753-14756.

[8] X. Song, Y. He, B. Wang, S. Peng, X. Pan, M. Wei, Q. Liu, H.-L. Qin, H. Tang, *Tetrahedron* **2022**, *108*, 132657.

[9] Q. Zheng, J. L. Woehl, S. Kitamura, D. Santos-Martins, C. J. Smedley, G. Li, S. Forli, J. E. Moses, D. W. Wolan, K. B. Sharpless, *Proc. Natl. Acad. Sci.* **2019**, *116*, 18808-18814.

[10] L. Tang, Y. Yang, L. Wen, X. Yang, Z. Wang, *Green Chemistry* **2016**, *18*, 1224-1228.

[11] T. S.-B. Lou, M. C. Willis, *Tetrahedron* **2020**, *76*, 130782.

[12] H. Mukherjee, J. Debreczeni, J. Breed, S. Tentarelli, B. Aquila, J. E. Dowling, A. Whitty, N. P. Grimster, *Org. Biomol. Chem.* **2017**, *15*, 9685-9695.

[13] S. Liu, Y. Huang, X.-H. Xu, F.-L. Qing, *J. Fluor. Chem.* **2020**, *240*, 109653.

[14] D. Louvel, A. Chelagha, J. Rouillon, P.-A. Payard, L. Khrouz, C. Monnereau, A. Tlili, *Chem. Eur. J.* **2021**, *27*, 8704-8708.

[15] Q. Lin, Z. Ma, C. Zheng, X.-J. Hu, Y. Guo, Q.-Y. Chen, C. Liu, *Chin. J. Chem .* **2020**, *38*, 1107-1110.

[16] W. Devine, J. L. Woodring, U. Swaminathan, E. Amata, G. Patel, J. Erath, N. E. Roncal, P. J. Lee, S. E. Leed, A. Rodriguez, K. Mensa-Wilmot, R. J. Sciotti, M. P. Pollastri, *J. Med. Chem.* **2015**, *58*, 5522-5537.

17. NMR Spectra

2-(Methylthio)benzenesulfonyl fluoride (3)

^{1}H-NMR Spectrum:

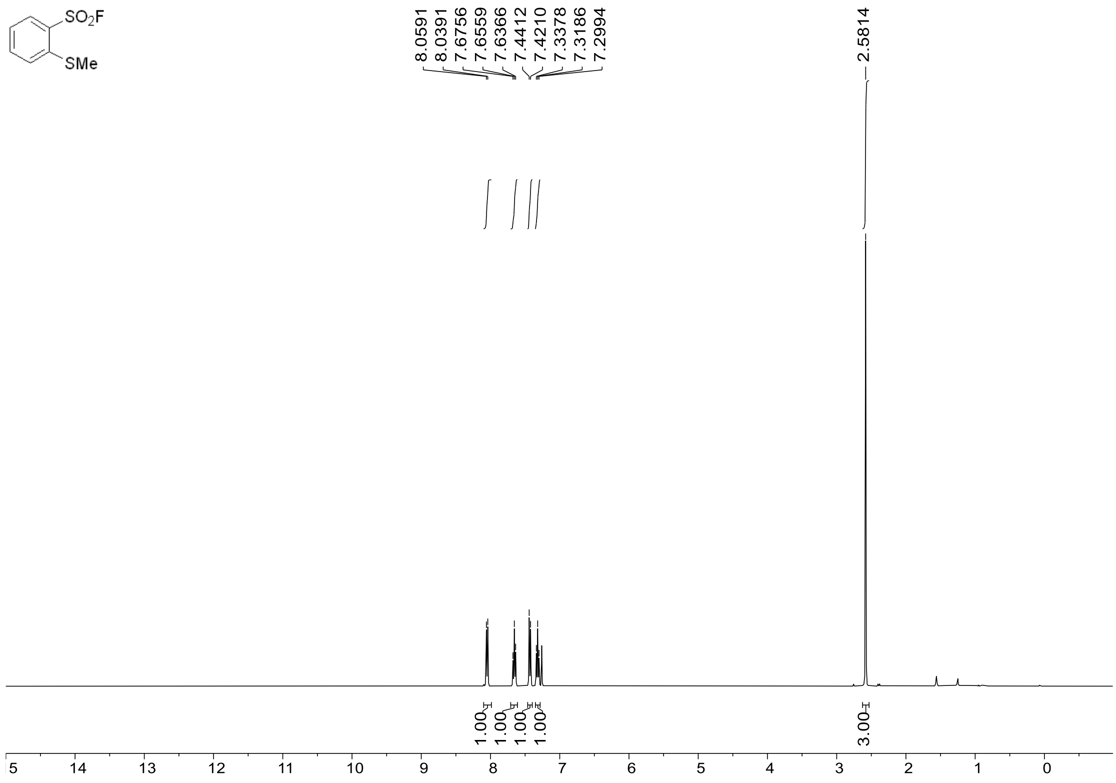

^{13}C-NMR Spectrum:

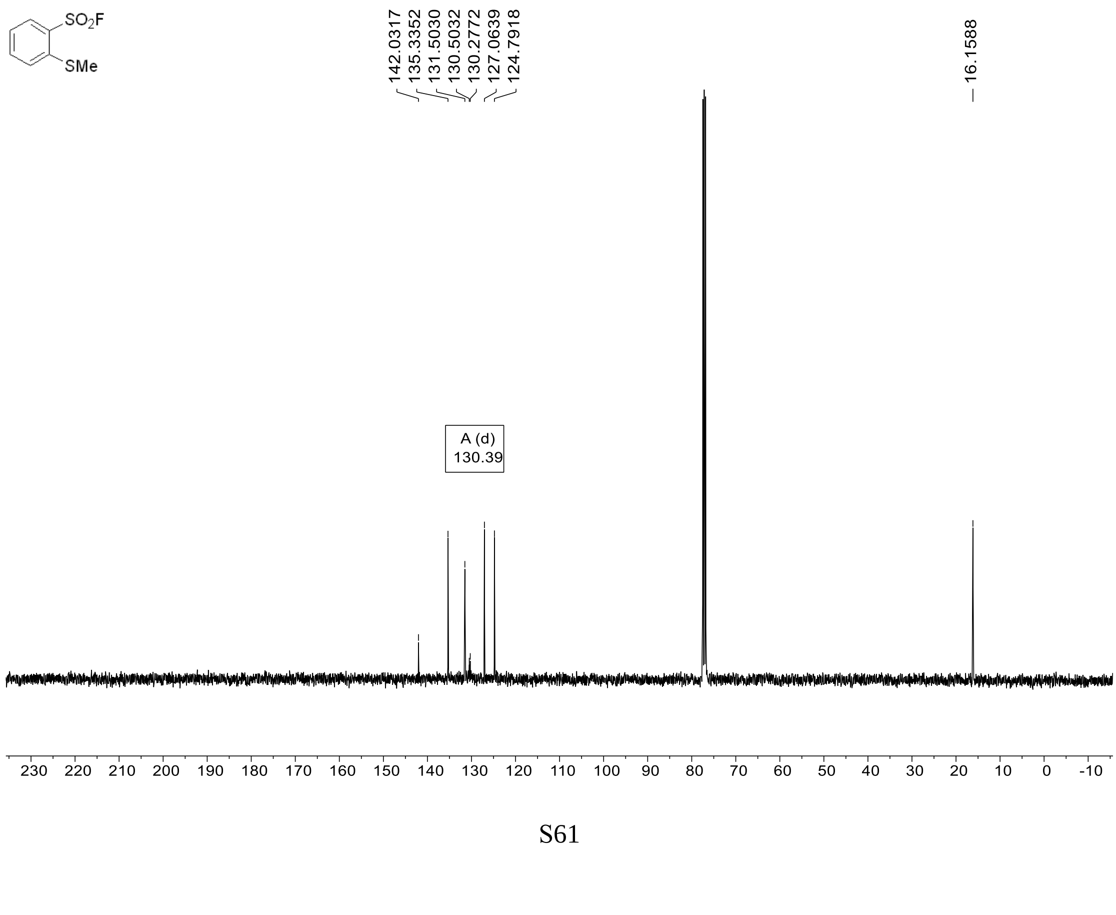

^{19}F-NMR Spectrum:

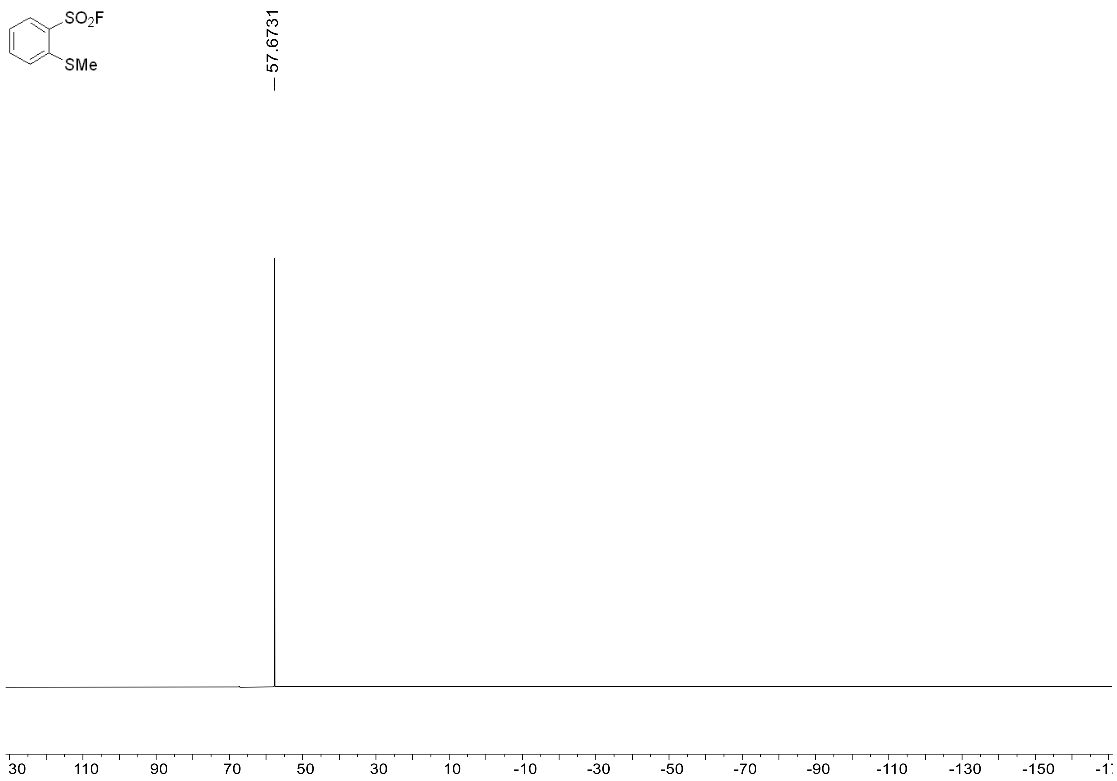

3,5-Dimethylbenzenesulfonyl fluoride (4)

^{1}H-NMR Spectrum:

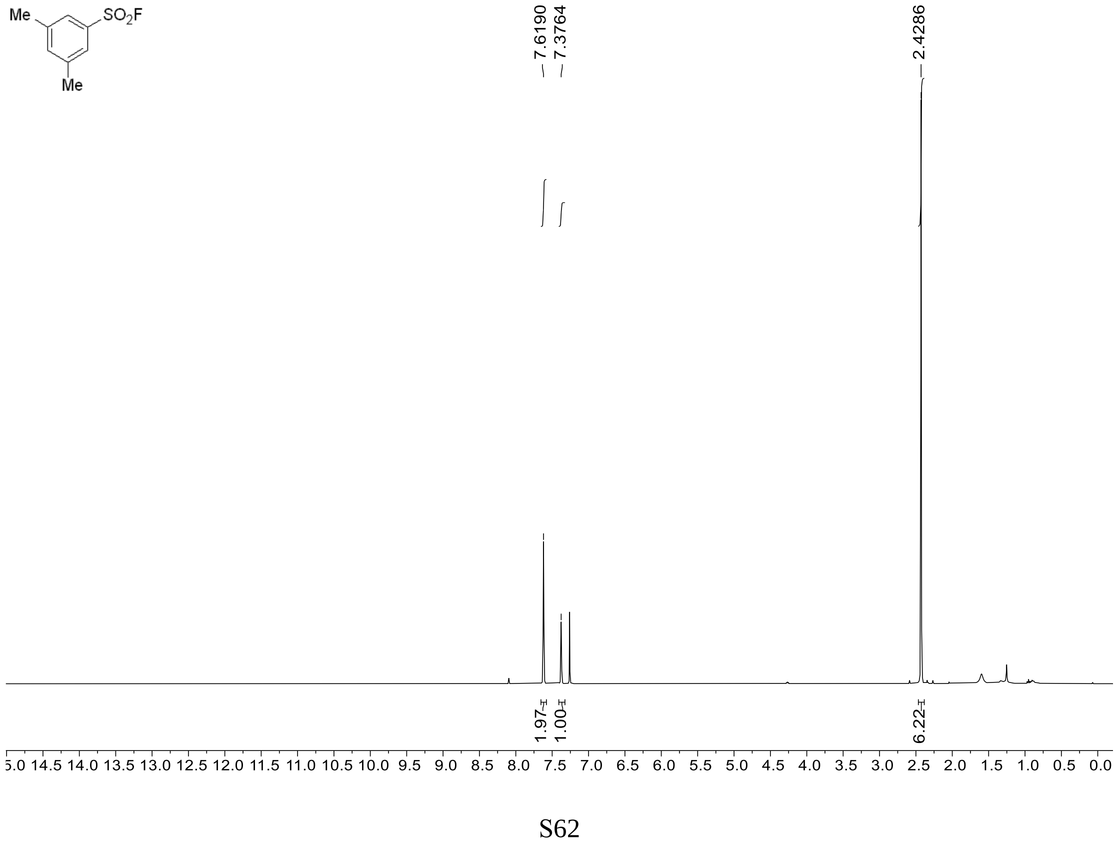

^{13}C-NMR Spectrum:

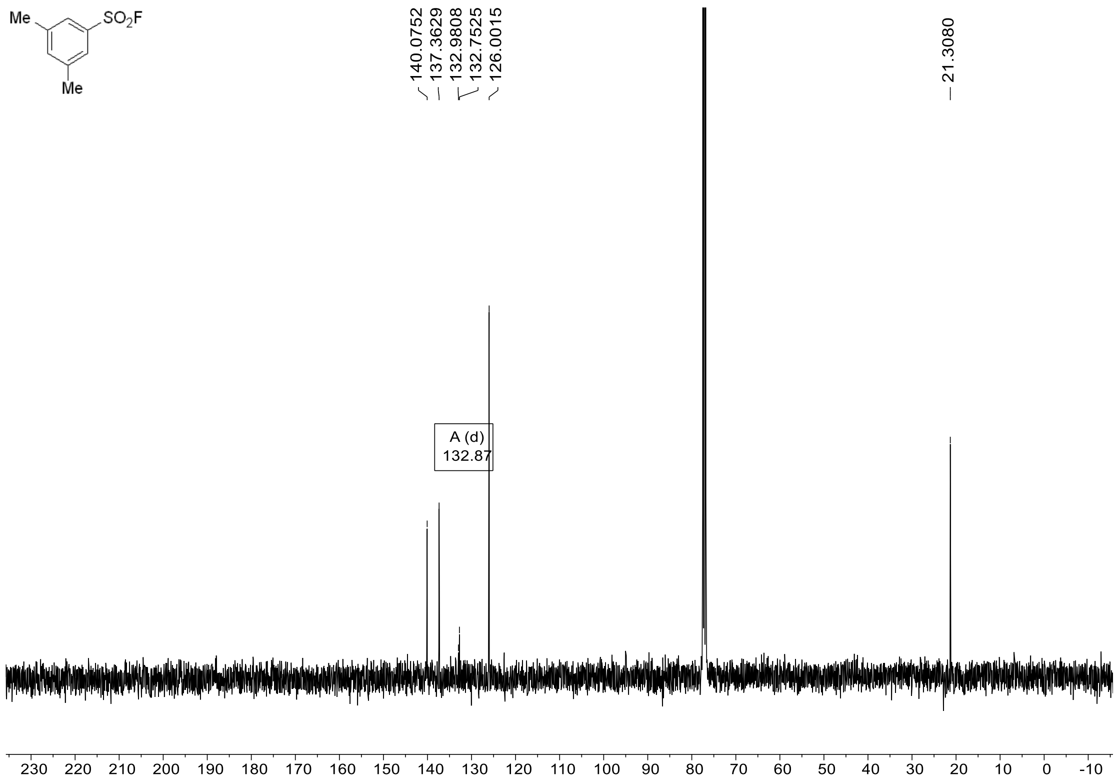

^{19}F-NMR Spectrum:

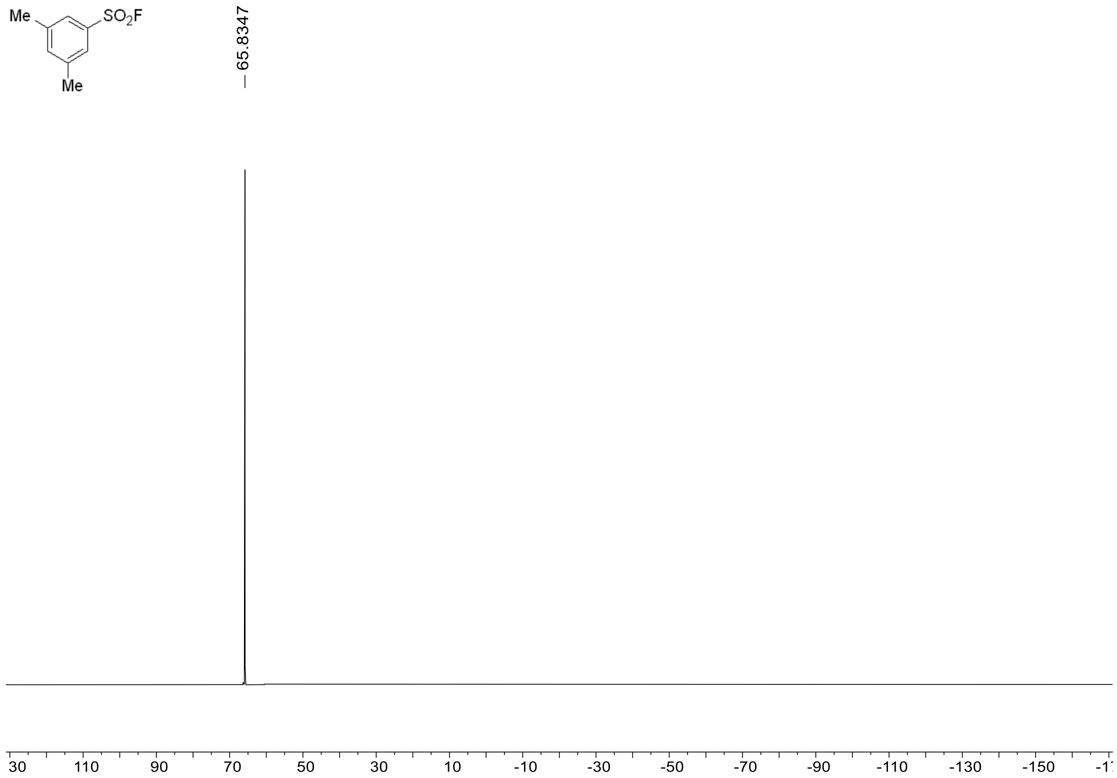

2,4,6-Trimethylbenzenesulfonyl fluoride (5)

^{1}H-NMR Spectrum:

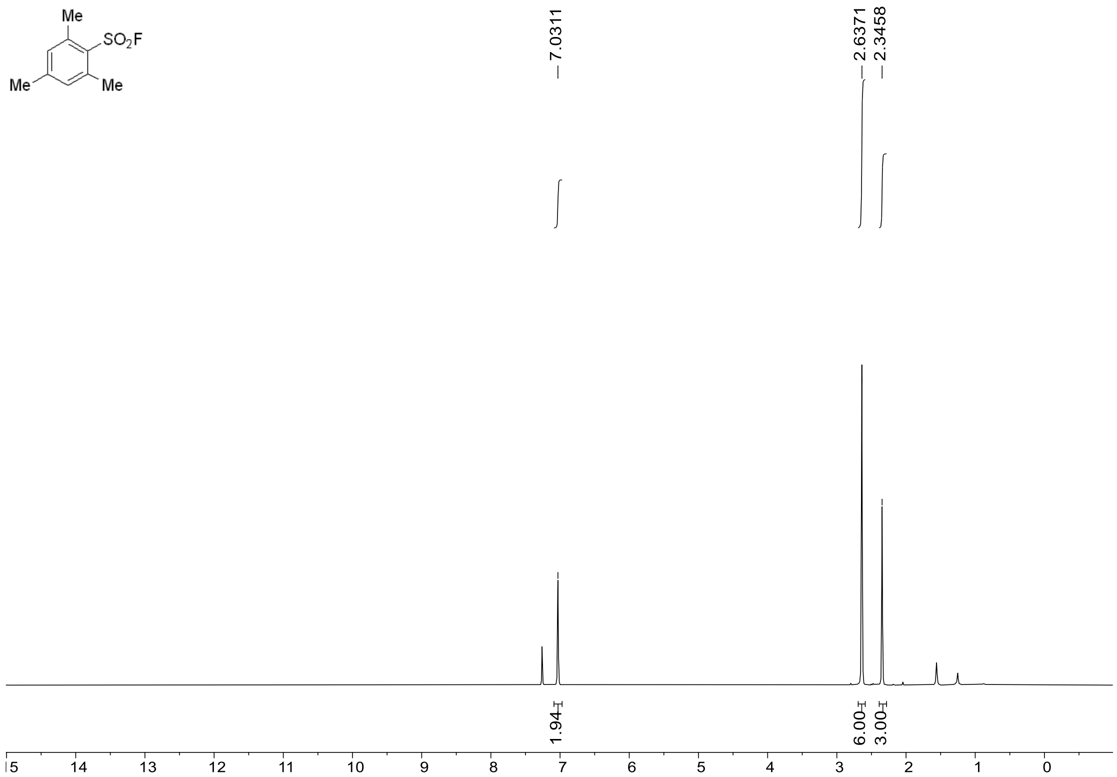

^{13}C-NMR Spectrum:

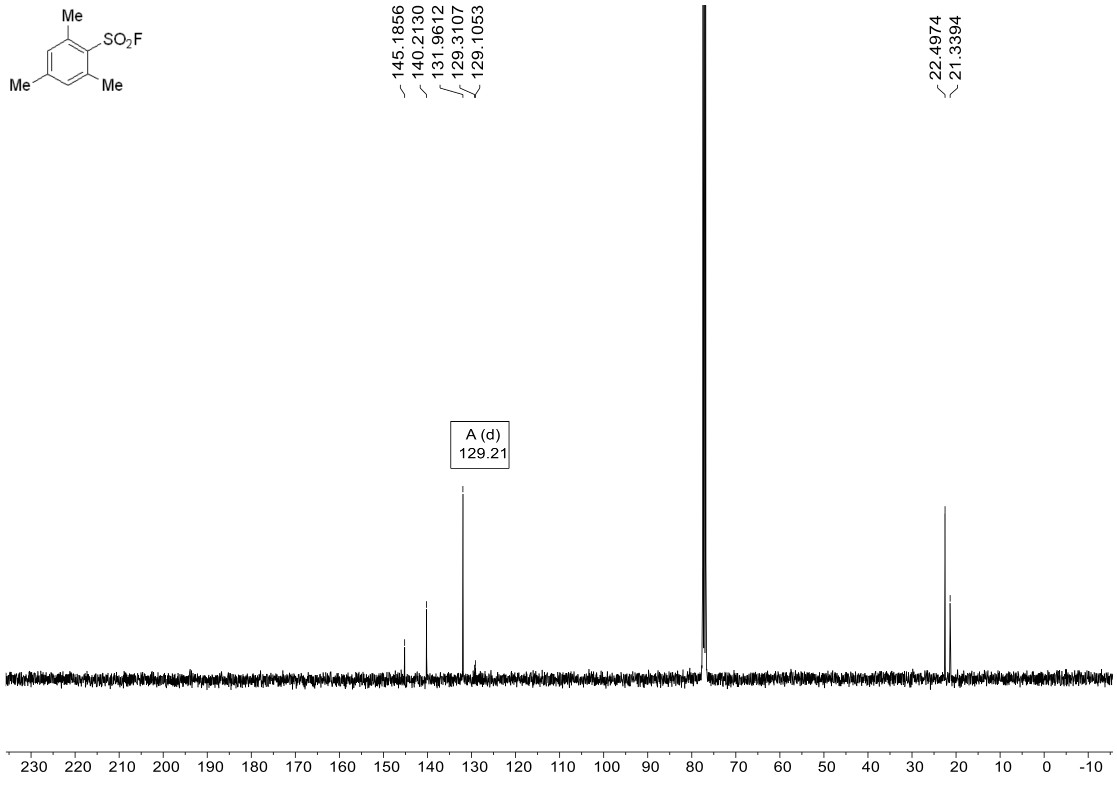

^{19}F-NMR Spectrum:

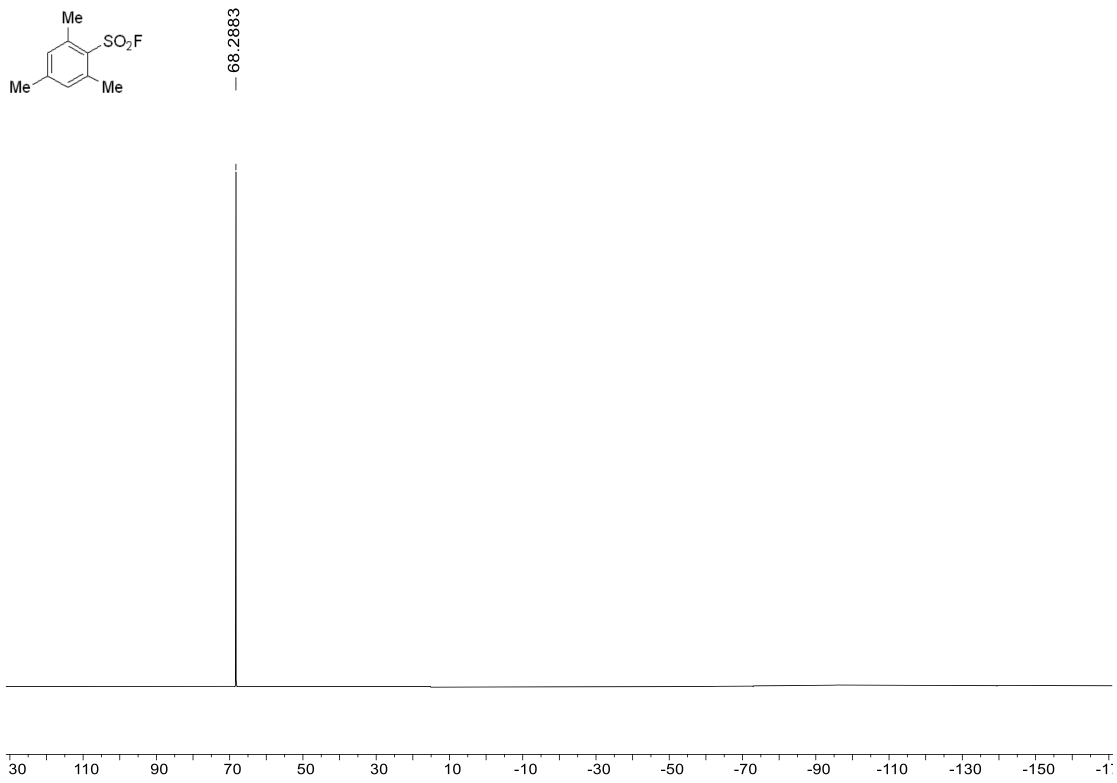

4-Methoxybenzenesulfonyl fluoride (6)

^{1}H-NMR Spectrum:

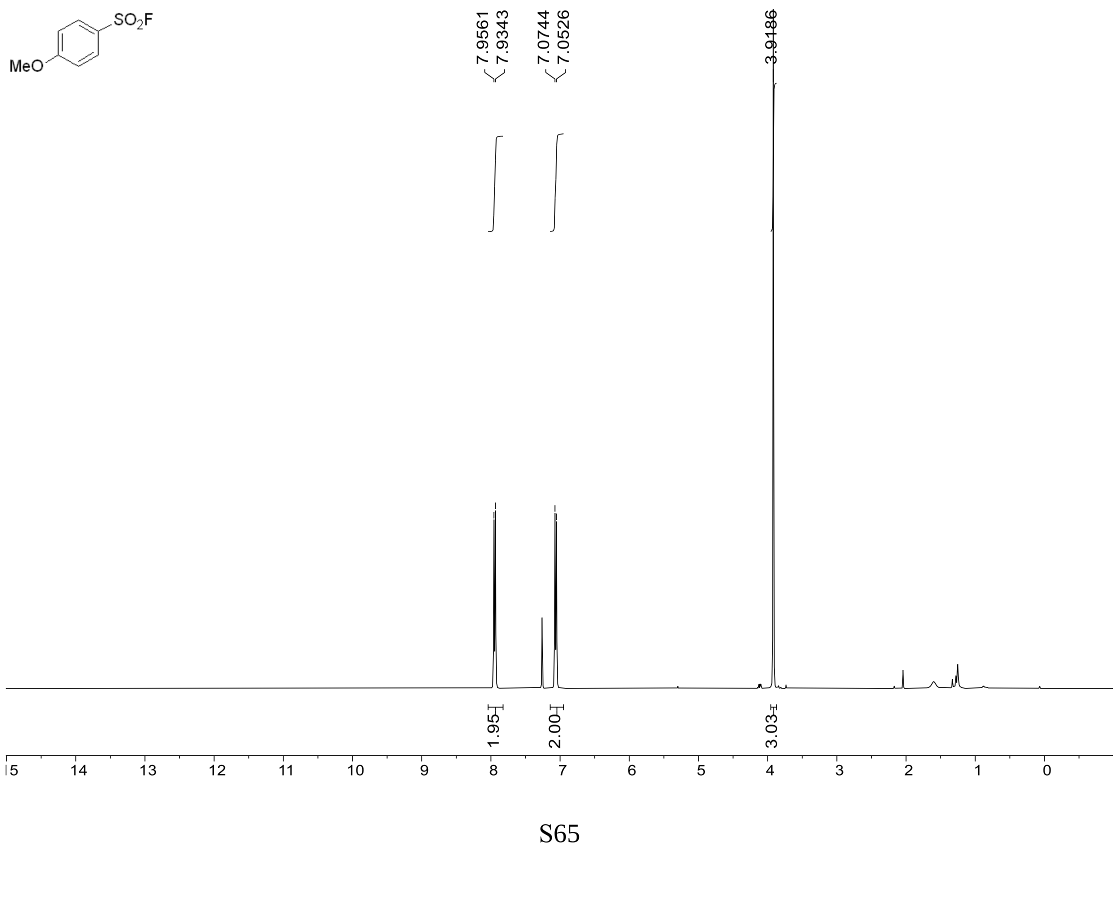

^{13}C-NMR Spectrum:

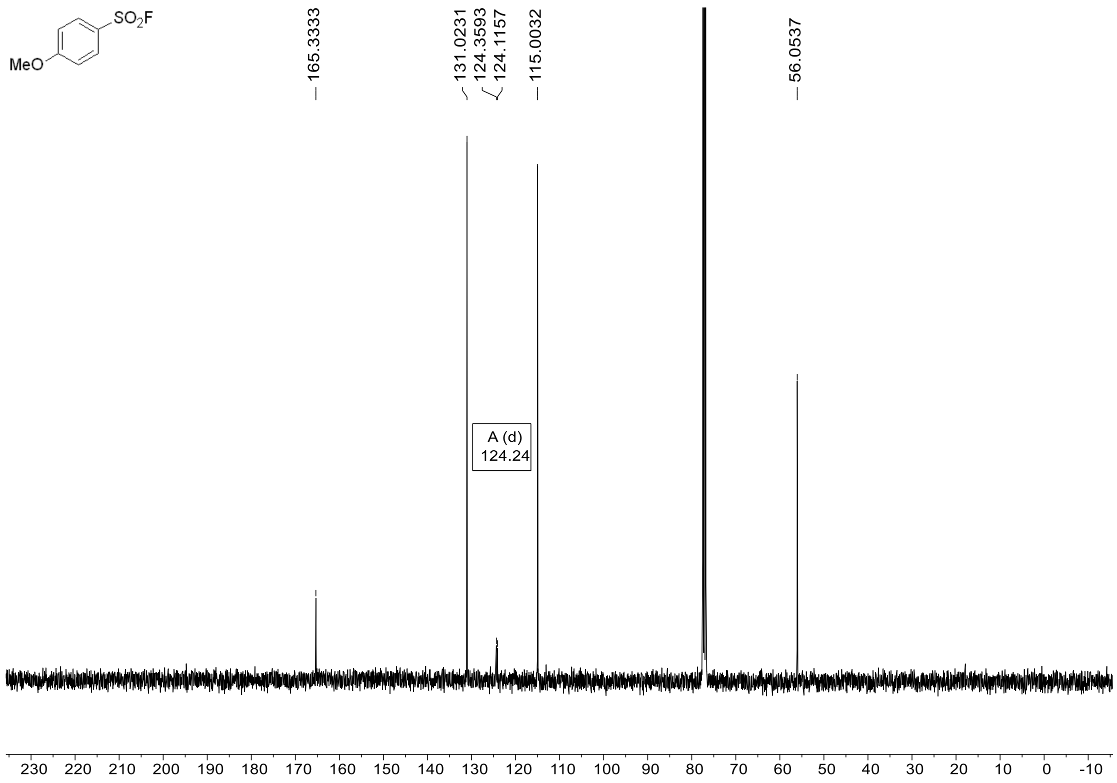

^{19}F-NMR Spectrum:

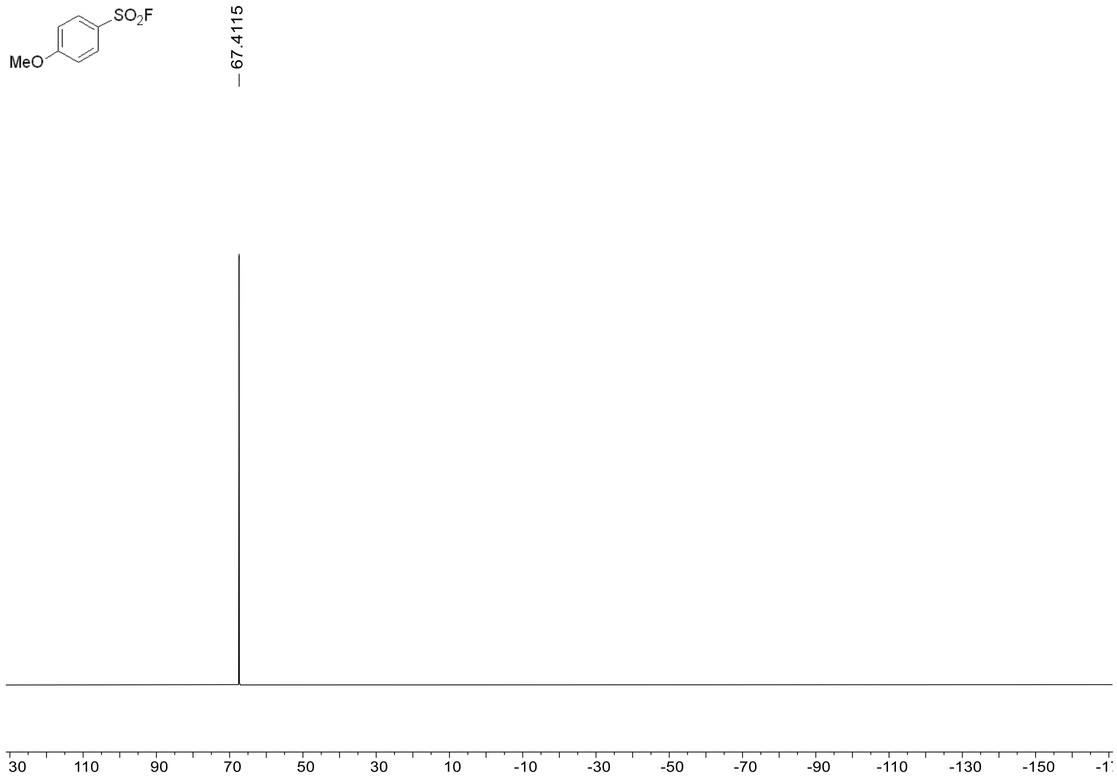

2,4-Dimethoxybenzenesulfonyl fluoride (7)

^{1}H-NMR Spectrum:

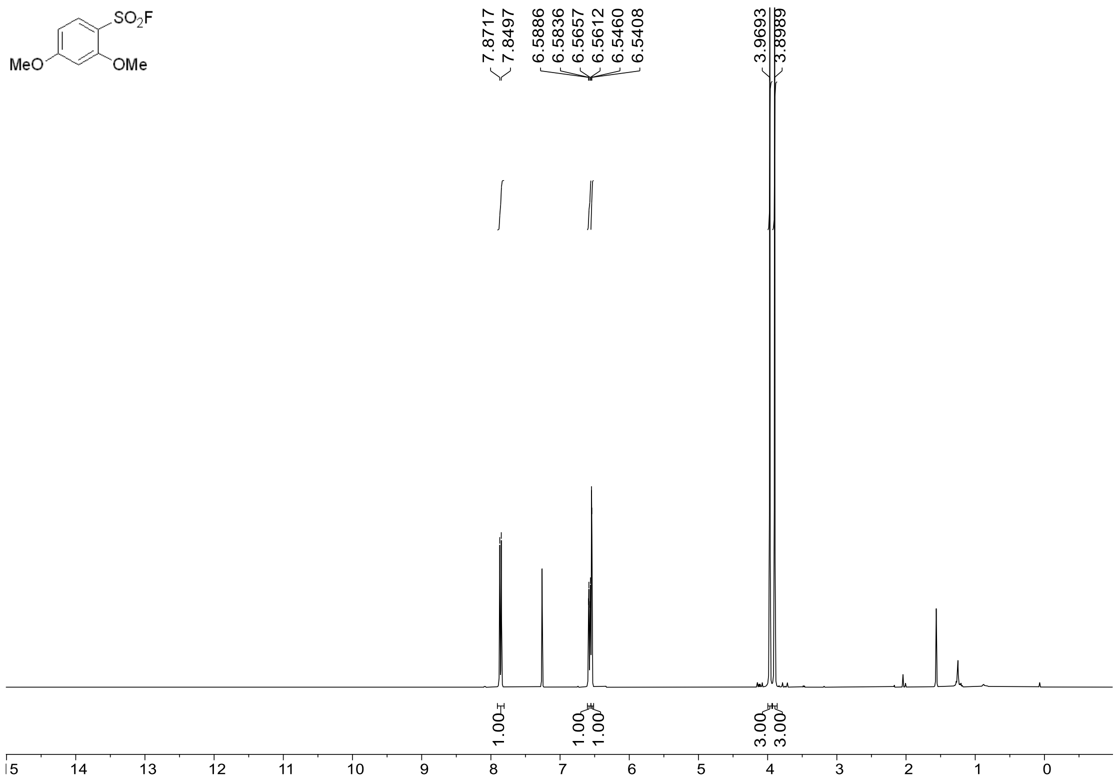

^{13}C-NMR Spectrum:

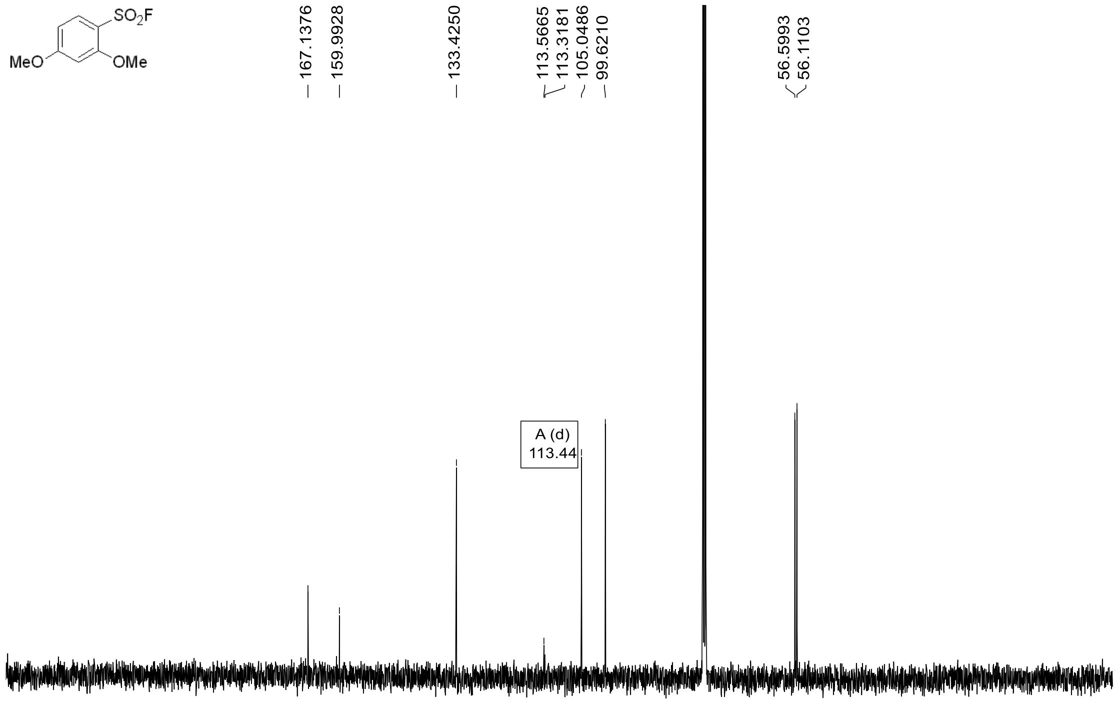

^{19}F-NMR Spectrum:

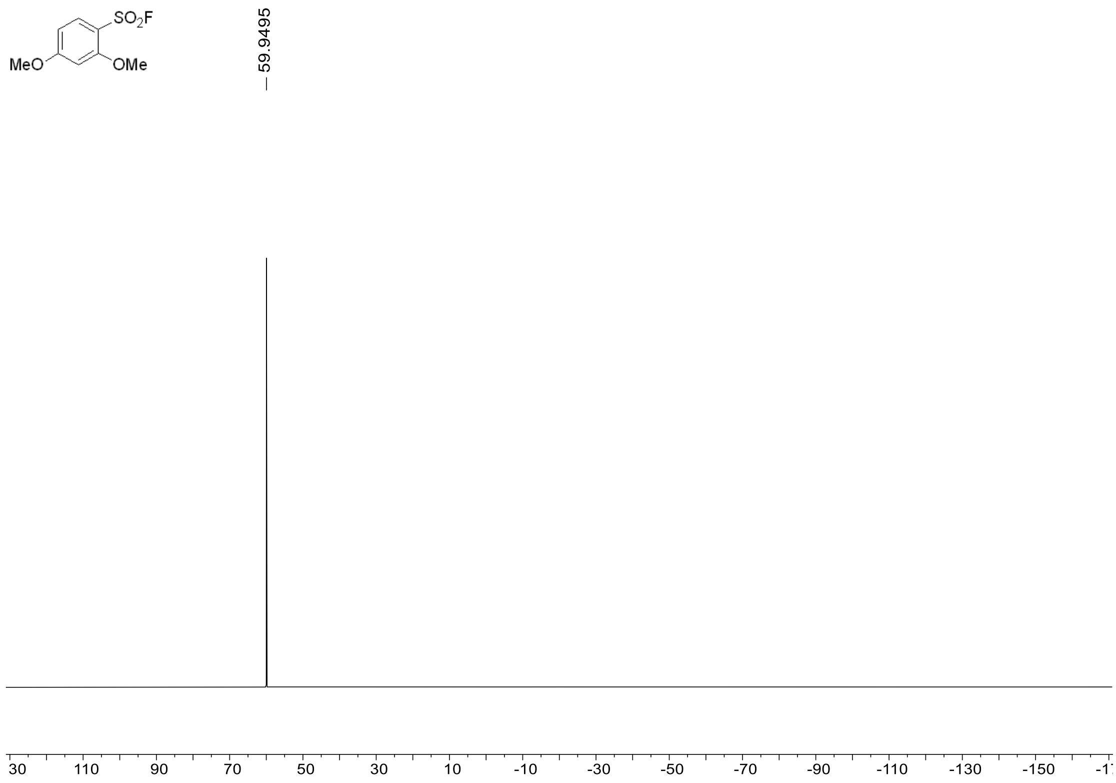

2-Methoxybenzenesulfonyl fluoride (8)

^{1}H-NMR Spectrum:

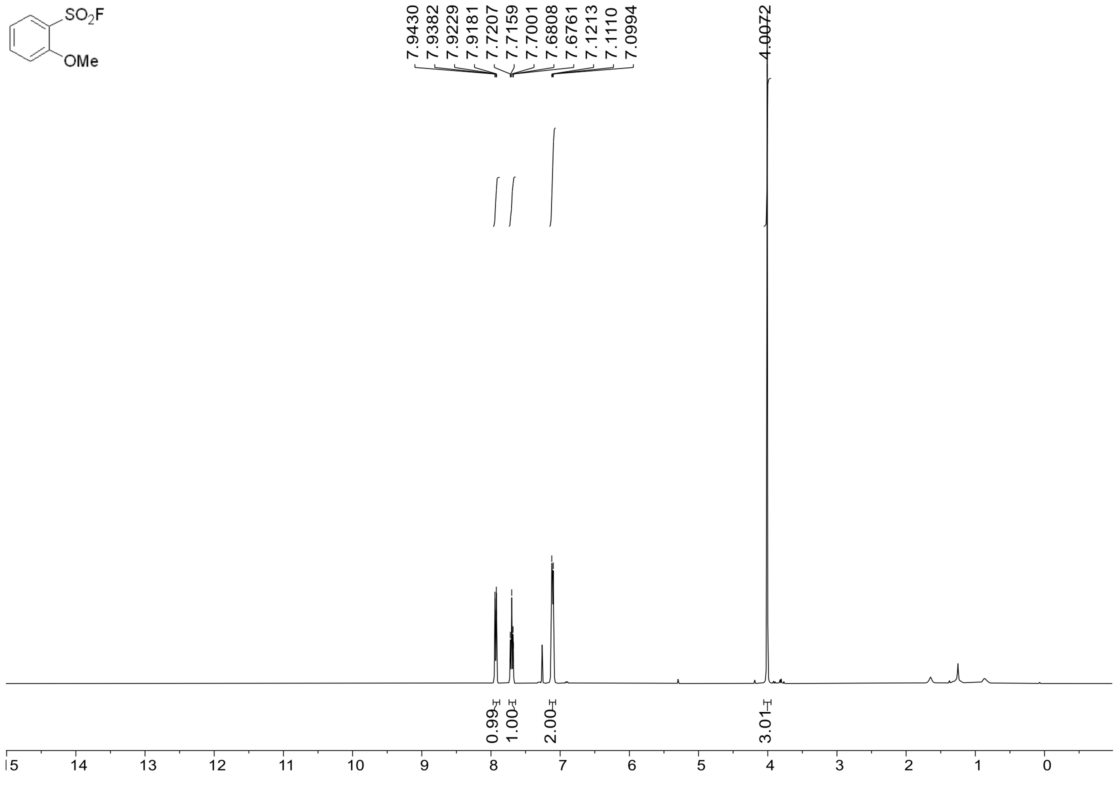

^{13}C-NMR Spectrum:

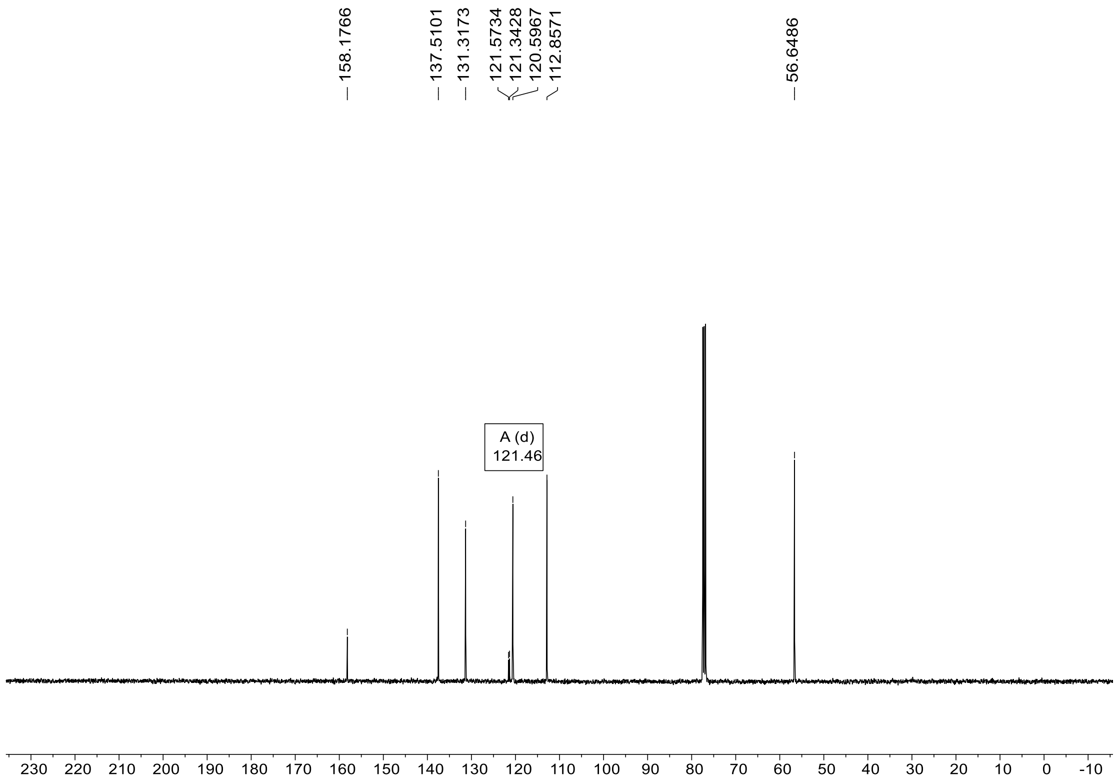

^{19}F-NMR Spectrum:

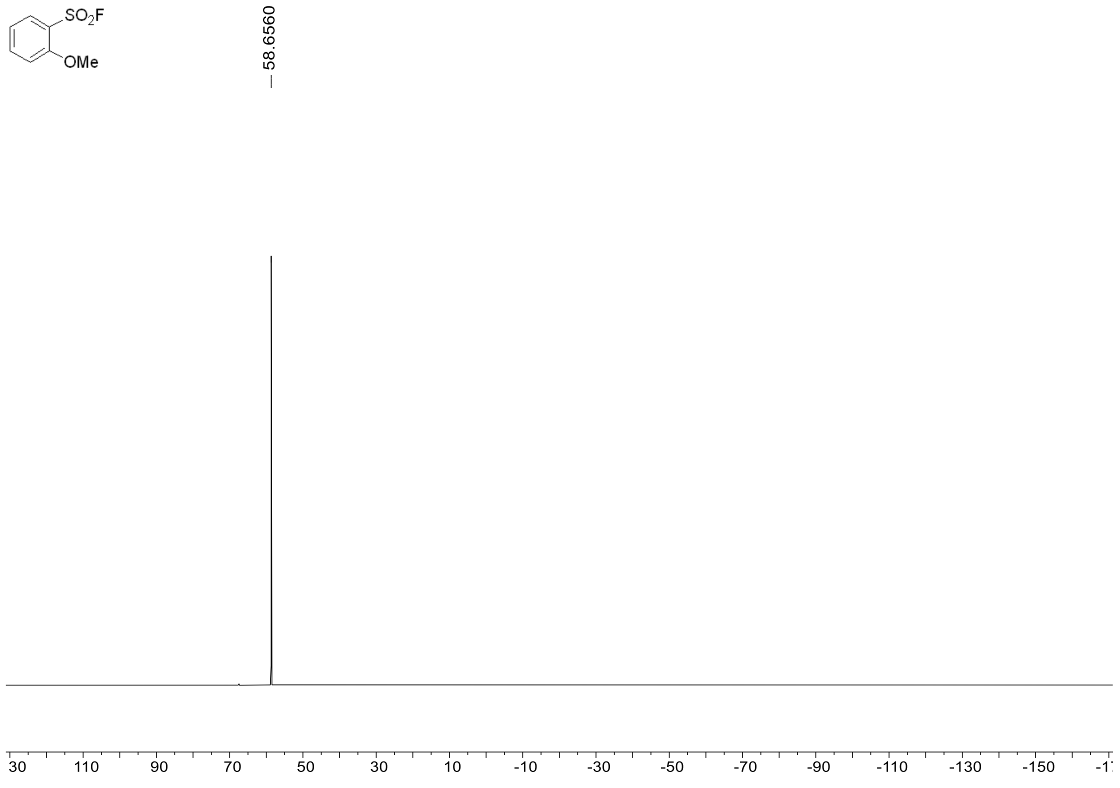

Benzo[d][1,3]dioxole-5-sulfonyl fluoride (9)

^{1}H-NMR Spectrum:

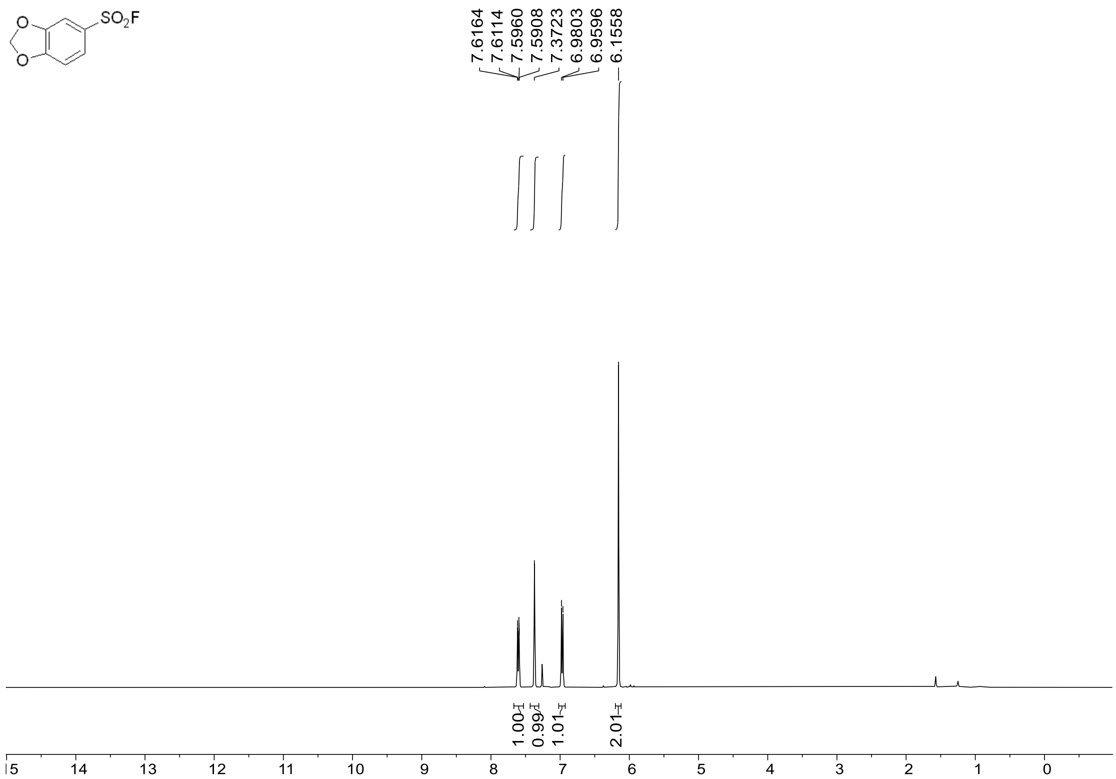

^{13}C-NMR Spectrum:

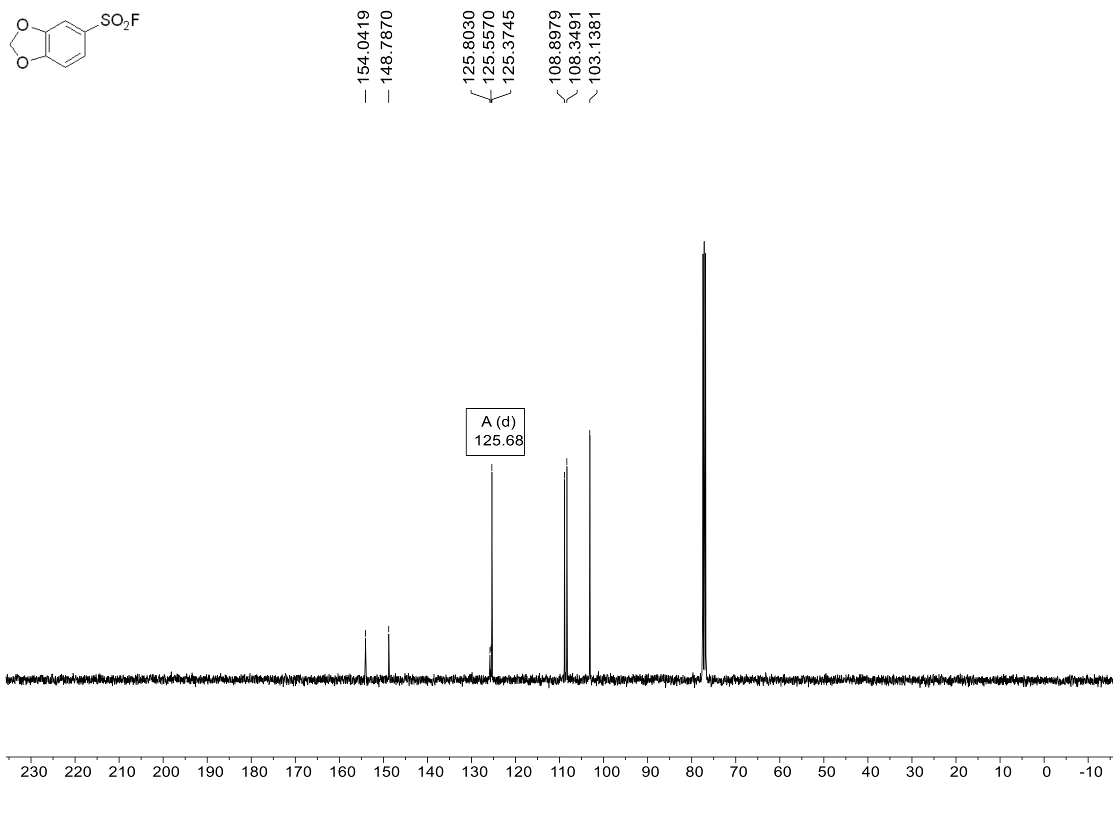

^{19}F-NMR Spectrum:

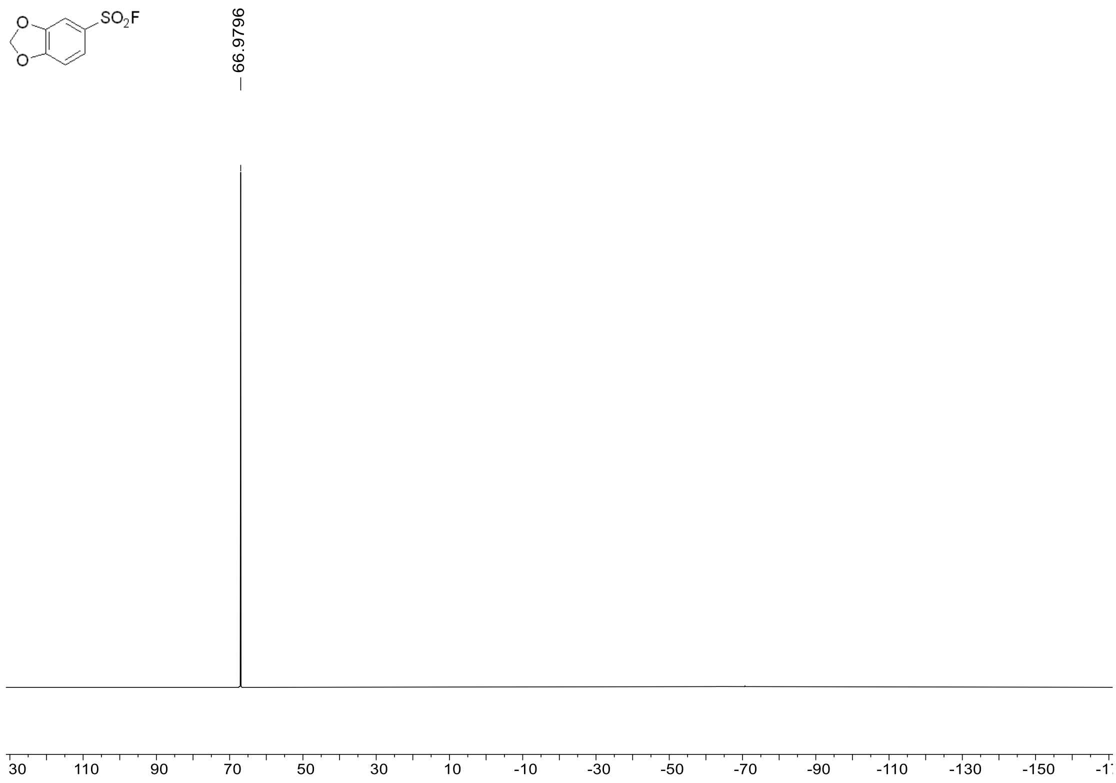

2-Aminobenzenesulfonyl fluoride (10)

^{1}H-NMR Spectrum:

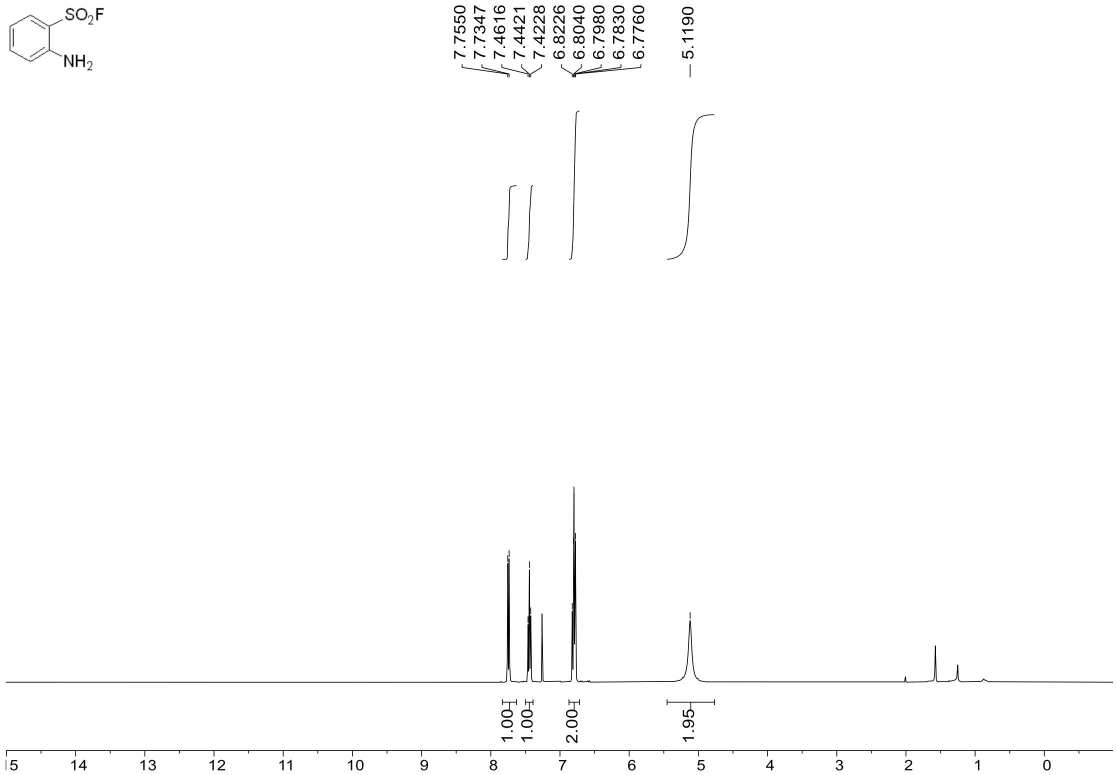

^{13}C-NMR Spectrum:

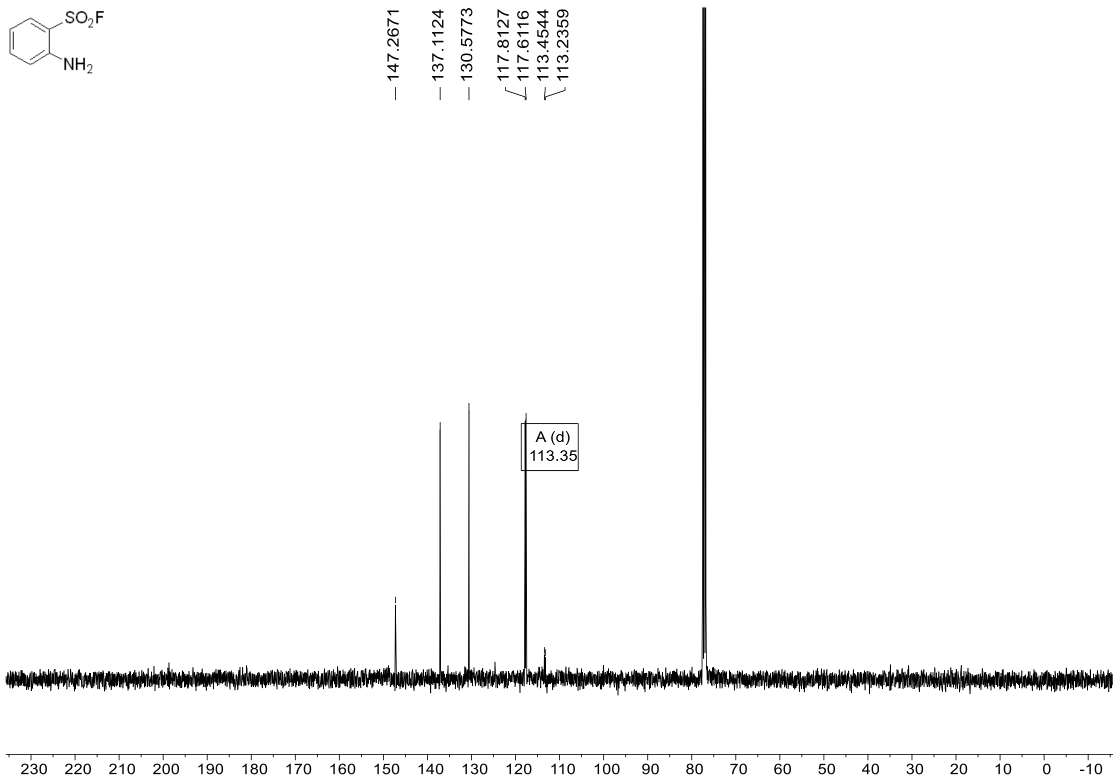

^{19}F-NMR Spectrum:

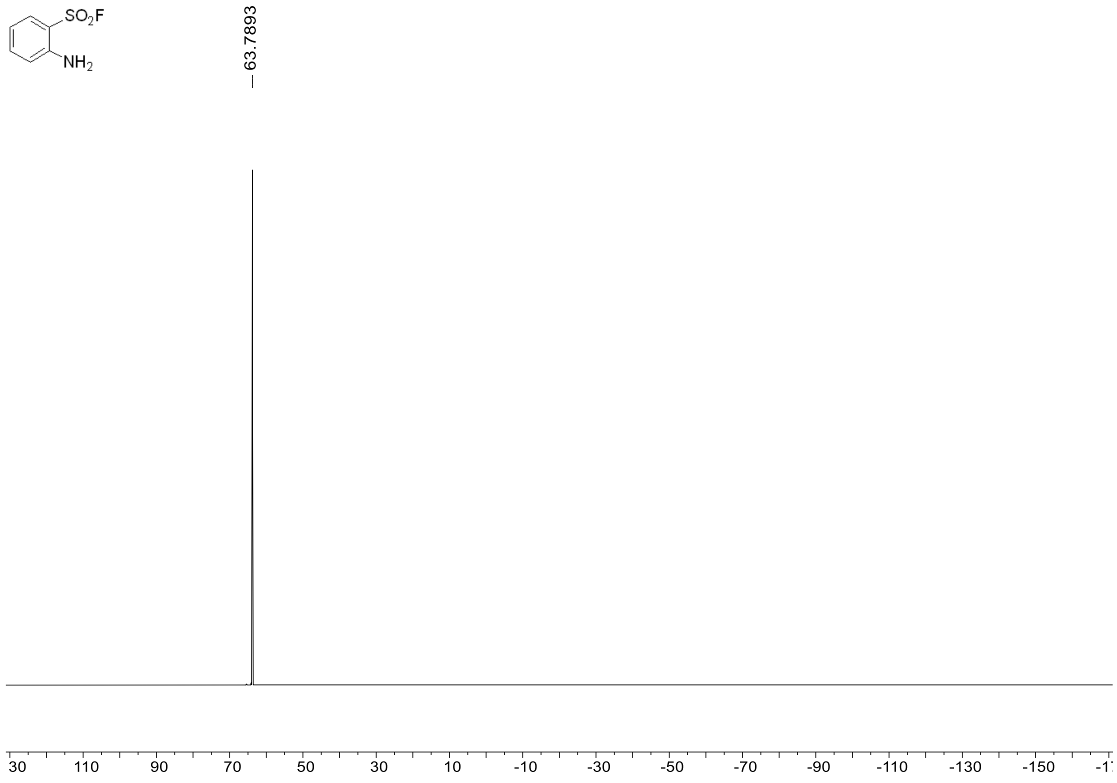

***tert*-Butyl (2-(fluorosulfonyl)phenyl)carbamate (11)**

^{1}H-NMR Spectrum:

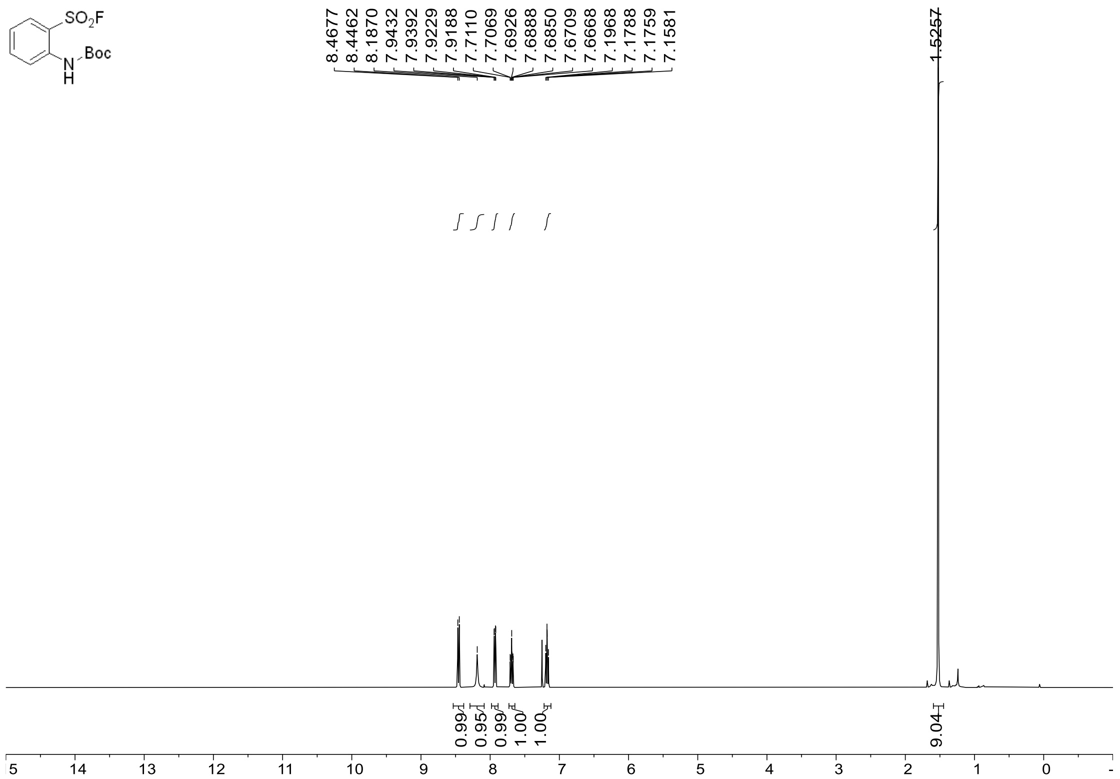

^{13}C-NMR Spectrum:

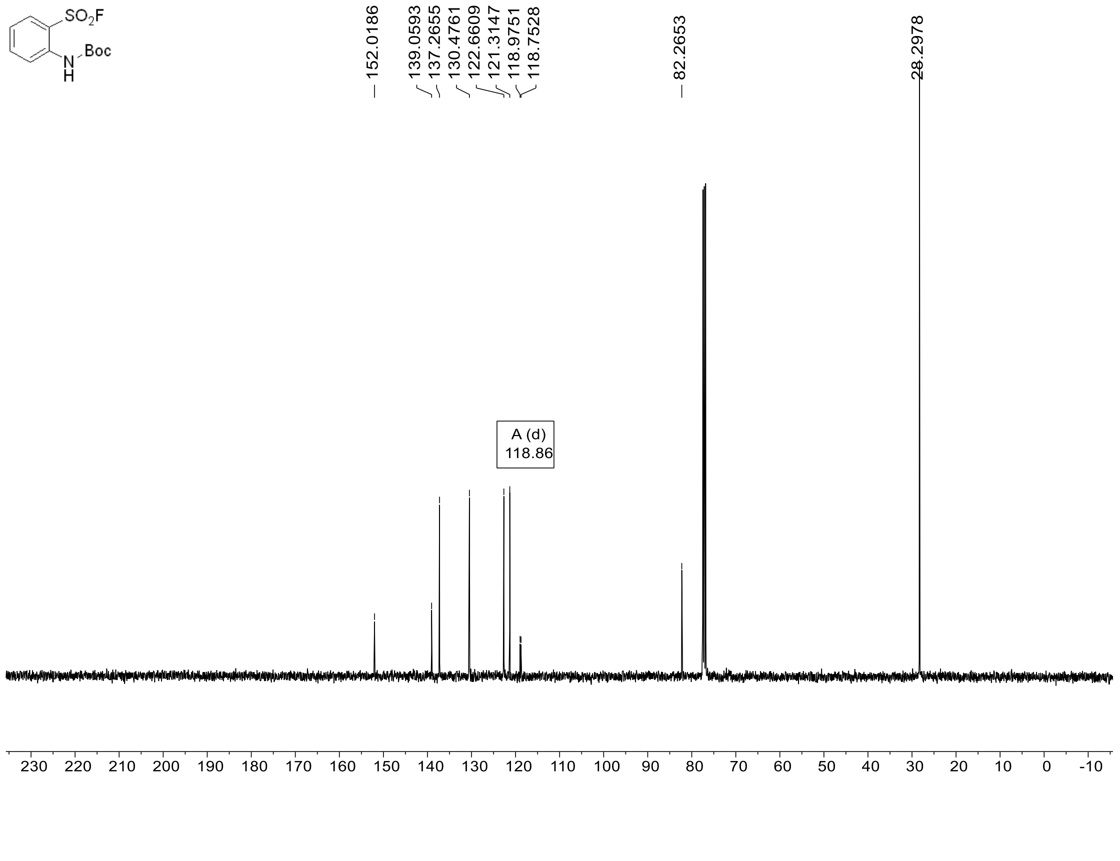

^{19}F-NMR Spectrum:

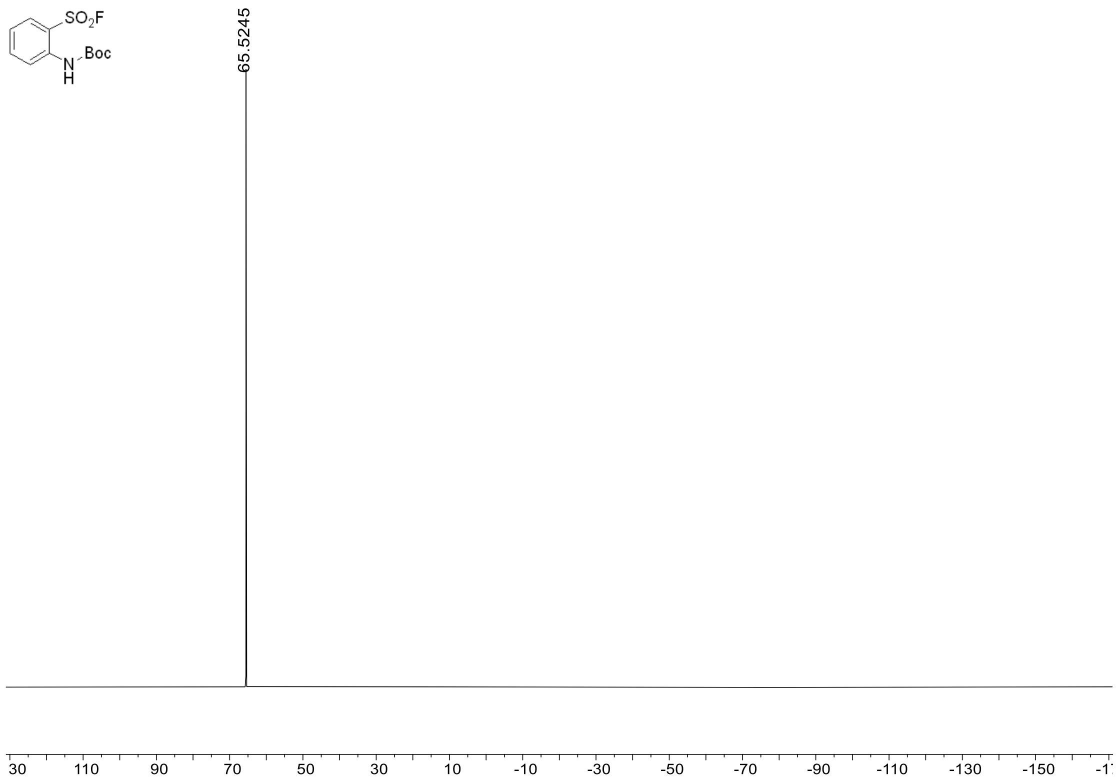

[1,1'-Biphenyl]-2-sulfonyl fluoride (12)

^{1}H-NMR Spectrum:

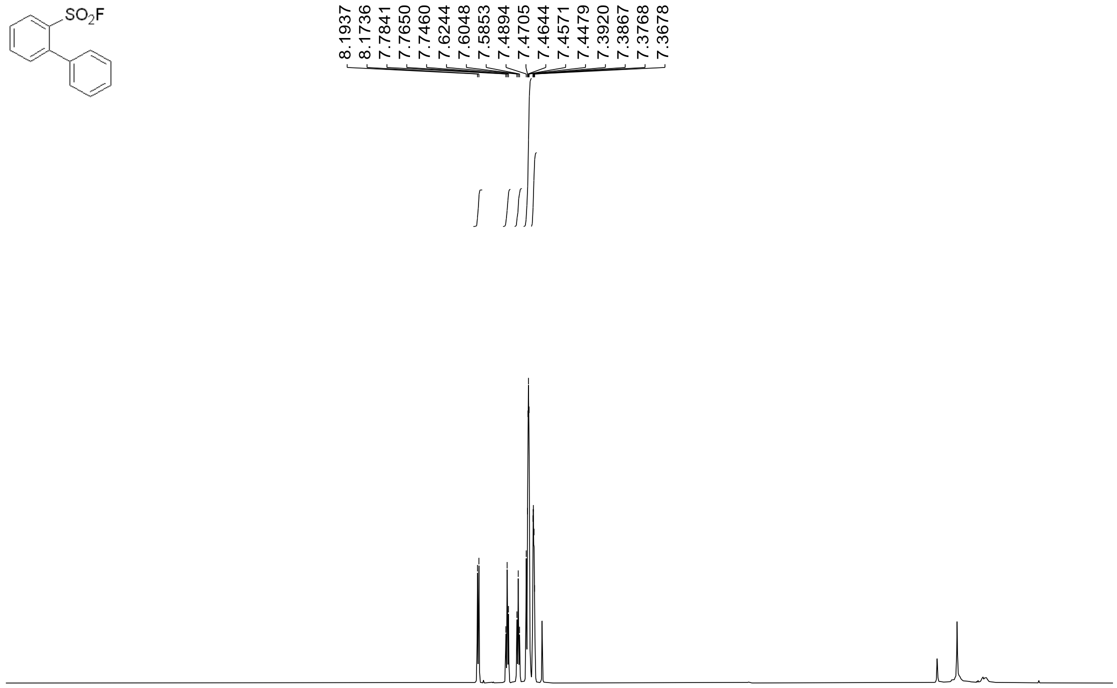

^{13}C-NMR Spectrum:

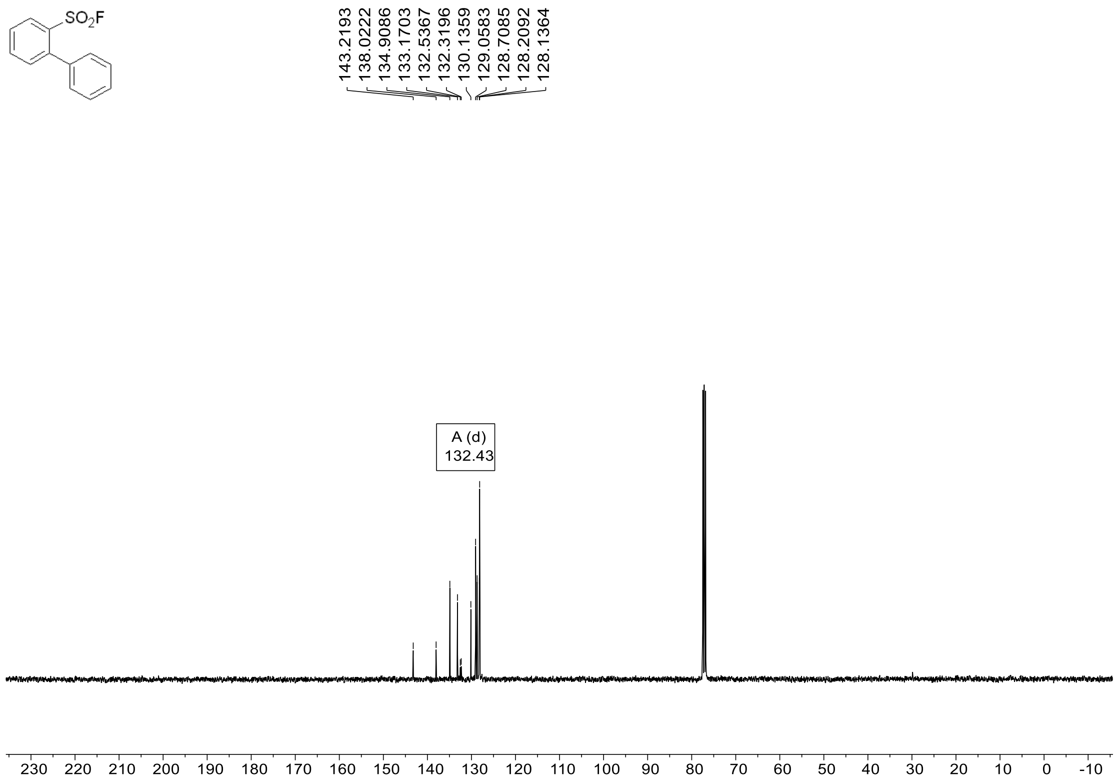

^{19}F-NMR Spectrum:

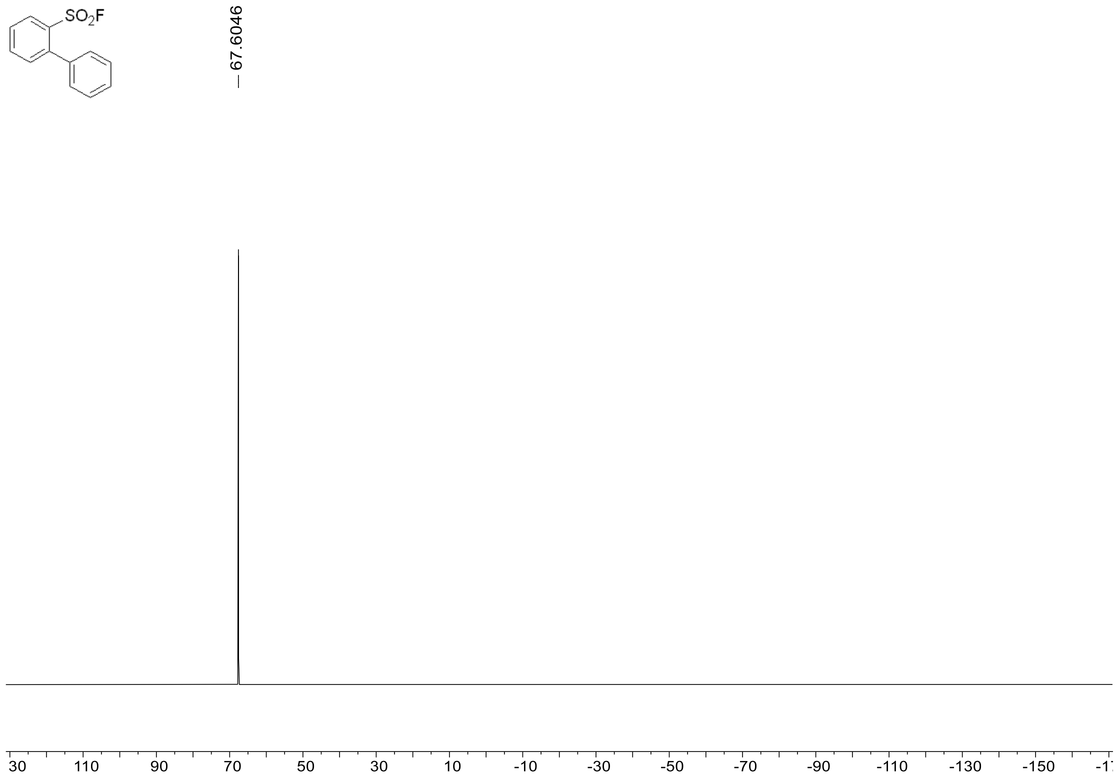

4-Fluorobenzenesulfonyl fluoride (13)

^{1}H-NMR Spectrum:

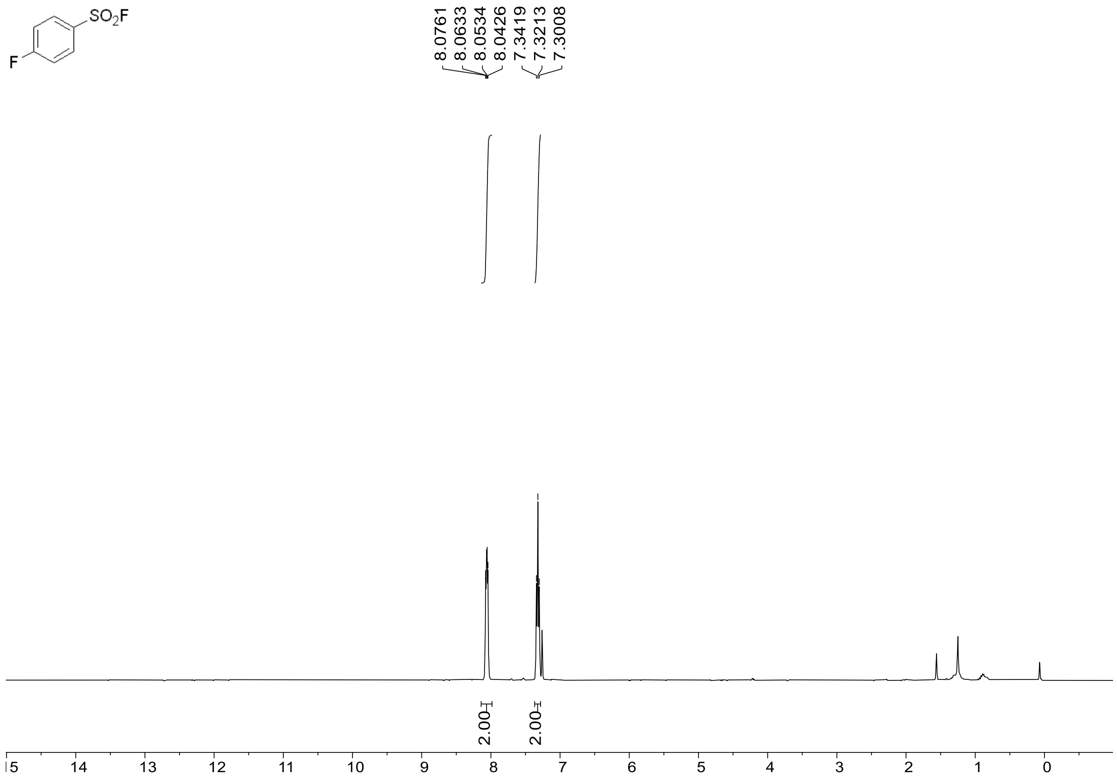

^{13}C-NMR Spectrum:

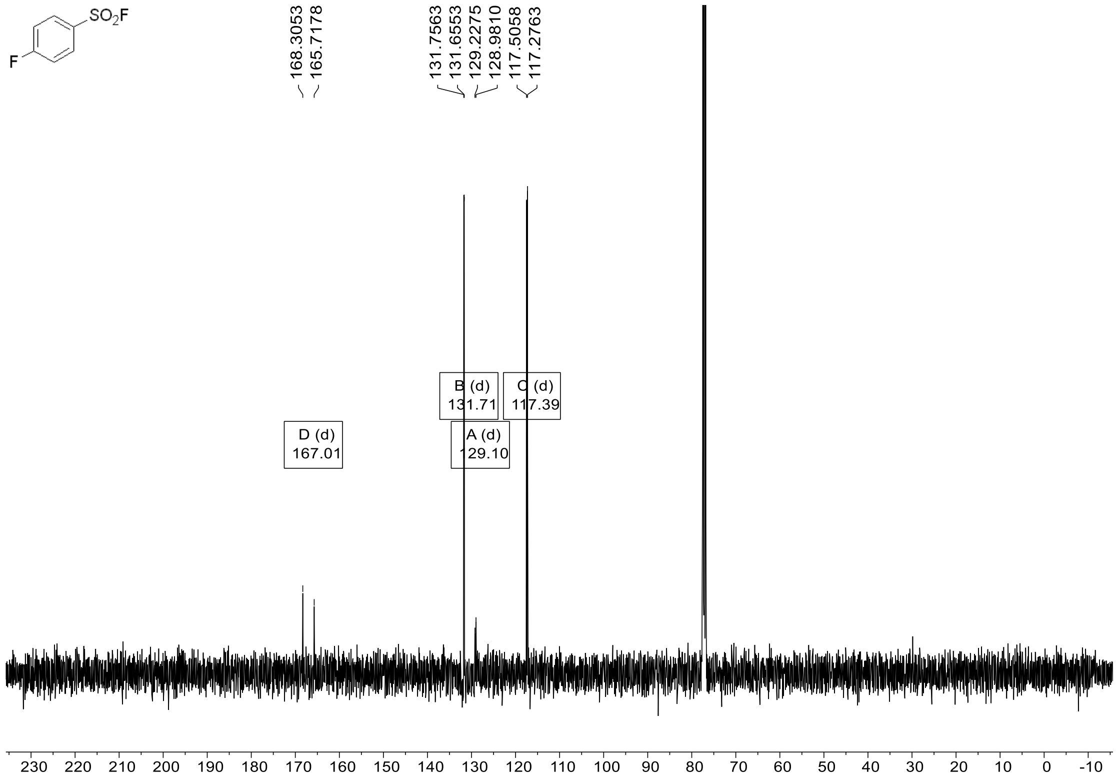

^{19}F-NMR Spectrum:

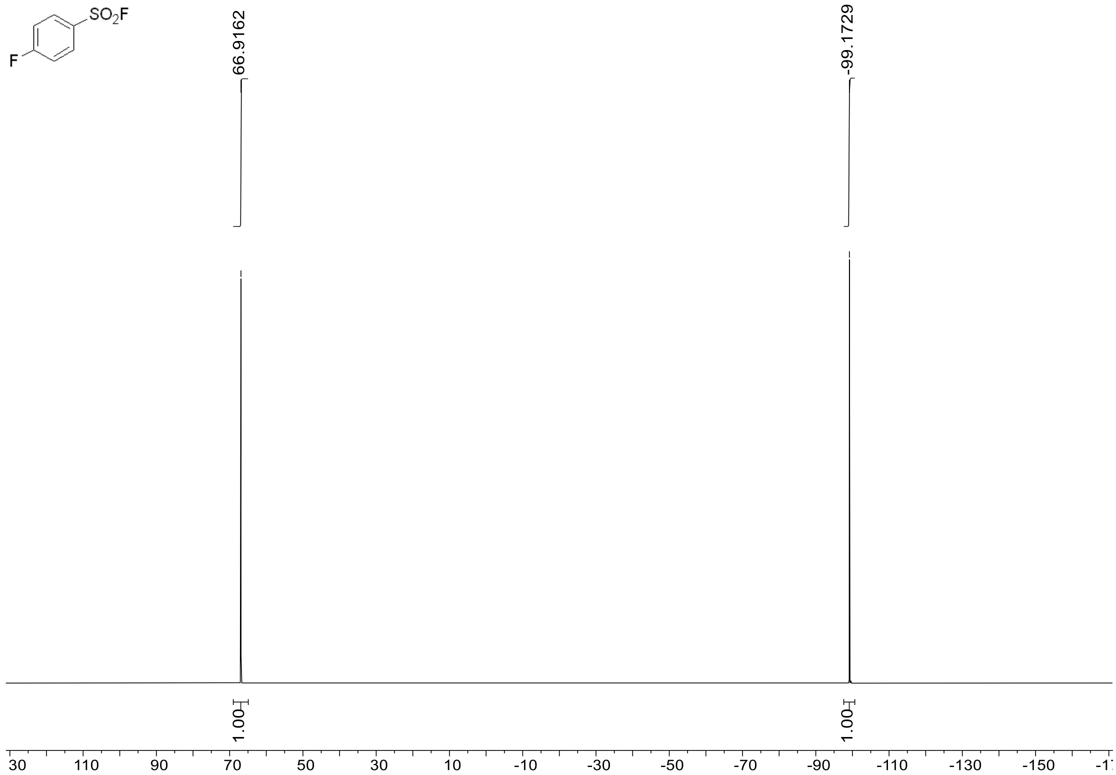

4-Chlorobenzenesulfonyl fluoride (14)

^{1}H-NMR Spectrum:

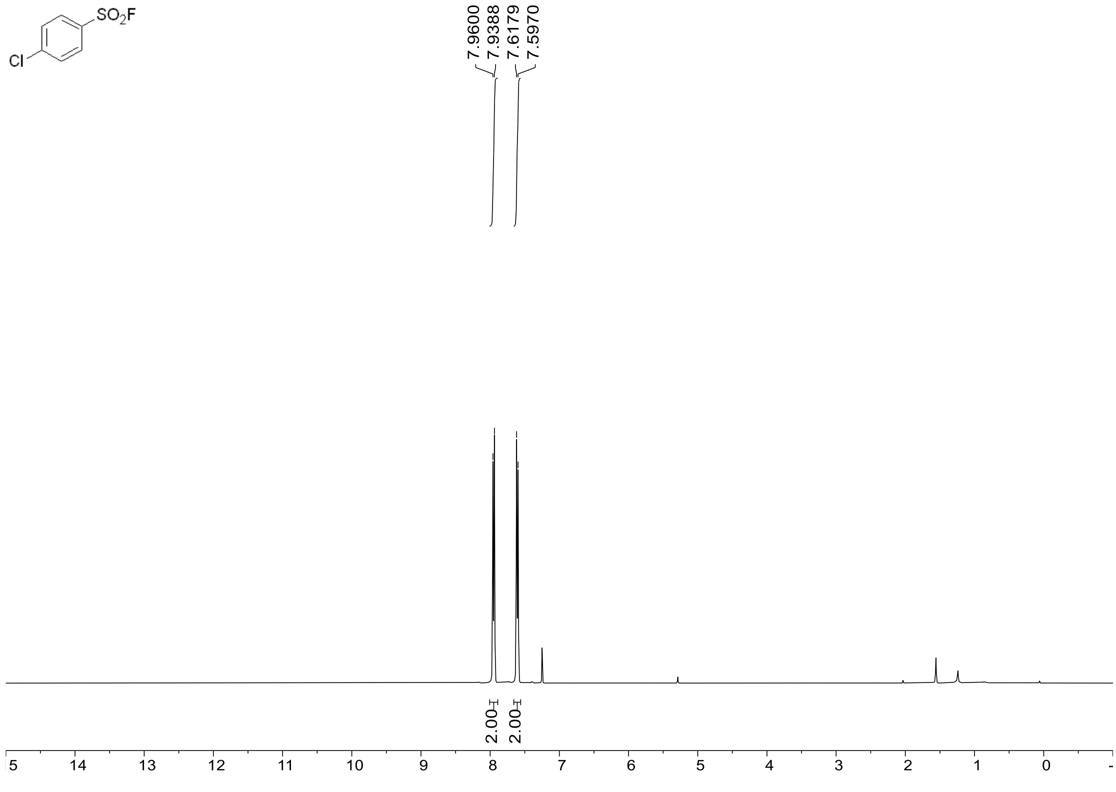

^{13}C-NMR Spectrum:

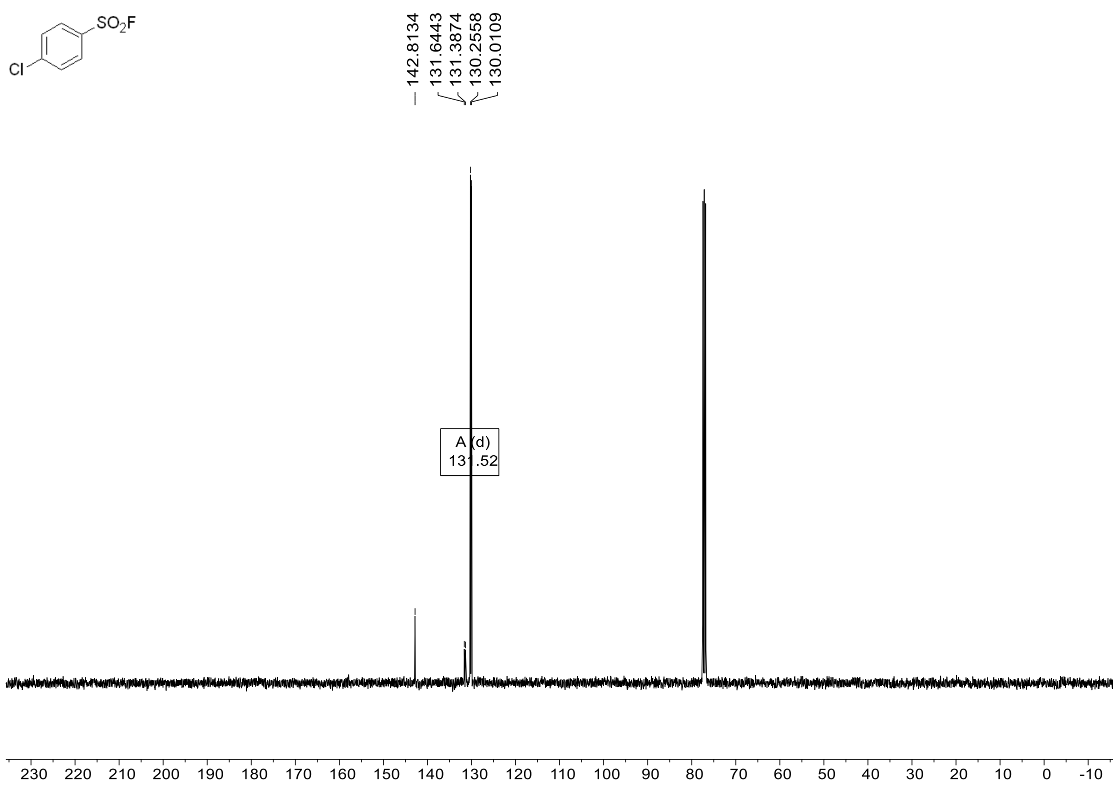

^{19}F-NMR Spectrum:

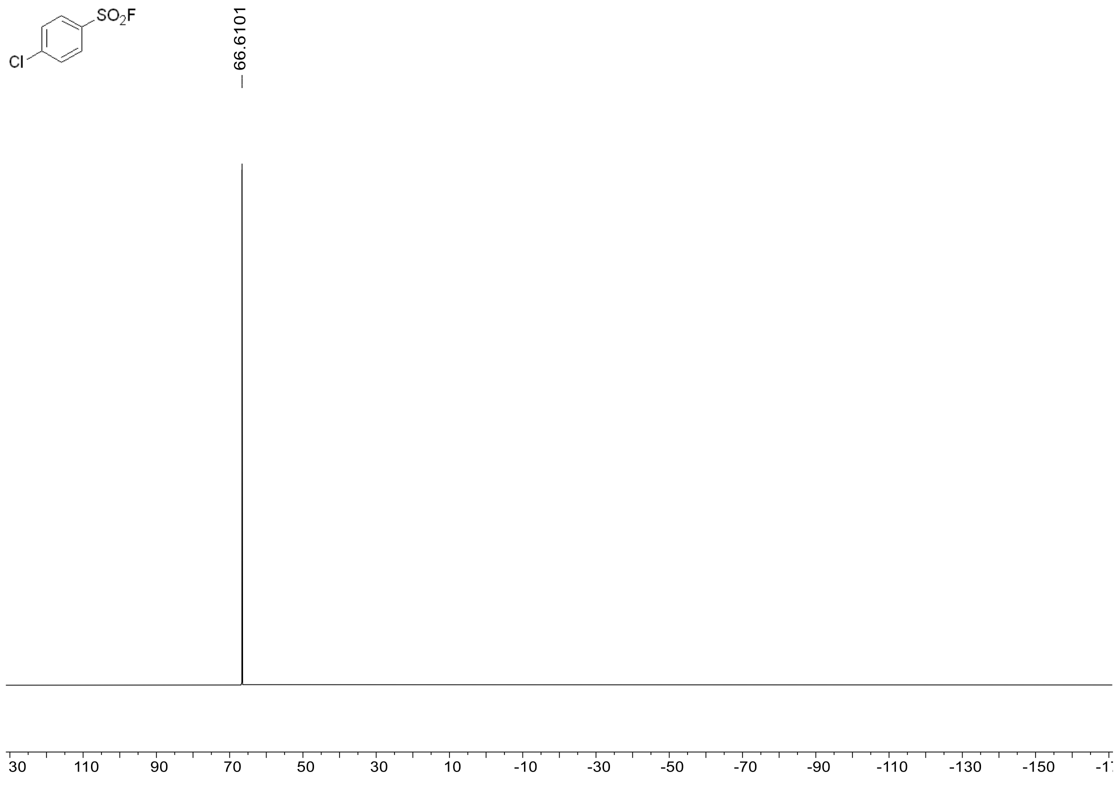

4-Bromobenzenesulfonyl fluoride (15)

^{1}H-NMR Spectrum:

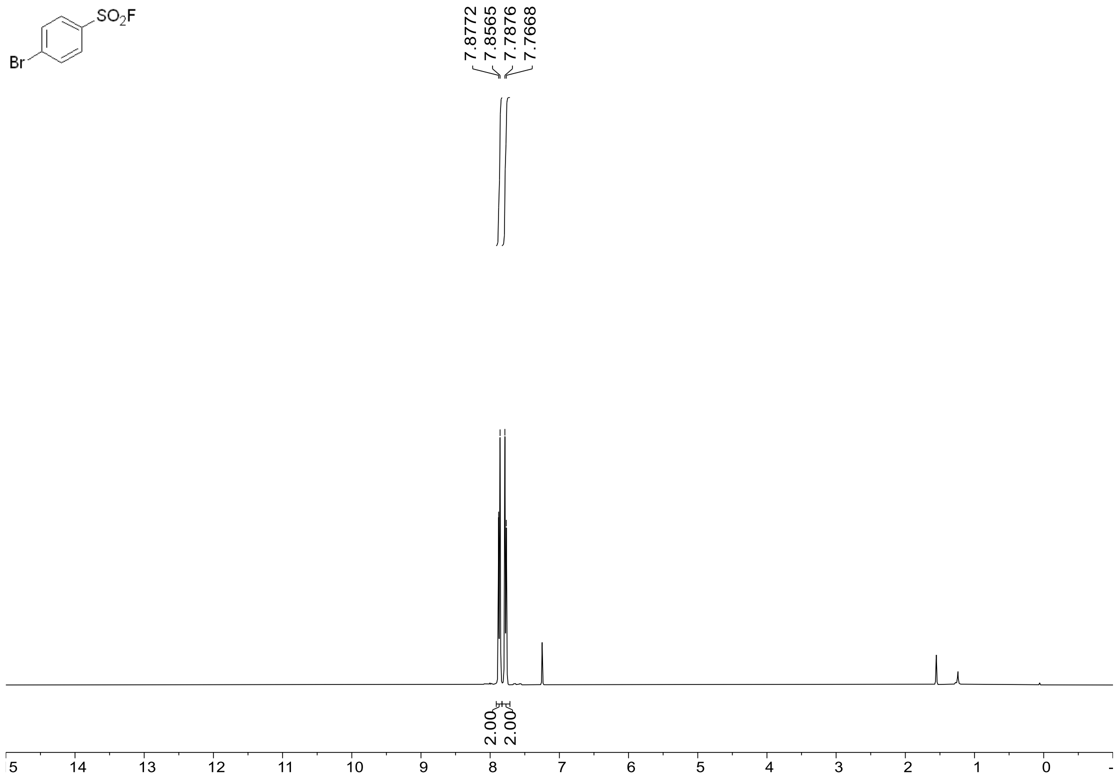

^{13}C-NMR Spectrum:

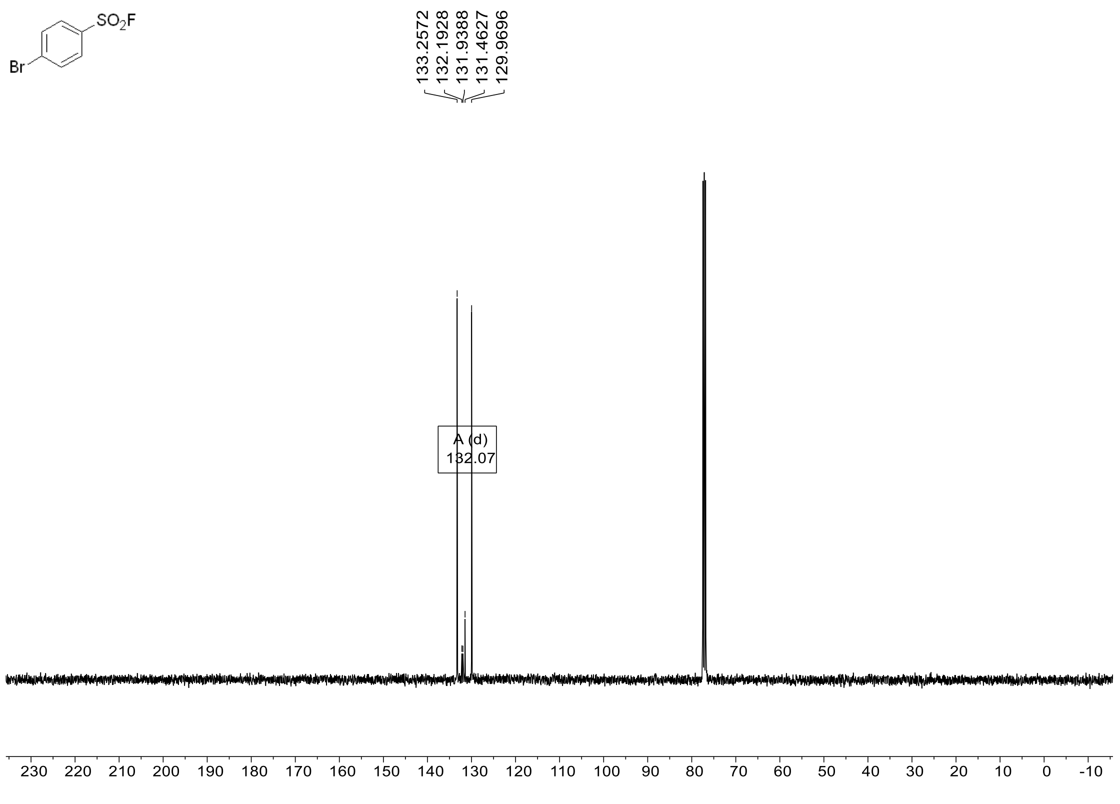

^{19}F-NMR Spectrum:

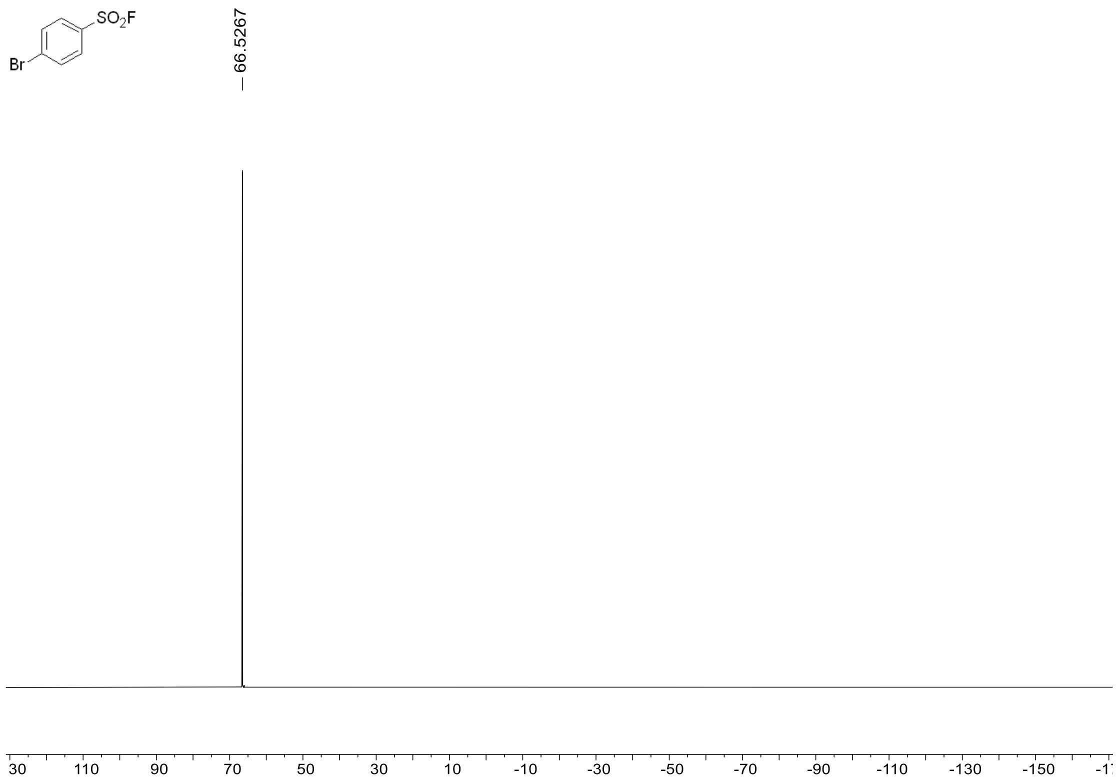

4-(Piperidine-1-carbonyl)benzenesulfonyl fluoride (16)

^{1}H-NMR Spectrum:

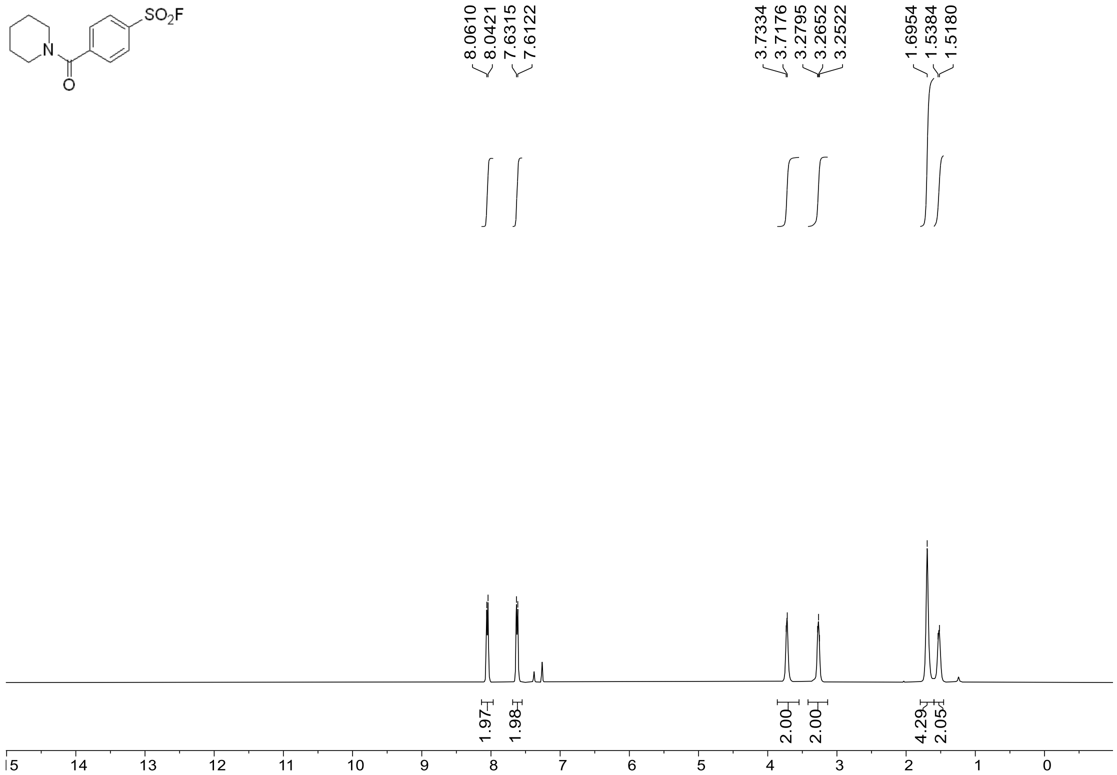

^{13}C-NMR Spectrum:

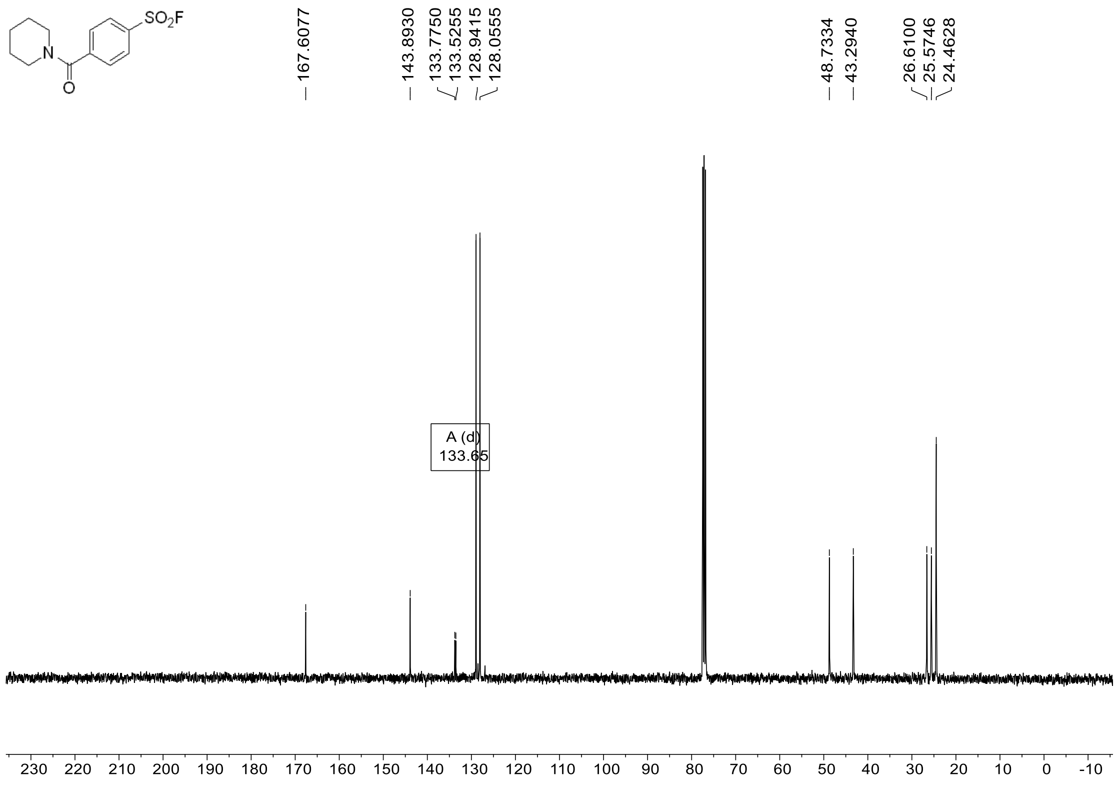

^{19}F-NMR Spectrum:

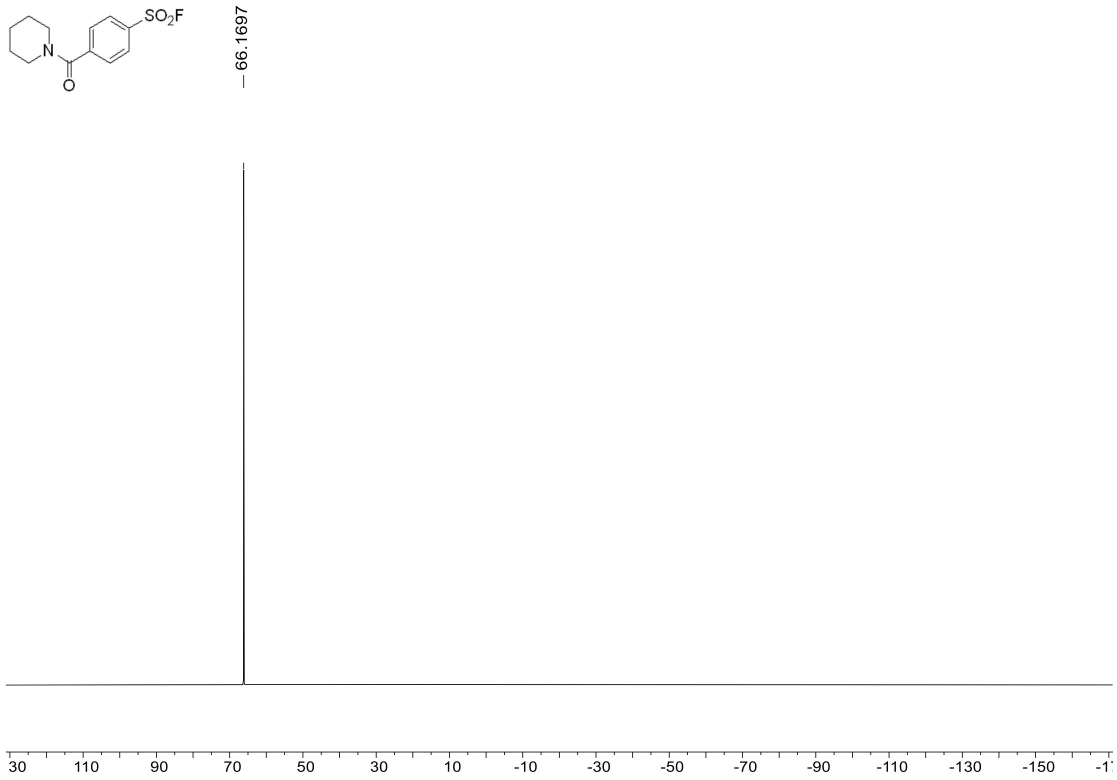

4-(Trifluoromethyl)benzenesulfonyl fluoride (17)

^{1}H-NMR Spectrum:

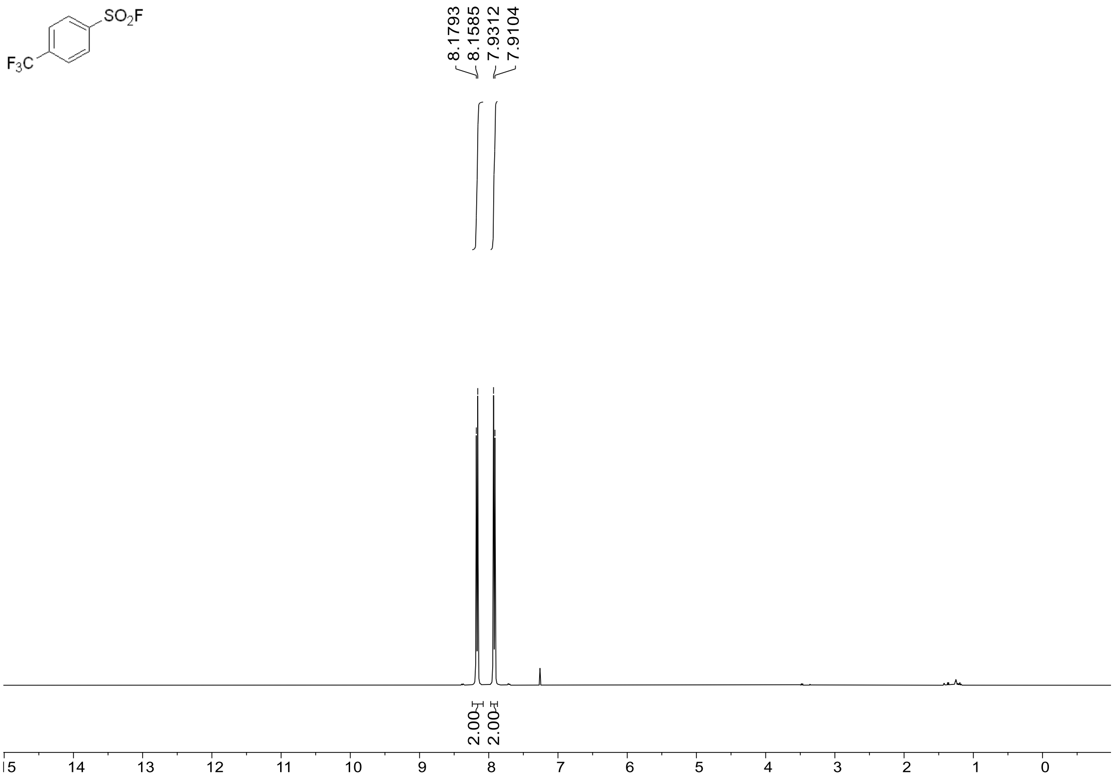

^{13}C-NMR Spectrum:

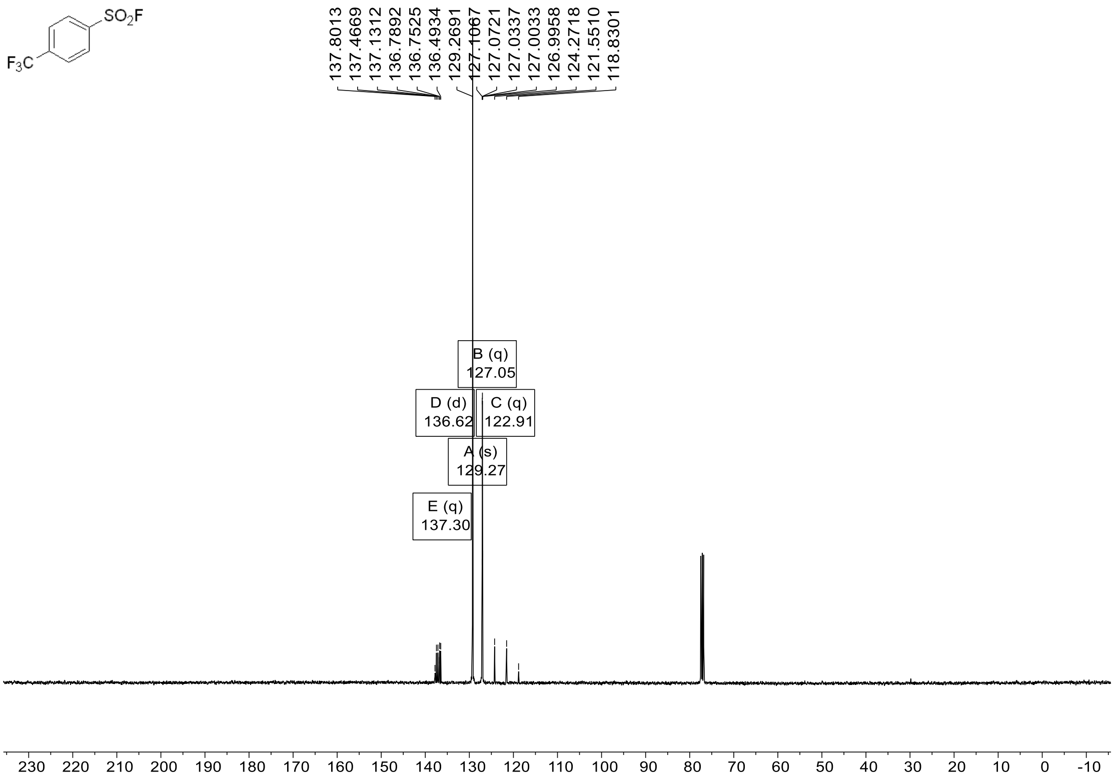

^{19}F-NMR Spectrum:

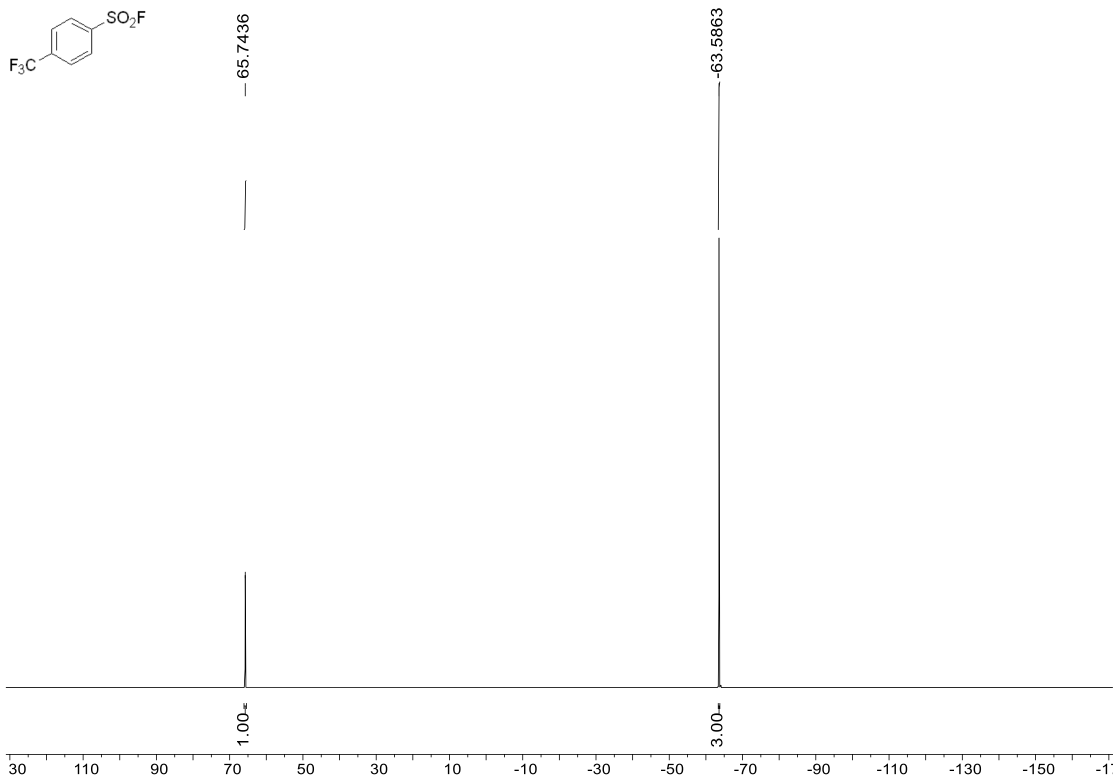

2-Methylbenzo[d]thiazole-6-sulfonyl fluoride (18)

^{1}H-NMR Spectrum:

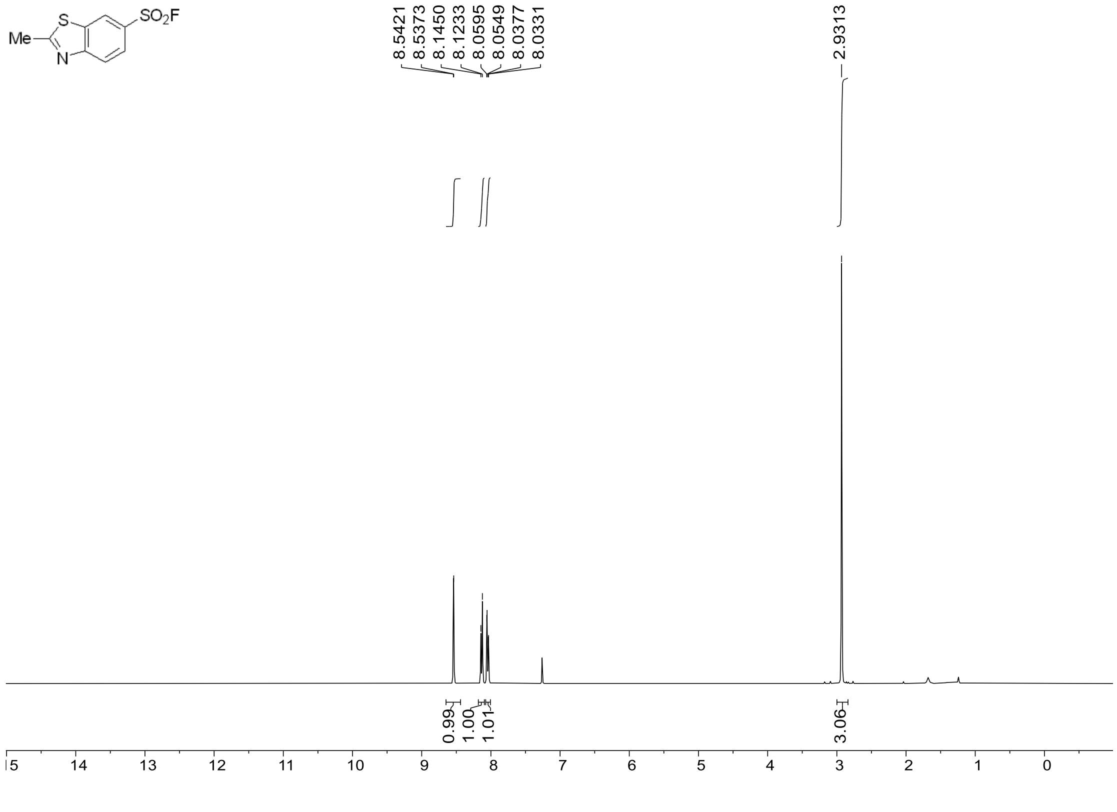

^{13}C-NMR Spectrum:

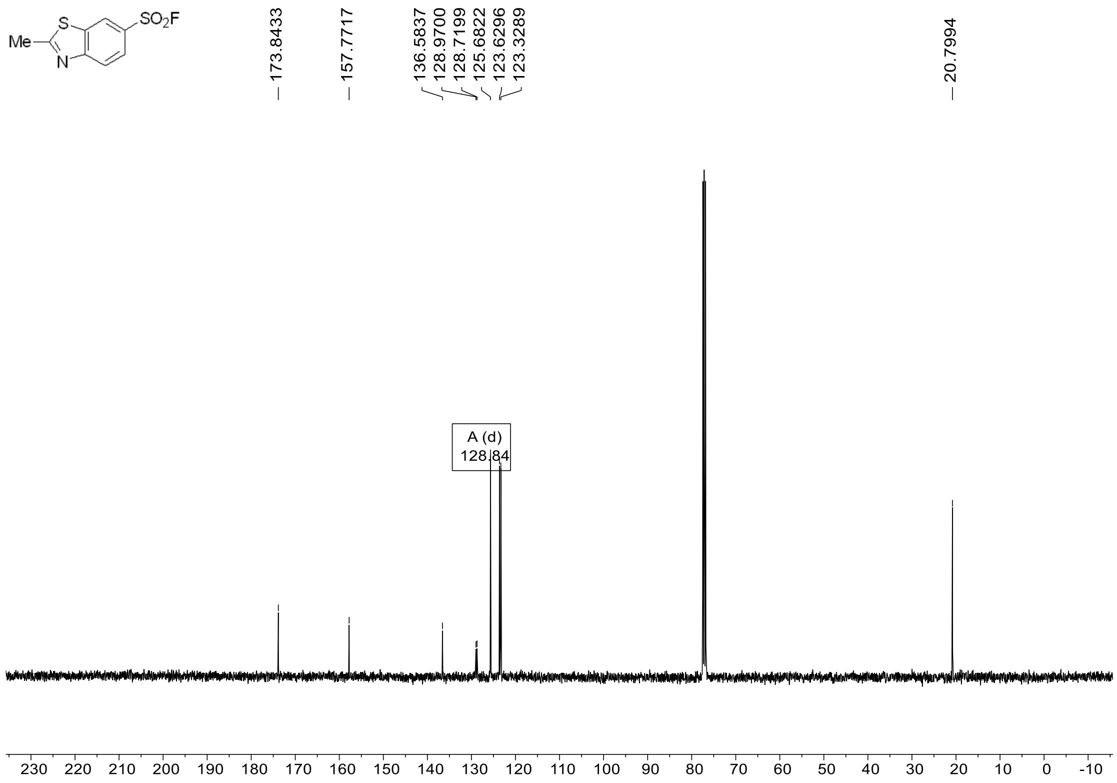

^{19}F-NMR Spectrum:

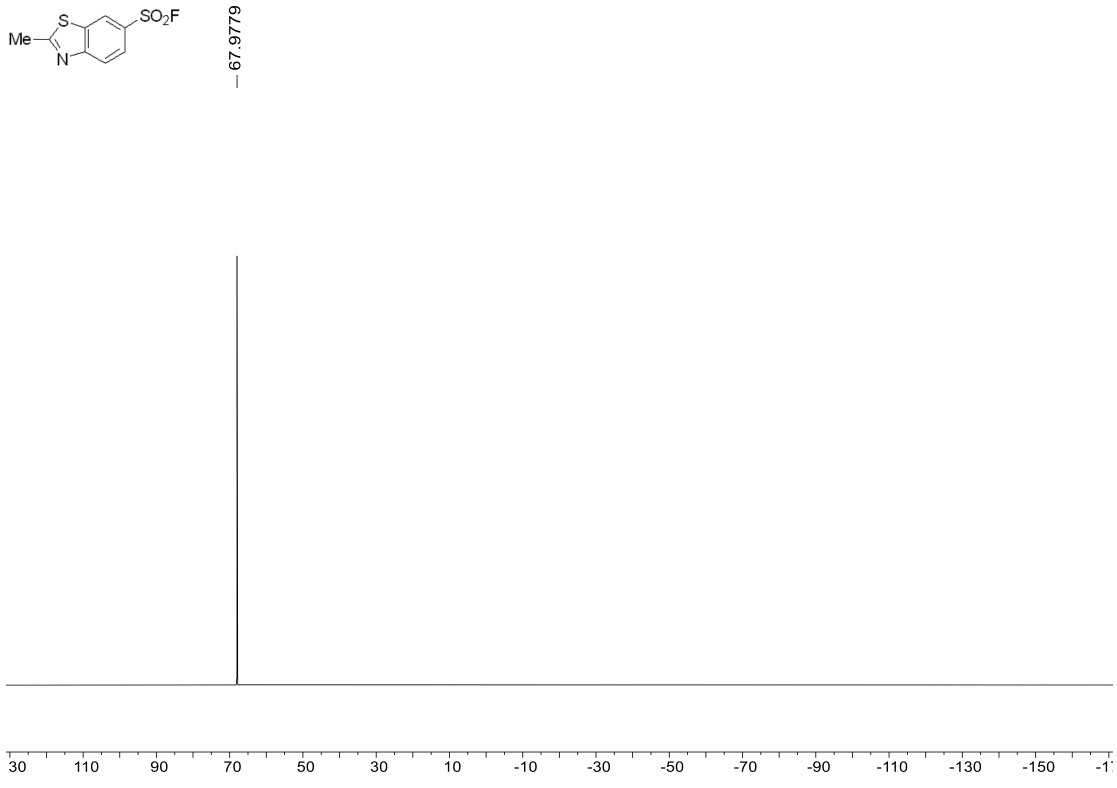

2-Acetylbenzenesulfonyl fluoride (19)

^{1}H-NMR Spectrum:

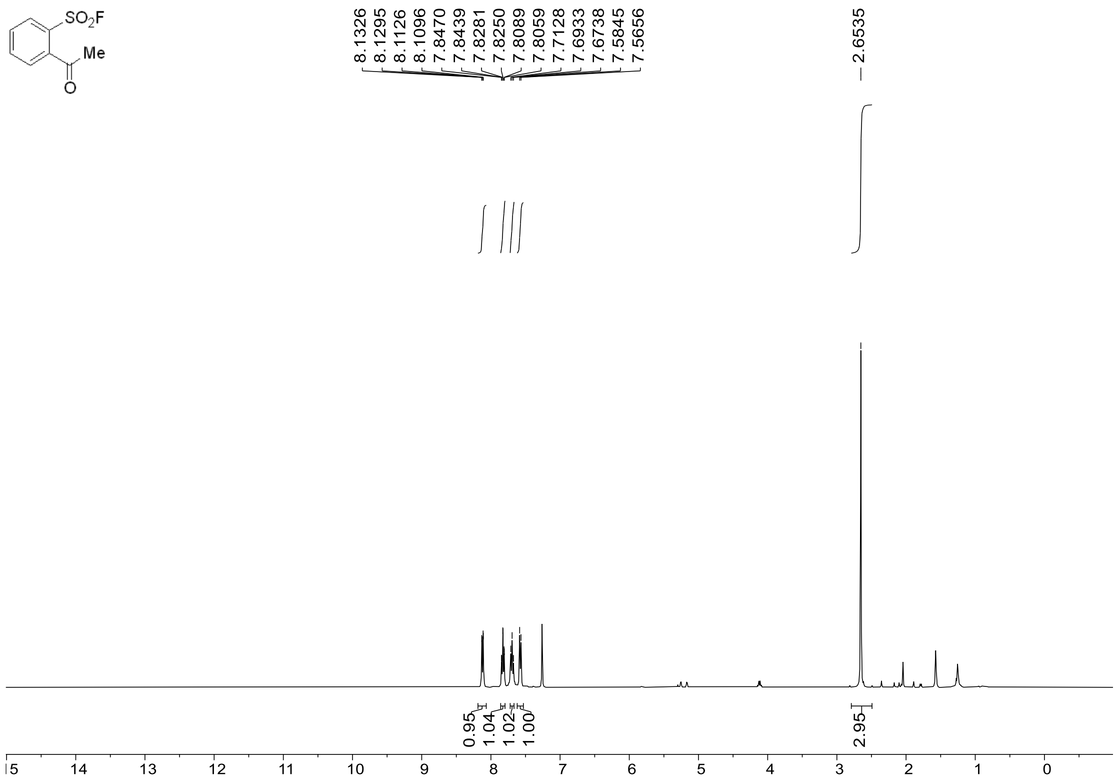

^{13}C-NMR Spectrum:

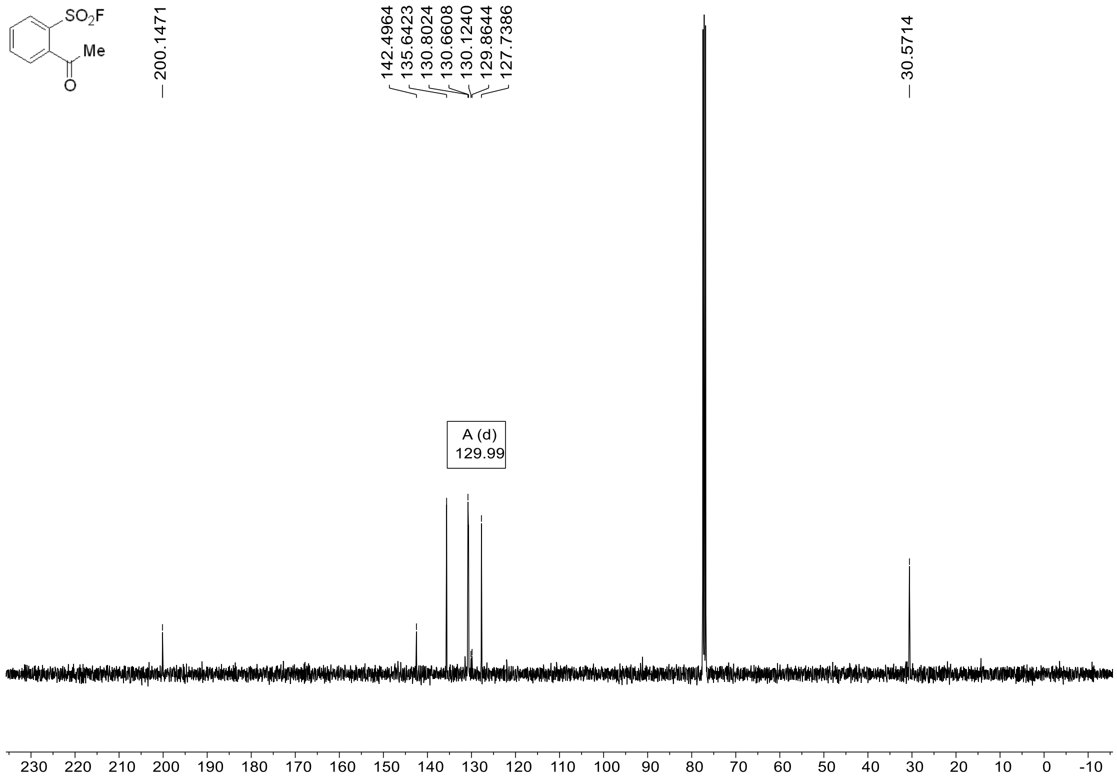

^{19}F-NMR Spectrum:

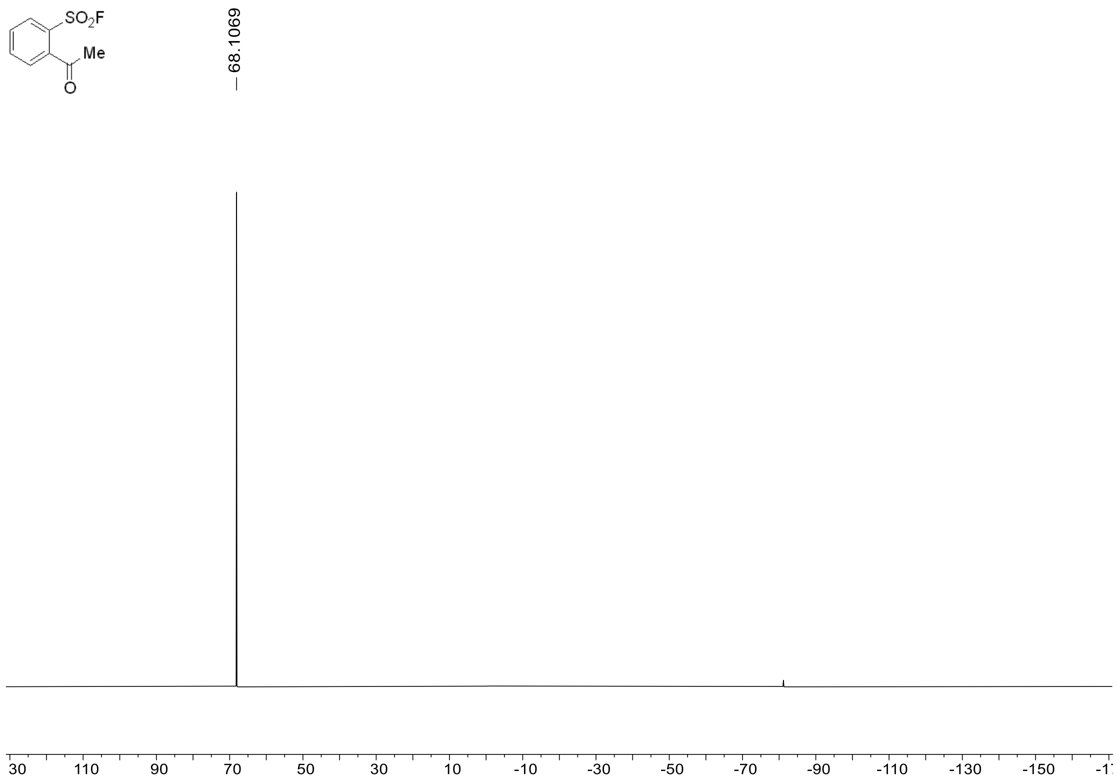

Methyl 3-(fluorosulfonyl)benzoate (20)

^{1}H-NMR Spectrum:

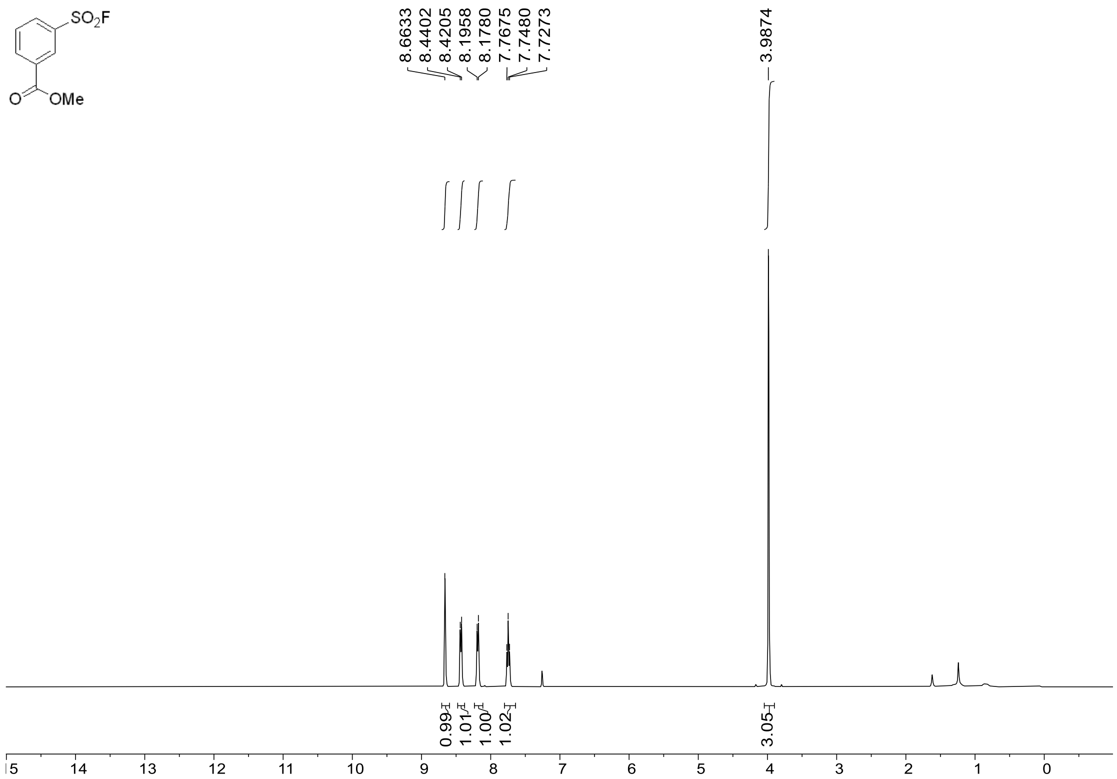

^{13}C-NMR Spectrum:

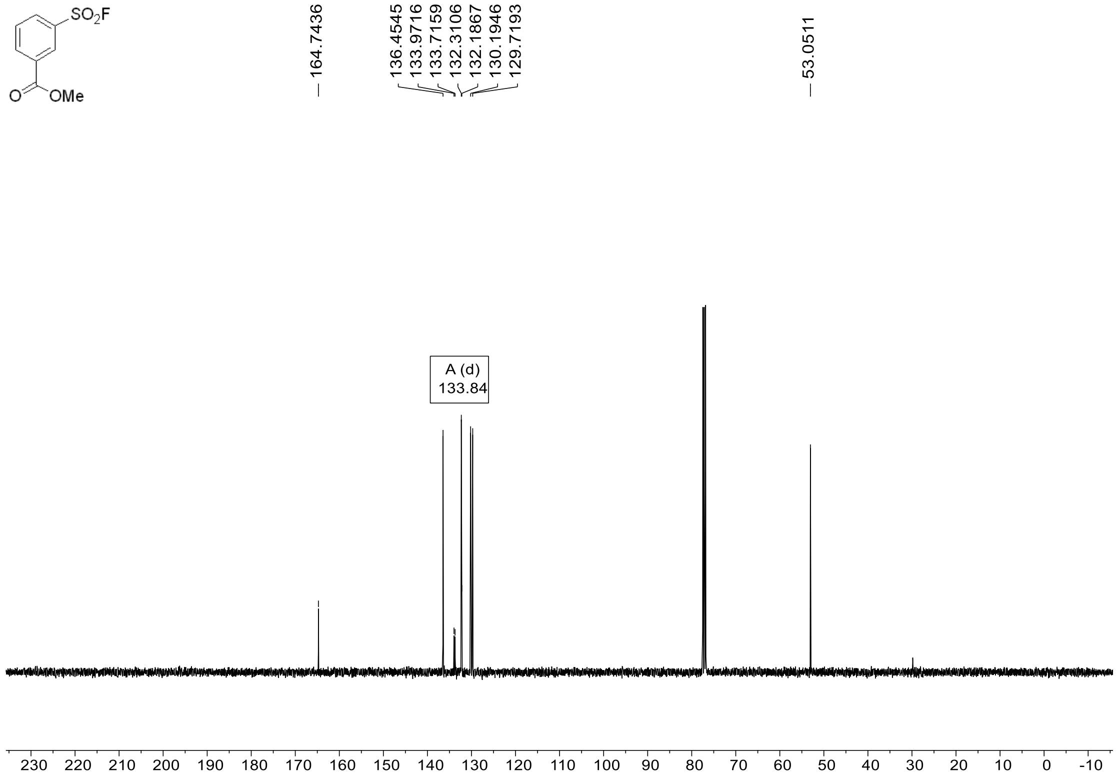

^{19}F-NMR Spectrum:

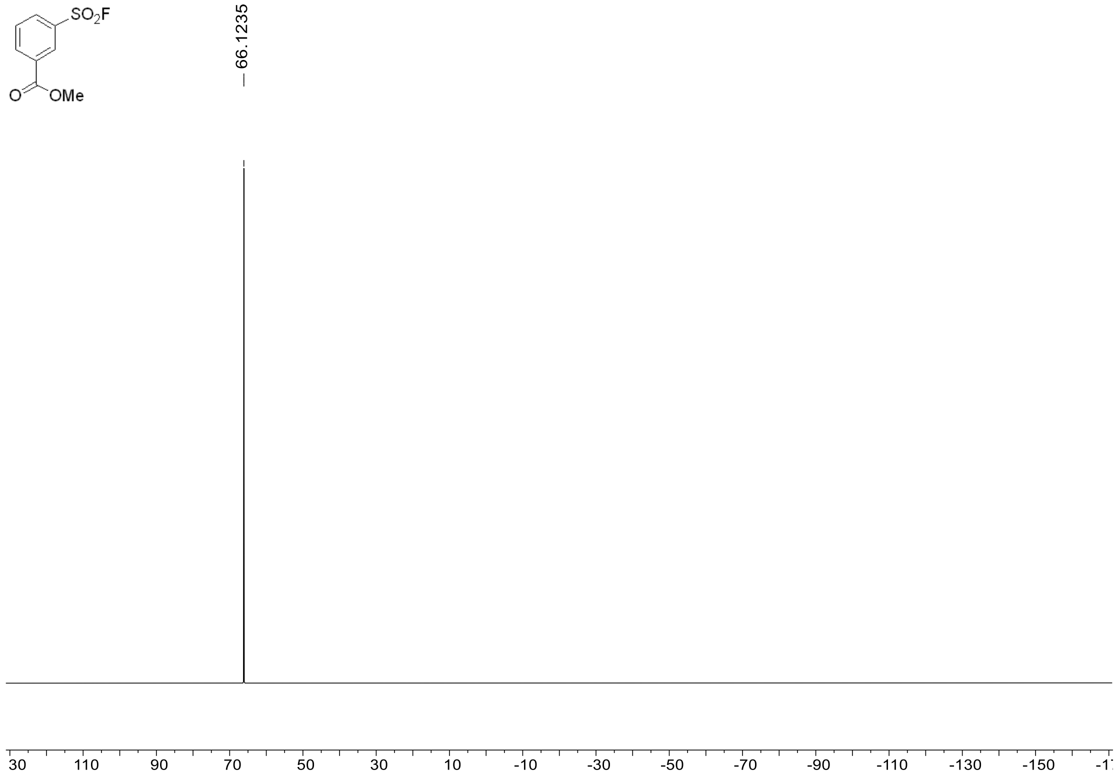

3-(Fluorosulfonyl)benzoic acid (21)

^{1}H-NMR Spectrum:

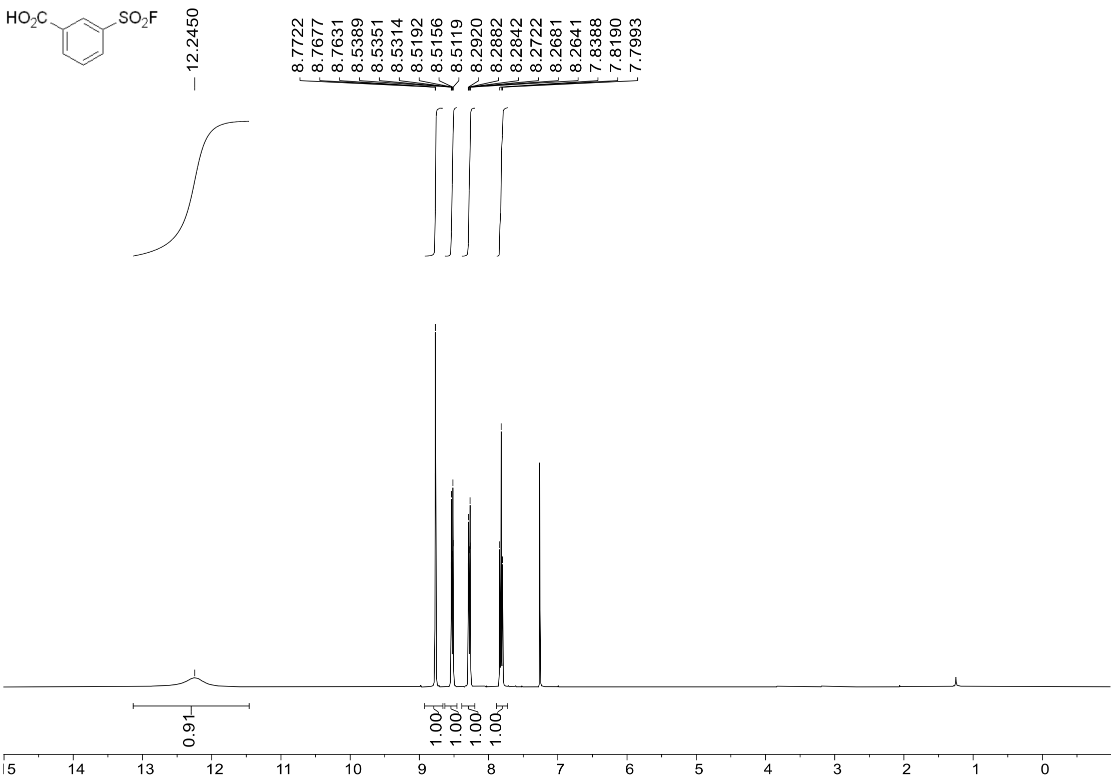

^{13}C-NMR Spectrum:

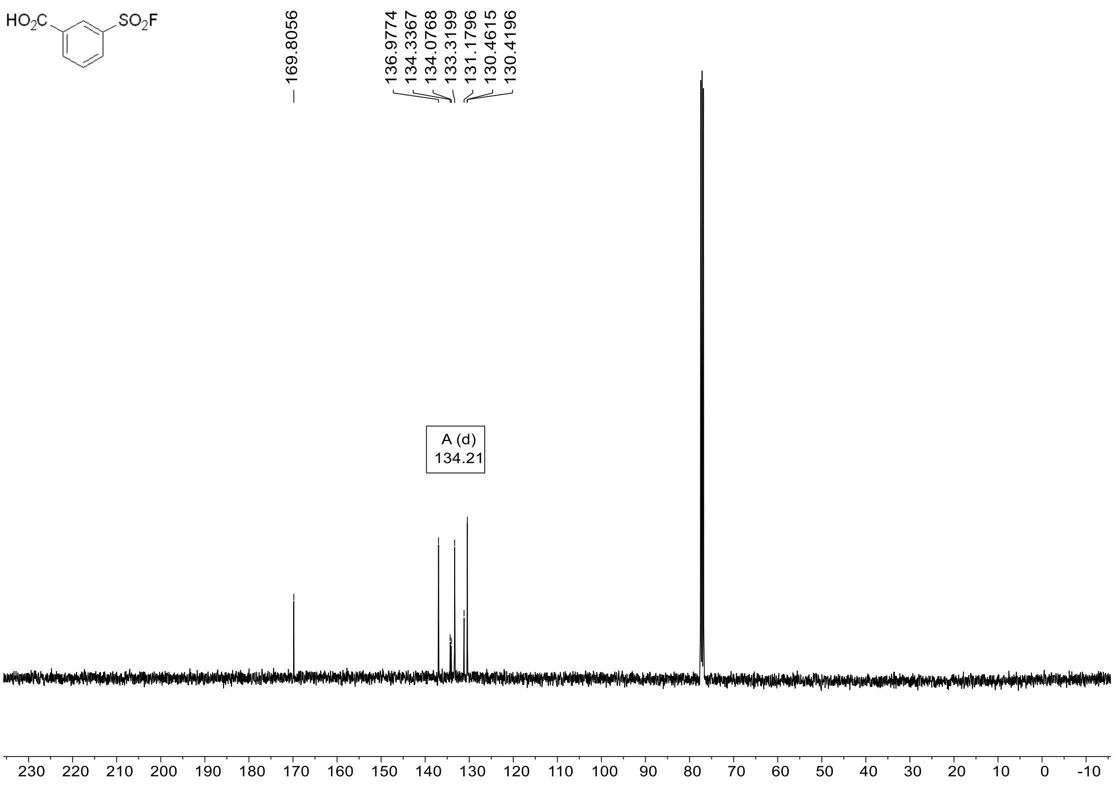

^{19}F-NMR Spectrum:

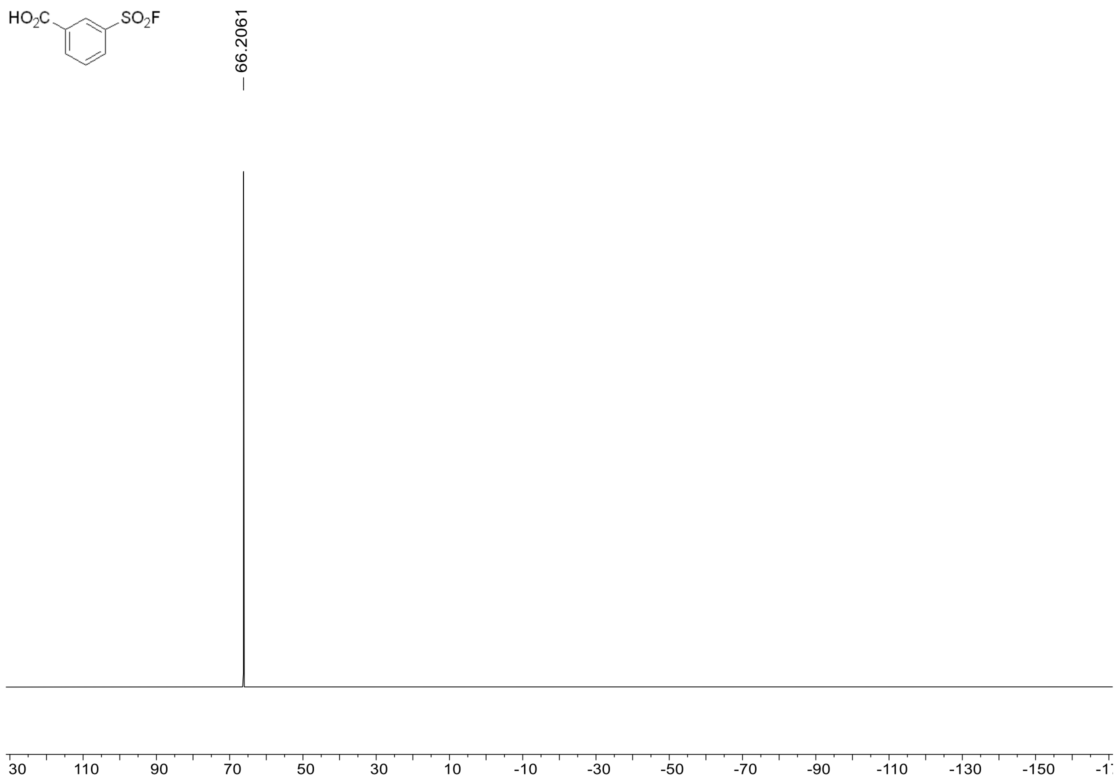

3-Cyanobenzenesulfonyl fluoride (22)

^{1}H-NMR Spectrum:

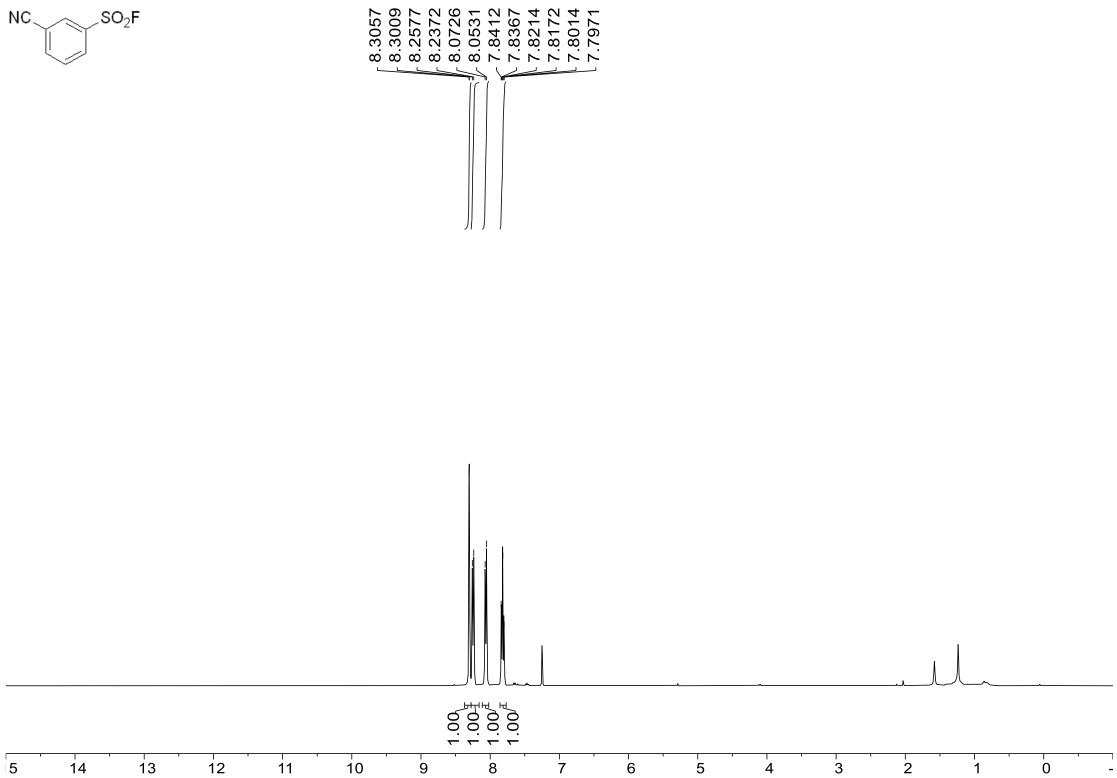

^{13}C-NMR Spectrum:

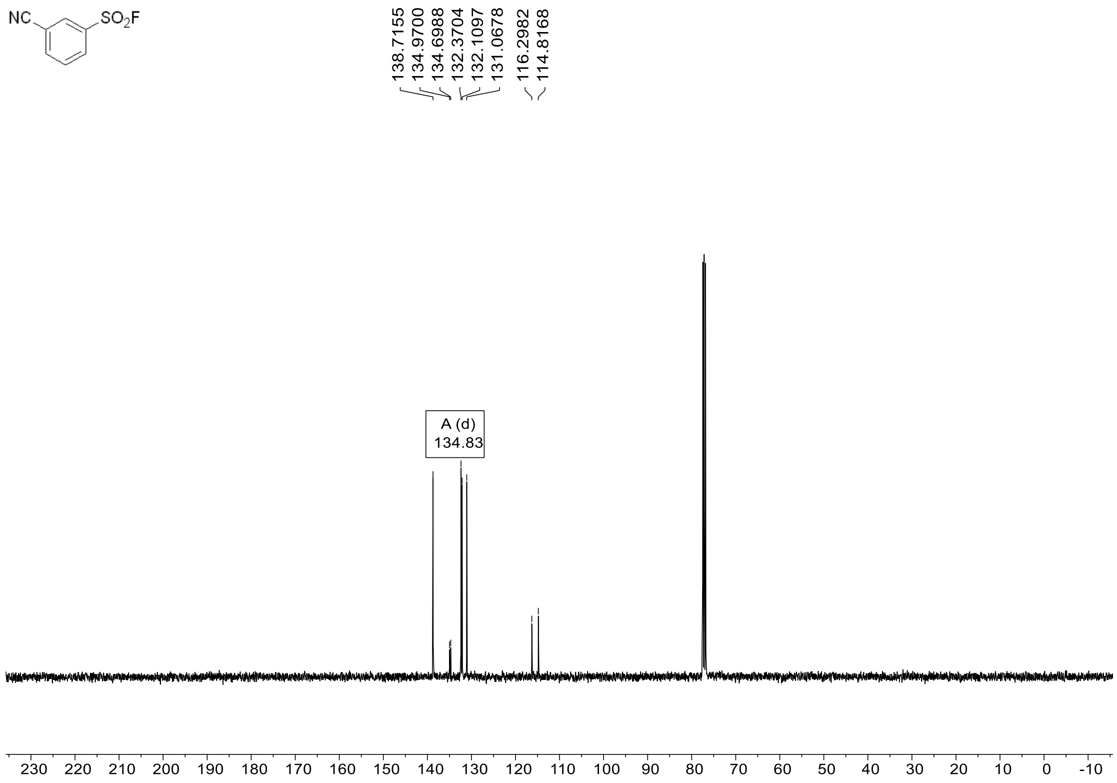

^{19}F-NMR Spectrum:

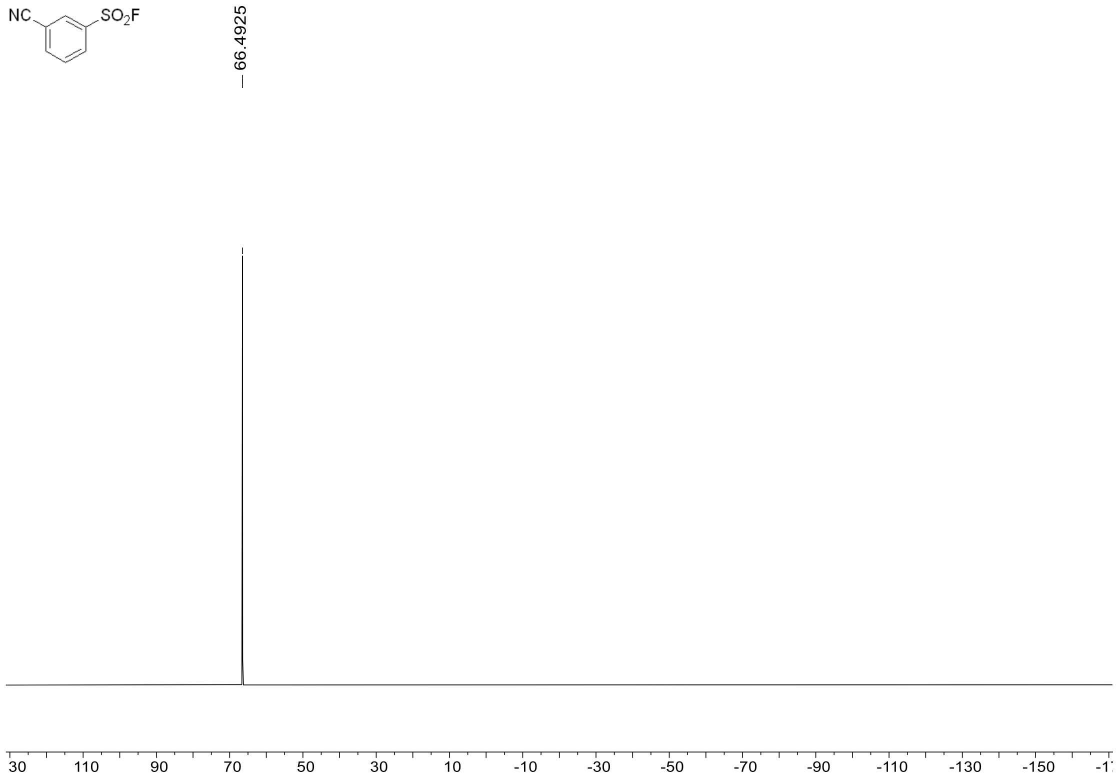

2-Cyanobenzenesulfonyl fluoride (23)

^{1}H-NMR Spectrum:

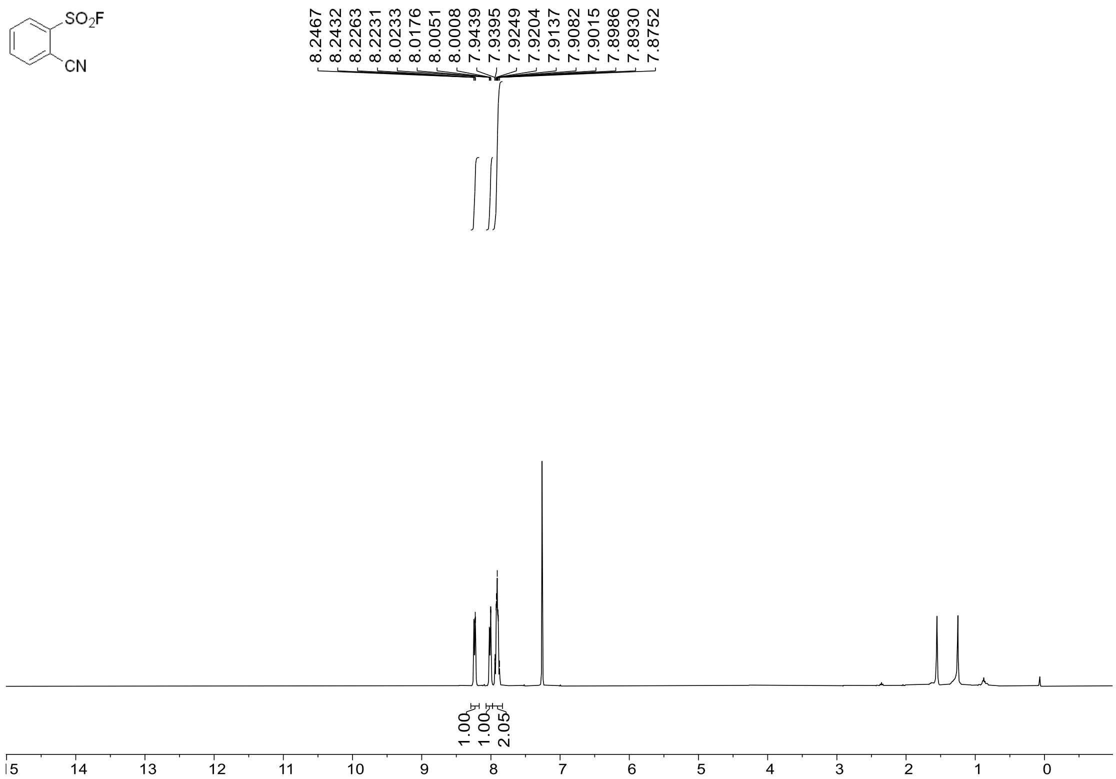

^{13}C-NMR Spectrum:

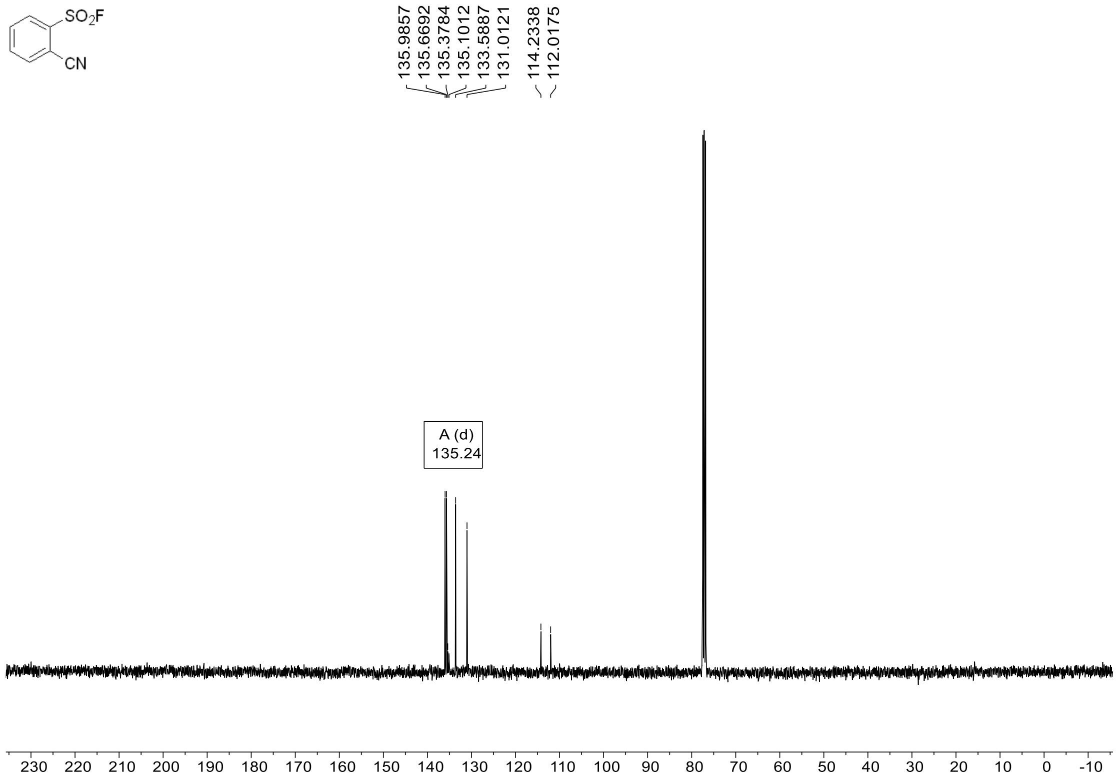

^{19}F-NMR Spectrum:

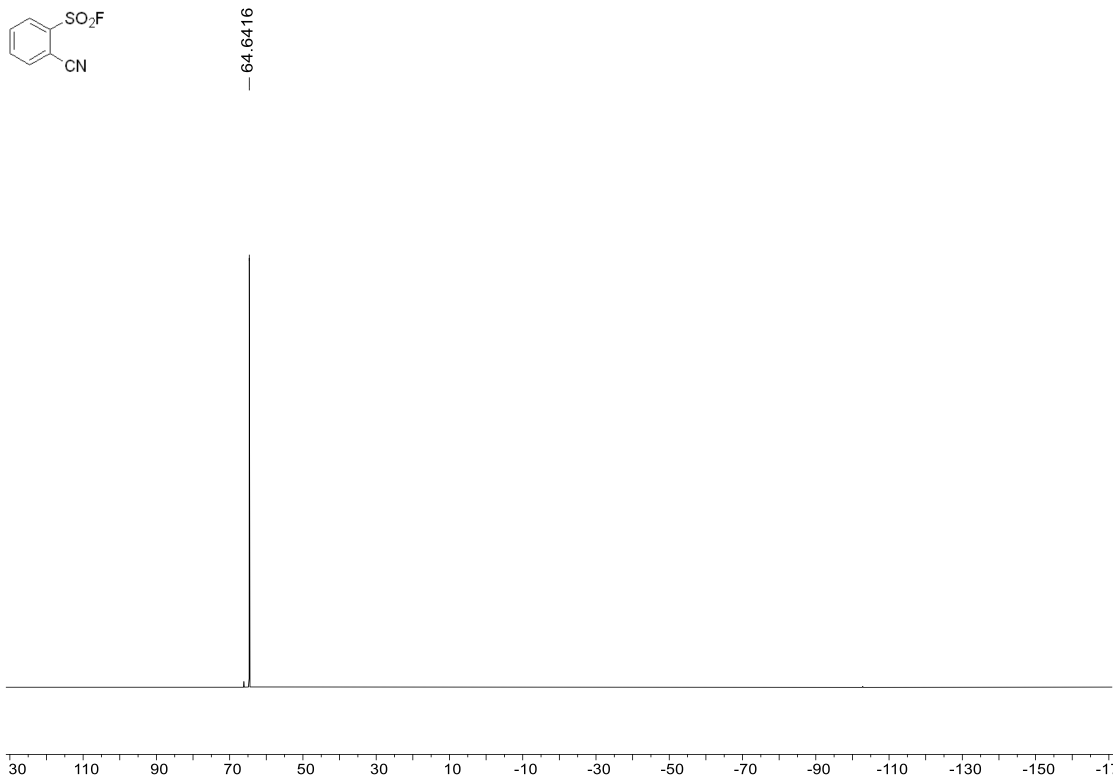

4-Cyanobenzenesulfonyl fluoride (24)

^{1}H-NMR Spectrum:

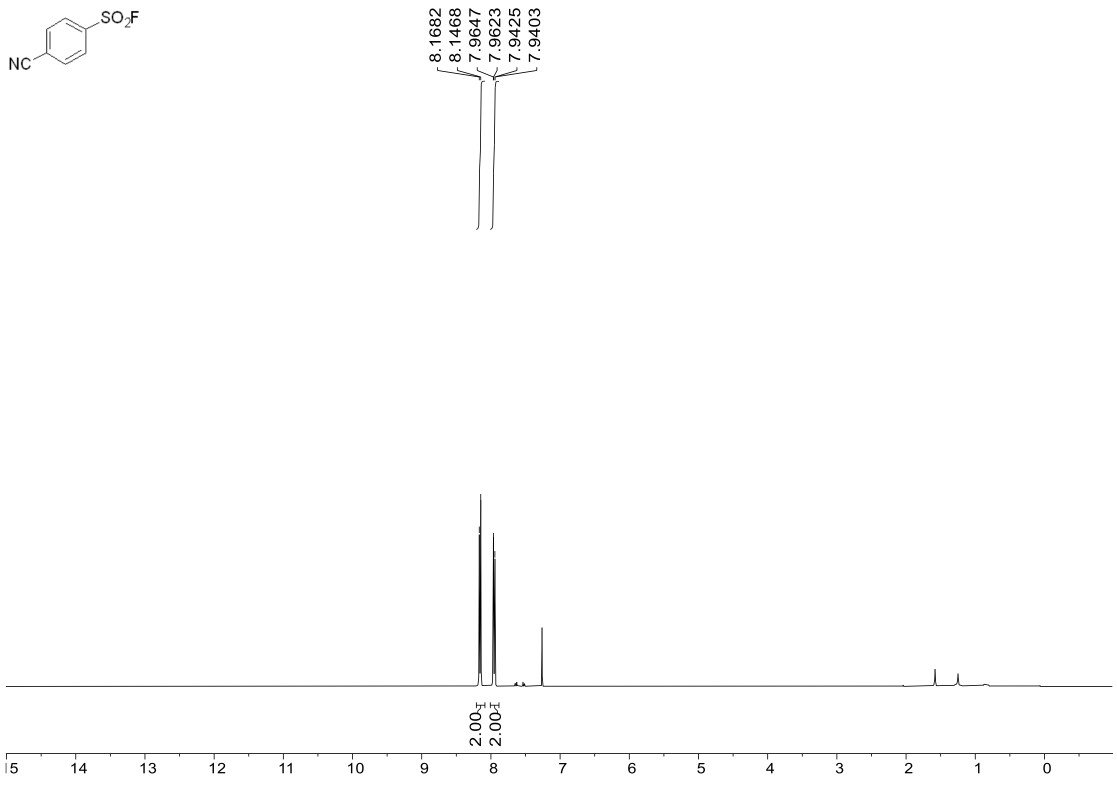

^{13}C-NMR Spectrum:

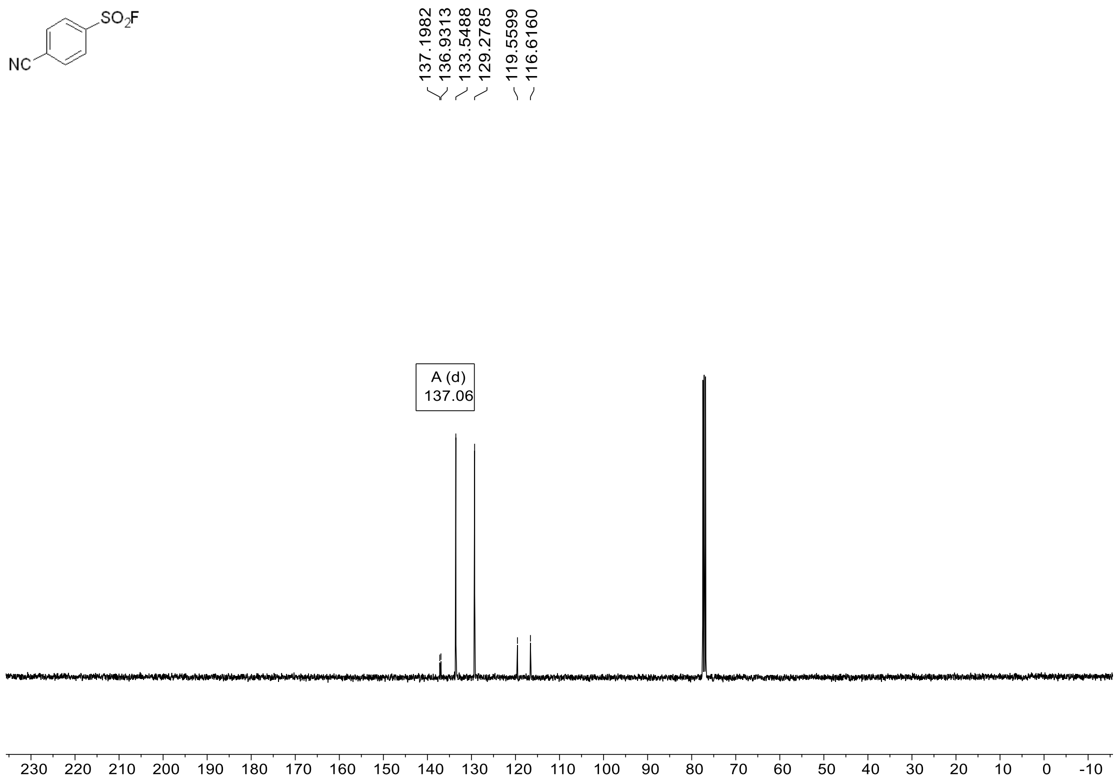

^{19}F-NMR Spectrum:

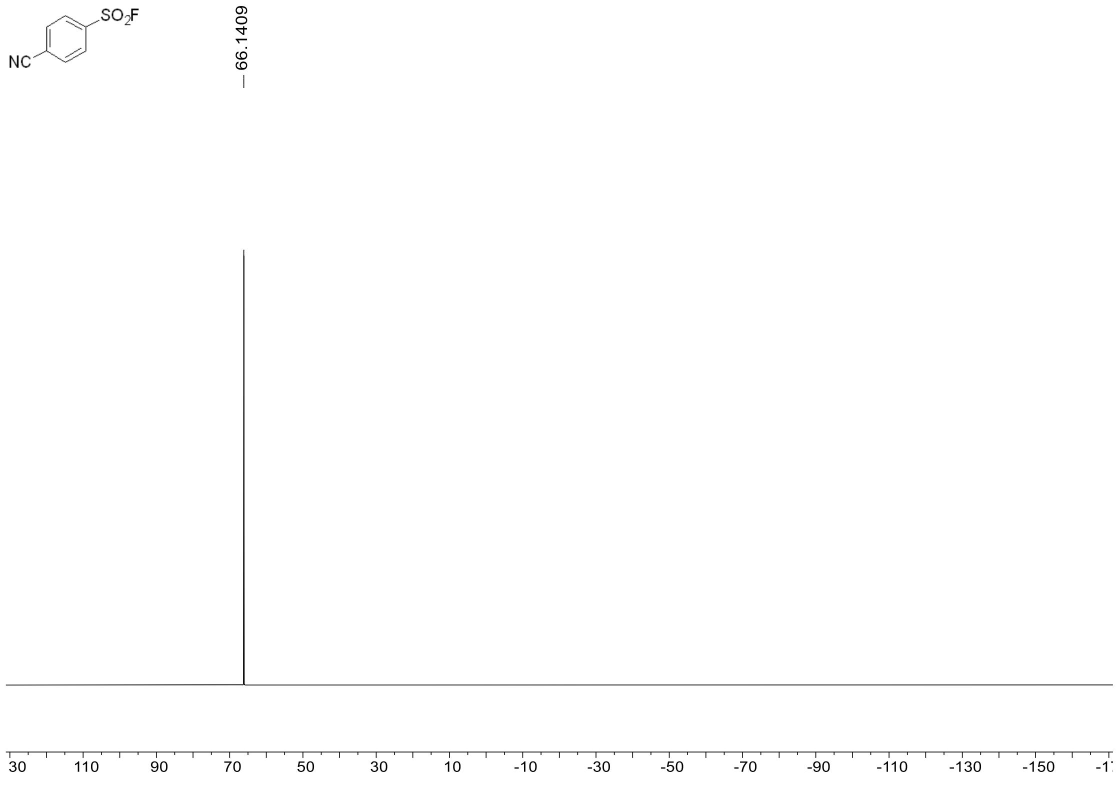

4-(Methylsulfonyl)benzenesulfonyl fluoride (25)

^{1}H-NMR Spectrum:

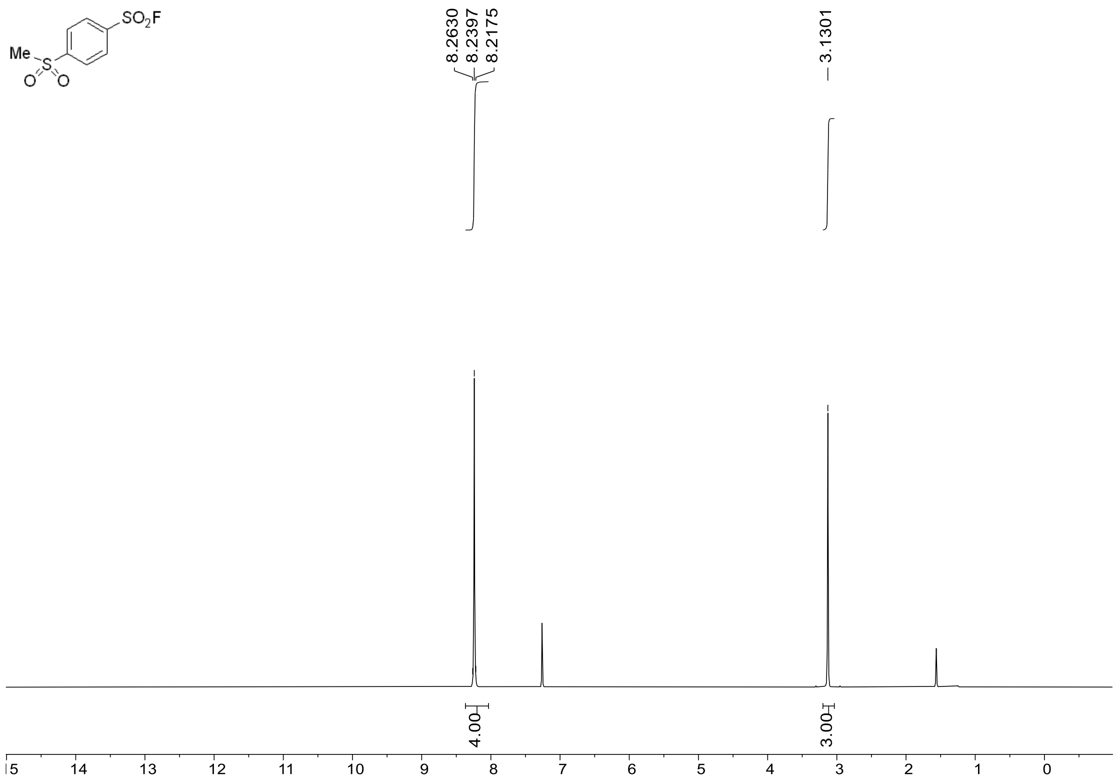

^{13}C-NMR Spectrum:

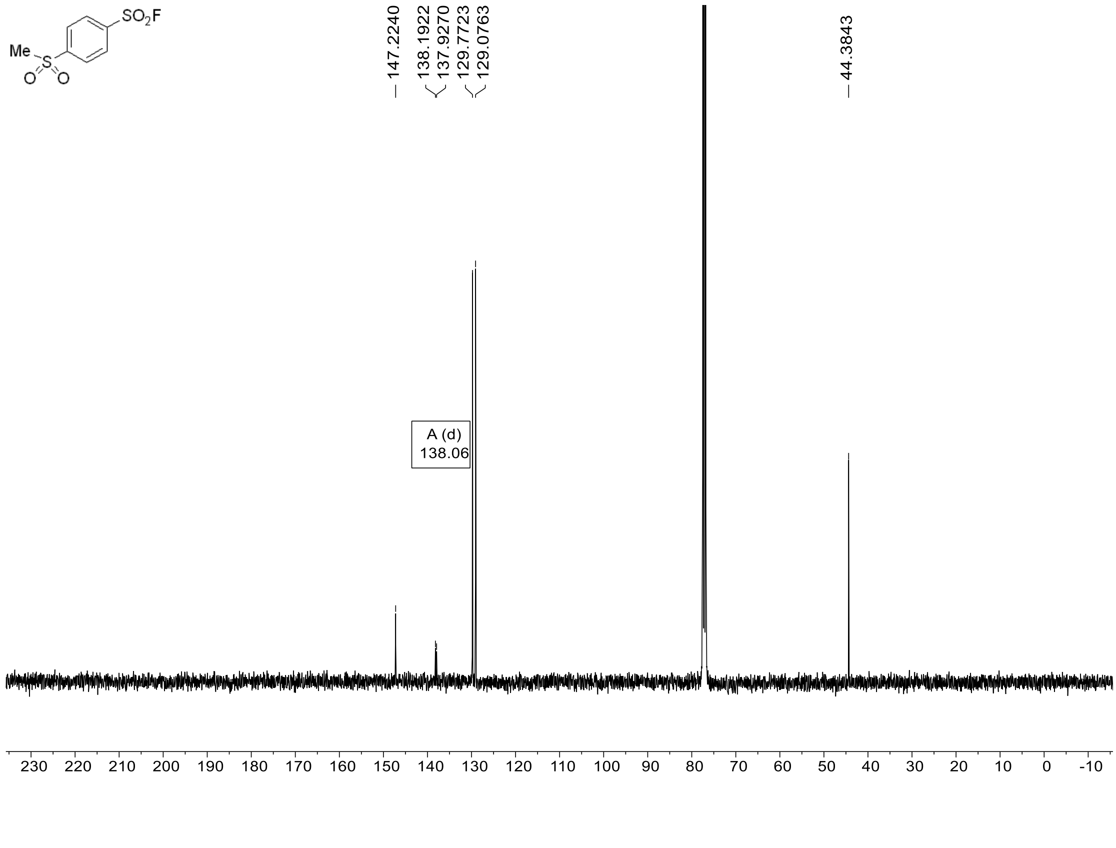

^{19}F-NMR Spectrum:

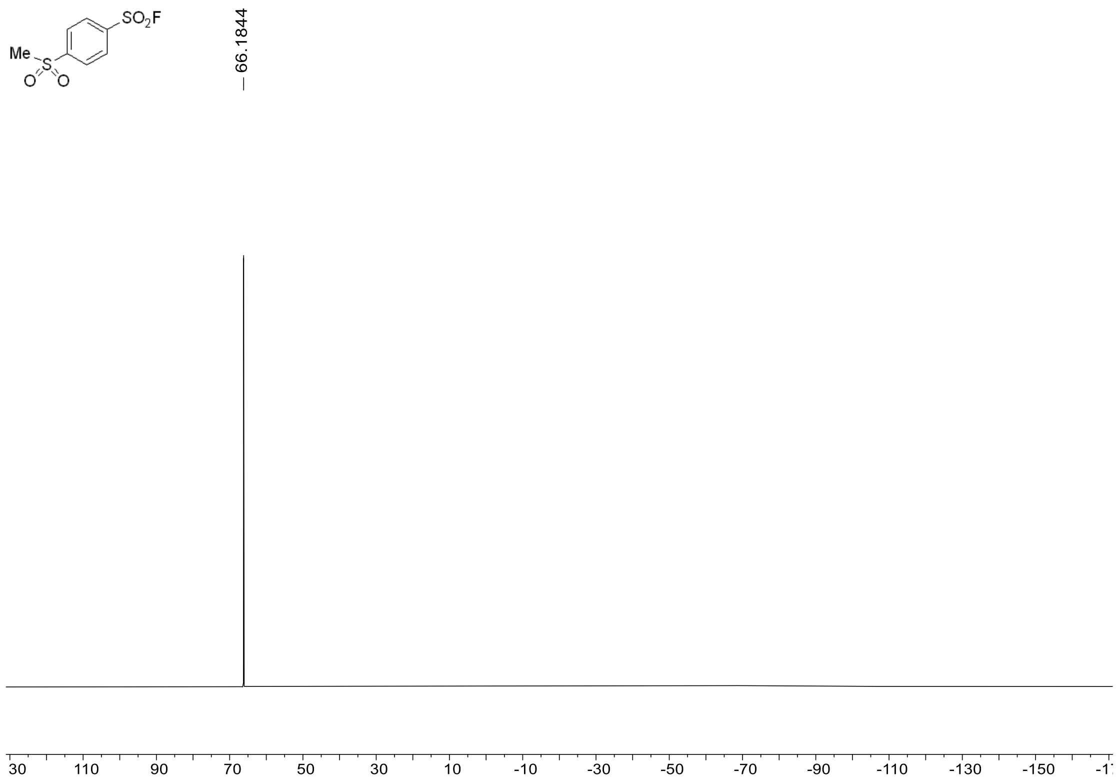

4-Acetylbenzenesulfonyl fluoride (26)

^{1}H-NMR Spectrum:

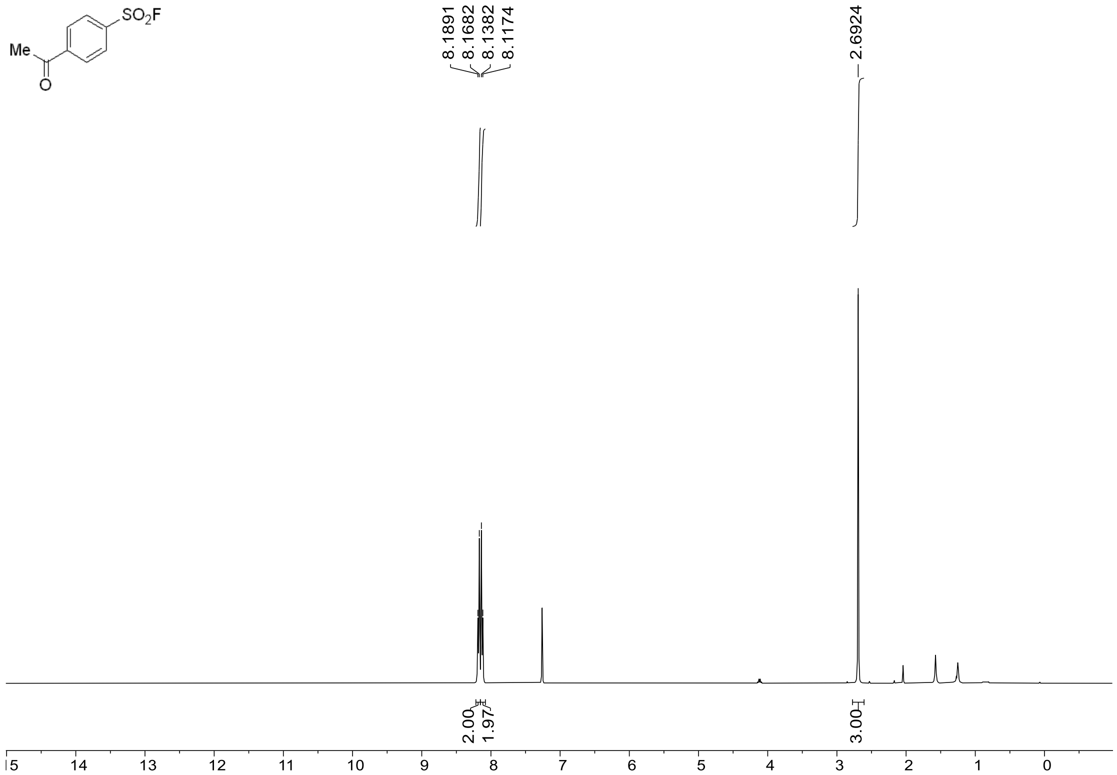

^{13}C-NMR Spectrum:

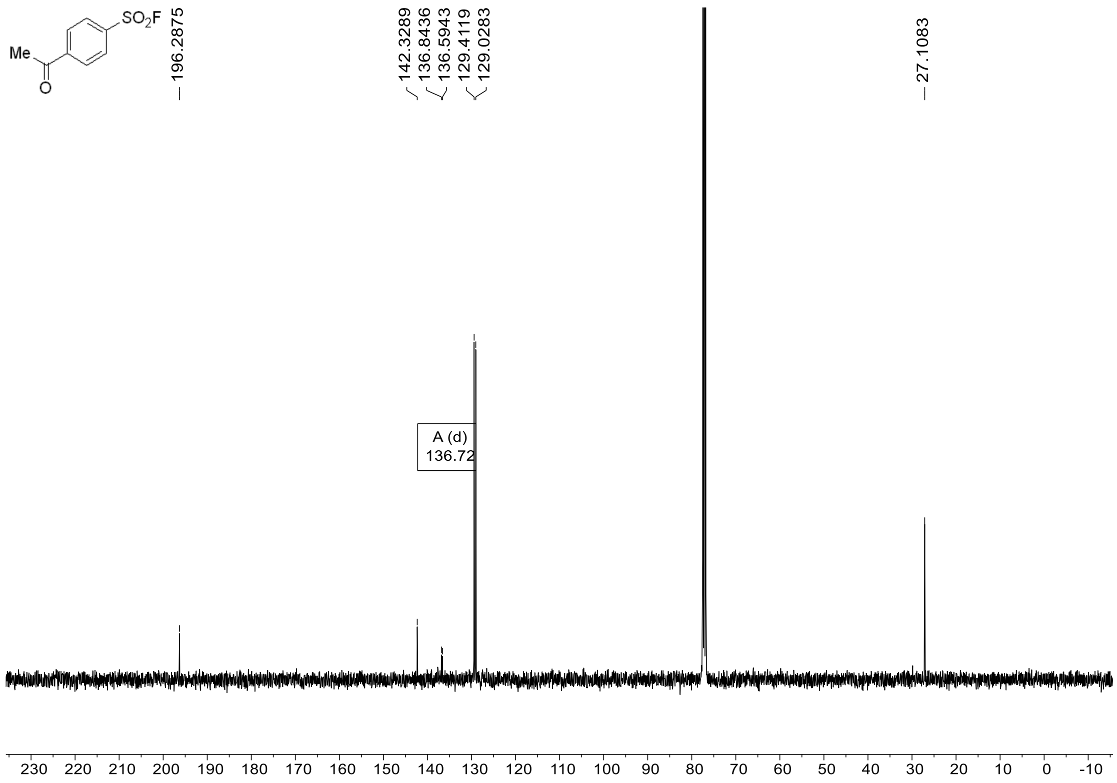

^{19}F-NMR Spectrum:

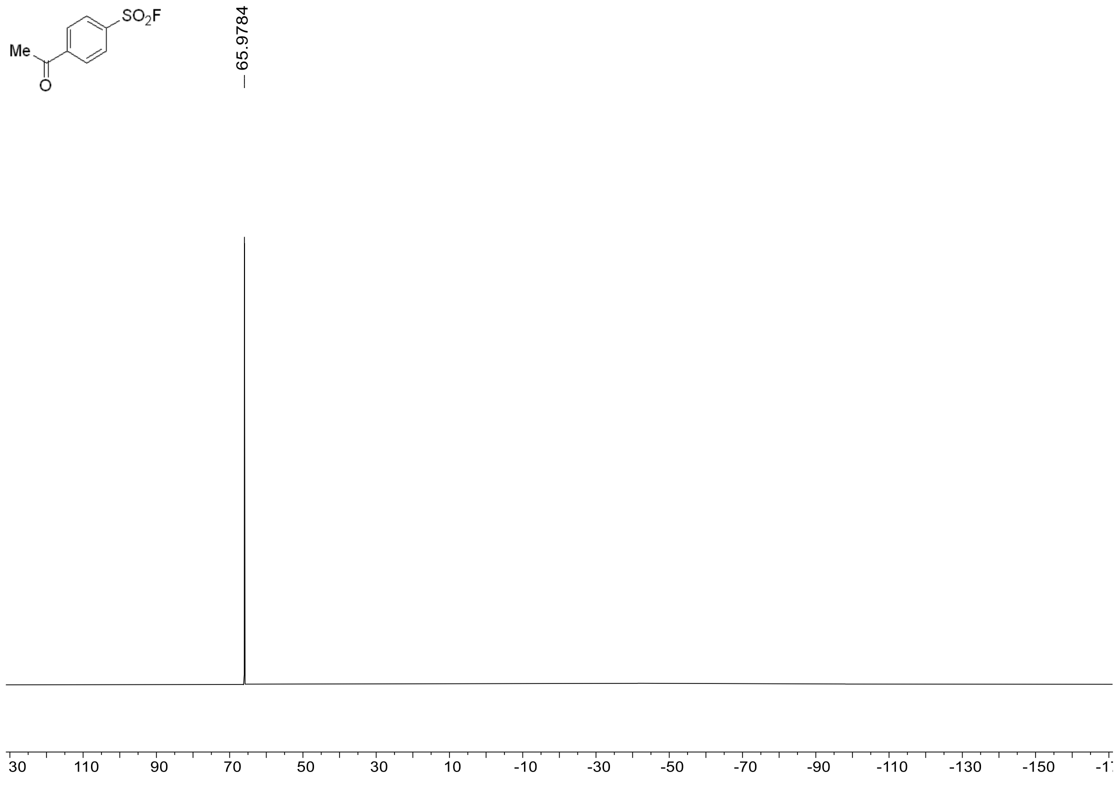

Methyl 4-(fluorosulfonyl)benzoate (27)

^{1}H-NMR Spectrum:

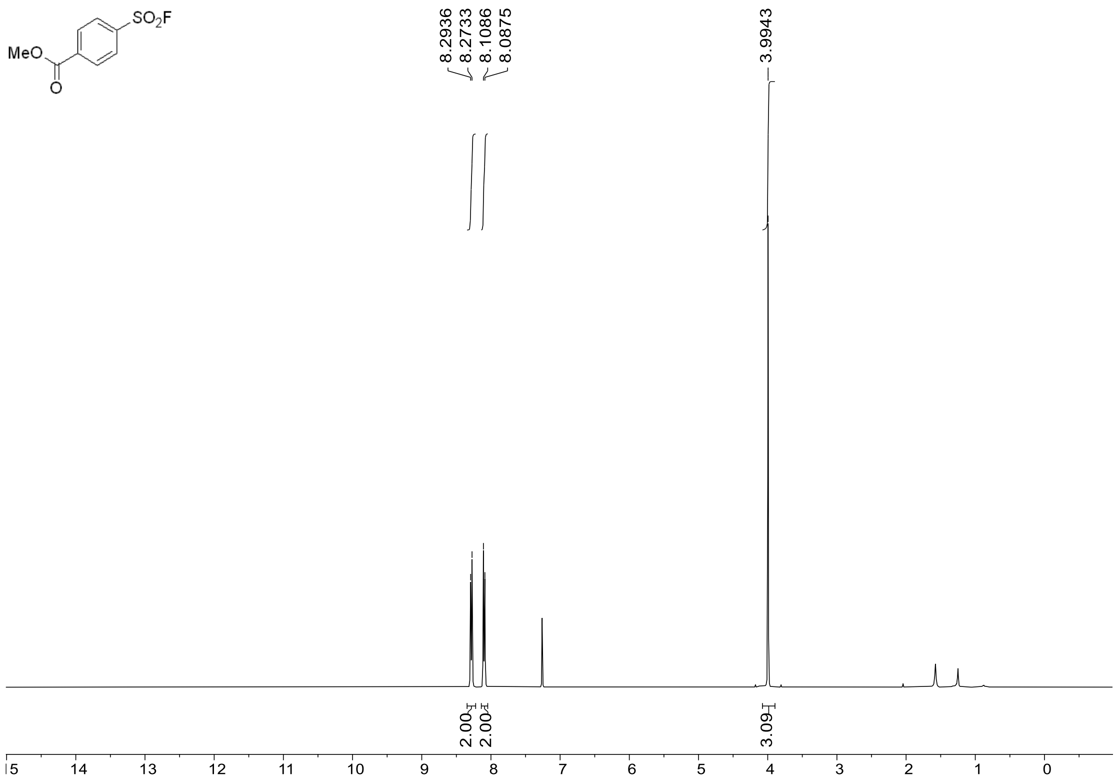

^{13}C-NMR Spectrum:

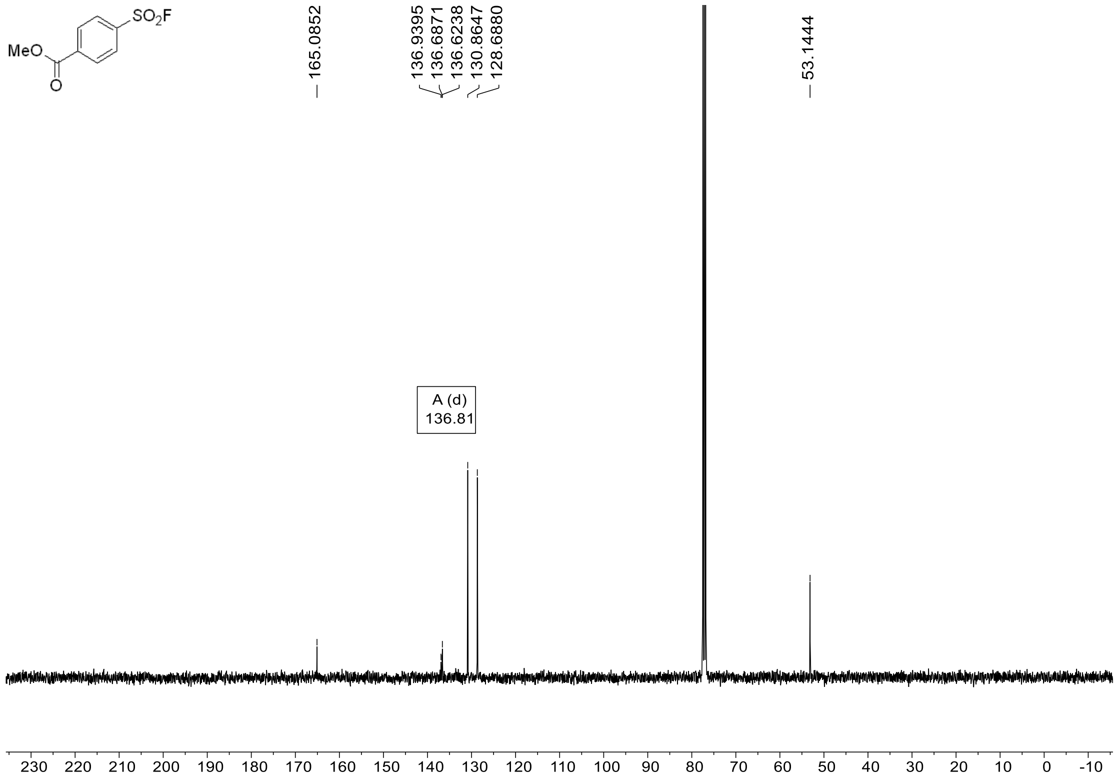

^{19}F-NMR Spectrum:

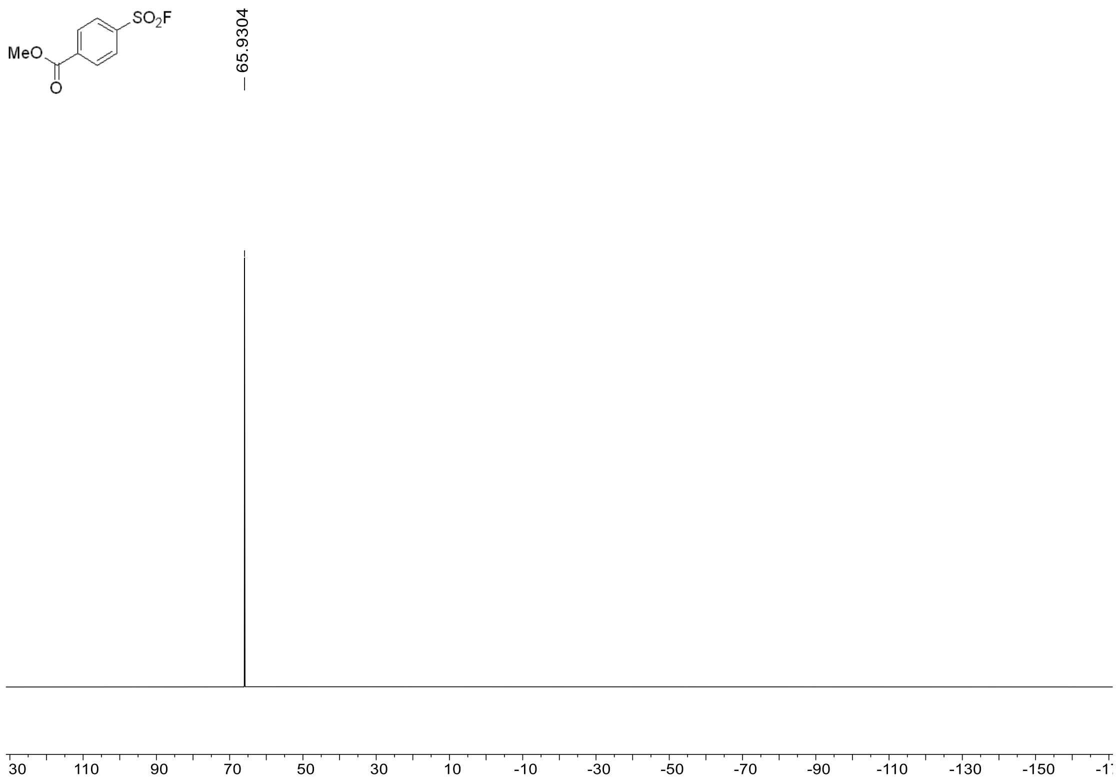

2-Methoxypyridine-3-sulfonyl fluoride (28)

^{1}H-NMR Spectrum:

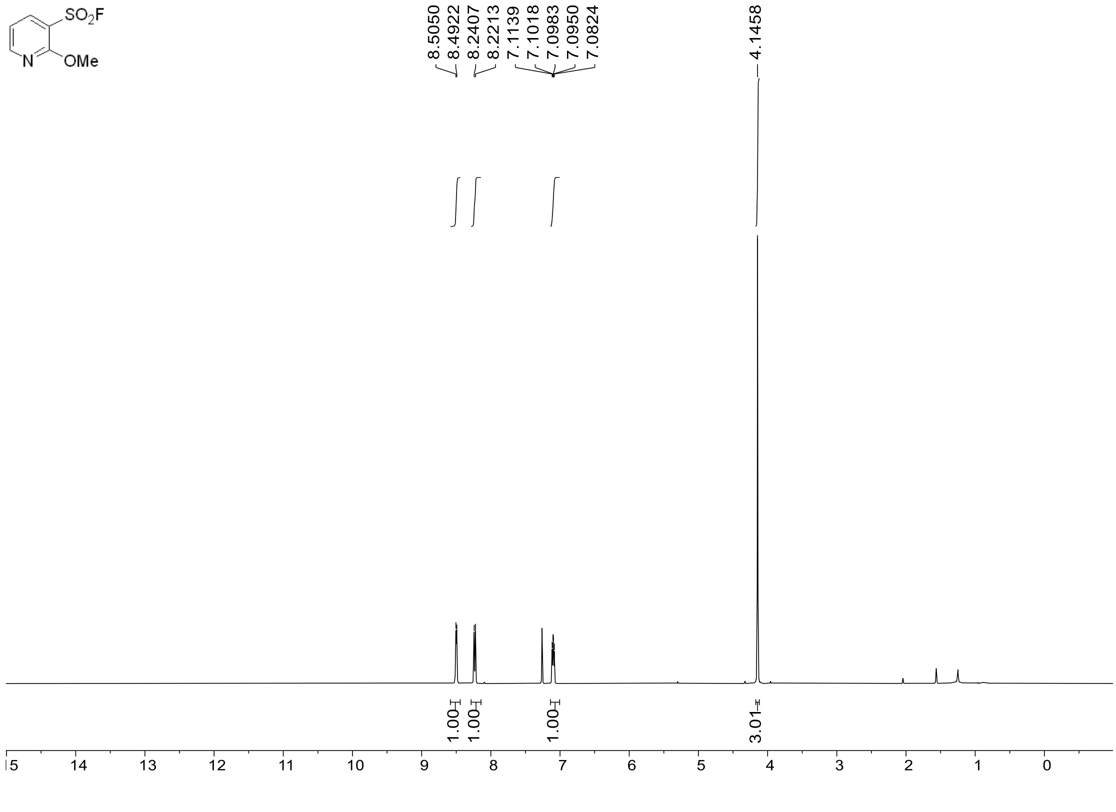

^{13}C-NMR Spectrum:

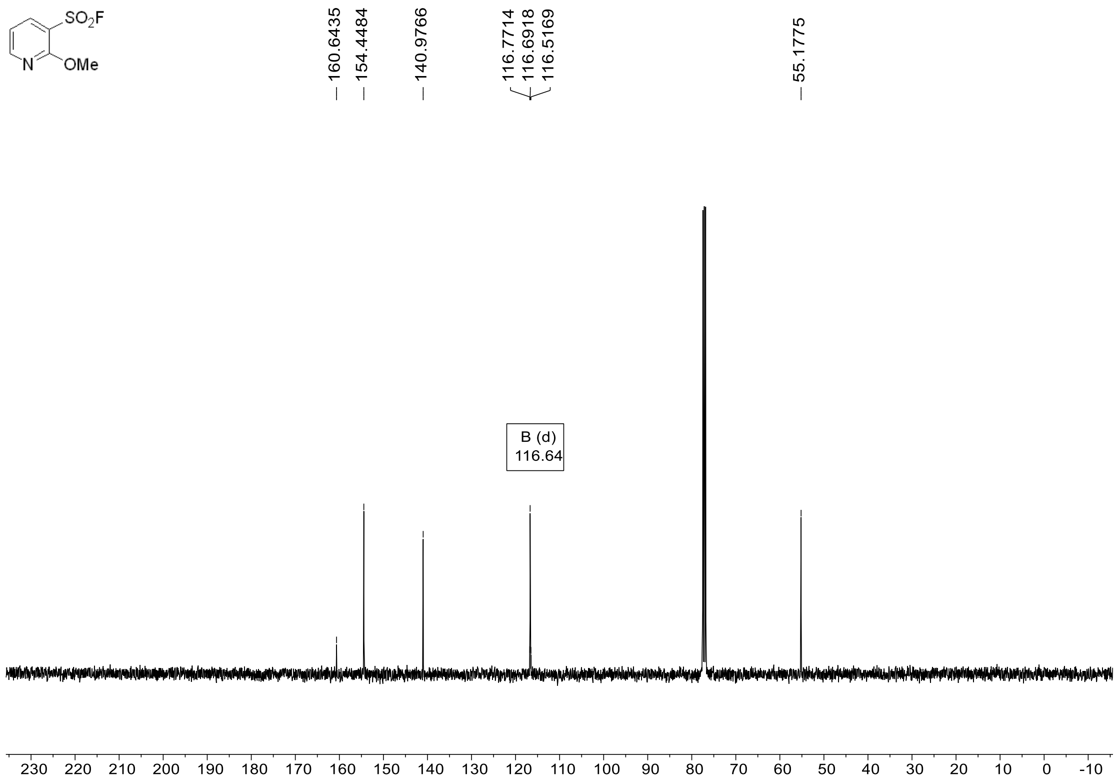

^{19}F-NMR Spectrum:

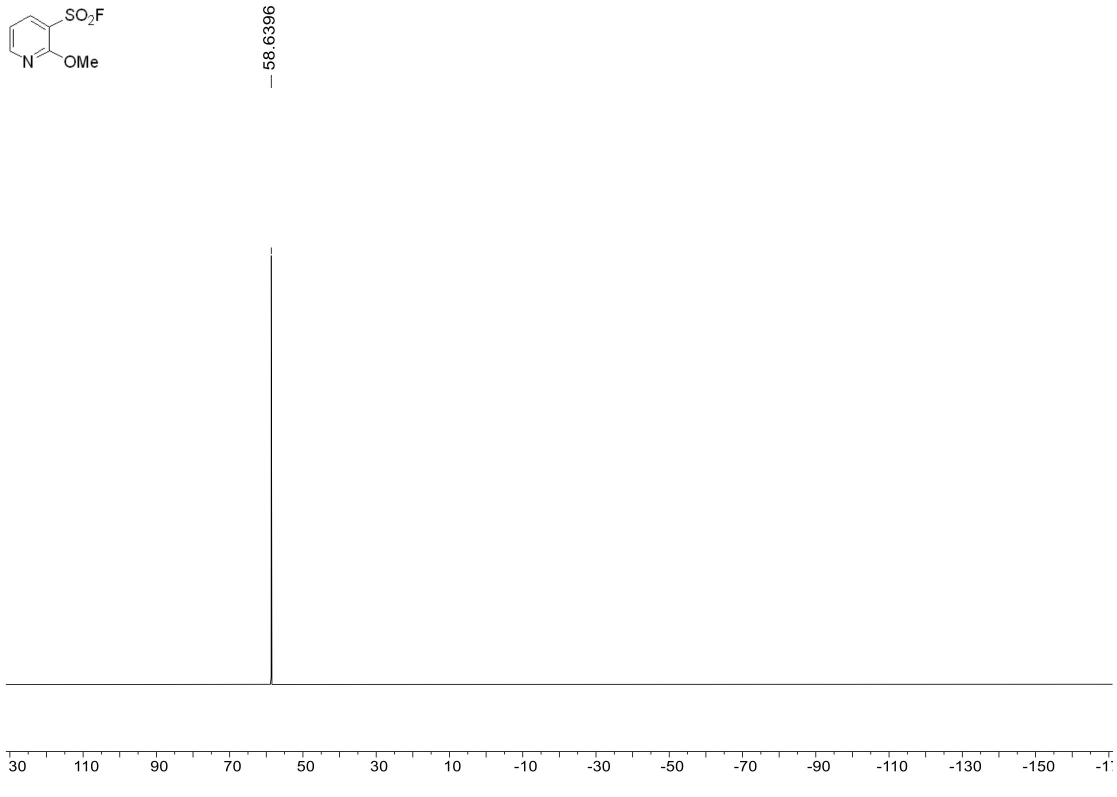

2-Aminopyridine-3-sulfonyl fluoride (29)

^{1}H-NMR Spectrum:

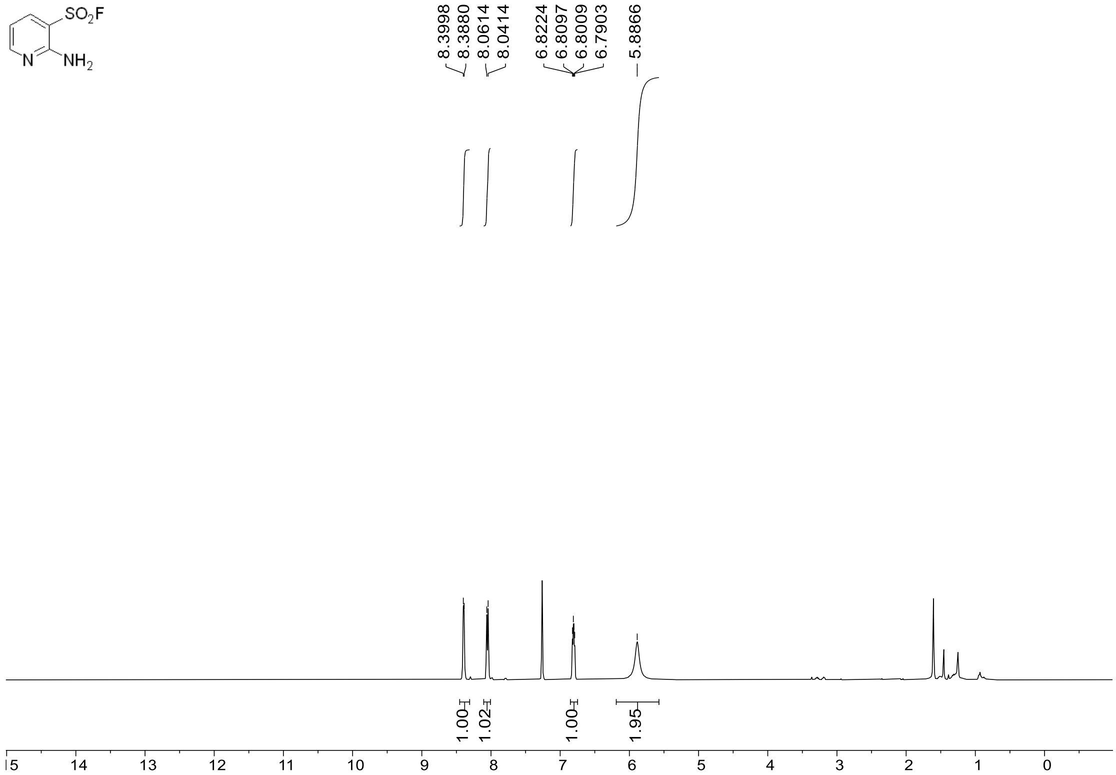

^{13}C-NMR Spectrum:

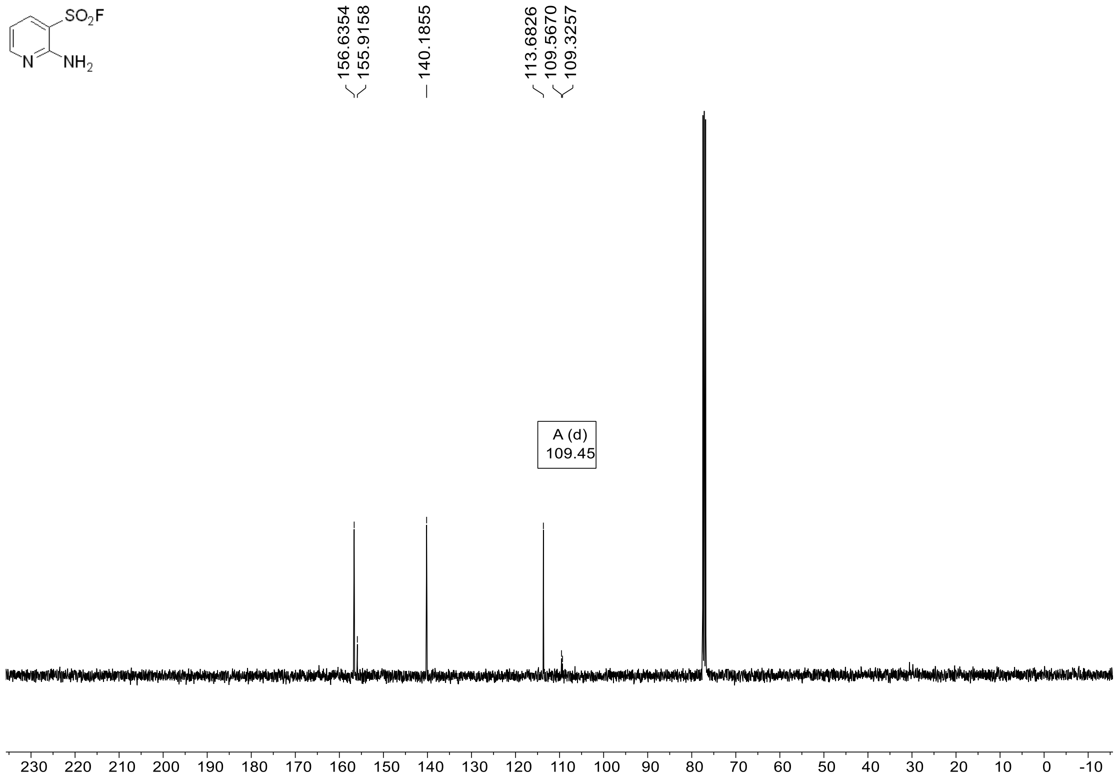

^{19}F-NMR Spectrum:

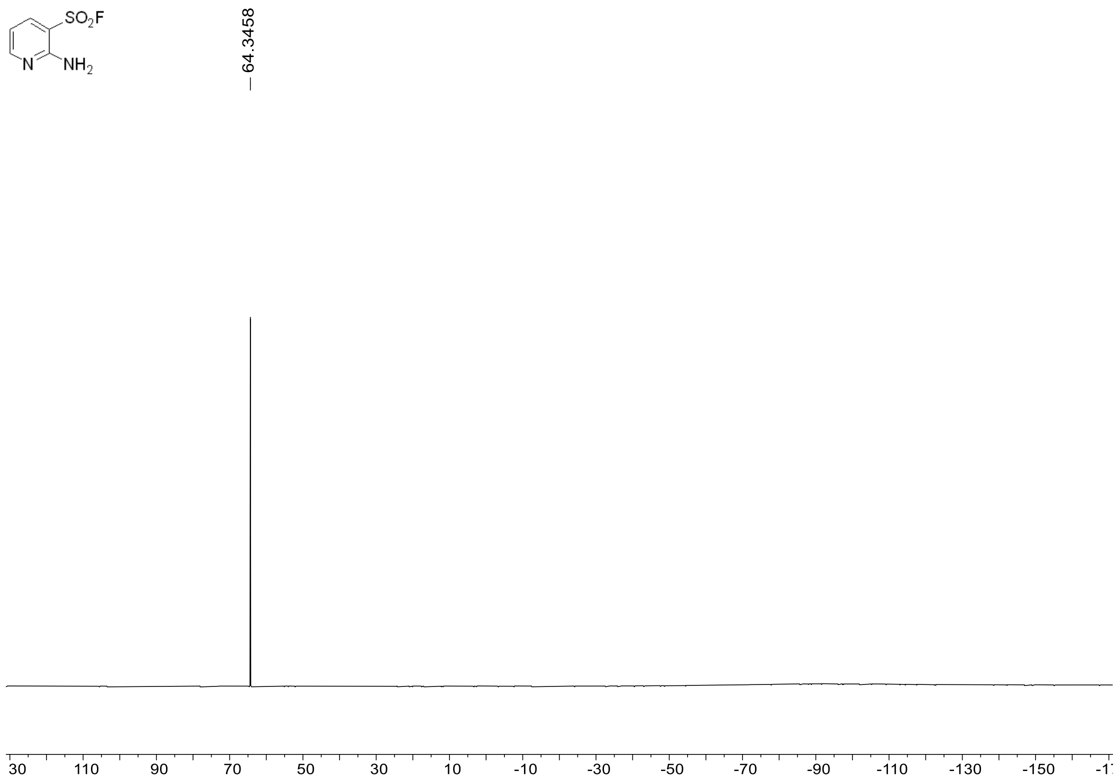

2-Methoxypyridine-4-sulfonyl fluoride (30)

^{1}H-NMR Spectrum:

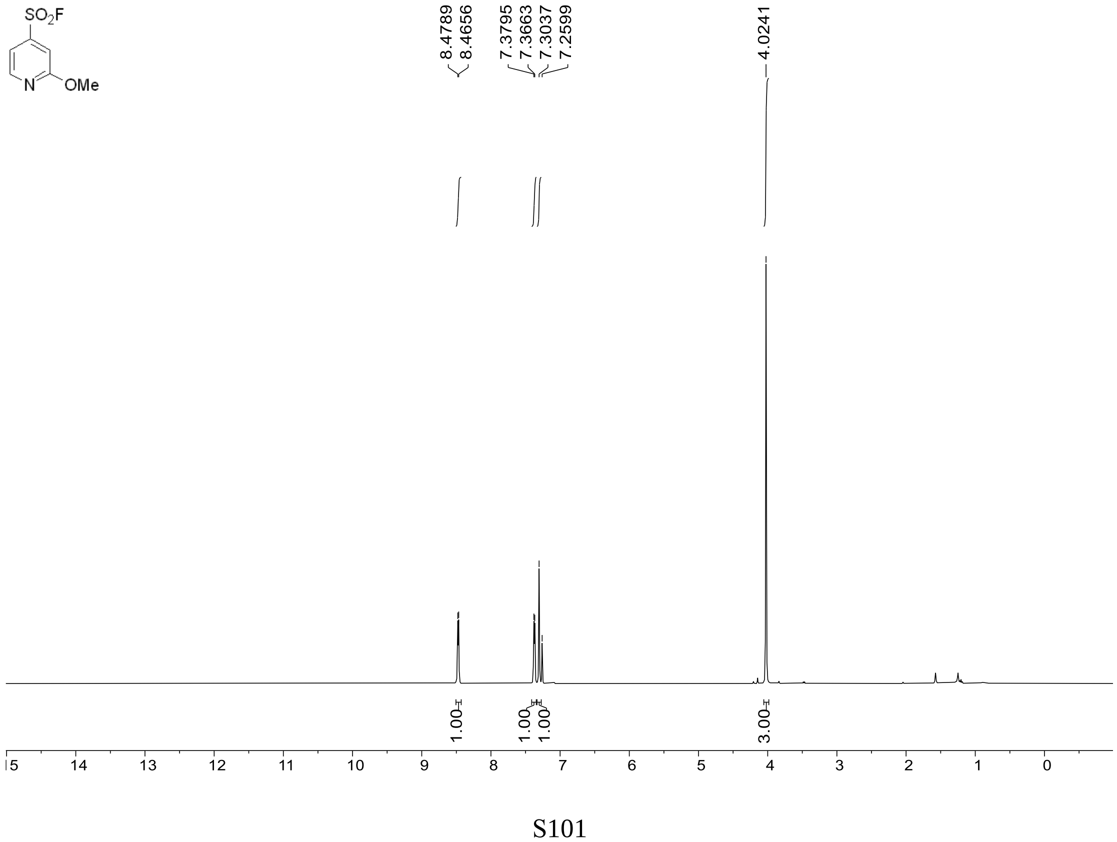

^{13}C-NMR Spectrum:

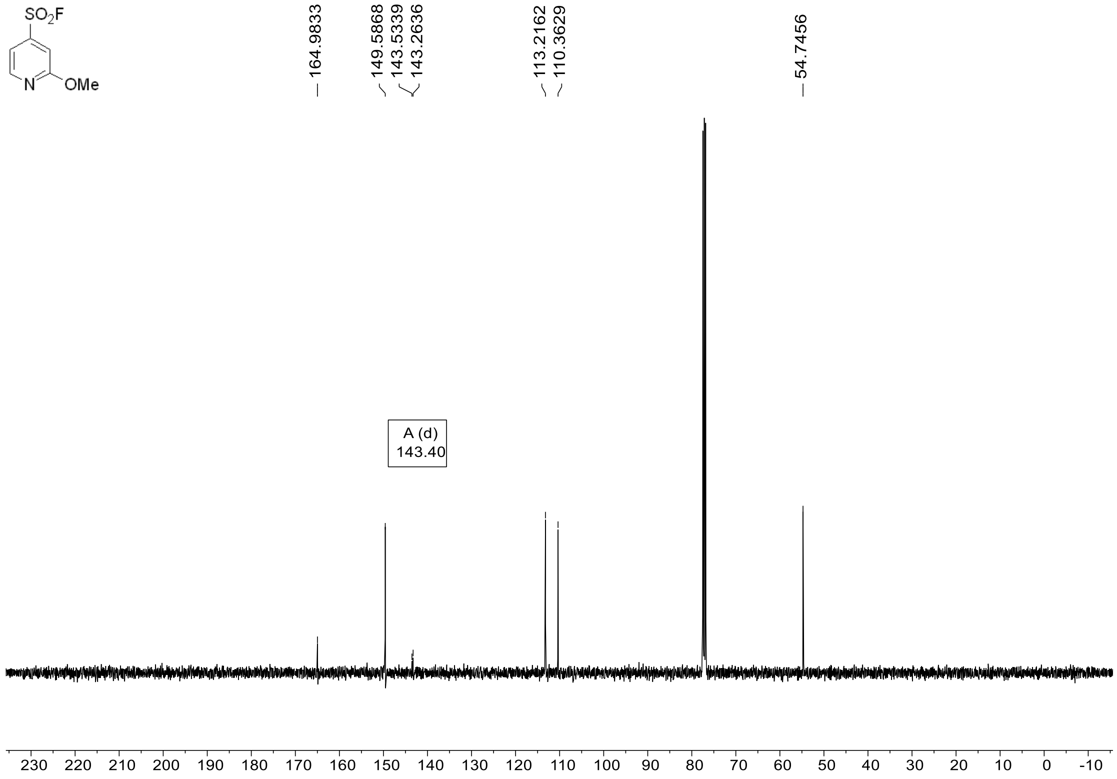

^{19}F-NMR Spectrum:

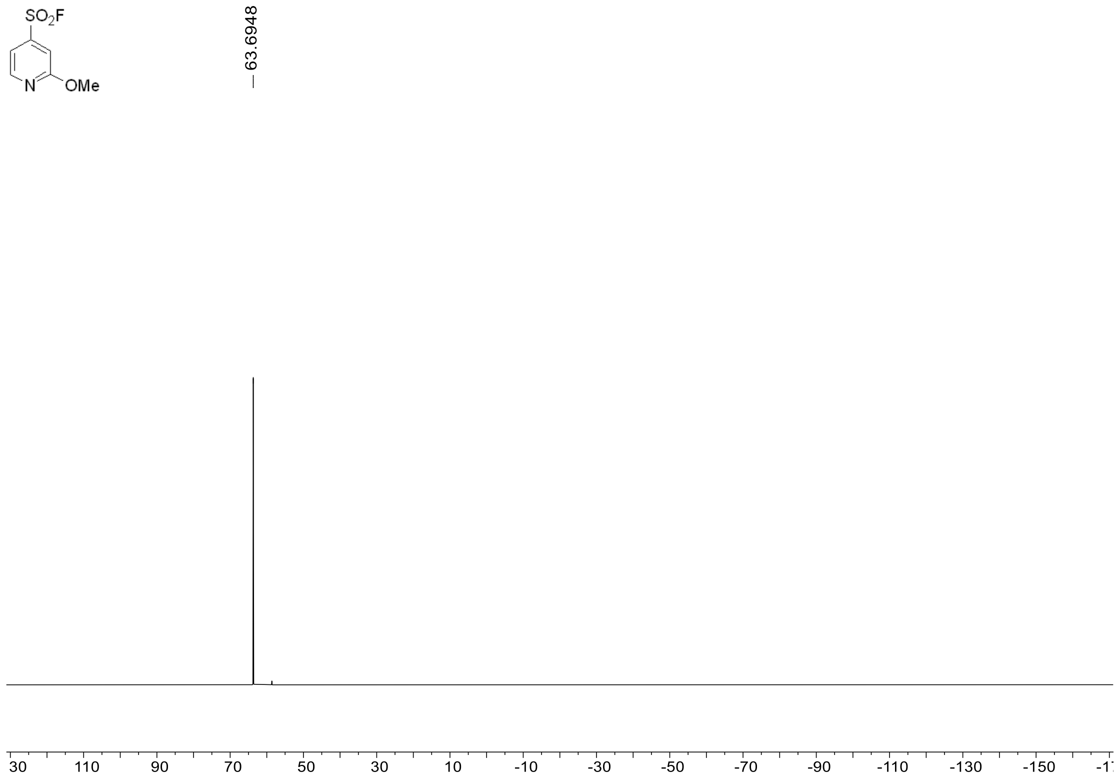

Tetrabutylammonium trifluoro(4-(fluorosulfonyl)phenyl)borate (39)

^{1}H-NMR Spectrum:

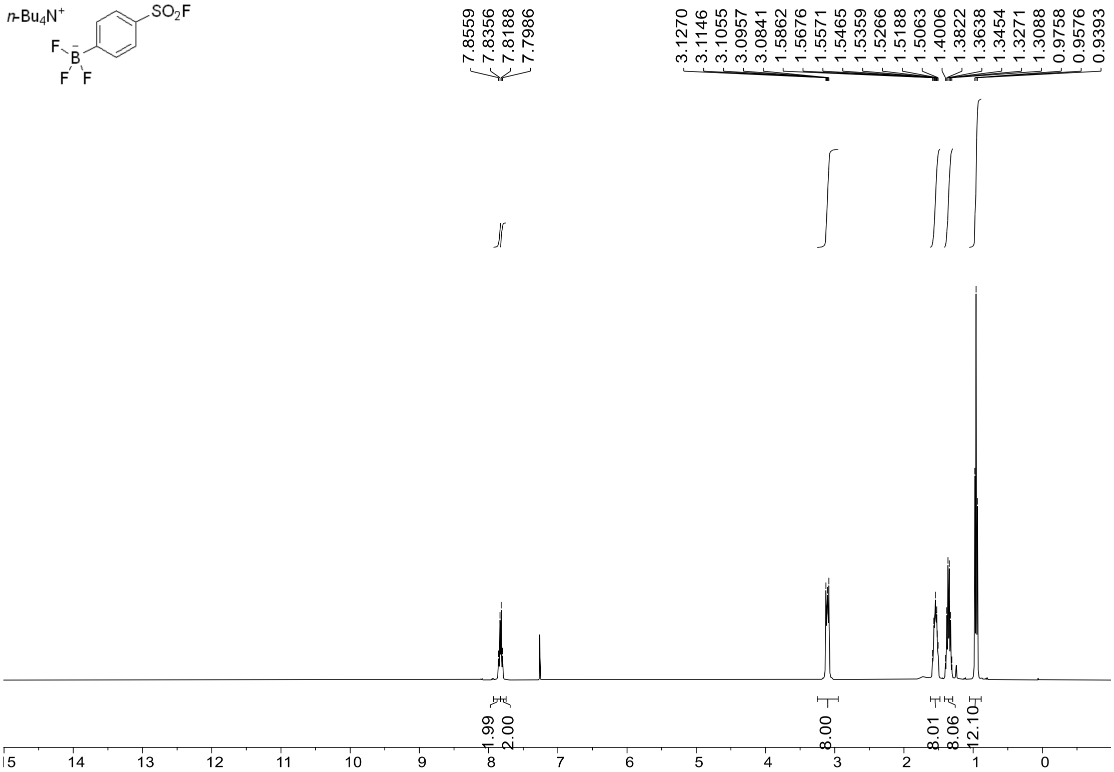

^{13}C-NMR Spectrum:

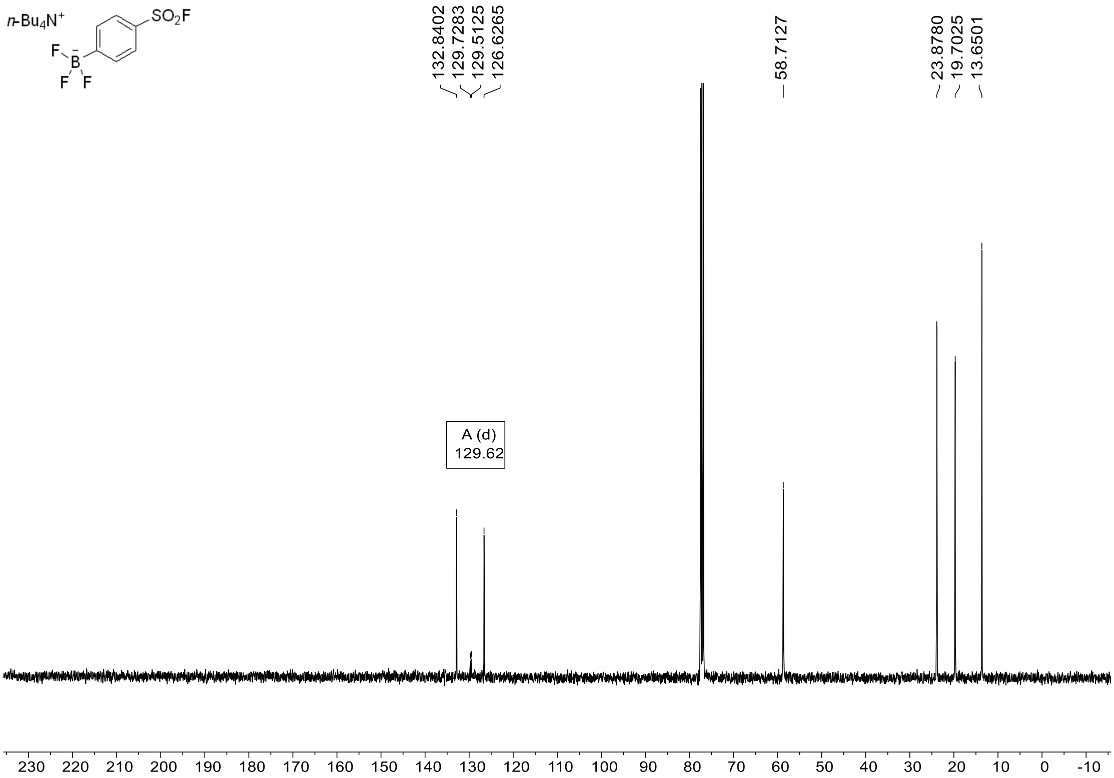

^{19}F-NMR Spectrum:

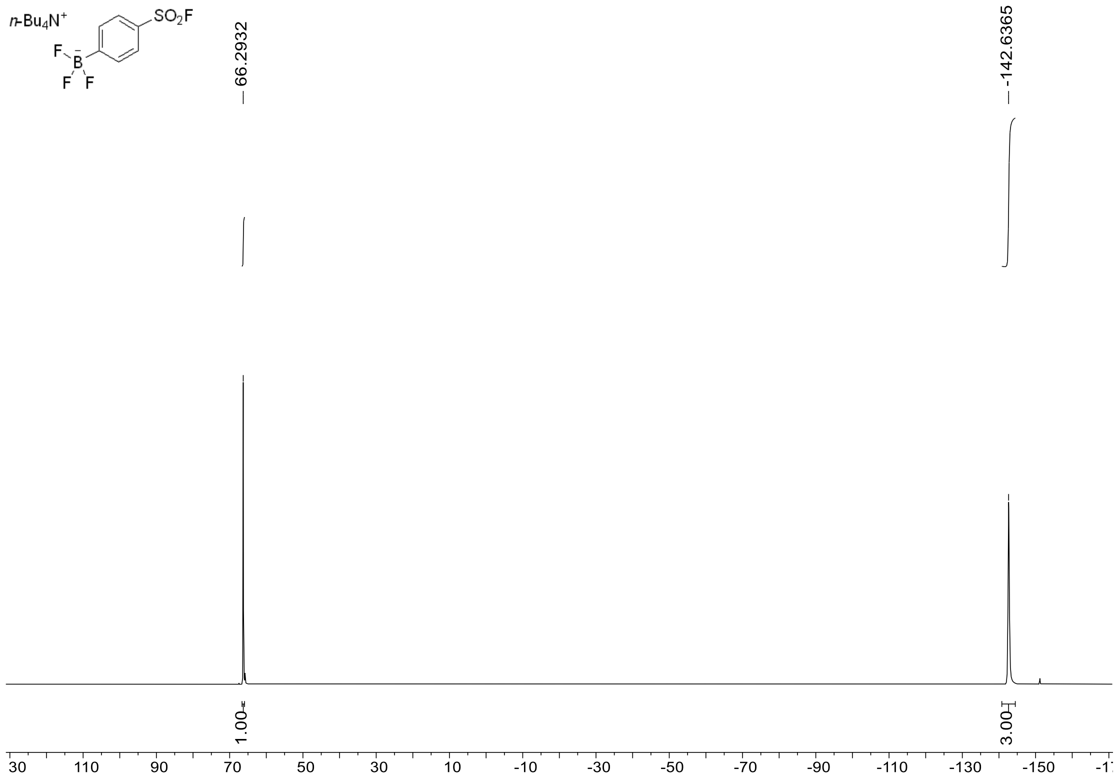

4,4,5,5-tetramethyl-2-(4-((4-methylbenzyl)sulfonyl)phenyl)-1,3,2-dioxaborolane (40)

^{1}H-NMR Spectrum:

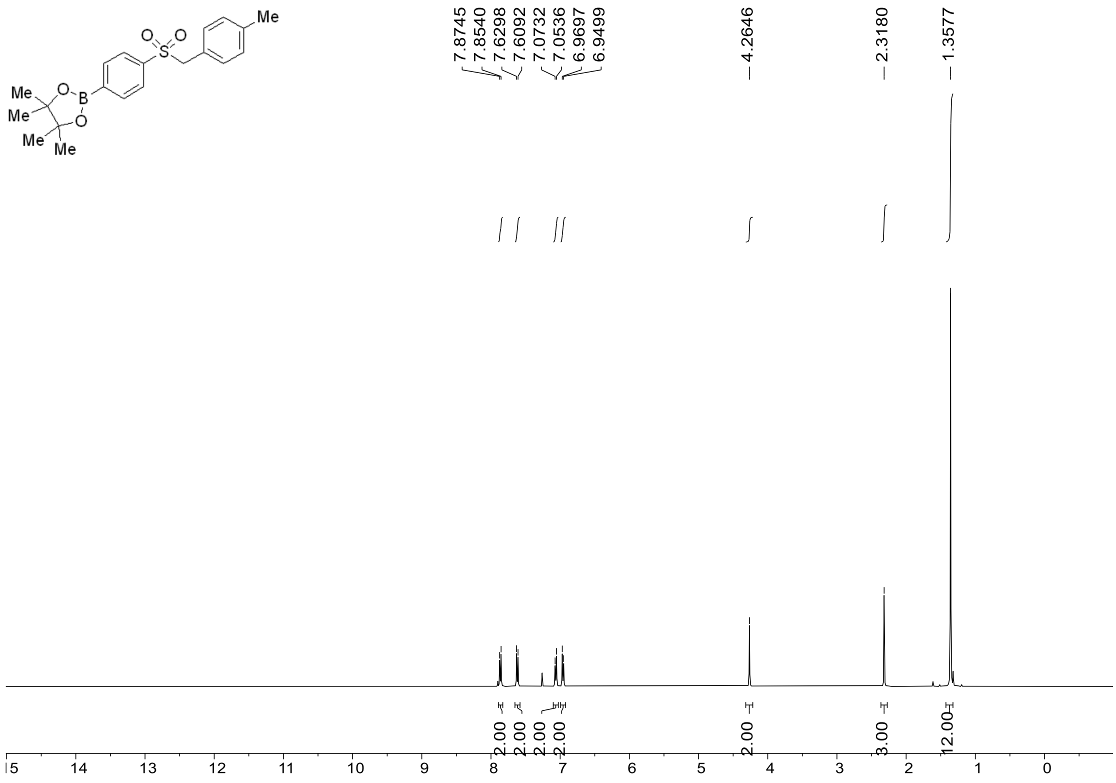

^{13}C-NMR Spectrum:

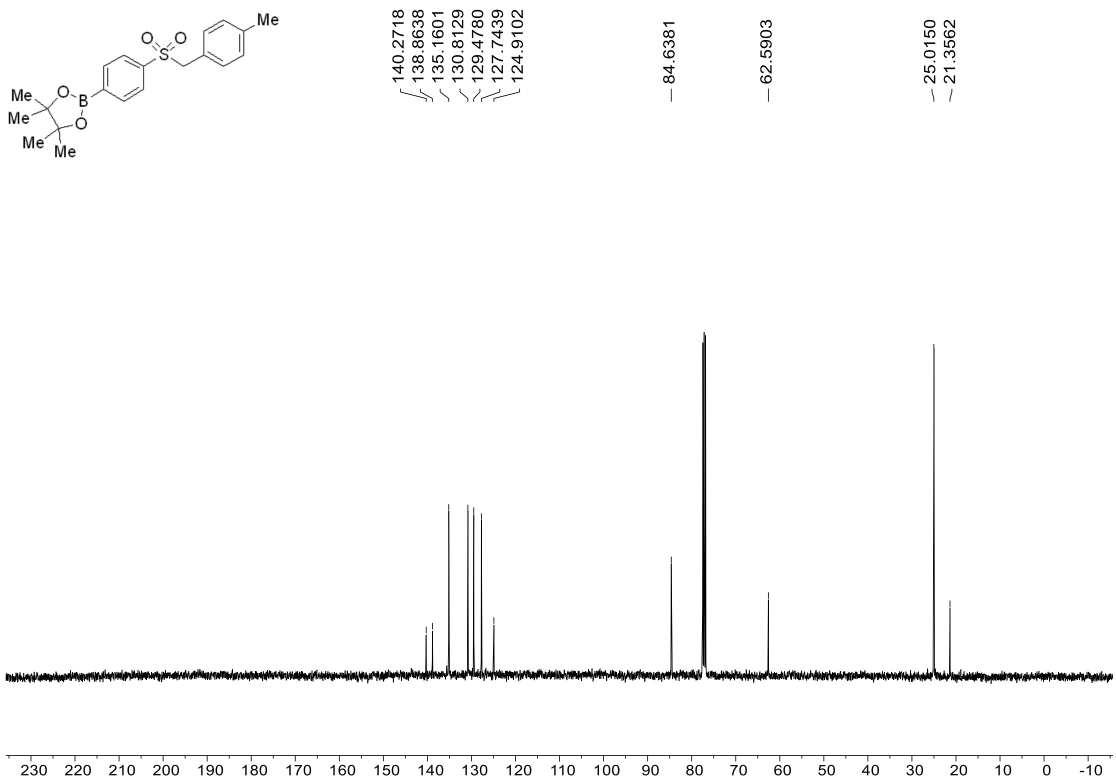

4-((4-(4,4,5,5-tetramethyl-1,3,2-dioxaborolan-2-yl)phenyl)sulfonyl)morpholine (41)

^{1}H-NMR Spectrum:

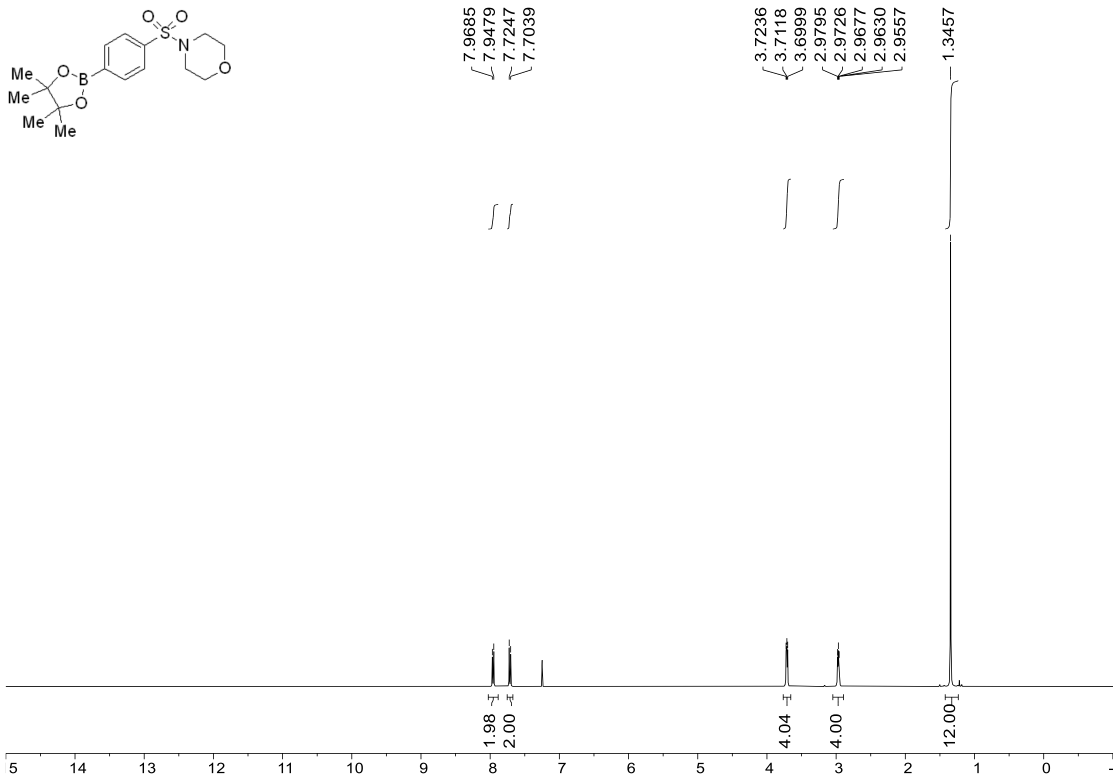

^{13}C-NMR Spectrum:

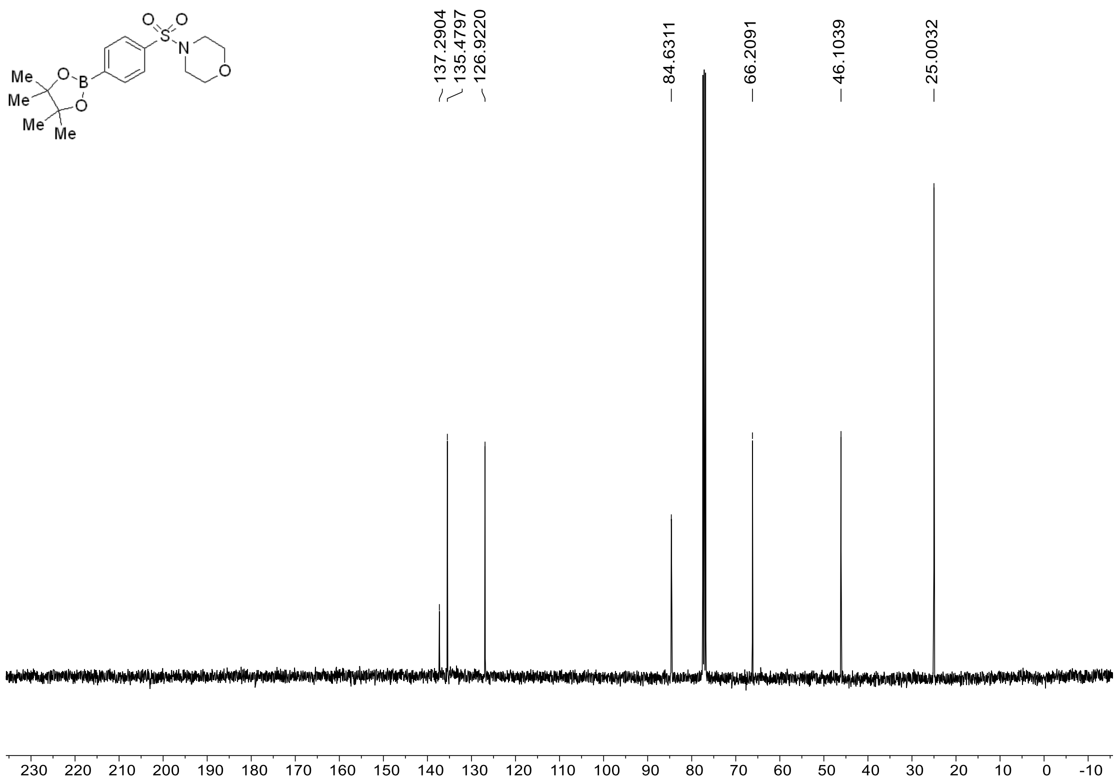

Supplement: Supplementary file 1 — Supporting Information [file ANIE-61-0-s001.pdf]
